# In Situ-Generated Formamidine as a Carbon/Nitrogen Source for Enaminone Formation: One-Pot Synthesis of Functionalized 4-Acyl-1,2,3-triazoles

**DOI:** 10.1021/acs.joc.4c01054

**Published:** 2024-08-19

**Authors:** Jia-Xin Lin, You-Xin Chen, Min-Cheng Chien, Hsiang-Jou Chen, Chian-Hui Lai, Chien-Fu Liang

**Affiliations:** † Department of Chemistry, National Chung Hsing University, Taichung 402, Taiwan; ‡ Graduate Institute of Biomedical Engineering, National Chung Hsing University, Taichung 402, Taiwan

## Abstract

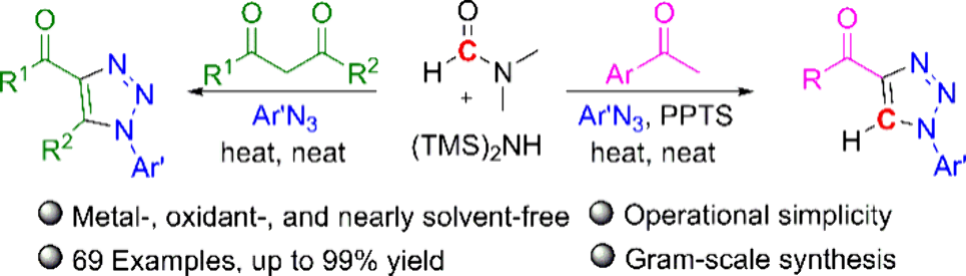

*N*,*N*-Dimethylformamide was reacted with hexamethyldisilazane to generate an *N*,*N*-dimethylformimidamide intermediate; thereafter, a reaction with acetophenones/β-diketones was induced to form enaminones. The one-pot synthetic protocol described in this paper can be applied to synthesize 1,4-disubstituted 1,2,3-triazoles and 1,4,5-trisubstituted 1,2,3-triazoles, in which organic azides are used as substrates under optimized conditions. Furthermore, this protocol uses readily available materials, is nearly free of solvent, can be applied to gram-scale operations, and leads to the formation of structurally diverse products with favorable yields.

## Introduction

Functionalized 1,2,3-triazoles play a critical role in numerous biological processes, especially in organic scaffolding, and they are frequently utilized in therapeutic medications.^1^ In addition, 4-acyl-1,2,3-triazoles are crucial N-heterocycles used in many fields, such as chemical synthesis,^2^ biology,^3^ medicinal chemistry,^4^ supramolecular chemistry,^5^ and materials science.^6^ However, synthesizing 4-acyl-1,2,3-triazoles is challenging. They are typically synthesized through the copper-catalyzed azide–alkyne cycloaddition of ynones, acetylenic carbinols, or acetylenic iminium salts to organic azides^7^ and the cyclization/oxidation of enones.^8^ These synthetic preparation methods can be tedious and have considerable limitations, including the risk of polymerization of ynones or enones and the lack of commercial availability of ynones/acetylene carbinols. Therefore, to overcome these limitations, the development of alternative methods under feasible and optimal conditions is highly desirable. In the literature, alternative methods that use readily available reagents to ensure optimal reaction conditions have been reported, such as an enaminone-based cycloaddition/elimination strategy,^9^ base-promoted cycloaddition of NH-based secondary enaminones and tosyl azide through the Regitz diazo transfer process,^10^ Lewis acid/base-catalyzed aerobic oxidative intermolecular cycloaddition of α/β-unsaturated or β/γ-unsaturated ketones,^11^ and copper-catalyzed C–C bond cleavage/reformation and cycloaddition from β-alkyl nitroalkanes.^12^ Despite their effectiveness, most of these methods require environmentally unfriendly agents or harsh conditions, such as high-cost catalysts, oxidative additives, corrosive reagents, substantial quantities of toxic solvents, and microwave/high operating temperatures, or their durations are long. Hence, developing direct, rapid, and environmentally friendly methods for the formation of 4-acyl-1,2,3-triazoles is an urgent need.

**Scheme 1:**
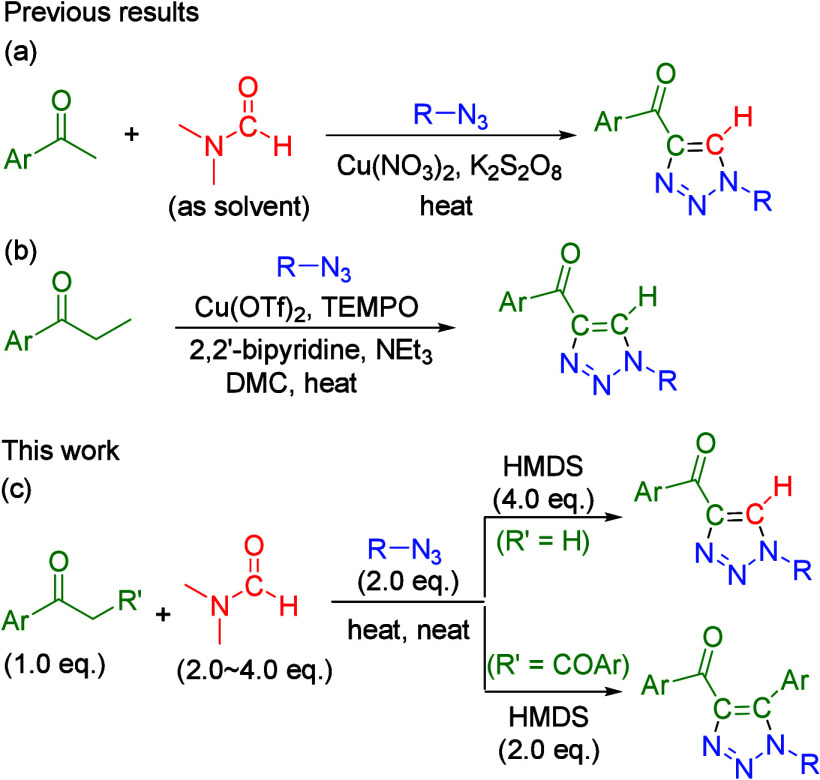
Direct Strategies for the Synthesis of 4-Acyl-1,2,3-triazoles

Some advances have been made in the direct synthesis of 4-acyl-1,2,3-triazoles, such as Cu-catalyzed oxidative dehydrogenation/cycloaddition of α-alkyl ketone with *N*,*N*-dimethylformamide (DMF) as a C1 donor (Scheme 1a)^13^ and Cu/TEMPO-catalyzed tandem multiple oxidative dehydrogenation/cycloaddition of β-alkyl ketone (Scheme 1b).^14^ Although these strategies have been used to successfully synthesize 4-acyl-1,2,3-triazoles using readily available aryl alkyl ketones, they require expensive transition metal catalysts and oxidants. In a previous study, as an alternative method, the authors proposed DMF as a C1 source for the formation of *N*-sulfonyl/aryl formamidine.^15^ On the basis of our success in utilizing DMF as a C1 source, as a method for the synthesis of 4-acyl-1,2,3-triazoles, we hypothesize that the direct condensation of α-methyl ketone with stoichiometric amounts of DMF would generate enaminones in situ. In the work presented here, we report a one-pot synthetic protocol for directly converting α-methyl ketones into enaminones under metal-free and oxidant-free conditions, and in this reaction, several functionalized azides are used as substrates to produce 1,4-disubstituted 1,2,3-triazoles (Scheme 1c). Moreover, this protocol can be applied for enaminone formation by using β-diketones as substrates to produce 1,4,5-trisubstituted 1,2,3-triazoles (Scheme 1c).

## Results and Discussion

In this study, we optimized the formation of 4-acyl-1,2,3-triazoles by using methyl phenyl ketones (**1a**) and phenyl azides (**3a**) with stoichiometric amounts of hexamethyldisilazane (HMDS) and DMF under heating conditions (Table 1). Initially, 4 equiv of HMDS and DMF agents were employed, which resulted in poor conversion (entry 1). However, a previous study produced enaminone in situ by using aryl methyl ketone with the dimethyl acetal of DMF in the presence of acidic additives, which exhibited increased reactivity;^16^ thus, reaction conversion can be optimized through this approach. Accordingly, in this study, the reaction conditions for the one-pot multicomponent synthetic protocol were optimized using catalytic amounts of acidic additives, improving the yield. The following acidic additives were employed: camphorsulfonic acid (CSA), boron trifluoride diethyl etherate (BF_3_·OEt_2_), pyridinium *p*-toluenesulfonate (PPTS), lanthanum(III) trifluoromethanesulfonate [La(OTf)_3_], and *p*-toluenesulfonic acid (Table 1, entries 2–6, respectively). We observed that the reaction proceeded favorably in the presence of a PPTS catalyst (entry 4), with a yield of 78% for the cycloaddition product (**4aa**). Therefore, PPTS was selected as the acid catalyst for further reactions. Subsequently, 3 and 5 equiv of HMDS and DMF agents were utilized to determine the optimal quantity of these reactants (Table 1, entries 7 and 8, respectively). The results revealed that entry 4 was the minimum amount of HMDS and DMF agents required for optimal activity. Additionally, when the amount of the PPTS catalyst was reduced 5-fold under optimized conditions, yields of 86% were obtained (entry 9). Moreover, when the quantities of phenyl azides (**3a**) were reduced to 1.2 equiv, the yield decreased (entry 10). Furthermore, we optimized the reaction temperature. Higher temperatures resulted in higher reaction yields, and the results revealed that the reaction proceeded most favorably at 120 °C (entry 11). Moreover, to investigate the formation of the enaminone intermediate using DMF as a C1 source, we utilized β-diketone (**2a**) under these optimized acid and temperature conditions. Although we expected to obtain 4,5-diacyl-1,2,3-triazole, we instead obtained a 4-acyl-1,2,3-triazole product. This product was a 1,4,5-trisubstituted 4-acyl-1,2,3-triazole (**5aa**), which was confirmed by ^1^H nuclear magnetic resonance (NMR) spectroscopy. This result suggests that the reaction route for the formation of the enaminone intermediate from β-diketone may differ from that of the α-methyl ketone. Nevertheless, after systematically evaluating the optimized conditions (entries 12–15), we found that the reaction proceeded favorably with reduced amounts of HMDS and DMF agents in the absence of the PPTS catalyst, providing a yield of 91% of the desired 1,4,5-trisubstituted 1,2,3-triazole (**5aa**) (entry 15).

**Table 1:**
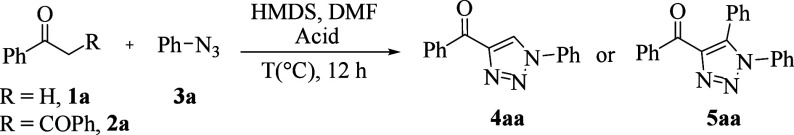
Optimized Conditions

entry^a^	HMDS (equiv)	DMF (equiv)	acid (equiv)	*T* (°C)	yield^b^ (%)
1	4	4	–	100	trace
2	4	4	CSA (0.5)	100	66
3	4	4	BF_3_·OEt_2_ (0.5)	100	trace
4	4	4	PPTS (0.5)	100	78
5	4	4	La(OTf)_3_ (0.01)	100	72
6	4	4	PTSA (0.5)	100	73
7	3	3	PPTS (0.5)	100	68
8	5	5	PPTS (0.5)	100	76
9	4	4	PPTS (0.1)	100	86
10^c^	4	4	PPTS (0.1)	100	69
11	4	4	PPTS (0.1)	120	92
12^d^	4	4	PPTS (0.1)	120	54
13^d^	4	4	–	120	53
14^d^	3	3	–	120	70
15^d^	2	2	–	120	91

a For the reactions, **1a** (0.83 mmol, 1.0 equiv) and **3a** (2.0 equiv) were reacted under a nitrogen atmosphere.

b Isolated yield.

c With 1.2 equiv of phenyl azide **3a**.

d Using **2a** (0.89 mmol, 1.0 equiv) as a reactant.

**Scheme 2:**
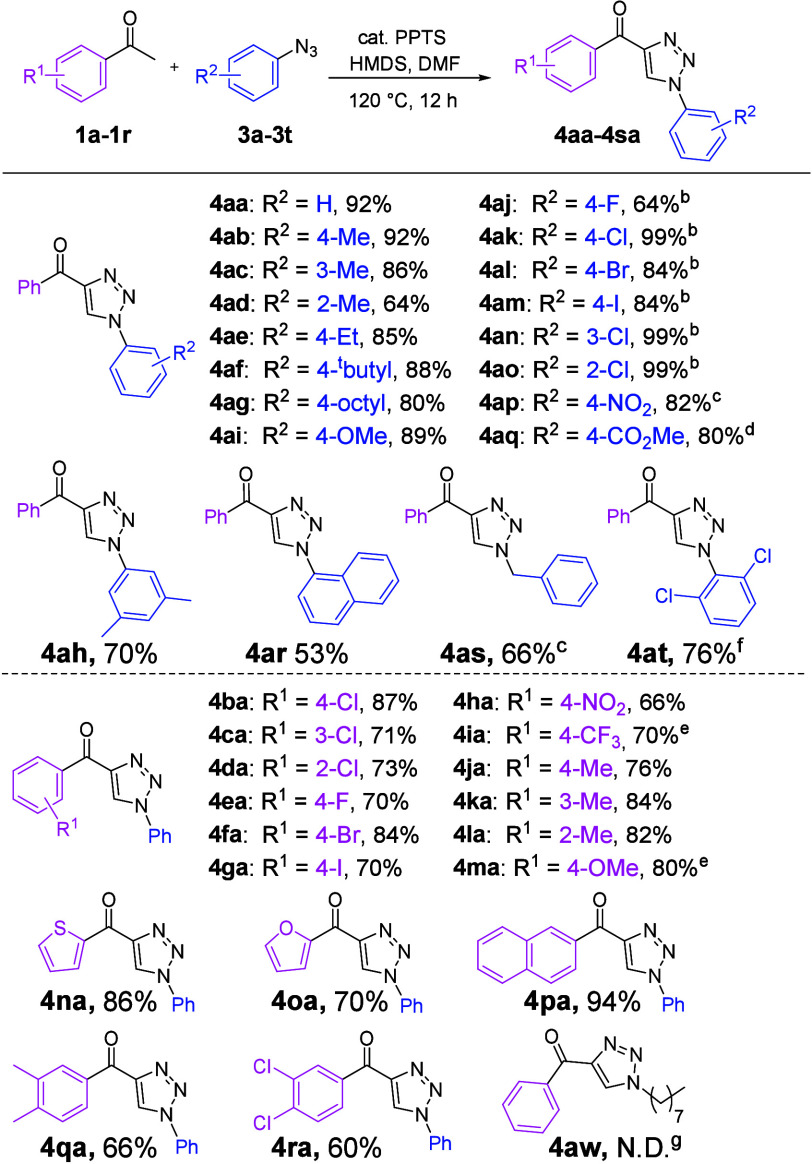
Formation of 4-Acyl-1,2,3-triazoles Using Various Aryl Methyl Ketones and Azides For the reactions, **1** (0.40–0.83 mmol, 1.0 equiv), HMDS (4.0 equiv), DMF (4.0 equiv), **3** (2.0 equiv), and PPTS (0.1 equiv) were stirred at 120 °C under a nitrogen atmosphere for 12 h. Reaction time of 2 h. For the reaction, **1a** (0.50 mmol for **3p** or 0.83 mmol for **3s**, 1.0 equiv), HMDS (4.0 equiv), and DMF (4.0 equiv) were stirred at 120 °C; after reaction for 8 h, **3p** or **3s** (2 equiv) was added to the reaction mixture for an additional 12 h. Reaction time of 4 h. With 3.0 equiv of phenyl azide. Reaction time of 6 h. The reaction produced enaminone **12** in 42% yield, including some unrecognized spots.

Subsequently, the optimized conditions for the direct one-pot multicomponent reaction were employed in the synthesis of a series of functionalized 4-acyl-1,2,3-triazoles, which involved the reaction of HMDS and DMF agents with diverse aryl methyl ketones (**1a**–**1r**) and organic azides (**3a**–**3t**) (Scheme 2). In the synthesis with aryl azides bearing electron-donating groups [methyl, ethyl, *tert*-butyl, octyl, methoxy, or naphthyl (**3a**–**3i** and **3r**)], the desired products (**4aa–4ai** and **4ar**, respectively) were formed in 53–92% yields. Furthermore, a reaction with aryl azides having electron-withdrawing groups (F, Cl, Br, I, NO_2_, or CO_2_Me, **3j**–**3***q***/3t**) resulted in yields of 64–99%. Moreover, aryl azides bearing *ortho*-substituted groups (**3d**, **3o**, and **3t**) produced **4ad**, **4ao**, and **4at** in 64%, 99%, and 76% yields, respectively. These results indicate that steric effects had a limited influence on the reactivity of aryl azides. Additionally, under optimized conditions, benzyl azide (**3s**) achieved a satisfactory yield of product **4as**. Subsequently, we applied the optimized conditions to functionalized aryl methyl ketones **1b**–**1r** with phenyl azides (**3a**) (Scheme 2). Various functionalized aryl methyl ketones were converted into the corresponding 4-acyl-1,2,3-triazole adducts (**4ba**–**4ra**, respectively), with reaction yields of 60–94%. The ease of reaction conversion and the high yields for the aryl system indicate its high functional group tolerance; chloro (**4ba**–**4da**), fluoro (**4ea**), bromo (**4fa**), iodo (**4ga**), nitro (**4ha**), trifluoro (**4ia**), methyl (**4ja**–**4la**), methoxy (**4ma**), thiophene (**4na**), furan (**4oa**), and naphthyl (**4pa**) products were all obtained at comparably high yields under optimized conditions. Moreover, substrates **1q** and **1r**, which contained disubstituted electron-donating and -withdrawing motifs in the aryl system, generated products **4qa** and **4ra** in 66% and 60% yields, respectively. However, this method proved to be unsuccessful in 1,4-disubstituted triazole formation using alkyl azide (**4aw**). According to a previous study of 4-acyl-1,2,3-triazole formation from an enaminone intermediate under metal-free conditions,^9^ the major issues for such reactions are the high reaction temperatures under microwave operating conditions required for the formation of the enaminone intermediate and the requirement of substantial amounts of toxic solvents in the cycloaddition step. The method proposed in this study overcomes these limitations and provides a green synthetic protocol for synthesizing 4-acyl-1,2,3-triazole.

**Scheme 3:**
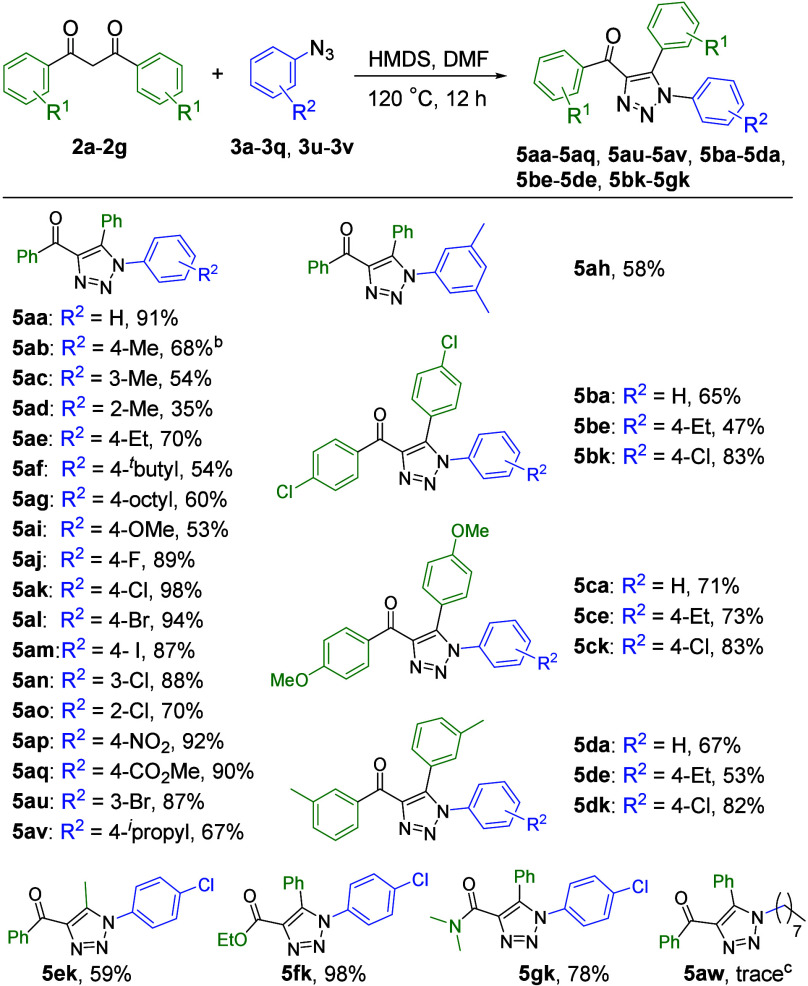
Formation of 4-Acyl-1,2,3-triazoles Using Various β-Diketones and Azides For the reactions, **2** (0.50–0.89 mmol, 1 equiv), **3** (2 equiv), HMDS (2 equiv), and DMF (2 equiv) were stirred at 120 °C under a nitrogen atmosphere for 12 h. For the reaction, **2a** (0.89 mmol, 1 equiv), **3b** (2 equiv), HMDS (2 equiv), and DMF (2 equiv) were stirred at 120 °C; after reaction for 6 h, **3b** (2 equiv) was add to the reaction mixture for an additional 6 h. The reaction afforded **2a** (40% conversion) and produced intermediate **15** in 90% yield (recovery yield).

We subsequently attempted to use β-diketone to perform cycloaddition under optimized conditions and obtained 1,4,5-trisubstituted 4-acyl-1,2,3-triazole derivatives (Scheme 3), indicating the broad application of the protocol proposed in this study for the formation of 4-acyl-1,2,3-triazole through the in situ generation of enaminone intermediates. Furthermore, we performed cycloaddition reactions of β-diketones (**2a**–**2d**) with a range of functionalized aryl azides (**3a**–**3q**, **3u**, and **3v**) under optimized conditions. This transformation method produced yields of 35–98% for the desired products (**5aa**–**5aq**, **5au**, **5av**, **5ba–5da**, **5be–5de**, and **5bk**–**5dk**). However, treatment of the *ortho*-substituted aryl azide (**3d**) with **2a** under optimized conditions formed **5ad**, with a reaction yield of only 35%; this may be caused by steric hindrance. These results revealed that the reaction yields of aryl azides bearing electron-withdrawing substituents (**3j**–**3q** and **3u**) were higher than those of aryl azides bearing electron-donating substituents (**3b**–**3i** and **3v**). In a previous study of 1,3-dipolar cycloaddition,^9c^ organic azides behaved as electrophiles, which demonstrated that the azides with electron-withdrawing groups induce rapid reactions and thus produce superior yields. Accordingly, we also tested the DMF/HMDS method developed in the study presented here on 1,3-dicarbonyl derivatives (**2e**–**2g**), which have been infrequently used in previous studies.^7−14,16^ Our results revealed that the method developed in the study presented here exhibits high functional group tolerance with respect to the resulting products, specifically the 5-methyl (**5ek**), 4-ester (**5fk**), and 4-amide (**5gk**) 1,2,3-triazole compounds. However, this method proved to be unsuccessful for 1,4,5-trisubstituted triazole formation using an alkyl azide (**5aw**).

**Scheme 4:**
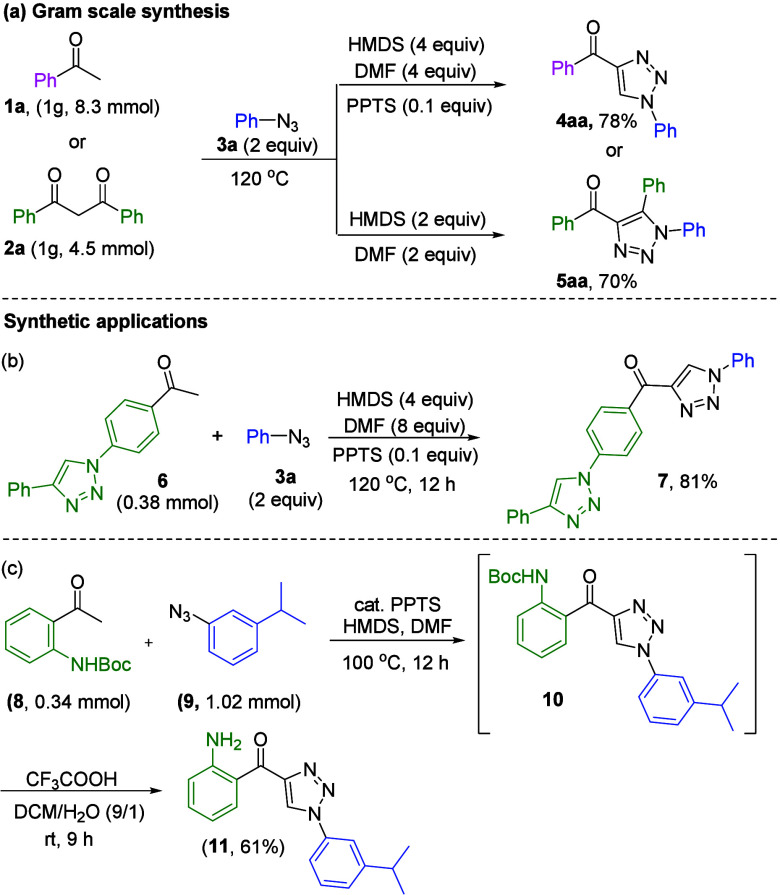
Gram-Scale Reaction and Synthetic Applications

In the next phase of the study, we employed methyl phenyl ketone (**1a**), β-diketone (**2a**), and phenyl azide (**3a**) for the synthesis of 4-acyl-1,2,3-triazole as a means of enhancing the scalability of the proposed synthetic method. Subsequently, we verified the approach in gram-scale synthesis with yields of 78% and 70% for products **4aa** and **5aa**, respectively (Scheme 4a). Inspired by the broad applicability of this methodology, we further exemplified this by synthesizing a bis-triazole **7** [81% (Scheme 4b)]. The practical potential of the 4-acyl-1,2,3-triazole structure may lie in the synthesis of key pharmaceutical compounds. The structure of compound **11** is a crucial suppressor of estrogen-related receptor α for the therapeutic treatment of breast cancer.^3a^ Under optimal conditions, it was smoothly converted into *N*-Boc-protected 4-acyl-1,2,3-triazole **10**. Subsequently, the Boc protecting group of compound **10** was replaced with an amino group, yielding ERRα suppressor compound **11** [61% (Scheme 4c)].

**Scheme 5:**
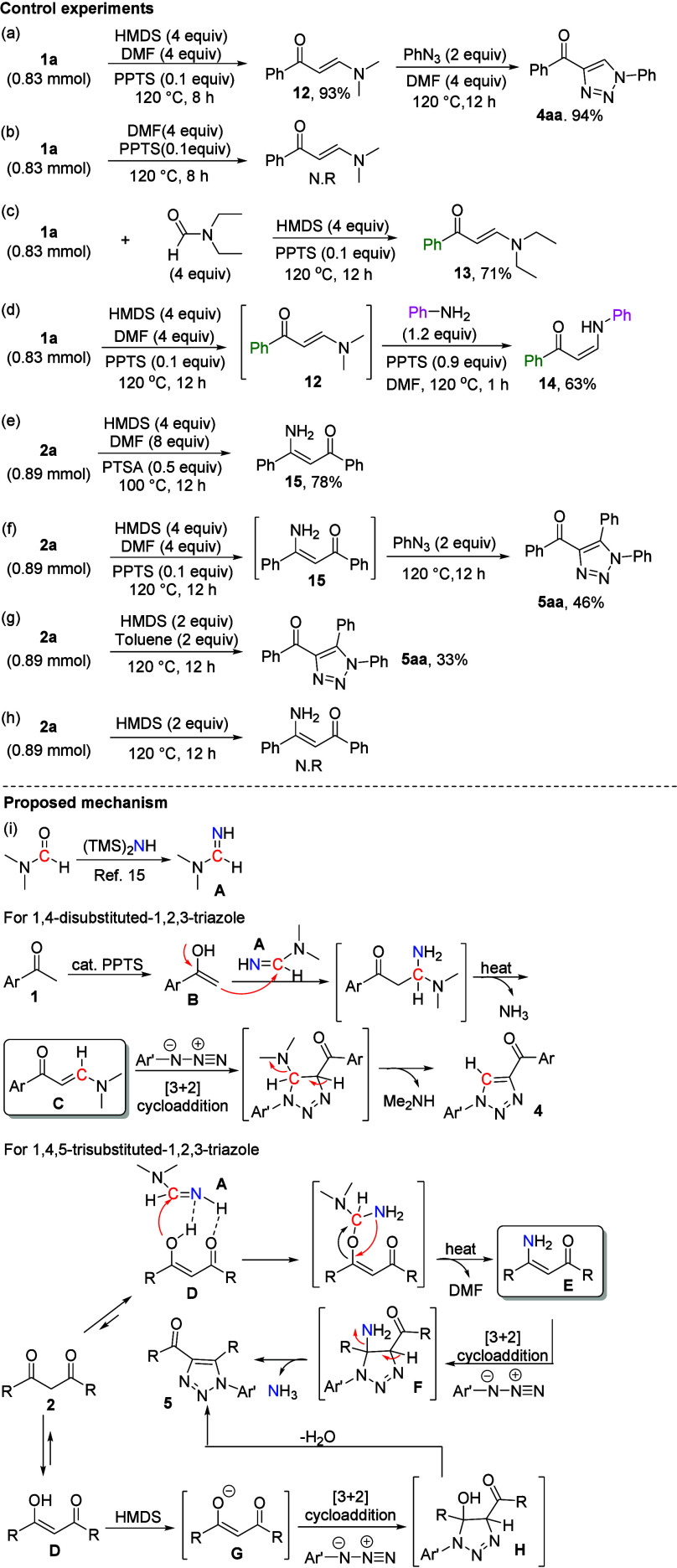
Control Experiment and Proposed Mechanism

To clarify the reaction mechanism, we conducted control experiments (Scheme 5). As expected, methyl phenyl ketone (**1a**) was converted into *N*,*N*-dimethyl enaminones (**12**) under HMDS/DMF-mediated conditions with a yield of 93%. Subsequently, *N*,*N*-dimethyl enaminone (**12**) was reacted with phenyl azide (**3a**) under the same reaction conditions, producing **4aa** in 94% yield (Scheme 5a). Moreover, we applied our developed protocol for deuteration on enaminone formation.^17^ These results indicate that a DMF agent can serve as a carbon source for the generation of 1,4-disubstituted 1,2,3-triazole. For comparison, in the absence of the HMDS agent under optimized conditions, we observed that compound **1a** was recovered without producing *N*,*N*-dimethyl enaminones (**12**) (Scheme 5b). Additionally, given the success of the reaction for the formation of enaminone, the generality of this protocol was further examined. The application of this approach to *N*,*N*-diethylformamide led to the formation of enaminone derivative **13**, with a good yield [71% (Scheme 5c)]. Moreover, to further determine the applicability scope of this method for enaminone synthesis, we conducted the reaction using a sequential one-pot process. The results revealed that this approach was efficient for the formation of *NH*-enaminone (**14**) [63% (Scheme 5d)]. Next, we observed that the β-diketone (**2a**) was successfully converted into the enaminone derivative (**15**) with a good yield [78% (Scheme 5e)]. Moreover, we observed that the β-diketone (**2a**) reacted under HMDS/DMF-mediated conditions to generate a β-aminoenone intermediate (**15**); thereafter, intermediate **15** was reacted with phenyl azide (**3a**) under optimized conditions, producing **5aa** in 46% yield (Scheme 5f). According to the literature report,^18^ 1,3-dipolar cycloaddition of β-diketones with azides in the presence of basic conditions led to the 1,2,3-triaole, in which the enolate was generated from β-diketones under basic conditions upon reaction with azides. For comparison, we performed a reaction using HMDS in the presence of toluene as a solvent. The results revealed that the desired product (**5aa**) was provided in 33% yield and confirmed that it may produce enolate from β-diketone and react with azide under basic conditions (Scheme 5g). Additionally, we observed that in the absence of the DMF agent under optimized conditions, compound **2a** was recovered without producing β-aminoenones (**15**) (Scheme 5h).

On the basis of the results of the control experiment and the literature we reviewed,^9c,15,18^ we suggest plausible mechanisms for the synthesis of 1,4-disubstituted and 1,4,5-trisubstituted 1,2,3-triazoles (Scheme 5i). One report suggested that a DMF agent can initially be reacted with HMDS under heating conditions to generate *N*,*N*-dimethylformimidamide intermediate **A**,^15^ which can then be used as a carbon/nitrogen source for the formation of enaminones. For the synthesis of 1,4-disubstituted 1,2,3-triazole, *N*,*N*-dimethylformimidamide intermediate **A** subsequently reacts with tautomer **B** under catalytic amounts of PPTS and heating conditions, resulting in the formation of enaminone intermediate **C** after the loss of a molecule of ammonia. Enaminone intermediate **C** reacts with an azide through inverse-electron-demand [3+2] cycloaddition with complete regioselectivity, producing 1,4-disubstituted 1,2,3-triazole **4** (Scheme 5i).^9c^ Next, according to our previous study, we verified that transamidination of a sulfonyl amide with *N*,*N*-dimethylformimidamide intermediate **A** occurs through hydrogen bonding.^15^ On the basis of these results, we suggest that the reaction for 1,4,5-trisubstituted 1,2,3-triazole synthesis is initiated through an intermolecular two-point hydrogen bonding system between *N*,*N*-dimethylformimidamide intermediate **A** and tautomer **D** to generate β-aminoenone intermediate **E**. Subsequently, the formation of 1,4,5-trisubstituted 1,2,3-triazole **5** from β-aminoenone intermediate **E** and aryl azide follows a mechanism similar to that underlying the formation of 1,4-disubstituted 1,2,3-triazoles (Scheme 5i). There is an alternative route in which the aryl azide undergoes 1,3-dipolar cycloaddition with the enolate intermediate (**G**) arising from the tautomerization of β-diketone under basic conditions, which produces the cycloaddition adduct (**H**). Finally, the dehydration process gives rise to 1,4,5-trisubstituted 1,2,3-triazoles (Scheme 5i).

## Conclusions

In conclusion, by employing readily available aryl methyl ketones and β-diketones as starting materials, we synthesized 1,4-disubstituted 4-acyl-1,2,3-triazoles and 1,4,5-trisubstituted 4-acyl-1,2,3-triazoles without using metal catalysts or oxidants. In the involved reactions, a DMF agent reacted with HMDS to generate an *N*,*N*-dimethylformimidamide intermediate in situ, which was then utilized as a carbon/nitrogen source for the formation of enaminones. Thereafter, a reaction with organic azides was induced to produce 4-acyl-1,2,3-triazoles. Additionally, the proposed method was successfully employed in the gram-scale synthesis of the desired products and effectively synthesized the enaminone derivatives and an analogue of a crucial pharmaceutic compound. Moreover, the conditions for this synthetic protocol are environmentally friendly (free of metal, free of oxidants, and nearly free of solvents), and the protocol can be applied for the formation of diverse functionalized 4-acyl-1,2,3-triazoles. Thus, the HMDS-mediated enaminone formation protocol described in this study is suitable for a wide range of applications in synthesizing diverse cycloaddition products.

## Data Availability

The data underlying this study are available in the published article and its Supporting Information.

## Supporting Information Available

Descriptions of experimental procedures for compounds and analytical characterization (PDF)
