# Supplementary material for: In Situ-Generated Formamidine as a Carbon/Nitrogen Source for Enaminone Formation: One-Pot Synthesis of Functionalized 4-Acyl-1,2,3-triazoles
Source: J Org Chem. 2024 Aug 19;89(17):12170–5. doi: 10.1021/acs.joc.4c01054 (PMC11382154; doi:10.1021/acs.joc.4c01054)
Supplement: Supplementary file 1 — jo4c01054_si_001.pdf [file jo4c01054_si_001.pdf]

Supporting Information

In Situ Generated Formamidine as a Carbon/Nitrogen Source for Enaminone Formation: One-Pot Synthesis of Functionalized 4-Acyl-1,2,3-Triazoles

Jia-Xin Lin,[‡a] You-Xin Chen,[‡a] Min-Chen Chien,[a] Hsiang-Jou Chen,[a] Chian-Hui Lai,[b] Chien-Fu Liang*[a]

[a] Department of Chemistry, National Chung Hsing University, Taichung 402, Taiwan.

[b] Graduate Institute of Biomedical Engineering, National Chung Hsing University, Taichung 402, Taiwan

E-mail: lcf0201@dragon.nchu.edu.tw

Table of contents

1. General Information……………………………………………………………S2

1. General Procedure…………………………………………………………….....S2

2. Characterization Data……………………………………………………………...S3

3. References…………………………………………………………………………..S39

4. Copies of ^{1}H and ^{13}C NMR Spectra of products…………………………………S42

General Information

All reactions were performed under nitrogen atmosphere. ^1H NMR and ^{13}C NMR spectra were reported on Varian 400 MHz and Jeol 400 MHz NMR spectrometer with $CDCl_3$ and $(CD_2)_3SO$ as the solvent. Chemical shifts were reported in parts per million (ppm) relative to residual solvent peak ($CDCl_3$ δ H = 7.26 ppm, δ C = 77.16 ppm; $(CD_3)_2SO$ δ H = 2.49 ppm, δ C = 39.15 ppm;). The IR spectra were measured on a Thermo Scientific Nicolet ATR-Diamond. TLC was performed on pre-coated glass plates of Silica Gel 60 F254 (0.25 mm, E. Merck); detection was performed by spraying with using a UV light. Flash column chromatography was carried out on Silica Gel 60 (230–400 mesh, E. Merck). High resolution mass spectrometry data were recorded on ESI spectrometer and EI spectrometer. Melting points were measured by Electrothermo (UK) "Mel-Temp 1101D" type melting point apparatus and are uncorrected. The following abbreviations were used to indicate multiplicities: s = singlet, d = doublet, t = triplet, q = quartet, quin. = quintet, sext = sextet, dd = doublet of doublets, dt = doublet of triplets, td = triplet of doublets, ddd = doublet of doublet of doublets, brs = broad single, m = multiplet. Yields of products refer to chromatographically purified products unless otherwise stated. All the acetophenones (**1a-1r**), 1,3-dicarbonyl compounds (**2a**, **2e-2f**), and hexamethyldisilazane were purchase from Sigma Aldrich, Alfa Aesar, TCI, or Acros Organics, and these reagents were used as received unless otherwise stated. All organic azides (**3a**–**3v**, and **14**) were known compounds and synthesized by our previous reported methods.[1]

Safety Statement. Caution! Caution should be exercised when using azides. Organic azides can be heat- and shock-sensitive and can explosively decompose with little input of external energy.

General procedure (I): One-pot synthesis of 1,4-disubstituted triazoles (4aa-4ao, 4aq, 4ar, 4at, 4ba-4sa)

HMDS (0.70 mL, 3.32 mmol, 4.0 equiv) DMF (0.26 mL, 3.32 mmol, 4.0 equiv) and azide **3a** (198 mg, 1.66 mmol, 2.0 equiv) was added to acetophenone **1a** (100 mg, 0.83 mmol, 1 equiv) and then stirred at 120 °C (oil bath) under nitrogen atmosphere for 12 hours. After the reaction completed, the crude products were purified by flash column chromatography to afford the desired product **4aa** (190.2 mg, 92% yield) as a white solid. The products of **4ab–4ao, 4aq, 4ar, 4at, 4ba–4sa** were synthesized by following general procedure I.

General procedure (II): One-pot two-step synthesis of 1.4-disubstituted triazoles (4ap, 4as)

HMDS (4.0 equiv) DMF (4.0 equiv) was added to acetophenone **1a** (0.5 mmol for

3p/0.83 mmol for **3s**) and then stirred at 120 °C (oil bath) under nitrogen atmosphere; after reaction for 8 h, azide **3p/3s** (2.0 equiv) was added to the reaction mixture for another 12 hours. After the reaction completed, the crude products were purified by flash column chromatography to afford the desired products **4ap** (120.6 mg, 82% yield) as a pale yellow solid/**4as** (153.0 mg, 66% yield) as a white solid, respectively.

General procedure (III): **The synthesis of 1,3-dicarbonyl compounds (2b-2d, 2g)**[2]
To a dried round flask was add NaH (200 mg, 5.0 mmol, 60% in mineral oil, 2.5 equiv) and dry THF (10.5 mL), the corresponding methyl 4-chlorobenzoate (375.5 mg, 2.2 mmol, 1.1 equiv) and 1-(4-chlorophenyl)ethan-1-one (0.26 mL, 2 mmol, 1.0 equiv) was subsequently added. The suspension was stirred and heat to reflux overnight. After cool to room temperature, the reaction mixture was quenched with ethyl acetate and 10% hydrochloric acid. The organic and aqueous layers were separated, and the aqueous layer was extracted with EtOAc (3 x 15 mL). The combined organic extracts were dried over Na_2SO_4. The organic layer was concentrated under reduced pressure and purified by column chromatography (EtOAc/ PE, 1:50) to furnish the desired product **2b** (229.1 mg, 40% yield) as a white solid. The products of **2c-2d** and **2g** were synthesized by following general procedure III.

General procedure (IV): **One-pot synthesis of 1,4,5-trisubstituted triazoles** (**5aa-5aq, 5au-5av, 5ba-5da, 5be-5de, 5bk-5gk**)
HMDS (0.375 mL, 1.784 mmol, 2.0 equiv) DMF (0.138 mL, 1.784 mmol. 2.0 equiv) and azide **3a** (212.5 mg, 1.784 mmol, 2.0 equiv) was added to 1,3-diketones **2a** (200 mg, 0.89 mmol, 1 equiv) and then stirred at 120 °C (oil bath) under nitrogen atmosphere for 12 hours. After the reaction completed, the crude products were purified by flash column chromatography to afford the desired product **5aa** (265.2 mg, 91% yield) as a white solid. The products of **5aa-5aq, 5au-5av, 5ba-5da, 5be-5de,** and **5bk-5gk** were synthesized by following general procedure IV.

Phenyl(1-phenyl-1H-1,2,3-triazol-4-yl)methanone (4aa)[3]

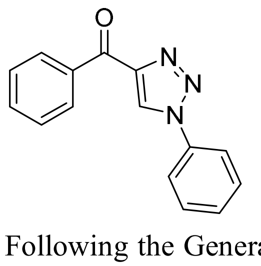

Following the General procedure (I), using acetophenone **1a** (100.0 mg, 0.83 mmol), phenyl azide **3a** (197.7 mg, 1.66 mmol), hexamethyldisilazane (0.70 mL, 3.33 mmol) and N,N-dimethylformamide (0.26 mL, 3.33 mmol), 4-methylbenzenesulfonate (20.9

mg, 0.08 mmol). The crude product was then purified by column chromatography (DCM) to afford **4aa** (190.2 mg, 92% yield) as a white solid; ^{1}H NMR (400 MHz, $CDCl_3$) δ 8.71 (s, 1H), 8.49 (d, *J* = 7.1 Hz, 2H), 7.82 (d, *J* = 8.1 Hz, 2H), 2H), 7.65 (t, *J* = 7.4 Hz, 1H), 7.67 - 7.50 (m, 5H); $^{13}C\{^1H\}$ NMR (100 MHz, $CDCl_3$) δ 185.4, 148.4, 136.3, 136.2, 133.3, 130.5, 129.8, 129.3, 128.3, 126.4, 120.6.

Gram scale synthetic method of product **4aa**: Following the general procedure (I), using acetophenone **1a** (1000 mg, 8.3 mmol), phenyl azide **3a** (1977 mg, 16.6 mmol), hexamethyldisilazane (7.0 mL, 33.3 mmol) and N,N-dimethylformamide (2.6 mL, 33.3 mmol), 4-methylbenzenesulfonate (209 mg, 0.8 mmol). The crude product was then purified by column chromatography (DCM) to afford **4aa** (1613.5 mg, 78% yield) as a white solid.

1-(4-Methylphenoyl)-4phenoyl-1,2,3-triazole (**4ab**)[3]

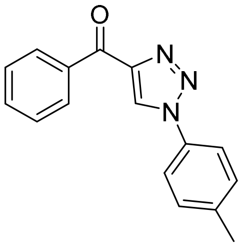

Following the General procedure (I), using acetophenone **1a** (100.0 mg, 0.83 mmol), 1-azido-4-methylbenzene **3b** (221.0 mg, 1.66 mmol), hexamethyldisilazane (0.70 mL, 3.33 mmol) and N,N-dimethylformamide (0.26 mL, 3.33 mmol), 4-methylbenzenesulfonate (20.9 mg, 0.08 mmol). The crude product was then purified by column chromatography (DCM) to afford **4ab** (179.8 mg, 92% yield) as a white solid; ^{1}H NMR (400 MHz, $CDCl_3$) δ 8.67 (s, 1H), 7.67 (d, *J* = 8.3 Hz, 1H), 7.62 (t, *J* = 7.3 Hz, 2H), 8.47(d, *J* = 8.1 Hz, 2H), 7.53 (t, *J* = 7.8 Hz, 2H), 7.34 (d, *J* = 8.2 Hz, 2H), 2.42 (s, 3H); $^{13}C\{^1H\}$ NMR (100 MHz, $CDCl_3$) δ 185.6, 148.5, 139.8, 136.5, 134.1, 133.4, 130.7, 130.5, 128.5, 126.4, 120.7, 21.2.

1-(3-Methylphenoyl)-4phenoyl-1,2,3-triazole (4ac)

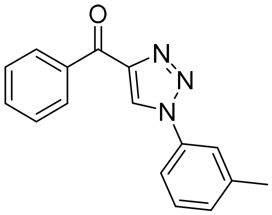

Following the General procedure (I), using acetophenone **1a** (100.0 mg, 0.83 mmol), 1-azido-3-methylbenzene **3c** (221.0 mg, 1.66 mmol), hexamethyldisilazane (0.70 mL, 3.33 mmol) and N,N-dimethylformamide (0.26 mL, 3.33 mmol), 4-

methylbenzenesulfonate (20.9 mg, 0.08 mmol). The crude product was then purified by column chromatography (DCM) to afford **4ac** (188.2 mg, 86% yield) as a white solid; m.p. 138-139 °C; ATR-IR (cm^{-1}): 1271, 1531, 1653, 3060, 3121**;** ^{1}**H** NMR (400 MHz, $CDCl_3$) δ 8.68 (s, 1H), 8.49(d, *J* = 7.1 Hz, 2H), 7.68 - 7.62 (m, 2H), 7.61 - 7.52 (m, 3H), 7.45 (t, *J* = 7.8 Hz, 2H), 7.32 (d, *J* = 7.6 Hz, 2H), 2.49 (s, 3H); $^{13}C\{^{1}H\}$ NMR (100 MHz, $CDCl_3$): δ 185.4, 148.3, 140.1, 136.4, 136.2, 133.3, 130.6, 130.1, 129.6, 128.4, 126.4, 121.3, 117.7, 21.3. HRMS (EI) m/z: $[M]^+$ calcd for $C_{16}H_{13}N_3O$ 263.1059; found 263.1069

1-(2-Methylphenoyl)-4phenoyl-1,2,3-triazole (4ad)[3]

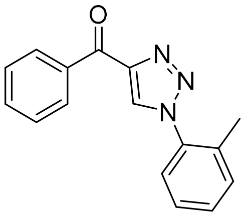

Following the General procedure (I), using acetophenone **1a** (100.0 mg, 0.83 mmol), 1-azido-2-methylbenzene **3d** (221.0 mg, 1.66 mmol), hexamethyldisilazane (0.70 mL, 3.33 mmol) and N,N-dimethylformamide (0.26 mL, 3.33 mmol), 4-methylbenzenesulfonate (20.9 mg, 0.08 mmol). The crude product was then purified by column chromatography (DCM) to afford **4ad** (125.0 mg, 64% yield) as a white solid; ^{1}H NMR (400 MHz, $CDCl_3$) δ 8.50 (d, *J* = 7.1 Hz, 2H), 8.46 (s, 1H), 7.63 (t, *J* = 7.4 Hz, 1H), 7.54 (t, *J* = 7.7 Hz, 2H), 7.48 - 7.44 (m, 1H), 7.41 - 7.34 (m, 3H), 2.27 (s, 3H); $^{13}C\{^{1}H\}$ NMR (100 MHz, $CDCl_3$) δ 185.7, 147.9, 136.6, 135.8, 133.6, 133.4, 131.7, 130.7, 130.5, 129.9, 128.5, 127.1,125.9, 18.0.

(1-(4-Ethylphenyl)-1H-1,2,3-triazol-4-yl)(phenyl)methanone (4ae)

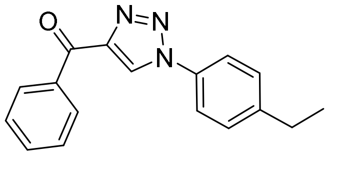

Following the General procedure (I), using acetophenone **1a** (100.0 mg, 0.83 mmol), 1-azido-4-ethylbenzene **3e** (244.3 mg, 1.66 mmol), hexamethyldisilazane (0.70 mL, 3.33 mmol) and N,N-dimethylformamide (0.26 mL, 3.33 mmol), 4-methylbenzenesulfonate (20.9 mg, 0.08 mmol). The crude product was then purified by column chromatography (DCM) to afford **4ae** (205.4 mg, 85% yield) as a white solid; m.p. 128 - 130 °C; ATR-IR (cm^{-1}): 1267, 1521, 1640, 2957, 3130; ^{1}H NMR (400 MHz, $CDCl_3$) δ 8.70 (s, 1H), 8.47 (d, *J* = 7.8 Hz, 2H), 7.69 (d, *J* = 7.1 Hz, 2H), 7.60 (t, *J* = 7.3 Hz, 1H), 7.51 (t, *J* = 7.7 Hz, 2H), 7.34 (d, *J* = 8.1 Hz, 2H), 2.70 (q, *J* = 7.6 Hz, 2H),

1.25 (t, J = 7.6 Hz, 3H); $^{13}C\{^1H\}$ NMR (100 MHz, $CDCl_3$) δ 185.5, 148.4, 1465.9, 136.5, 134.2, 133.3, 130.6, 129.2, 128.4, 126.4, 120.7, 28.5, 15.4. HRMS (EI) m/z: $[M]^+$ calcd for $C_{17}H_{15}N_3O$ 277.1215; found 277.1220.

(1-(4-(Tert-butyl)phenyl)-1H-1,2,3-triazol-4-yl)(phenyl)methanone (4af)

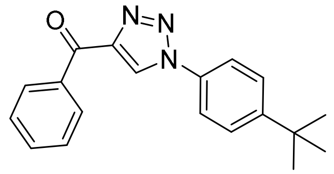

Following the General procedure (I), using acetophenone **1a** (80.0 mg, 0.67 mmol), 1-azido-4-(*tert*-butyl) benzene **3f** (233.2 mg, 1.33 mmol), hexamethyldisilazane (0.56 mL, 2.66 mmol) and N,N-dimethylformamide (0.21 mL, 2.66 mmol), 4-methylbenzenesulfonate (16.8 mg, 0.07 mmol). The crude product was then purified by column chromatography (DCM) to afford **4af** (179.6 mg, 88% yield) as a white solid; m.p 160 - 162 °C; ATR-IR (cm^{-1}): 1266, 1522, 1641, 2957, 3067, 3128; ^1H NMR (400 MHz, $CDCl_3$) δ 8.74 (s, 1H), 8.47 (d, J = 7.1 Hz, 2H), 7.72 (d, J = 8.8 Hz, 2H), 7.59 (t, J = 7.4 Hz, 1H), 7.56 - 7.45 (m, 4H), 1.33 (s, 9H); $^{13}C\{^1H\}$ NMR (100 MHz, $CDCl_3$) δ 185.4, 152.8, 148.3, 136.4, 133.9, 133.3, 130.6, 128.3, 126.7, 126.4, 120.4, 34.8, 31.2. HRMS (EI) m/z: $[M]^+$ calcd for $C_{19}H_{19}N_3O$ 305.1528; found 305.1535.

(1-(4-Octylphenyl)-1H-1,2,3-triazol-4-yl)(phenyl)methanone (4ag)

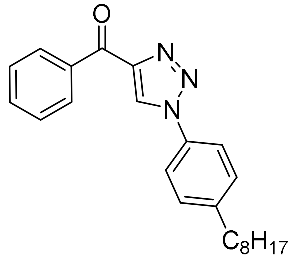

Following the General procedure (I), using acetophenone **1a** (100.0 mg, 0.83 mmol), 1-azido-4-octylbenzene **3g** (384.0 mg, 1.66 mmol), hexamethyldisilazane (0.70 mL, 3.33 mmol) and N,N-dimethylformamide (0.26 mL, 3.33 mmol), 4-methylbenzenesulfonate (20.9 mg, 0.08 mmol). The crude product was then purified by column chromatography (DCM) to afford **4ag** (238.2 mg, 80% yield) as a white solid; m.p. 97 - 99 °C; ATR-IR (cm^{-1}): 1255, 1267, 1525, 1632, ,2854, 2921, 3134; ^1H NMR (400 MHz, $CDCl_3$) δ 8.70 (s, 1H), 8.47 (d, J = 7.0 Hz, 2H), 7.69 (d, J = 8.4 Hz, 2H), 7.61 (t, J = 7.4 Hz, 1H), 7.51 (t, J = 7.8 Hz, 2H), 7.33 (d, J = 8.4 Hz, 2H), 2.66 (t, J = 7.6 Hz, 2H), 1.70 -1.55 (m, 2H), 1.31 - 1.26 (m, 11H), 0.87 (t, J = 6.7 Hz, 3H); $^{13}C\{^1H\}$ NMR (100 MHz, $CDCl_3$) δ 185.6, 148.4, 144.8, 136.5, 134.2, 133.4, 130.7, 129.8, 128.4, 126.4, 120.7, 35.6, 31.9, 31.3, 29.4, 29.3, 29.2, 22.7, 14.1. HRMS (EI) m/z: $[M]^+$ calcd

for $C_{23}H_{27}N_3O$ 361.2154; found 361.2151.

(1-(3,5-Dimethylphenyl)-1H-1,2,3-triazol-4-yl)(phenyl)methanone (4ah)

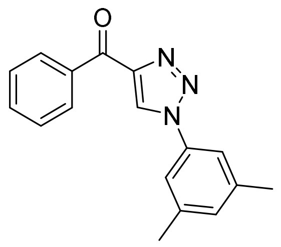

Following the General procedure (I), using acetophenone **1a** (100.0 mg, 0.83 mmol), 1-azido-3,5-dimethylbenzene **3h** (244.3 mg, 1.66 mmol), hexamethyldisilazane (0.70 mL, 3.33 mmol) and N,N-dimethylformamide (0.26 mL, 3.33 mmol), 4-methylbenzenesulfonate (20.9 mg, 0.08 mmol). The crude product was then purified by column chromatography (DCM) to afford **4ah** (230.1 mg, 70% yield) as a white solid; m.p. 107 - 109 °C; ATR-IR (cm^{-1}): 1240, 1294, 1531, 1653, 3127; ^1H NMR (400 MHz, $(CD_3)_2SO$) δ 9.52 (s, 1H), 8.25 (d, *J* = 7.4 Hz, 2H), 7.72 (t, *J* = 7.3 Hz, 1H), 7.67 (s, 1H) 7.61 (t, *J* = 7.7 Hz, 2H), 7.19 (s, 1H), 2.38 (s, 6H); $^{13}C\{^1H\}$ NMR (100 MHz, $CDCl_3$) δ 185.4, 148.2, 139.8, 136.4, 136.1, 133.3, 131.0, 130.5, 128.3, 126.4, 118.4, 21.2. HRMS (EI) m/z: $[M]^+$ calcd for $C_{17}H_{15}N_3O$ 277.1215; found 277.1212.

1-(4-Methoxyphenyl)-4-phenoyl-1,2,3-triazole (4ai)[3]

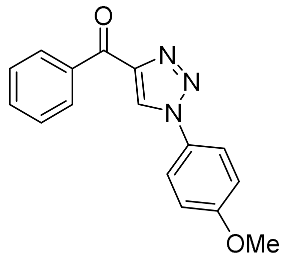

Following the General procedure (I), using acetophenone **1a** (100.0 mg, 0.83 mmol), 1-azido-4-methoxybenzene **3i** (247.6 mg, 1.66 mmol), hexamethyldisilazane (0.70 mL, 3.33 mmol) and N,N-dimethylformamide (0.26 mL, 3.33 mmol), 4-methylbenzenesulfonate (20.9 mg, 0.08 mmol). The crude product was then purified by column chromatography (DCM) to afford **4ai** (165.4 mg, 89% yield) as a white solid; ^1H NMR (400 MHz, $CDCl_3$) δ 8.61 (s, 1H), 8.49 (d, *J* = 7.2 Hz, 2H), 7.71 (d, *J* = 9.0 Hz, 2H), 7.64 (t, *J* = 7.4 Hz, 1H), 7.55 (t, *J* = 7.9 Hz, 2H), 7.07(d, *J* = 9.0 Hz, 2H), 3.89 (s, 3H); $^{13}C\{^1H\}$ NMR (100 MHz, $CDCl_3$) δ 185.7, 160.4, 148.4, 136.6, 133.4, 130.7, 129.8, 128.5, 126.5, 122.4, 115.0, 55.7.

(1-(4-Fluorophenyl)-1H-1,2,3-triazol-4-yl)(phenyl)methanone (4aj)[4]

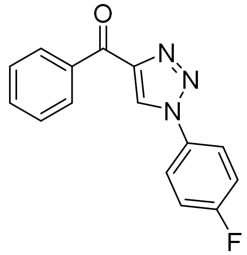

Following the General procedure (I), using acetophenone **1a** (80.0 mg, 0.67 mmol), 1-azido-4-fluorobenzene **3j** (183.7 mg, 1.33 mmol), hexamethyldisilazane (0.558 mL, 2.66 mmol) and N,N-dimethylformamide (0.21 mL, 2.66 mmol), 4-methylbenzenesulfonate (16.8 mg, 0.07 mmol). The crude product was then purified by column chromatography (DCM) to afford **4aj** (115.1 mg, 64% yield) as a white solid; ^{1}H NMR (400 MHz, $CDCl_3$) δ 8.66 (s, 1H), 8.49 (d, J = 7.4 Hz, 2H), 7.84 - 7.76 (m, 2H), 7.65 (t, J = 7.3 Hz, 1H), 7.56 (t, J = 7.7 Hz, 2H), 7.28 (t, J = 8.4 Hz, 2H); ^{13}C{^{1}H} NMR (100 MHz, $CDCl_3$) δ 185.6, 163.1 (d, J = 250.5 Hz), 148.8, 136.4, 133.6, 132.7 (d, J = 3.0 Hz), 130.6 (2C), 128.5 (2C), 126.5, 122.8 (d, J = 9.0 Hz, 2C), 117.0 (d, J = 23.0 Hz, 2C).^{19}F NMR (376 MHz, $CDCl_3$) δ -112.0.

(1-(4-Chlorophenyl)-1H-1,2,3-triazol-4-yl)(phenyl)methanone (4ak)

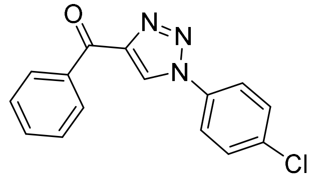

Following the General procedure (I), using acetophenone **1a** (100.0 mg, 0.83 mmol), 1-azido-4-chlorobenzene **3k** (254.9 mg, 1.66 mmol), hexamethyldisilazane (0.70 mL, 3.33 mmol) and N,N-dimethylformamide (0.26 mL, 3.33 mmol), 4-methylbenzenesulfonate (20.9 mg, 0.08 mmol). The crude product was then purified by column chromatography (DCM) to afford **4ak** (196.1 mg, 99% yield) as a white solid; m.p. 219 -221 °C; ATR-IR (cm^{-1}): 1267, 1501, 1521, 1637, 3015, 3023, 3124; ^{1}H NMR (400 MHz, $(CD_3)_2SO$) δ 9.62 (s, 1H), 8.27 (d, J = 8.0 Hz, 2H), 8.09 (d, J = 8.8 Hz, 2H), 7.73 (d, J = 8.7 Hz, 3H), 7.62 (t, J = 7.6 Hz, 2H); ^{13}C{^{1}H} NMR $(CD_3)_2SO$): 185.1, 147.2, 136.5, 134.9, 133.9 133.5, 130.0, 129.9, 128.7, 128.2, 122.5. HRMS (EI) m/z:[M]$^{+}$ calcd for 283.0512; found 283.0515.

(1-(4-Bromophenyl)-1H-1,2,3-triazol-4-yl)(phenyl)methanone (4al)

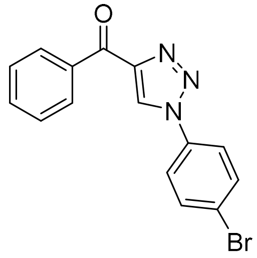

Following the General procedure (I), using acetophenone **1a** (80.0 mg, 0.67 mmol), 1-azido-4-bromobenzene **3l** (263.7 mg, 1.33 mmol), hexamethyldisilazane (0.56 mL, 2.66 mmol) and N,N-dimethylformamide (0.21 mL, 2.66 mmol), 4-methylbenzenesulfonate (16.8 mg, 0.07 mmol). The crude product was then purified by column chromatography (DCM) to afford **4al** (182.9 mg, 84% yield) as a white solid; m.p. 232- 235°C; ATR-IR (cm^{-1}): 1267, 1523, 1639, 3052, 3086, 3098, 3129; ^{1}H NMR (400 MHz, $CDCl_3$) δ 8.69 (s, 1H), 8.48 (d, J = 7.2 Hz, 2H), 7.79 - 7.70 (m, 4H), 7.65 (t, J = 7.4 Hz, 1H), 7.56 (t, J = 7.6 Hz, 2H). $^{13}C\{^{1}H\}$ NMR (100 MHz, $CDCl_3$) δ 185.5, 148.9, 136.4, 135.4, 133.7, 133.3, 130.8, 128.6, 126.3, 123.5, 122.3. HRMS (EI) m/z: $[M]^+$ calcd for 327.0007; found 326.9999.

(1-(4-Iodophenyl)-1H-1,2,3-triazol-4-yl)(phenyl)methanone (4am)

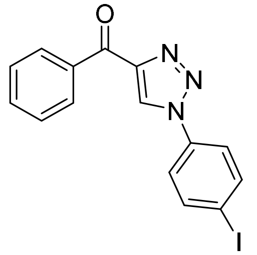

Following the General procedure (I), using acetophenone **1a** (80.0 mg, 0.67 mmol), 1-azido-4-iodobenzene **3m** (325.9 mg, 1.33 mmol), hexamethyldisilazane (0.56 mL, 2.66 mmol) and N,N-dimethylformamide (0.21 mL, 2.66 mmol), 4-methylbenzenesulfonate (16.8 mg, 0.07 mmol). The crude product was then purified by column chromatography (DCM) to afford **4am** (211.2 mg, 84% yield) as a white solid; m.p. 243 - 245 °C; ATR-IR (cm^{-1}): 1265, 1524, 1640, 3050, 3092, 3130; ^{1}H NMR (400 MHz, $(CD_3)_2SO$) δ 9.61 (s, 1H), 8.26 (d, J = 7.1 Hz, 2H), 8.01 (d, J = 8.8 Hz, 2H), 7.86 (d, J = 8.8 Hz, 2H), 7.73 (t, J = 7.4 Hz, 1H), 7.62 (t, J = 7.6 Hz, 2H); $^{13}C\{^{1}H\}$ NMR $(CD_3)_2SO$) δ 185.0, 147.2, 138.6, 136.5, 135.7, 133.5, 130.0, 128.6, 128.0, 122.6, 95.4. HRMS (EI) m/z: $[M]^+$ calcd for $C_{15}H_{10}IN_3O$ 374.9869; found 374.9858

1-(3-Chlorophenyl)-4-phenoyl-1,2,3-triazole (4an)[3]

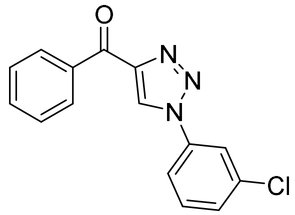

Following the General procedure (I), using acetophenone **1a** (100.0 mg, 0.83 mmol), 1-azido-3-chlorobenzene **3n** (255.0 mg, 1.66 mmol), hexamethyldisilazane (0.70 mL, 3.33 mmol) and N,N-dimethylformamide (0.26 mL, 3.33 mmol), 4-methylbenzenesulfonate (20.9 mg, 0.08 mmol). The crude product was then purified by column chromatography (DCM) to afford **4an** (197.0 mg, 99% yield) as a white solid; ^{1}H NMR (400 MHz, $CDCl_3$): δ 8.70 (s, 1H), 8.48 (d, J = 7.5 Hz, 2H), 7.88 (d, J = 1.7 Hz, 1H), 7.73 - 7.05 (m, 1H), 7.66 (t, J = 7.4 Hz, 1H), 7.57 - 7.49 (m, 4H); ^{13}C{^{1}H} NMR (100 MHz, $CDCl_3$): δ 185.4, 148.8, 137.3, 136.3, 135.9, 133.6, 131.1, 130.7, 129.7, 128.6, 126.5, 121.2, 118.8.

(1-(2-Chlorophenyl)-1H-1,2,3-triazol-4-yl)(phenyl)methanone (4ao)

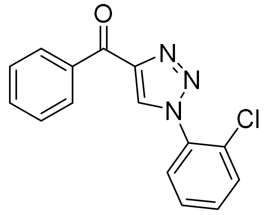

Following the General procedure (I), using acetophenone **1a** (100.0 mg, 0.83 mmol), 1-azido-2-chlorobenzene **3o** (255.0 mg, 1.66 mmol), hexamethyldisilazane (0.70 mL, 3.33 mmol) and N,N-dimethylformamide (0.26 mL, 3.33 mmol), 4-methylbenzenesulfonate (20.9 mg, 0.08 mmol). The crude product was then purified by column chromatography (DCM) to afford **4ao** (197.1 mg, 99% yield) as a white solid; m.p. 97-98 °C; ATR-IR (cm^{-1}): 1246, 1277, 1340, 1495, 1514, 1533, 1647, 1661, 3051, 3132; ^{1}H NMR (400 MHz, $CDCl_3$): δ 8.69 (s, 1H), 8.46 (d, J = 7.1 Hz, 2H), 7.68 - 7.57 (m, 3H), 7.53 (t, J = 7.5 Hz, 2H), 7.50 - 7.42 (m, 2H); ^{13}C{^{1}H} NMR (100 MHz, $CDCl_3$) δ 185.4, 147.7, 136.4, 134.1, 133.4, 131.4, 130.9, 130.5, 130.5, 128.6, 128.4, 128.1, 127.6. HRMS (EI) m/z: $[M]^+$ calcd for $C_{15}H_{10}ClN_3O$ 283.0512; found 283.0506

(1-(4-Nitrophenyl)-1H-1,2,3-triazol-4-yl)(phenyl)methanone (4ap)[5]

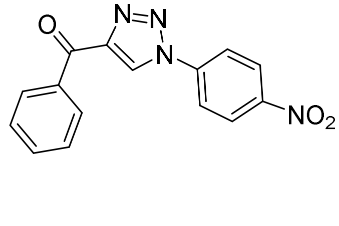

Following the General procedure (II), using acetophenone **1a** (60.0 mg, 0.5 mmol), 4-nitrophenyl azide **3p** (164.1 mg, 1.0 mmol), hexamethyldisilazane (0.42 mL, 2.0 mmol) and N,N-dimethylformamide (0.16 mL, 2.0 mmol), 4-methylbenzenesulfonate (12.5 mg, 0.05 mmol). The crude product was then purified by column chromatography (DCM) to afford **4ap** (120.6 mg, 82% yield) as a pale yellow solid; m.p. 288-290°C; ^{1}H NMR (400 MHz, $(CD_2)_3SO$) δ 9.80 (s, 1H), 8.49 (d, *J* = 9.1 Hz, 2H), 8.37 (d, *J* = 9.0 Hz, 2H), 8.26 (d, *J* = 7.8 Hz, 2H), 7.74 (t, *J* = 7.2 Hz, 1H), 7.62 (t, *J* = 7.8 Hz, 2H); ^{13}C{^{1}H} NMR (100 MHz, $(CD_2)_3SO$) δ 184.9, 147.3, 147.2, 140.4, 136.4, 133.6, 129.9, 128.7, 125.5, 121.5.

Methyl 4-(4-benzoyl-1H-1,2,3-triazol-1-yl)benzoate (4aq)

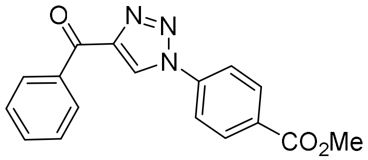

Following the General procedure (I), using acetophenone **1a** (100.0 mg, 0.83 mmol), methyl 4-azidobenzoate **3q** (294.1 mg, 1.66 mmol), hexamethyldisilazane (0.70 mL, 3.33 mmol) and N,N-dimethylformamide (0.26 mL, 3.33 mmol), 4-methylbenzenesulfonate (20.9 mg, 0.08 mmol). Reaction time is 4 hr. The crude product was then purified by column chromatography (DCM) to afford **4aq** (201.7 mg, 80% yield) as a white solid; m.p. 201-204°C; ATR-IR (cm^{-1}): 1268, 1285, 1528, 1638, 1715, 3132; ^{1}H NMR (400 MHz, $CDCl_3$): δ 8.79 (s, 1H), 8.48 (d, *J* = 7.5 Hz, 2H), 8.26 (d, *J* = 8.6 Hz, 2H), 7.93 (d, *J* = 8.5 Hz, 2H), 7.65 (t, *J* = 7.3 Hz, 1H), 7.55 (t, *J* = 7.6 Hz, 2H), 3.97 (s, 3H); ^{13}C{^{1}H} NMR (100 MHz, $CDCl_3$): δ 185.4, 165.8, 148.9, 139.5, 136.4, 133.7, 131.6, 131.1, 128.6, 126.4, 120.4, 52.7. HRMS (EI) m/z: $[M]^+$ calcd for $C_{17}H_{13}N_3O_3$ 307.0957; found 307.0948

(1-(Naphthalen-1-yl)-1H-1,2,3-triazol-4-yl)(phenyl)methanone (4ar)[4]

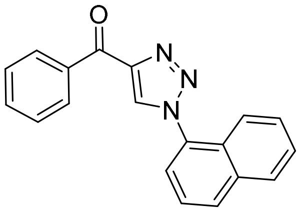

Following the General procedure (I), using acetophenone **1a** (63.1 mg, 0.53 mmol), 1-azidonaphthalene **3r** (326.7 mg, 1.1 mmol), hexamethyldisilazane (0.44 mL, 2.1 mmol) and N,N-dimethylformamide (0.16 mL, 2.1 mmol), 4-methylbenzenesulfonate (13.1 mg, 0.05 mmol). The crude product was then purified by column chromatography (DCM) to afford **4ar** (84.0 mg, 53% yield) as a white solid; ^{1}H NMR (400 MHz, $CDCl_3$):

δ 8.66 (s, 1H), 8.56 (d, *J* = 7.1 Hz, 2H), 8.09 (dd, *J* = 7.6, 1.8 Hz, 1 H), 8.02 - 7.99 (m, 2H), 7.71 - 7.54 (m, 8H); $^{13}C\{^1H\}$ NMR (100 MHz, $CDCl_3$): δ 185.7, 148.1, 136.6, 134.3, 133.5, 133.1,131.0,130.8 (2C), 128.6 (2C), 128.5, 128.3, 127.4, 125.1, 123.8, 122.1.

(1-Benzyl-1H-1,2,3-triazol-4-yl)(phenyl)methanone (4as)[6]

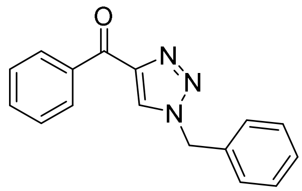

Following the General procedure (II), using acetophenone **1a** (100.0 mg, 0.83 mmol), (azidomethyl)benzene **3s** (332.2 mg, 2.5 mmol), hexamethyldisilazane (0.70 mL, 3.33 mmol) and N,N-dimethylformamide (0.26 mL, 3.33 mmol), 4-methylbenzenesulfonate (20.9 mg, 0.08 mmol). The crude product was then purified by column chromatography (DCM) to afford **4as** (153.0 mg, 66% yield) as a white solid; ^1H NMR (400 MHz, CDCl3) δ 8.42 (d, *J* = 7.1 Hz, 2H), 8.16 (s, 1H), 7.61 (t, *J* = 7.3 Hz, 1 H), 7.51 (t, J = 7.8 Hz, 2H), 7.42 - 7.39 (m, 3H), 7.35 - 7.30 (m, 2H), 5.61 (s, 2H); $^{13}C\{^1H\}$ NMR (100 MHz, $CDCl_3$) δ 185.7, 148.4, 136.5, 133.7, 133.3, 130.6, 129.4, 129.2, 128.5, 128.4, 54.4.

(1-(2,6-Dichlorophenyl)-1H-1,2,3-triazol-4-yl)(phenyl)methanone (4at)

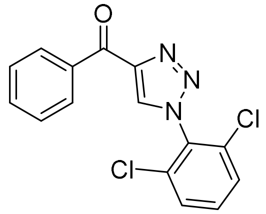

Following the General procedure (I), using acetophenone **1a** (100.0 mg, 0.83 mmol), 2-azido-1,3-dichlorobenzene **3t** (300.8 mg, 1.66 mmol), hexamethyldisilazane (0.70 mL, 3.33 mmol) and N,N-dimethylformamide (0.26 mL, 3.33 mmol), 4-methylbenzenesulfonate (20.9 mg, 0.08 mmol). The crude product was then purified by column chromatography (DCM) to afford **4at** (200.0 mg, 76% yield) as an off-white solid; m.p. 123 – 125 °C; ATR-IR (cm^{-1}): 1244, 1263, 1442, 1519, 1657, 3134; ^1H NMR (400 MHz, $CDCl_3$) ***δ*** 8.52 (d, *J* = 7.2 Hz, 2H), 8.46 (s, 1H), 7.65 (t, *J* = 7.3 Hz, 1H), 7.58 - 7.54 (m, 4H), 7.52 - 7.48 (m, 2H); $^{13}C\{^1H\}$ NMR (100 MHz, $CDCl_3$) ***δ*** 185.4, 148.0, 136.5, 133.8, 133.6, 132.7, 132.3, 131.1, 130.7, 129.1, 128.6. HRMS (EI) m/z: $[M]^+$ calcd for $C_{15}H_9Cl_2N_3O$ 317.0123; found 317.0131

(4-Chlorophenyl)(1-phenyl-1H-1,2,3-triazol-4-yl)methanone (4ba)[4]

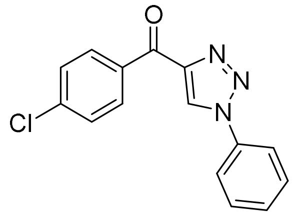

Following the General procedure (I), using 4'-Chloroacetophenone **1b** (100.0 mg, 0.65 mmol), phenyl azide **3a** (154 mg, 1.29 mmol), hexamethyldisilazane (0.54 mL, 2.59 mmol) and N,N-dimethylformamide (0.2 mL, 2.59 mmol), 4-methylbenzenesulfonate (16.1 mg, 0.06 mmol). The product was purified by flash column chromatography (DCM) afforded **4ba** (159.4 mg, 87% yield) as an off-white solid; ^{1}H NMR (400 MHz, $CDCl_3$): δ 8.71 (s, 1H), 8.51 (d, J = 8.6 Hz, 2H), 7.81 (d, J = 7.7 Hz, 2H), 7.59 (t, J = 7.6 Hz, 2H), 7.53 (d, J = 8.7 Hz, 3H); ^{13}C{^{1}H} NMR (100 MHz, $CDCl_3$): δ 184.2, 148.5, 140.1, 136.4, 134.7, 132.2, 130.1, 129.7, 128.9, 126.6, 120.9.

(3-Chlorophenyl)(1-phenyl-1H-1,2,3-triazol-4-yl)methanone (4ca)[5]

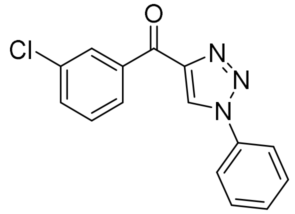

Following the General procedure (I), using 3-chloroacetophenone **1c** (100.0 mg, 0.65 mmol), phenyl azide **3a** (154.1 mg, 1.29 mmol), hexamethyldisilazane (0.543 mL, 2.59 mmol) and N,N-dimethylformamide (0.2 mL, 2.59 mmol), 4-methylbenzenesulfonate (16.3 mg, 0.06 mmol). The product was purified by flash column chromatography (DCM) afforded **4ca** (130.5 mg, 71% yield) as an off-white solid; ^{1}H NMR (400 MHz, $CDCl_3$) δ 8.72 (s, 1H), 8.48 - 8.44 (m, 2H), 7.82 - 7.80 (m, 2H), 7.62 - 7.48 (m, 5H);^{13}C{^{1}H} NMR (100 MHz, $CDCl_3$) δ 184.1, 148.3, 137.9, 136.3, 134.8, 133.4, 130.6, 130.1, 129.8, 129.7, 129.0, 126.6, 120.9.

(2-Chlorophenyl)(1-phenyl-1H-1,2,3-triazol-4-yl)methanone (4da)

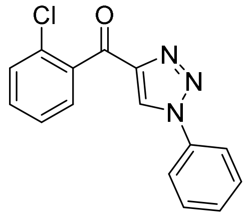

Following the General procedure (I), using 2-chloroacetophenone **1d** (100.0 mg, 0.65 mmol), phenyl azide **3a** (154.1 mg, 1.29 mmol), hexamethyldisilazane (0.543 mL, 2.59

mmol) and N,N-dimethylformamide (0.2 mL, 2.59 mmol), 4-methylbenzenesulfonate (16.3 mg, 0.06 mmol). The product was purified by flash column chromatography (DCM) afforded **4da** (133.6 mg, 73% yield) as an off-white solid; m.p. 124-129 °C; ATR-IR (cm^{-1}): 1241, 1521, 1667, 3057, 3127; ^1H NMR (400 MHz, $CDCl_3$) δ 8.64 (s, 1 H), 7.79 (d, *J* = 7.7 Hz, 2 H), 7.69 (dd, *J* = 7.5 ,1.6 Hz, 1 H), 7.58 (t, *J* = 7.6 Hz, 2 H), 7.54 - 7.45 (m, 3 H), 7.42 (td, *J* = 7.3 ,1.7 Hz, 1 H); $^{13}C\{^1H\}$ NMR (100 MHz, $CDCl_3$) δ 186.8, 147.8, 137.2, 136.2, 132.0, 131.8, 130.4, 130.2, 129.9, 129.5, 126.6, 125.7, 120.7. HRMS (EI) m/z: $[M]^+$ calcd for $C_{15}H_{10}ClN_3O$ 283.0512; found 283.0504

(4-Fluorophenyl)(1-phenyl-1H-1,2,3-triazol-4-yl)methanone (4ea)[4]

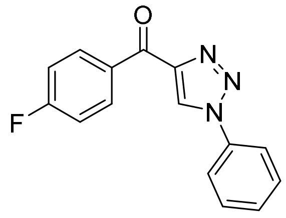

Following the General procedure (I), using 4-fluoroacetophenone **1e** (80.0 mg, 0.58 mmol), phenyl azide **3a** (138.2 mg, 1.16 mmol), hexamethyldisilazane (0.482 mL, 2.32 mmol) and N,N-dimethylformamide (0.18 mL, 2.32 mmol), 4-methylbenzenesulfonate (15.1 mg, 0.06 mmol). The product was purified by flash column chromatography (DCM) afforded **4ea** (108.0 mg, 70% yield) as an off-white solid; ^1H NMR (400 MHz, $CDCl_3$): δ 8.71 (s, 1H), 8.63 – 8.59 (m, 2H), 7.82 – 7.80 (m, 2H), 7.60 (t, *J* = 7.5 Hz, 2H), 7.53 (t, *J* = 7.3 Hz, 1H), 7.23 (t, *J* = 8.6 Hz, 2H); $^{13}C\{^1H\}$ NMR (100 MHz, $CDCl_3$): δ 183.8, 166.1 (d, *J* = 255.9 Hz), 148.6, 136.4, 133.6 (d, *J* = 9.0 Hz,2C), 132.7 (d, *J* = 6.9 Hz), 130.1 (2C), 129.6, 126.6, 120.8 (2C), 115.7 (d, *J* = 21.8 Hz, 2C); ^{19}F NMR (376 MHz, $CDCl_3$) δ -104.2.

(4-Bromophenyl)(1-phenyl-1H-1,2,3-triazol-4-yl)methanone (4fa)[3]

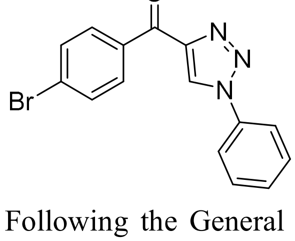

Following the General procedure (I), using 4-bromoacetophenone **4f** (80.0 mg, 0.4 mmol), phenyl azide **3a** (95.3 mg, 0.8 mmol), hexamethyldisilazane (0.335mL, 1.6 mmol) and N,N-dimethylformamide (0.13 mL, 1.6 mmol), 4-methylbenzenesulfonate (10.1 mg, 0.04 mmol). The crude product was then purified by column chromatography (DCM) to afford **4fa** (110.1 mg, 84% yield) as a white solid; ^1H NMR (400 MHz, $(CD_3)_2SO$): δ 9.62 (s, 1H), 8.24 (d, *J* = 8.7 Hz, 2H), 8.03 (d, *J* = 8.3 Hz, 2H), 7.84 (d, *J*

= 8.7 Hz, 2H), 7.65 (t, J = 7.7 Hz, 2H), 7.56 (t, J = 7.4 Hz, 1H); $^{13}C\{^{1}H\}$ NMR (100 MHz, $(CD_3)_2SO$): δ 184.0, 147.0, 136.0, 135.4, 132.0, 131.7, 130.0, 129.5, 128.2, 127.7, 120.7.

(4-Iodophenyl)(1-phenyl-1*H*-1,2,3-triazol-4-yl)methanone (4ga)

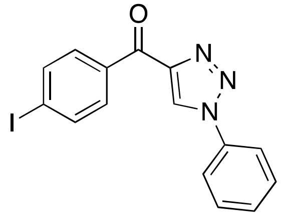

Following the General procedure (I), using 4-iodoacetophenone **4g** (100.0 mg, 0.4 mmol), phenyl azide **3a** (95.3 mg, 0.8 mmol), hexamethyldisilazane (0.335mL, 1.6 mmol) and N,N-dimethylformamide (0.13 mL, 1.6 mmol), 4-methylbenzenesulfonate (10.1 mg, 0.04 mmol). The crude product was then purified by column chromatography (DCM) to afford **4ga** (105.1 mg, 70% yield) as a white solid; m.p. 189-191 °C; ATR-IR (cm^{-1}):1265, 1521, 1557, 1580, 1641, 3131; ^{1}H NMR (400 MHz, $CDCl_3$):
δ 8.70 (s, 1H), 8.26 (d, J = 8.4 Hz, 2H), 7.92 (d, J = 8.1 Hz, 2H), 7.81 (d, J = 8.0 Hz, 2H), 7.59 (t, J = 7.8 Hz, 2H), 7.52 (t, J = 7.2 Hz, 1H); $^{13}C\{^{1}H\}$ NMR (100 MHz, $CDCl_3$): δ 184.6, 148.4, 137.8, 136.3, 135.6, 132.1, 130.1, 129.7, 126.5, 120.9, 101.9. HRMS (EI) m/z: $[M]^+$ calcd for $C_{15}H_{10}IN_3O$ 374.9869; found 374.9872

(4-Nitrophenyl)(1-phenyl-1H-1,2,3-triazol-4-yl)methanone (4ha)

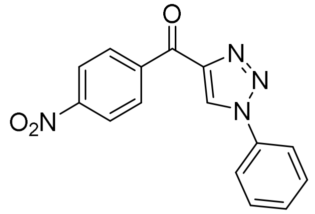

Following the General procedure (I), using 4-nitroacetophenone **4h** (100.0 mg, 0.61 mmol), phenyl azide **3a** (144.3 mg, 1.21 mmol), hexamethyldisilazane (0.503 mL, 2.42 mmol) and N,N-dimethylformamide (0.19 mL, 2.42 mmol), 4-methylbenzenesulfonate (15.8 mg, 0.06 mmol). The crude product was then purified by column chromatography (DCM) to afford **4ha** (117.4 mg, 66% yield) as a white solid; m.p. 212-213°C; ATR-IR (cm^{-1}): 1262, 1525, 1644, 3138; ^{1}H NMR (400 MHz, $CDCl_3$): δ 8.77 (s, 1H), 8.70 (d, J = 8.9 Hz, 2H), 8.39 (d, J = 8.9 Hz, 2H), 7.82 (d, J = 7.9 Hz, 2H), 7.61 (t, J = 7.5 Hz, 2H), 7.55 (t, J = 7.3 Hz, 1H); $^{13}C\{^{1}H\}$ NMR (100 MHz, $(CD_3)_2SO$): δ 176.4, 146.9, 141.8, 136.5, 136.1, 136.0, 130.0, 129.4, 129.0, 127.4, 120.7. HRMS (EI) m/z: $[M]^+$ calcd for $C_{15}H_{10}N_4O_3$ 294.0753; found 294.0746.

(1-Phenyl-1H-1,2,3-triazol-4-yl)(4-(trifluoromethyl)phenyl)methanone(4ia)[3]

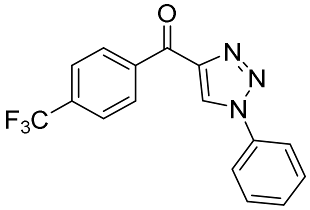

Following the General procedure (I), using 4-(trifluoromethyl)acetophenone **1i** (100.0 mg, 0.53 mmol), phenyl azide **3a** (189.4 mg, 1.59 mmol), hexamethyldisilazane (0.446 mL, 2.13mmol) and N,N-dimethylformamide (0.17 mL, 2.13 mmol), 4-methylbenzenesulfonate (12.6 mg, 0.05 mmol). The crude product was then purified by column chromatography (DCM) to afford **4ia** (118.2 mg, 70% yield) as a white solid; ^{1}H NMR (400 MHz, $CDCl_3$): δ 8.74 (s, 1H), 8.63 (d, J = 8.4 Hz, 2H), 7.85 - 7.79 (m, 4H), 7.61 (t, J = 7.2 Hz, 2H), 7.54 (t, J = 7.2 Hz, 1H); ^{13}C{^{1}H} NMR (100 MHz, $CDCl_3$): δ 184.6, 148.2, 139.2, 136.3, 134.7, 134.4, 131.1, 130.1, 129.8, 126.7, 125.5 (q, J = 3.0 Hz), 120.9. ^{19}F NMR (376 MHz, $CDCl_3$) δ -63.0.

(1-Phenyl-1H-1,2,3-triazol-4-yl)(p-tolyl)methanone (4ja)[5]

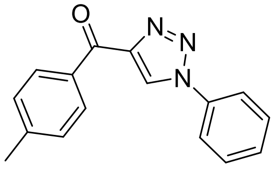

Following the General procedure (I), using 4'-Methylacetophenone **1j** (100.0 mg, 0.75 mmol), phenyl azide **3a** (177.6 mg, 1.49 mmol), hexamethyldisilazane (0.624 mL, 2.98 mmol) and N,N-dimethylformamide (0.23 mL, 2.98 mmol), 4-methylbenzenesulfonate (18.9 mg, 0.08 mmol). The crude product was then purified by column chromatography (DCM) to afford **4ja** (148.7 mg, 76% yield) as an off-white solid; ^{1}H NMR (400 MHz, $CDCl_3$) δ 8.69 (s, 1H), 8.42 (d, J = 8.2 Hz, 2H), 7.81 (d, J = 7.81 Hz, 2H), 7.58 (t, J = 7.6 Hz, 2H), 7.51 (t, J = 7.6 Hz, 2H), 7.35 (d, J = 8.0 Hz, 2H), 2.46 (s, 3H); ^{13}C{^{1}H} NMR (100 MHz, $CDCl_3$) δ 184.9, 148.6, 144.3, 136.3, 133.8, 130.7, 129.8, 129.3, 129.1, 126.3, 120.6, 21.7.

(1-Phenyl-1H-1,2,3-triazol-4-yl)(m-tolyl)methanone (4ka)

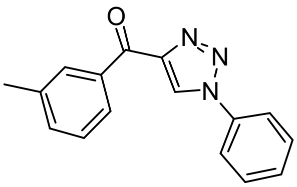

Following the General procedure (I), using 3-methylacetophenone **1k** (100.0 mg, 0.75

mmol), phenyl azide **3a** (177.6 mg, 1.49 mmol), hexamethyldisilazane (0.624 mL, 2.98 mmol) and N,N-dimethylformamide (0.23 mL, 2.98 mmol), 4-methylbenzenesulfonate (18.9 mg, 0.08 mmol). The crude product was then purified by column chromatography (DCM) to afford **4ka** (164.9 mg, 84% yield) as an off-white solid; m.p. 124-126°C; ATR-IR (cm^{-1}): 1268, 1523, 1600, 1644, 2928, 3057, 3127; ^1H NMR (400 MHz, $CDCl_3$) δ 8.69 (s, 1H), 8.30 (d, *J* = 6.6 Hz, 2H), 8.26 (s, 1H), 7.82 (d, *J* = 7.8 Hz, 2H), 7.59 (t, *J* = 7.7 Hz, 2H), 7.51 (t, *J* = 7.3 Hz, 1H), 7.49-7.40 (m, 2H), 2.47 (s, 3H); $^{13}C\{^1H\}$ NMR (100 MHz, $CDCl_3$) δ 185.6, 148.5, 138.1, 136.4, 136.3, 134.2, 130.9, 129.8, 129.4, 126.4, 120.6, 21.4. HRMS (EI) m/z: $[M]^+$ calcd for $C_{16}H_{13}N_3O$ 263.1059; found 263.1054.

(1-Phenyl-1H-1,2,3-triazol-4-yl)(o-tolyl)methanone (4la)

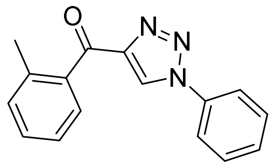

Following the General procedure (II), using 2-methylacetophenone **1l** (100.0 mg, 0.75 mmol), phenyl azide **3a** (177.6 mg, 1.49 mmol), hexamethyldisilazane (0.624 mL, 2.98 mmol) and N,N-dimethylformamide (0.23 mL, 2.98 mmol), 4-methylbenzenesulfonate (18.9 mg, 0.08 mmol). The crude product was then purified by column chromatography (DCM) to afford **4la** (161.6 mg, 82% yield) as a brown solid; m.p.106-109 °C; ATR-IR (cm^{-1}): 1240, 1521, 1669, 3131; ^1H NMR (400 MHz, $(CD_3)_2SO$) δ 9.50 (s, 1H), 8.00 (d, *J* = 7.8 Hz, 2H), 7.70 (d, *J* = 7.6 Hz, 1H), 7.62 (t, *J* = 7.7 Hz, 2H), 7.58 – 7.46 (m, 2 H), 7.38 – 7.32 (m, 2H), 2.37 (s, 3H); $^{13}C\{^1H\}$ NMR (100 MHz, $(CD_3)_2SO$) δ 188.8, 147.7, 137.4, 136.1, 131.1, 129.9, 129.6, 129.3, 127.6, 125.4, 120.7, 19.8. HRMS (EI) m/z: $[M]^+$ calcd for 263.1059; found 263.1053

(4-Methoxyphenyl)(1-phenyl-1H-1,2,3-triazol-4-yl)methanone (4ma)[4]

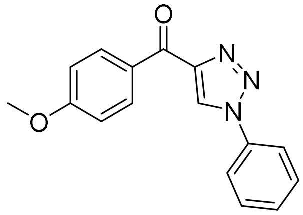

Following the General procedure (I), using 4-methoxylacetophenone **1m** (100.0 mg, 0.67 mmol), phenyl azide **3a** (237.6 mg, 2.00 mmol), hexamethyldisilazane (0.558 mL, 2.67 mmol) and N,N-dimethylformamide (0.2 mL, 2.67 mmol), 4-methylbenzenesulfonate (16.8 mg, 0.067 mmol). The product was purified by flash column chromatography (DCM) afforded **4ma** (147.7 mg, 80% yield) as a brown solid;

^{1}H NMR (400 MHz, $CDCl_3$) δ 8.68 (s, 1H), 8.58 (d, *J* = 8.5 Hz, 2H), 7.81 (d, *J* = 8.2 Hz, 2H), 7.58 (t, *J* = 7.7 Hz, 2H), 7.51 (t, *J* = 7.1Hz, 1H), 7.03 (d, *J* = 8.6 Hz, 2H), 3.92 (s, 3H); $^{13}C\{^{1}H\}$ NMR (100 MHz, $CDCl_3$) δ 183.8, 164.0, 149.0, 136.5, 133.2, 130.0, 129.5, 129.4, 126.3, 120.8, 113.8, 55.6.

(1-Phenyl-1H-1,2,3-triazol-4-yl)(thiophen-2-yl)methanone (4na)[5]

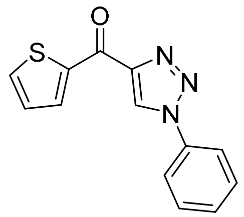

Following the General procedure (I), using 2-acetylthiophene **1n** (100.0 mg, 0.79 mmol), phenyl azide **3a** (189 mg, 1.59 mmol), hexamethyldisilazane (0.664 mL, 3.17 mmol) and N,N-dimethylformamide (0.25 mL, 3.17 mmol), 4-methylbenzenesulfonate (19.9 mg, 0.08 mmol). The crude product was then purified by column chromatography (DCM) to afford **4na** (175.9 mg, 86% yield). as an off-white solid; ^{1}H NMR (400 MHz, $(CD_3)_2SO$) δ 9.63 (s, 1H), 8.63 (dd, *J* = 3.8, 1.1 Hz, 1H), 8.18 (dd, *J* = 4.9, 1.1 Hz, 1H), 8.05 (d, *J* = 7.5, 2H), 7.65 (t, *J* = 7.7 Hz, 2H), 7.56 (t, *J* = 7.4 Hz, 1H), 7.38 (dd, *J* = 4.9, 3.9 Hz, 1H); $^{13}C\{^{1}H\}$ NMR (100 MHz, $(CD_3)_2SO$) δ 176.4, 146.9, 141.8, 136.5, 136.1, 136.0, 130.0, 129.5, 129.0, 127.4, 120.7.

Furan-2-yl(1-phenyl-1H-1,2,3-triazol-4-yl)methanone (4oa)

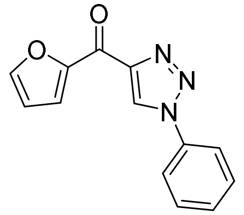

Following the General procedure (I), using 2-furyl methyl ketone **1o** (100.0 mg, 0.91 mmol), phenyl azide **3a** (216.8 mg, 1.82 mmol), hexamethyldisilazane (0.761 mL, 3.63 mmol) and N,N-dimethylformamide (0.28 mL, 3.63 mmol), 4-methylbenzenesulfonate (22.6 mg, 0.09 mmol). The crude product was then purified by column chromatography (DCM) to afford **4oa** (175.9 mg, 70% yield). as an brown solid; m.p.134-137 °C**;** ATR-IR (cm^{-1}): 1287, 1397, 1464, 1528, 1563, 1640, 3112, 3139;^{1}H NMR (400 MHz, $CDCl_3$) δ 8.73 (s, 1H), 8.27 (d, *J* = 3.6 Hz, 1H), 7.91 - 7.73 (m, 3H), 7.59 (t, *J* = 7.6 Hz, 2H), 7.52 (t, *J* = 7.4 Hz, 1H), 6.75 - 6.61 (m, 1H); $^{13}C\{^{1}H\}$ NMR (100 MHz, $(CD_3)_2SO$) δ 171.3, 150.5, 149.0, 146.2, 136.1, 129.9, 129.4, 127.2, 122.4, 120.7, 112.9. HRMS (EI) m/z: $[M]^+$ calcd for 239.0695; found 239.0687.

Naphthalen-2-yl(1-phenyl-1H-1,2,3-triazol-4-yl)methanone(4pa)[4]

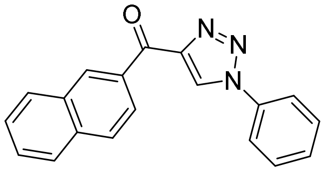

Following the General procedure (I), using 2-acetonaphthone **1p** (100.0 mg, 0.59 mmol), phenyl azide **3a** (140.1 mg, 1.18 mmol), hexamethyldisilazane (0.493 mL, 2.35 mmol) and N,N-dimethylformamide (0.18 mL, 2.35 mmol), 4-methylbenzenesulfonate (14.9 mg, 0.06 mmol). The crude product was then purified by column chromatography (DCM) to afford **4pa** (164.6 mg, 94% yield) as a off-white solid; ^{1}H NMR (400 MHz, $CDCl_3$) δ 9.32 (s, 1H), 8.76 (s, 1H), 8.40 (d, J = 8.7 Hz, 1H), 8.08 (d, J = 8.0 Hz, 1H), 7.98 (d, J = 8.7 Hz, 1H), 7.92 (d, J = 8.2 Hz, 1H), 7.85 (d, J = 7.9 Hz, 2H), 7.69 - 7.49 (m, 5H); ^{13}C{^{1}H} NMR (100 MHz, $CDCl_3$) δ 185.2, 148.9, 136.5, 135.8, 133.7, 133.7, 132.6, 130.2, 130.0, 129.6, 128.8, 128.3, 127.8, 126.7, 126.5, 125.5, 120.8.

(3,4-Dimethylphenyl)(1-phenyl-1H-1,2,3-triazol-4-yl)methanone (4qa)

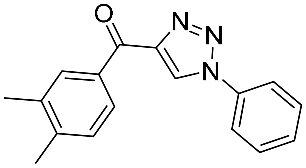

Following the General procedure (I), using 3,4-dimethylacetophenone **1q** (100.0 mg, 0.67 mmol), phenyl azide **3a** (241.1 mg, 2.02 mmol), hexamethyldisilazane (0.566 mL, 2.68 mmol) and N,N-dimethylformamide (0.21 mL, 2.68 mmol), 4-methylbenzenesulfonate (16.8 mg, 0.07 mmol). The crude product was then purified by column chromatography (DCM) to afford **4qa** (122.9 mg, 66% yield) as a off-white solid; m.p. 158-160 °C; ATR-IR (cm^{-1}): 1258, 1521, 1595, 1634, 3066, 3121; ^{1}H NMR (400 MHz, $CDCl_3$) δ 8.69 (s, 1H), 8.26 (dd, J = 7.9, 1.7 Hz, 1H), 8.23 (s, 1H), 7.81 (d, J = 7.9 Hz, 2H), 7.56 (t, J = 7.6 Hz, 2H), 7.49 (t, J = 7.4 Hz,1H), 7.29 (d, J = 7.9 Hz, 1 H), 2.36 (d, J = 7.6 Hz, 6 H); ^{13}C{^{1}H} NMR (100 MHz, $CDCl_3$) δ 185.4, 143.2, 136.9, 136.5, 134.3, 134.6, 130.0, 129.8, 129.5, 128.6, 126.3, 120.8, 20.2, 19.9. HRMS (EI) m/z: $[M]^+$ calcd for $C_{17}H_{15}N_3O$ 277.1215; found 277.1212.

(3,4-Dichlorophenyl)(1-phenyl-1H-1,2,3-triazol-4-yl)methanone(4ra)

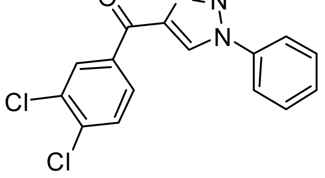

Following the General procedure (I), using 3,4-dichloroacetophenone **1r** (100.0 mg, 0.53 mmol), phenyl azide **3a** (126.3 mg, 1.06 mmol), hexamethyldisilazane (0.444 mL, 2.12 mmol) and N,N-dimethylformamide (0.17 mL, 2.12 mmol), 4-methylbenzenesulfonate (13.3 mg, 0.05 mmol). The crude product was then purified by column chromatography (DCM) to afford **4ra** (100.7 mg, 60% yield) as a off-white solid; m.p. 209 -211°C; ATR-IR (cm^{-1}):1240, 1267, 1523, 1582, 1639, 3086, 3136; ^{1}H NMR (400 MHz, $(CD_3)_2SO$) δ 9.66 (s, 1H), 8.49 (d, *J* = 2.0 Hz, 1H), 8.24 (dd, *J* = 8.4, 2.0 Hz, 1H), 8.02 (d, *J* = 7.4 Hz, 2H), 7.90 (d, *J* = 8.4 Hz, 1H), 7.64 (t, *J* = 7.7 Hz, 2H), 7.56 (t, *J* = 7.4 Hz, 1H); $^{13}C\{^{1}H\}$ NMR (100 MHz, $(CD_3)_2SO$) δ 182.6, 176.9, 146.7, 136.5, 136.3, 136.0, 131.8, 131.1, 130.0, 130.0, 129.5, 129.4, 120.7. HRMS (EI) m/z: $[M]^+$ calcd for $C_{15}H_9Cl_2N_3O$ 317.0123; found 317.0131.

1,3-Bis(4-chlorophenyl)propane-1,3-dione (**2b**)[7]

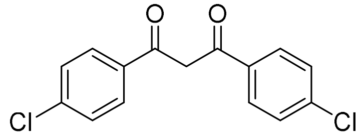

Following the General procedure (III), using 1-(4-chlorophenyl)ethan-1-one (0.26 mL, 2 mmol), methyl 4-chlorobenzoate (375.3 mg, 2.2 mmol), Sodium hydride (60% in mineral oil, 200.0 mg, 5.0 mmol) and tetrahydrofuran (7.0 mL) The crude product was then purified by precipitation in Ethyl acetate to afford **2b** (229.1 mg, 40% yield) as a white solid; ^{1}H NMR (400 MHz, $CDCl_3$) δ 7.92 (d, *J* = 8.6 Hz, 4H), 7.47 (d, *J* = 8.6 Hz, 4H), 6.77 (s, 1H).

1,3-Bis(4-methoxyphenyl)propane-1,3-dione (**2c**)[7]

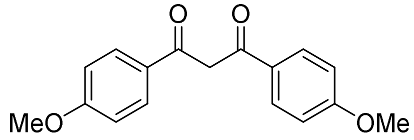

Following the General procedure(III), using1-(4-methoxyphenyl)ethan-1-one (750.9 mg, 5 mmol), methyl 4-methoxybenzoate (913.9 mg, 5.5 mmol), Sodium hydride (60% in mineral oil, 500.0 mg, 12.5 mmol) and tetrahydrofuran (17.5 mL). The crude product was then purified by flash column chromatography (DCM) to afford **2c** (1071.4 mg, 75% yield) as brown solid; ^{1}H NMR (400 MHz, $CDCl_3$) δ 7.96 (d, *J* = 8.7 Hz, 4H), 6.98 (d, *J* = 8.7 Hz, 4H), 6.73 (s, 1H), 3.89 (s, 6H).

1,3-Di-*m*-tolylpropane-1,3-dione (2d)[8]

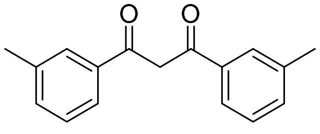

Following the General procedure (III), using 1-(*m*-tolyl)ethan-1-one (0.40 mL, 3 mmol), methyl 3-methylbenzoate (0.964 mL, 7.5 mmol), Sodium hydride (60% in mineral oil, 300mg, 7.5 mmol) and tetrahydrofuran (10.5 mL). The crude product was then purified by precipitation in Ethyl acetate to afford **2d** (534.7 mg, 70% yield) as a red oil; ^{1}H NMR (400 MHz, $CDCl_3$) δ 7.85 – 7.72 (m, 4H), 7.43 – 7.33 (m, 4H), 6.83 (s, 1H), 2.45 (s, 6H).

***N,N*-Dimethyl-3-oxo-3-phenylpropanamide (2g)**[9,10]

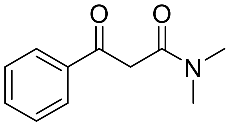

Ethyl 3-oxo-3-phenylpropanoate (90% 1.92 mL, 10.0 mmol), dimethylammonium chloride (1.63 g, 20.0 mmol) and DMAP (5.62 g, 4.6 mmol,) were dissolved in 20 mL of toluene. The mixture was stirred sufficiently under reflux until the reaction was completed as judged by TLC. After cooling to room temperature, the solvent was quenched with water and extracted with ethyl acetate. The ethyl acetate layer was washed with brine, and dried over anhydrous Na_2SO_4. After the solvent was evaporated in vacuo, the residue was purified by column chromatography to afford pure **2g** (1127 mg, 59% yield) as a light yellow solid; ^{1}H NMR (400 MHz, $CDCl_3$) δ ketone/enol 1:0.6 ketone 8.04 – 8.00 (m, 2H), 7.59 (t, J = 7.6 Hz, 1H), 7.48 (t, J = 7.6 Hz, 2H), 4.11 (s, 2H), 3.05 (s, 3H), 2.99 (s, 3H); *enol* δ 7.81 – 7.75 (m, 2H), 7.44 – 7.38 (m, 3H), 5.79 (s, 1H), 3.07 (s, 6H).

(1,5-Diphenyl-1H-1,2,3-triazol-4-yl)(phenyl)methanone (5aa)[11]

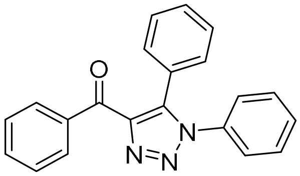

Following the General procedure (IV), using dibenzoylmethane **2a** (200.0 mg, 0.892 mmol), phenyl azide **3a** (212.5 mg, 1.784 mmol), hexamethyldisilazane (0.375 mL, 1.784 mmol) and N,N-dimethylformamide (0.138 mL, 1.784mmol). The crude product was then purified by column chromatography (DCM/EtOAc, 50:1) to afford **5aa** (265.2 mg, 91% yield) as a white solid; ^{1}H NMR (400 MHz, $CDCl_3$) δ 8.29 (d, J = 7.6 Hz, 2H), 7.60 (t, J = 7.6 Hz, 1H), 7.50 (t, J = 7.6 Hz, 2H), 7.44 – 7.31 (m, 10H); $^{13}C\{^1H\}$

NMR (100 MHz, $CDCl_3$): δ 186.8, 143.7, 141.3, 137.3, 136.0, 133.2, 130.8, 130.3, 129.9, 129.6, 129.4, 128.6, 128.4, 126.1, 125.4.

Phenyl(5-phenyl-1-(p-tolyl)-1H-1,2,3-triazol-4-yl)methanone (**5ab**)[11]

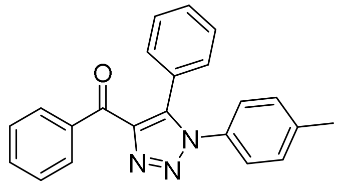

Following the General procedure (IV), using dibenzoylmethane **2a** (200.0 mg, 0.892 mmol), 1-azido-4-methylbenzene **3b** (475.0 mg, 3.568 mmol), hexamethyldisilazane (0.375 mL, 1.784 mmol) and N,N-dimethylformamide (0.138 mL, 1.784 mmol). The crude product was then purified by column chromatography (DCM/EtOAc, 50:1) to afford **5ab** (205.8 mg, 68% yield) as a light yellow solid; ^{1}H NMR (400 MHz, $CDCl_3$) δ 8.28 (d, J = 7.4 Hz, 2H), 7.59 (t, J = 7.4 Hz, 1H), 7.50 (t, J = 7.4 Hz, 2H), 7.44 – 7.30 (m, 5H), 7.23 – 7.17 (m, 4H), 2.38 (s, 3H); ^{13}C{^{1}H} NMR (100 MHz, $CDCl_3$): δ 186.8, 143.6, 141.2, 139.8, 137.3, 133.5, 133.2, 130.8, 130.3, 130.0, 129.8, 128.5, 128.3, 126.2, 125.1, 21.3.

Phenyl(5-phenyl-1-(m-tolyl)-1H-1,2,3-triazol-4-yl)methanone (**5ac**)[11]

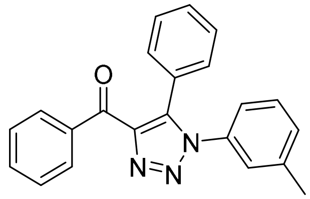

Following the General procedure (IV), using dibenzoylmethane **2a** (200.0 mg, 0.892 mmol), 1-azido-3-methylbenzene **3c** (191.2 mg, 1.784 mmol), hexamethyldisilazane (0.375 mL, 1.784 mmol) and N,N-dimethylformamide (0.138 mL, 1.784 mmol). The crude product was then purified by column chromatography (DCM/EtOAc, 50:1) to afford **5ac** (163.3 mg, 54% yield) as a white solid; ^{1}H NMR (400 MHz, $CDCl_3$) δ 8.28 (d, J = 7.4 Hz, 2H), 7.60 (t, J = 7.4 Hz, 1H), 7.50 (t, J = 7.4 Hz, 2H), 7.43 – 7.31 (m, 5H), 7.28 – 7.20 (m, 3H), 7.02 (d, J = 6.7 Hz, 1H), 2.35 (s, 3H); ^{13}C{^{1}H} NMR (100 MHz, $CDCl_3$): δ 186.9, 143.6, 141.2, 139.8, 137.3, 135.9, 133.2, 130.8, 130.4, 130.3, 129.9, 129.1, 128.5, 128.4, 126.2, 126.0, 122.4, 21.4.

Phenyl(5-phenyl-1-(o-tolyl)-1H-1,2,3-triazol-4-yl)methanone (5ad)[11]

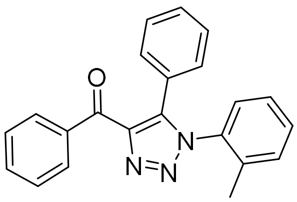

Following the General procedure (IV), using dibenzoylmethane **2a** (200.0 mg, 0.892 mmol), 1-azido-2-methylbenzene **3d** (237.0 mg, 1.784 mmol), hexamethyldisilazane (0.375 mL, 1.784 mmol) and N,N-dimethylformamide (0.138 mL, 1.784 mmol). The crude product was then purified by column chromatography (DCM/EtOAc, 50:1) to afford **5ad** (105.2 mg, 35% yield) as a white solid; ^{1}H NMR (400 MHz, $CDCl_3$) δ 8.27 (d, J = 8.1 Hz, 2H), 7.57 (t, J = 7.4 Hz, 1H), 7.47 (t, J = 7.6 Hz, 2H), 7.37 – 7.28 (m, 2H), 7.26 – 7.17 (m, 4H), 2.00 (s, 3H); ^{13}C{^{1}H} NMR (100 MHz, $CDCl_3$): δ 186.9, 142.9, 142.4, 137.3, 135.1, 134.9, 133.2, 131.3, 130.8, 130.4, 129.9, 129.8, 128.3, 127.8, 126.9, 125.7, 17.7.

(1-(4-Ethylphenyl)-5-phenyl-1H-1,2,3-triazol-4-yl)(phenyl)methanone (**5ae**)

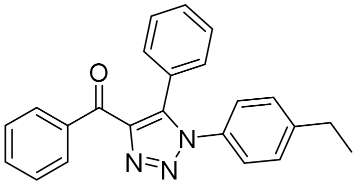

Following the General procedure (IV), using dibenzoylmethane **2a** (200.0 mg, 0.892 mmol), 1-azido-4-ethylbenzene **3e** (262.5 mg, 1.784 mmol), hexamethyldisilazane (0.375 mL, 1.784 mmol) and N,N-dimethylformamide (0.138 mL, 1.784 mmol). The crude product was then purified by column chromatography (DCM/EtOAc, 50:1) to afford **5ae** (315.3 mg, 70% yield) as a brown solid; ^{1}H NMR (400 MHz, $CDCl_3$) δ 8.29 (d, J = 7.4 Hz, 2H), 7.59 (t, J = 7.4 Hz, 1H), 7.49 (t, J = 7.4 Hz, 2H), 7.43 – 7.32 (m, 5H), 7.25 – 7.20 (m, 4H), 2.67 (q, J = 7.6 Hz, 2H), 1.24 (t, J = 7.6 Hz, 3H); ^{13}C{^{1}H} NMR (100 MHz, $CDCl_3$): δ 186.8, 145.9, 143.6, 141.1, 137.3, 133.6, 133.1, 130.8, 130.3, 129.8, 128.8, 128.5, 128.3, 126.2, 125.1, 28.5, 15.2.

(1-(4-(tert-butyl)phenyl)-5-phenyl-1H-1,2,3-triazol-4-yl)(phenyl)methanone (5af)

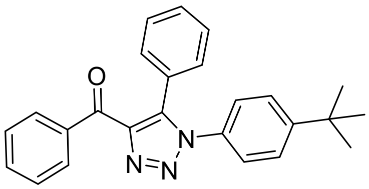

Following the General procedure (IV), using dibenzoylmethane **2a** (200.0 mg, 0.892 mmol), 1-azido-4-(*tert*-butyl)benzene **3f** (312.5 mg, 1.784 mmol), hexamethyldisilazane (0.375 mL, 1.784 mmol) and N,N-dimethylformamide (0.138 mL, 1.784 mmol). The crude product was then purified by column chromatography (DCM/EtOAc, 50:1) to afford **5af** (184.2 mg, 54% yield) as a yellow solid; m.p. 122-125°C; ATR-IR (cm^{-1}): 1248, 1506, 1546, 1660, 2966, 3169.^{1}H NMR (400 MHz, $CDCl_3$) δ 8.28 (d, *J* = 7.1 Hz,2H), 7.60 (t, *J* = 7.4 Hz, 1H), 7.50 (t, *J* = 7.6 Hz, 2H), 7.43 -7.35 (m, 7H), 7.26 – 7.22 (m, 2H), 1.32 (s, 9H); $^{13}C\{^{1}H\}$ NMR (100 MHz, $CDCl_3$): δ 186.7, 152.7, 143.5, 141.0, 137.2, 133.2, 133.0, 130.7, 130.2, 129.7, 128.4, 128.2, 126.2, 126.1, 124.6, 34.7, 31.1.HRMS (EI) m/z: $[M]^+$ calcd for $C_{25}H_{23}N_3O$ 381.1841; found 381.1836.

(1-(4-Octylphenyl)-5-phenyl-1H-1,2,3-triazol-4-yl)(phenyl)methanone (**5ag**)

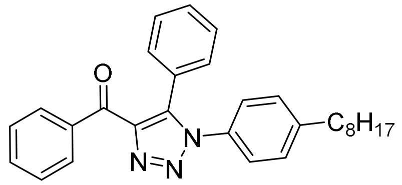

Following the General procedure (IV), using dibenzoylmethane **2a** (200.0 mg, 0.892 mmol), 1-azido-4-octylbenzene **3g** (412.6 mg, 1.784 mmol), hexamethyldisilazane (0.375 mL, 1.784 mmol) and N,N-dimethylformamide (0.138 mL, 1.784 mmol). The crude product was then purified by column chromatography (DCM/EtOAc, 50:1) to afford **5ag** (232.0 mg, 60% yield) as a brown solid, m.p. 65-66 °C; ATR-IR (cm^{-1}): 1248, 1515, 1597, 1665, 2851, 2292, 3060.^{1}H NMR (400 MHz, $CDCl_3$) δ 8.28 (dd, *J* =7.4, 1.3 Hz, 2H), 7.59 (tt, *J* = 7.4, 1.3 Hz, 1H), 7.50 (t, *J* = 7.4 Hz, 2H), 7.44 – 7.31 (m, 5H), 7.22 (d, *J* = 8.9 Hz, 2H), 7.19 (d, *J* = 8.9 Hz, 2H), 2.62 (t, *J* = 7.6 Hz, 2H), 1.67-1.57 (m, 2H), 1.37 – 1.21 (m, 10H), 0.88 (t, *J* = 6.9 Hz, 3H); $^{13}C\{^{1}H\}$ NMR (100 MHz, $CDCl_3$): δ 186.9, 144.8, 143.6, 141.2, 137.3, 133.6, 133.2, 130.8, 130.3, 129.9, 129.3, 128.5, 128.4, 126.3, 125.2, 35.6, 31.9, 31.2, 29.5, 29.3, 29.3, 22.8, 14.2. HRMS (EI) m/z : $[M]^+$ calcd for $C_{29}H_{31}N_3O$ 437.2467; found 437.2464

(1-(3,5-Dimethylphenyl)-1H-1,2,3-triazol-4-yl)(phenyl)methanone (5ah)

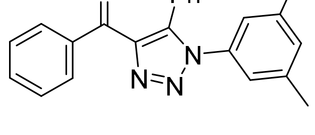

Following the General procedure (IV), using dibenzoylmethane **2a** (200.0 mg, 0.892 mmol), 1-azido-3,5-dimethylbenzene **3h** (263.0 mg, 1.784 mmol),

hexamethyldisilazane (0.375 mL, 1.784 mmol) and N,N-dimethylformamide (0.138 mL, 1.784 mmol). The crude product was then purified by column chromatography (DCM/EtOAc, 50:1) to afford **5ah** (182.0 mg, 58% yield) as a yellow oil; ATR-IR (cm^{-1}): 1243, 1513, 1577, 1728, 2360, 2962.^{1}H NMR (400 MHz, $CDCl_3$) δ 8.28 (d, *J* = 7.2 Hz, 2H), 7.59 (t, *J* = 7.4 Hz,1H), 7.49 (t, *J* = 7.6 Hz,2H), 7.43 – 7.31 (m,5H), 7.04 (s,1H), 6.97-6.88 (m, 2H) ,2.26 (s, 6H);^{13}C{^{1}H} NMR (100 MHz, $CDCl_3$): δ 186.8, 143.4, 141.1, 139.2, 137.2, 135.7, 133.1, 131.2, 130.7, 130.2, 129.8, 128.4, 128.3, 126.2, 123.0, 21.2. HRMS (EI) m/z: $[M]^+$ calcd for $C_{23}H_{19}N_3O$ 353.1528; found 353.1528

(1-(4-Methoxyphenyl)-5-phenyl-1H-1,2,3-triazol-4-yl)(phenyl)methanone (5ai)[11]

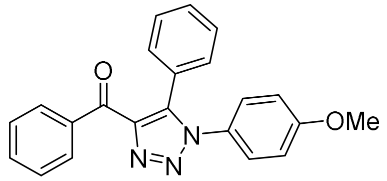

Following the General procedure (IV), using dibenzoylmethane **2a** (200.0 mg, 0.892 mmol), 1-azido-4-methoxybenzene **3i** (266.1 mg, 1.784 mmol), hexamethyldisilazane (0.375 mL, 1.784 mmol) and N,N-dimethylformamide (0.138 mL, 1.784 mmol). The crude product was then purified by column chromatography (DCM/EtOAc, 50:1) to afford **5ai** (168.5 mg, 53% yield) as a light yellow solid; ^{1}H NMR (400 MHz, $CDCl_3$) δ 8.28 (d, *J* = 7.4 Hz, 2H), 7.59 (t, *J* = 7.4 Hz, 1H), 7.49 (t, *J* = 7.4 Hz, 2H), 7.43 – 7.31 (m, 5H), 7.24 (d, *J* = 9.0 Hz, 2H), 6.89 (d, *J* = 9.0 Hz, 2H), 3.81 (s, 3H); ^{13}C{^{1}H} NMR (100 MHz, $CDCl_3$): δ 186.8, 160.2, 143.5, 141.2, 137.3, 133.1, 130.8, 130.3, 129.8, 128.8, 128.5, 128.3, 126.7, 126.2, 114.5, 55.6.

(1-(4-Fluorophenyl)-5-phenyl-1H-1,2,3-triazol-4-yl)(phenyl)methanone (5aj)[11]

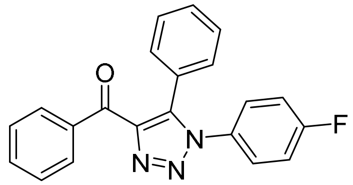

Following the General procedure (IV), using dibenzoylmethane **2a** (200.0 mg, 0.892 mmol), 1-azido-4-fluorobenzene **3j** (224.6 mg, 1.784 mmol), hexamethyldisilazane (0.375 mL, 1.784 mmol) and N,N-dimethylformamide (0.138 mL, 1.784 mmol) The crude product was then purified by column chromatography (DCM/EtOAc, 50:1) to afford **5aj** (271.0 mg, 89% yield) as a light yellow solid; ^{1}H NMR (400 MHz, $CDCl_3$) δ 8.28 (d, *J* = 7.7 Hz, 2H), 7.60 (t, *J* = 7.7 Hz, 1H), 7.49 (t, *J* = 7.7 Hz, 2H), 7.45 – 7.28 (m, 7H), 7.09 (t, *J* = 8.3 Hz, 2H); ^{13}C{^{1}H} NMR (100 MHz, $CDCl_3$): .δ 186.6 , 162.8 (d, *J* = 250.8 Hz), 143.6 , 141.4 , 137.1, 133.2, 132.0 (d, *J* = 3.1 Hz), 130.7, 130.2,

130.0 , 128.6, 128.3, 127.2 (d, J = 8.9 Hz), 125.9, 116.5 (d, J = 23.2 Hz). ^{19}F NMR (376 MHz, CDCl3) δ -110.33.

(1-(4-Chlorophenyl)-5-phenyl-1H-1,2,3-triazol-4-yl)(phenyl)methanone (**5ak**)[11]

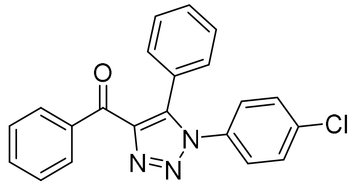

Following the General procedure (IV), using dibenzoylmethane **2a** (200.0 mg, 0.892 mmol), 1-azido-4-chlorobenzene **3k** (273.9 mg, 1.784 mmol), hexamethyldisilazane (0.375 mL, 1.784 mmol) and N,N-dimethylformamide (0.138 mL, 1.784 mmol). The crude product was then purified by column chromatography (DCM/EtOAc, 50:1) to afford **5ak** (315.3 mg, 98% yield) as a brown solid; ^{1}H NMR (400 MHz, $CDCl_3$) δ 8.27 (d, J = 7.4 Hz, 2H), 7.61 (t, J = 7.4 Hz, 1H), 7.50 (t, J = 7.4 Hz, 2H), 7.47 – 7.31 (m, 7H), 7.30 – 7.24 (m, 2H); ^{13}C{^{1}H} NMR (100 MHz, $CDCl_3$): δ 186.6, 143.8, 141.2, 137.1, 135.7, 134.4, 133.3, 130.8, 130.3, 130.2, 129.7, 128.8, 128.4, 126.5, 125.9.

(1-(4-Bromophenyl)-5-phenyl-1H-1,2,3-triazol-4-yl)(phenyl)methanone (**5al**)[11]

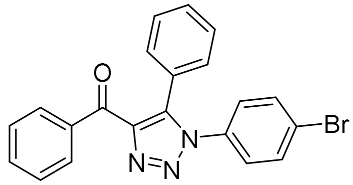

Following the General procedure (IV), using dibenzoylmethane **2a** (200.0 mg, 0.892 mmol), 1-azido-4-bromobenzene **3l** (353.0 mg, 1.784 mmol), hexamethyldisilazane (0.375 mL, 1.784 mmol) and N,N-dimethylformamide (0.138 mL, 1.784 mmol). The crude product was then purified by column chromatography (DCM/EtOAc, 50:1) to afford **5al** (341.5 mg, 94% yield) as a brown solid; ^{1}H NMR (400 MHz, $CDCl_3$) δ 8.27 (d, J = 8.0 Hz, 2H), 7.60 (t, J = 8.0 Hz, 1H), 7.57 – 7.28 (m, 9H), 7.21 (d, J = 8.4 Hz, 2H); ^{13}C{^{1}H} NMR (100 MHz, $CDCl_3$): δ 186.6, 143.8, 141.2, 137.1, 134.9, 133.3, 132.7, 130.8, 130.3, 130.2, 128.8, 128.4, 126.7, 125.8, 123.7.

(1-(4-Iodophenyl)-5-phenyl-1H-1,2,3-triazol-4-yl)(phenyl)methanone (**5am**)[11]

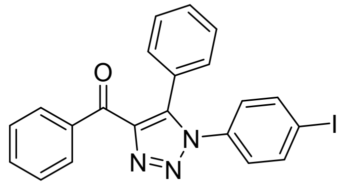

Following the General procedure (IV), using dibenzoylmethane **2a** (200.0 mg, 0.892 mmol), 1-azido-4-iodobenzene **3m** (437.1 mg, 1.784 mmol), hexamethyldisilazane (0.375 mL, 1.784 mmol) and N,N-dimethylformamide (0.138 mL, 1.784 mmol). The crude product was then purified by column chromatography (DCM/EtOAc, 50:1) to afford **5am** (349.5 mg, 87% yield) as a brown solid; ^{1}H NMR (400 MHz, $CDCl_3$) δ 8.27 (d, *J* = 7.7 Hz, 2H), 7.73 (d, *J* = 8.1 Hz, 2H), 7.60 (t, *J* = 7.7 Hz, 1H), 7.49 (t, *J* = 7.7 Hz, 2H), 7.46 – 7.30 (m, 5H), 7.07 (d, *J* = 8.1 Hz, 2H); ^{13}C{^{1}H} NMR (100 MHz, $CDCl_3$): δ 186.6, 143.8, 141.1, 138.6, 137.1, 135.6, 133.3, 130.7, 130.2, 130.1, 128.7, 128.4, 126.7, 125.8, 95.3.

(1-(3-Chlorophenyl)-5-phenyl-1H-1,2,3-triazol-4-yl)(phenyl)methanone (5an)[11]

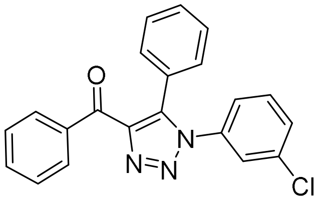

Following the General procedure (IV), using dibenzoylmethane **2a** (200.0 mg, 0.892 mmol), 1-azido-2-chlorobenzene **3n** (273.93.1 mg, 1.784 mmol), hexamethyldisilazane (0.375 mL, 1.784 mmol) and N,N-dimethylformamide (0.138 mL, 1.784 mmol). The crude product was then purified by column chromatography (DCM/EtOAc, 50:1) to afford **5an** (320.9 mg, 88% yield) as a white solid; ^{1}H NMR (400 MHz, $CDCl_3$) δ 8.27 (d, *J*=7.1 Hz, 2H), 7.61 (t, *J*=7.4 Hz, 1H), 7.54–7.29 (m, 10H), 7.14 (ddd, *J* = 8.0, 2.0, 1.0 Hz, 1H); ^{13}C{^{1}H} NMR (100 MHz, $CDCl_3$) δ 186.6, 143.8, 141.3, 137.1, 136.9, 135.2, 133.4, 130.8, 130.4, 130.2 (3C), 129.8, 128.8, 128.4, 125.7, 125.6, 123.4.

(1-(2-Chlorophenyl)-5-phenyl-1H-1,2,3-triazol-4-yl)(phenyl)methanone (**5ao**)[11]

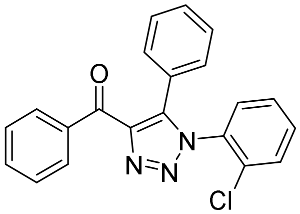

Following the General procedure (IV), using dibenzoylmethane **2a** (200.0 mg, 0.892 mmol), 1-azido-2-chlorobenzene **3o** (273.9 mg, 1.784 mmol), hexamethyldisilazane (0.375 mL, 1.784 mmol) and N,N-dimethylformamide (0.138 mL, 1.784 mmol). The crude product was then purified by column chromatography (DCM/EtOAc, 50:1) to afford **5ao** (225.8 mg, 70% yield) as a white solid; ^{1}H NMR (400 MHz, $CDCl_3$) δ 8.31 (d, *J* = 7.4 Hz, 2H), 7.61 (t, *J* = 7.4 Hz, 1H), 7.52 (d, *J* = 8.0 Hz, 2H), 7.50 – 7.28 (m, 9H); ^{13}C{^{1}H} NMR (100 MHz, $CDCl_3$): δ 186.7, 143.2, 142.9, 137.2, 133.8, 133.3,

132.0, 131.8, 130.8, 130.7, 130.0, 129.9, 129.6, 128.4 (2C), 127.8, 125.6.

(1-(4-Nitrophenyl)-5-phenyl-1H-1,2,3-triazol-4-yl)(phenyl)methanone (5ap)[12]

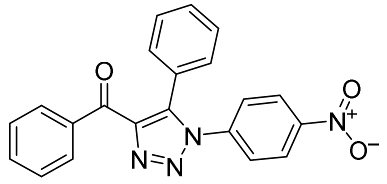

Following the General procedure (IV), using dibenzoylmethane **2a** (200.0 mg, 0.892 mmol), 1-azido-4-nitrobenzene **3p** (293.0 mg, 1.784 mmol), hexamethyldisilazane (0.375 mL, 1.784 mmol) and N,N-dimethylformamide (0.138 mL, 1.784 mmol). The crude product was then purified by column chromatography (DCM/EtOAc, 50:1) to afford **5ap** (306.0 mg, 92% yield) as a light yellow solid; ^{1}H NMR (400 MHz, $CDCl_3$) δ 8.35 – 8.22 (m, 4H), 7.63 (t, *J* = 7.4 Hz, 1H), 7.57 – 7.40 (m, 7H), 7.36 (dd, *J* = 8.2, 1.4 Hz, 2H); ^{13}C{^{1}H} NMR (100 MHz, $CDCl_3$): δ 186.3, 147.8, 144.1, 141.4, 140.7, 136.9, 133.5, 130.7, 130.6, 130.2, 129.0, 128.4, 125.7, 125.4, 124.9.

Methyl 4-(4-benzoyl-5-phenyl-1H-1,2,3-triazol-1-yl)benzoate (5aq)

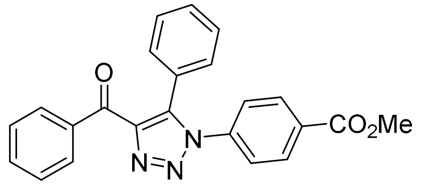

Following the General procedure (IV), using dibenzoylmethane **2a** (200.0 mg, 0.892 mmol), methyl 4-azidobenzoate **3q** (316.0 mg, 1.784 mmol), hexamethyldisilazane (0.375 mL, 1.784 mmol) and N,N-dimethylformamide (0.138 mL, 1.784 mmol). The crude product was then purified by column chromatography (DCM/EtOAc, 50:1) to afford **5aq** (307.5 mg, 90% yield) as a white solid, m.p. 186-187 °C; ATR-IR (cm^{-1}): 1272, 1433, 1608, 1650, 1719, 2953, 3031. ^{1}H NMR (400 MHz, $CDCl_3$) δ 8.27 (d, *J* = 7.4 Hz, 2H), 8.08 (d, *J* = 8.7 Hz, 2H), 7.61 (t, *J* = 7.4 Hz, 1H), 7.50 (t, *J* = 7.4 Hz, 2H), 7.46 – 7.30 (m, 7H), 3.93 (s, 3H); ^{13}C{^{1}H} NMR (100 MHz, $CDCl_3$): δ 186.6, 165.9, 143.9, 141.3, 139.4, 137.1, 133.4, 131.1, 130.8, 130.8, 130.2 (2C), 128.8, 128.4, 125.8, 125.0, 52.6. HRMS (EI) m/z: [M]$^+$ calcd for $C_{23}H_{17}N_3O_3$ 383.1270; found 383.1262

(1-(3-Bromophenyl)-5-phenyl-1H-1,2,3-triazol-4-yl)(phenyl)methanone (**5au**)

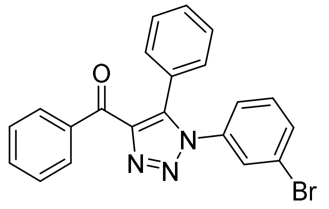

Following the General procedure (IV), using dibenzoylmethane **2a** (200.0 mg, 0.892 mmol), 1-azido-3-bromobenzene **3u** (306.9 mg, 1.784 mmol), hexamethyldisilazane (0.375 mL, 1.784 mmol) and N,N-dimethylformamide (0.138 mL, 1.784 mmol). The crude product was then purified by column chromatography (DCM/EtOAc, 50:1) to afford **5au** (314.9 mg, 87% yield) as a white solid, m.p. 134-135 °C; ATR-IR (cm^{-1}): 1579, 1596, 1651, 1719, 2953, 3031. ^{1}H NMR (400 MHz, $CDCl_3$) δ 8.27 (dd, J = 8.0, 1.1 Hz, 2H), 7.64 – 7.58 (m, 2H), 7.57 (dd, J = 8.0, 1.1 Hz, 1H), 7.51 (t, J = 8.0 Hz, 2H), 7.46 – 7.37 (m, 3H), 7.35 (dd, J = 8.1, 1.5 Hz, 2H), 7.25 (t, J = 8.1 Hz, 1H), 7.18 (dd, J = 8.1, 1.1 Hz, 1H).; ^{13}C{^{1}H} NMR (100 MHz, $CDCl_3$): δ 186.6, 143.7, 141.3, 137.1, 136.9, 133.3, 132.7, 130.8, 130.6, 130.2 (2C), 128.8, 128.4 (2C), 125.7, 12.8, 122.9. HRMS (EI) m/z : $[M]^+$ calcd for $C_{21}H_{14}BrN_3O$ 403.0320; found 403.0324

(1-(4-Isopropylphenyl)-5-phenyl-1H-1,2,3-triazol-4-yl)(phenyl)methanone (**5av**)[11]

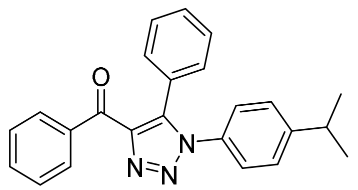

Following the General procedure (IV), using dibenzoylmethane **2a** (200.0 mg, 0.892 mmol), 1-azido-4-isopropylbenzene **3v** (287.5 mg, 1.784 mmol), hexamethyldisilazane (0.375 mL, 1.784 mmol) and N,N-dimethylformamide (0.138 mL, 1.784 mmol). The crude product was then purified by column chromatography (DCM/EtOAc, 50:1) to afford **5av** (218.2 mg, 67% yield) as a brown solid; ^{1}H NMR (400 MHz, $CDCl_3$) δ 8.29 (d, J = 7.5 Hz, 2H), 7.59 (t, J = 7.5 Hz, 1H), 7.50 (t, J = 7.5 Hz, 2H), 7.44 – 7.32 (m, 5H), 7.28 – 7.21 (m, 4H), 2.93 (sep, J = 6.9 Hz, 1H), 1.25 (d, J = 6.9 Hz, 6H); ^{13}C{^{1}H} NMR (100 MHz, $CDCl_3$): δ 186.8, 150.6, 143.6, 141.1, 137.3, 133.6, 133.2, 130.8, 130.3, 129.8, 128.5, 128.3, 127.4, 126.2, 125.1, 33.9, 23.8.

(4-Chlorophenyl)(5-(4-chlorophenyl)-1-phenyl-1H-1,2,3-triazol-4-yl)methanone (5ba)

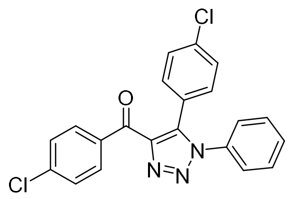

Following the General procedure (IV), using 1,3-bis(4-chlorophenyl)propane-1,3-dione **2b** (146.6 mg, 0.5 mmol), azidobenzene **3a** (119.1 mg, 1.0 mmol), hexamethyldisilazane (0.21 mL, 1.0 mmol) and N,N-dimethylformamide (0.077 mL, 1.0 mmol). The crude product was then purified by column chromatography (DCM) to afford **5ba** (128.6 mg, 65% yield) as a white solid, m.p. 182-183 °C; ATR-IR (cm^{-1}): 1479, 1583, 1655, 1721, 2954, 3099. ^1H NMR (400 MHz, $CDCl_3$) δ 8.31 (d, J = 8.7 Hz, 2H), 7.49 (dd, J = 8.7, 2.2 Hz, 2H), 7.47 – 7.41 (m, 3H), 7.36 (dd, J = 8.7, 2.1 Hz, 2H), 7.33 – 7.27 (m, 4H); $^{13}C\{^1H\}$ NMR (100 MHz, $CDCl_3$): δ 185.2, 143.4, 140.5, 139.9, 136.5, 135.6, 135.4, 132.3, 131.7, 129.9, 129.7, 129.0, 128.8, 125.4, 124.4. HRMS (EI) m/z: $[M]^+$ calcd for $C_{21}H_{13}Cl_2N_3O$ 393.0436; found 393.0439.

(4-Chlorophenyl)(5-(4-chlorophenyl)-1-(4-ethylphenyl)-1H-1,2,3-triazol-4-yl)methanone (5be)

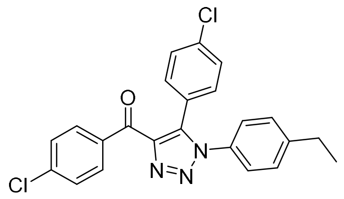

Following the General procedure (IV), using 1,3-bis(4-chlorophenyl)propane-1,3-dione **2b** (146.6 mg, 0.5 mmol), 1-azido-4-ethylbenzene **3e** (147.2 mg, 1.0 mmol), hexamethyldisilazane (0.21 mL, 1.0 mmol) and N,N-dimethylformamide (0.077 mL, 1.0 mmol). The crude product was then purified by column chromatography (DCM) to afford **5be** (99.9 mg, 47% yield) as a white solid, m.p. 152-153 °C; ATR-IR (cm^{-1}): 1479, 1514, 1583, 1660, 2974, 3077, 3010. ^1H NMR (400 MHz, $CDCl_3$) δ 8.30 (d, J = 8.6 Hz, 2H), 7.49 (d, J = 8.6 Hz, 2H), 7.36 (d, J = 8.5 Hz,2H), 7.29 (d, J = 8.5 Hz, 2H), 7.25 (d, J = 8.5 Hz, 2H), 7.21 (d, J = 8.5 Hz, 2H), 2.70 (q, J = 7.6 Hz, 2H), 1.26 (t, J = 7.6 Hz, 3H); $^{13}C\{^1H\}$ NMR (100 MHz, $CDCl_3$): δ 185.3, 146.4, 143.4, 140.4, 139.8, 136.3, 135.5, 133.3, 132.3, 131.7, 129.1, 129.0, 128.7, 125.2, 124.6, 28.6, 15.3. HRMS (EI) m/z: $[M]^+$ calcd for $C_{23}H_{17}Cl_2N_3O$ 421.0749; found 421.0740

(1,5-Bis(4-chlorophenyl)-1H-1,2,3-triazol-4-yl)(4-chlorophenyl)methanone (**5bk**)

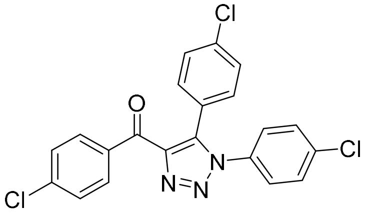

Following the General procedure (IV), using 1,3-bis(4-chlorophenyl)propane-1,3-dione **2b** (146.6 mg, 0.5 mmol), 1-azido-4-chlorobenzene **3k** (153.6 mg, 1.0 mmol), hexamethyldisilazane (0.21 mL, 1.0 mmol) and N,N-dimethylformamide (0.077 mL, 1.0 mmol). The crude product was then purified by column chromatography (DCM) to afford **5bk** (178.3 mg, 83% yield) as a brown solid, m.p. 179-180 °C; ATR-IR (cm^{-1}): 1479, 1583, 1659, 1721, 3099. ^1H NMR (400 MHz, $CDCl_3$) δ 8.29 (d, *J* = 8.6 Hz, 2H), 7.50 (d, *J* = 8.6 Hz, 2H), 7.42 (d, *J* = 8.8 Hz, 2H), 7.39 (d, *J* = 8.6 Hz, 2H), 7.29 (d, *J* = 8.6 Hz, 2H), 7.26 (d, *J* = 8.8 Hz, 2H); $^{13}C\{^1H\}$ NMR (100 MHz, $CDCl_3$): δ 185.0, 143.6, 140.5, 140.0, 136.7, 136.1, 135.3, 134.1, 132.3, 131.6, 130.0, 129.2, 128.8, 126.5, 124.1. HRMS (EI) m/z: $[M]^+$ calcd for $C_{21}H_{12}Cl_3N_3O$ 427.0046; found 427.0052.

(4-Methoxyphenyl)(5-(4-methoxyphenyl)-1-phenyl-1H-1,2,3-triazol-4-yl)methanone (**5ca**)

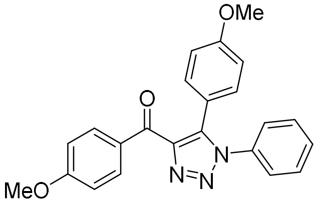

Following the General procedure (IV), using 1,3-bis(4-methoxyphenyl)propane-1,3-dione **2c** (142.2 mg, 0.5 mmol), azidobenzene **3a** (119.1 mg, 1.0 mmol), hexamethyldisilazane (0.21 mL, 1.0 mmol) and N,N-dimethylformamide (0.077 mL, 1.0 mmol). The crude product was then purified by column chromatography (DCM) to afford **5ca** (137.5 mg, 71% yield) as a light yellow solid, m.p. 111-112 °C; ATR-IR (cm^{-1}): 1255, 1490, 1587, 1661, 2957, 3023. ^1H NMR (400 MHz, $CDCl_3$) δ 8.33 (d, *J* = 9.0 Hz, 2H), 7.45 -7.41 (m, 3H), 7.37 - 7.32 (m, 2H), 7.30 - 7.26 (m, 2H), 6.99 (d, *J* = 9.0 Hz, 2H), 6.87 (d, *J* = 9.0 Hz, 2H), 3.90 (s, 3H), 3.81 (s, 3H); $^{13}C\{^1H\}$ NMR (100 MHz, $CDCl_3$): δ 185.3, 163.7, 160.7, 143.7, 140.9, 136.2, 133.3, 131.8, 130.3, 129.4 (2C), 125.4, 118.1, 114.1, 113.6, 55.6, 55.3. HRMS (EI) m/z: $[M]^+$ calcd for $C_{23}H_{19}N_3O_3$ 385.1426; found 385.1431.

(1-(4-Ethylphenyl)-5-(4-methoxyphenyl)-1H-1,2,3-triazol-4-yl)(4-methoxyphenyl)methanone (5ce)

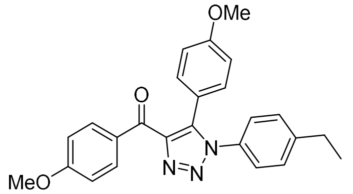

Following the General procedure (IV), using 1,3-bis(4-methoxyphenyl)propane-1,3-dione **2c** (142.2 mg, 0.5 mmol), 1-azido-4-ethylbenzene **3e** (147.2 mg, 1.0 mmol), hexamethyldisilazane (0.21 mL, 1.0 mmol) and N,N-dimethylformamide (0.077 mL, 1.0 mmol). The crude product was then purified by column chromatography (DCM) to afford **5ce** (151.5 mg, 73% yield) as a brown solid, m.p. 112-113 °C; ATR-IR (cm^{-1}): 1248, 1494, 1596, 1647, 2840, 2931, 2962, 3075. ^{1}H NMR (400 MHz, $CDCl_3$) δ 8.33 (d, J = 9.0 Hz, 2H), 7.28 (d, J = 8.9 Hz, 2H), 7.25 – 7.21 (m, 4H), 6.98 (d, J = 9.0 Hz, 2H), 6.87 (d, J = 8.9 Hz, 2H), 3.89 (s, 3H), 3.81 (s, 3H), 2.69 (q, J = 7.6 Hz, 2H), 1.25 (t, J = 7.6 Hz, 3H); $^{13}C\{^{1}H\}$ NMR (100 MHz, $CDCl_3$): δ 185.4, 163.7, 160.6, 145.8, 143.6, 140.8, 133.8, 133.3, 131.8, 130.3, 128.8, 125.2, 118.2, 114.0, 113.6, 55.6, 55.3, 28.6, 15.3. HRMS (EI) m/z: $[M]^+$ calcd for $C_{25}H_{23}N_3O_3$ 413.1739; found 413.1730

(1-(4-Chlorophenyl)-5-(4-methoxyphenyl)-1H-1,2,3-triazol-4-yl)(4-methoxyphenyl)methanone (5ck)

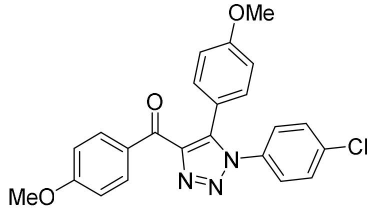

Following the General procedure (IV), using 1,3-bis(4-methoxyphenyl)propane-1,3-dione **2c** (142.2 mg, 0.5 mmol), 1-azido-4-chlorobenzene **3k** (153.6 mg, 1.0 mmol), hexamethyldisilazane (0.21 mL, 1.0 mmol) and N,N-dimethylformamide (0.077 mL, 1.0 mmol). The crude product was then purified by column chromatography (DCM) to afford **5ck** (174.2 mg, 83% yield) as a white solid, m.p. 176-177 °C; ATR-IR (cm^{-1}): 1249, 1491, 1596, 1636, 2841, 2926, 3000. ^{1}H NMR (400 MHz, $CDCl_3$) δ 8.32 (d, J = 9.0 Hz, 2H), 7.40 (d, J = 8.9 Hz, 2H), 7.28 (d, J = 8.8 Hz, 2H), 7.27 (d, J = 8.8 Hz, 2H), 6.98 (d, J = 9.0 Hz. 2H), 6.89 (d, J = 8.9 Hz, 2H), 3.90 (s, 3H), 3.82 (s, 3H); ^{13}C NMR (100 MHz, $CDCl_3$): δ 185.1, 163.8, 160.8, 143.8, 140.9, 135.5, 134.7, 133.3, 131.8, 130.2, 129.7, 126.5, 117.7, 114.2, 113.7, 55.6, 55.4. HRMS (EI) m/z: $[M]^+$ calcd for $C_{23}H_{18}ClN_3O_3$ 419.1037; found 419.1029

(1-Phenyl-5-(*m*-tolyl)-1H-1,2,3-triazol-4-yl)(*m*-tolyl)methanone (**5da**)

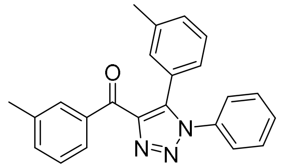

Following the General procedure (IV), using 1,3-di-m-tolylpropane-1,3-dione **2d** (126.2 mg, 0.5 mmol), azidobenzene **3a** (119.1 mg, 1.0 mmol), hexamethyldisilazane (0.21 mL, 1.0 mmol) and N,N-dimethylformamide (0.077 mL, 1.0 mmol). The crude product was then purified by column chromatography (DCM) to afford **5da** (117.6 mg, 67% yield) as a brown solid, m.p. 107-109 °C; ATR-IR (cm^{-1}): 1259, 1499, 1597, 1644, 2853, 2922, 3064. ^{1}H NMR (400 MHz, $CDCl_3$) δ 8.06 (d, J = 7.0 Hz, 1H), 8.03 (s, 1H), 7.45 – 7.37 (m, 5H), 7.37 – 7.31 (m, 2H), 7.25 – 7.14 (m, 3H), 7.08 (d, J = 7.0 Hz, 1H), 2.42 (s, 3H), 2.29 (s, 3H); $^{13}C\{^1H\}$ NMR (100 MHz, $CDCl_3$): δ 187.1, 143.8, 141.3, 138.3, 138.0, 137.3, 136.1, 134.0, 131.2, 130.8, 130.7, 129.5, 129.4, 128.4, 128.2, 128.0, 127.3, 126.0, 125.3, 21.5, 21.4. HRMS (EI) m/z: $[M]^+$ calcd for $C_{23}H_{19}N_3O$ 353.1528; found 353.1533

(1-(4-Ethylphenyl)-5-(m-tolyl)-1H-1,2,3-triazol-4-yl)(m-tolyl)methanone (**5de**)

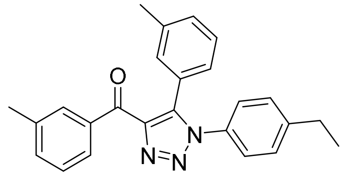

Following the General procedure (IV), using 1,3-di-m-tolylpropane-1,3-dione **2d** (126.2 mg, 0.5 mmol), 1-azido-4-ethylbenzene **3e** (147.2 mg, 1.0 mmol), hexamethyldisilazane (0.21 mL, 1.0 mmol) and N,N-dimethylformamide (0.077 mL, 1.0 mmol). The crude product was then purified by column chromatography (DCM) to afford **5de** (99.6 mg, 53% yield) as a brown solid, m.p. 136-137 °C; ATR-IR (cm^{-1}): 1258, 1511, 1599, 1651, 2856, 2924, 2977, 3023. ^{1}H NMR (400 MHz, $CDCl_3$) δ 8.06 (d, J = 6.7 Hz, 1H), 8.03 (s, 1H), 7.38 (d, J = 7.4 Hz, 2H), 7.25 – 7.17 (m, 6H), 7.15 (s, 1H), 7.08 (d, J = 6.7 Hz, 1H), 2.68 (q, J = 7.6 Hz, 2H), 2.42 (s, 3H), 2.29 (s, 3H), 1.24 (t, J = 7.6 Hz, 3H); $^{13}C\{^1H\}$ NMR (100 MHz, $CDCl_3$): δ 187.2, 145.9, 143.7, 141.2, 138.3, 138.0, 137.4, 133.9, 133.7, 131.2, 130.8, 130.6, 128.7, 128.4, 128.2, 128.1, 127.3, 126.2, 125.2, 28.6, 21.5, 21.4, 15.3. HRMS (EI) m/z: $[M]^+$ calcd for $C_{25}H_{23}N_3O$ 381.1841; found 381.1845

(1-(4-Chlorophenyl)-5-(m-tolyl)-1H-1,2,3-triazol-4-yl)(m-tolyl)methanone (**5dk**)

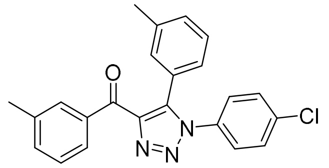

Following the General procedure (IV), using 1,3-di-m-tolylpropane-1,3-dione **2d** (126.2 mg, 0.5 mmol), 1-azido-4-chlorobenzene **3k** (153.6 mg, 1.0 mmol), hexamethyldisilazane (0.21 mL, 1.0 mmol) and N,N-dimethylformamide (0.077 mL, 1.0 mmol). The crude product was then purified by column chromatography (DCM) to afford **5dk** (158.0 mg, 82% yield) as a brown solid, m.p. 158-159 °C; ATR-IR (cm^{-1}): 1258, 1493, 1599, 1652, 2855, 2924, 3065. ^{1}H NMR (400 MHz, $CDCl_3$) δ 8.05 (d, J = 7.2 Hz, 1H), 8.02 (s, 1H), 7.44 – 7.35 (m, 4H), 7.29 (d, J = 8.8 Hz, 2H), 7.25 – 7.20 (m, 2H), 7.15 (s, 1H), 7.06 (d, J = 7.2 Hz, 1H), 2.42 (s, 3H), 2.32 (s, 3H); $^{13}C\{^{1}H\}$ NMR (100 MHz, $CDCl_3$): δ 187.0, 143.9, 141.2, 138.6, 138.1, 137.2, 135.6, 134.6, 134.1, 131.2, 130.9, 130.7, 129.7, 128.6, 128.3, 128.0, 127.2, 126.4, 125.8, 21.6, 21.5. HRMS (EI) m/z: $[M]^+$ calcd for $C_{23}H_{18}ClN_3O$ 387.1138; found 387.1132

1-(1,5-Diphenyl-1H-1,2,3-triazol-4-yl)ethan-1-one (**5ek**)[13]

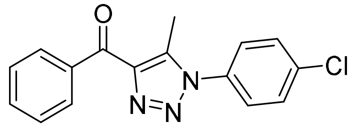

Following the General procedure (IV), using 1-phenylbutane-1,3-dione **2e** (81.1 mg, 0.5 mmol), 1-azido-4-chlorobenzene **3k** (153.6 mg, 1.0 mmol), hexamethyldisilazane (0.21 mL, 1.0 mmol) and N,N-dimethylformamide (0.077 mL, 1.0 mmol). The crude product was then purified by column chromatography (DCM) to afford **5ek** (88.3 mg, 59% yield) as a brown solid; ^{1}H NMR (400 MHz, $CDCl_3$) δ 8.39 – 8.35 (m, 2H), 7.65 – 7.56 (m, 3H), 7.56 – 7.44 (m, 4H), 2.68 (s, 3H); $^{13}C\{^{1}H\}$ NMR (100 MHz, $CDCl_3$): δ 187.6, 143.7, 140.0, 137.4, 136.4, 134.0, 133.1, 130.7, 130.1, 128.4, 126.7, 10.7.

Ethyl 1-(4-chlorophenyl)-5-phenyl-1H-1,2,3-triazole-4-carboxylate (**5fk**)

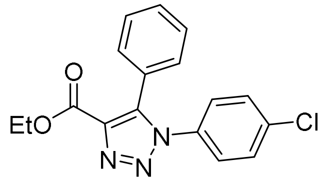

Following the General procedure (IV), using ethyl 3-oxo-3-phenylpropanoate (purity 90%) **2f** (0.096 mL, 0.5 mmol), 1-azido-4-chlorobenzene **3k** (153.6 mg, 1.0 mmol), hexamethyldisilazane (0.21 mL, 1.0 mmol) and N,N-dimethylformamide (0.077 mL,

1.0 mmol). The crude product was then purified by column chromatography (Hexane/EtOAc, 2:1) to afford **5fk** (158.4 mg, 98% yield) as a brown solid, m.p. 91-92 °C; ATR-IR (cm^{-1}): 1229, 1223, 1493, 1566, 1717, 2981, 3061. ^{1}H NMR (400 MHz, $CDCl_3$) δ 7.48 – 7.39 (m, 3H), 7.35 (d, *J* = 8.4 Hz, 2H), 7.28 (d, *J* = 7.6 Hz, 2H), 7.22 (d, *J* = 8.4 Hz, 2H), 4.37 (q, *J* = 7.1 Hz, 2H), 1.32 (t, *J* = 7.1 Hz, 3H); ^{13}C{^{1}H} NMR (100 MHz, $CDCl_3$): δ 160.8, 140.8, 137.1, 135.5, 134.3, 130.2, 130.1, 129.6, 128.5, 126.3, 125.5, 61.3, 14.1. HRMS (EI) m/z: $[M]^+$ calcd for $C_{17}H_{14}ClN_3O_2$ 327.0775; found 327.0781

1-(4-Chlorophenyl)-*N,N*-dimethyl-5-phenyl-1H-1,2,3-triazole-4-carboxamide (5gk)

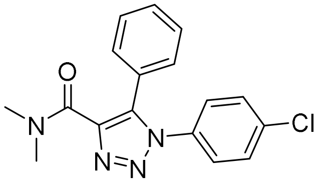

Following the General procedure(IV), using *N,N*-dimethyl-3-oxo-3-phenylpropanamide **2g** (95.6 mg, 0.50 mmol), 1-azido-4-chlorobenzene **3k** (153.6 mg, 1.00 mmol), hexamethyldisilazane (0.21 mL, 1.00 mmol) and N,N-dimethylformamide (0.077 mL, 1.00 mmol). The crude product was then purified by column chromatography (Hexane/Acetone 3:1) to afford **5gk** (127.1mg, 78% yield) as a white solid, m.p. 194-195 °C; ATR-IR (cm^{-1}): 1402, 1496, 1634, 2928; ^{1}H NMR (400 MHz, $CDCl_3$) δ 7.41 – 7.34 (m, 5H), 7.31 (dt, *J* = 8.4, 2.1 Hz, 2H), 7.27 – 7.23 (m, 2H), 3.17 (s, 3H), 3.09 (s, 3H); ^{13}C{^{1}H} NMR (100 MHz, $CDCl_3$) δ 162.5, 141.0, 137.7, 135.5, 134.8, 129.9 (2C), 129.7, 128.9, 126.4, 125.9, 39.0, 35.6: HRMS (EI) m/z : $[M]^+$ calcd. for $C_{17}H_{15}ClN_4O$ 326.0934; found 326.0932

1-(4-(4-Phenyl-1H-1,2,3-triazol-1-yl)phenyl)ethan-1-one (6)[14]

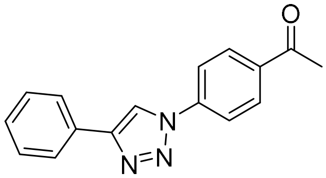

1-(4-Azidophenyl)ethan-1-one (200.0 mg, 1.24 mmol), and phenyl acetylene (0.14 ml, 1.24 mmol), were suspended in *tert*-BuOH/H_2O (4:1, 20 mL). To this mixture was added $CuSO_4·5H_2O$ (15.0 mg, 0.06 mmol) and (+)-sodium ascorbate (75.0 mg, 0.37 mmol). The mixture was stirred at 100 °C (oil bath) for 24 h. The reaction was monitored by TLC which indicated complete conversion. The resulting solution was

concentrated under reduced pressure. The residue was dissolved in 40 mL of brine solution and then extracted with ethyl acetate (3 x 30 mL). The combined organic layers were washed with 5 % aq. NH_4OH (2 x 25 mL), dried over $MgSO_4$, filtered and the solvent was removed under vacuum. The crude product was recrystallized from ethanol to give pure product **6** (248.6 mg, 76% yield) as a yellow solid; ^{1}H NMR (400 MHz, $(CD_3)_2SO$) δ 9.45 (s, 1H), 8.20 (d, J = 8.8 Hz, 2H), 8.13 (d, J = 8.8 Hz, 2H), 7.95 (d, J = 7.1 Hz, 2H), 7.51 (t, J = 7.6 Hz, 2H), 7.40 (t, J = 7.4 Hz, 1H), 2.65 (s, 3H).

(4-(4-Phenyl-1H-1,2,3-triazol-1-yl)phenyl)(1-phenyl-1H-1,2,3-triazol-4-yl)methanone (7)

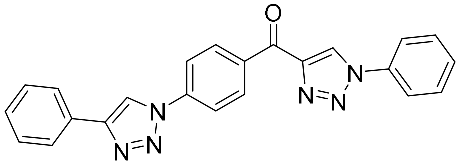

Using 1-(4-(4-Phenyl-1H-1,2,3-triazol-1-yl)phenyl)ethan-1-one (100.0 mg, 0.38 mmol), phenyl azide **3a** (90.5 mg, 0.76 mmol), hexamethyldisilazane (0.32 mL, 1.52 mmol) and N,N-dimethylformamide (0.24 mL, 3.04 mmol), 4-methylbenzenesulfonate (10.1 mg, 0.04 mmol) then stirred at 120 °C (oil bath) under nitrogen atmosphere for 12 h. After the reaction completed, the crude products were purified by flash column chromatography (DCM) to afford **7** (120.6 mg, 81% yield) as a white solid; m.p. 244-246°C; ATR-IR (cm^{-1}):1266, 1407, 1510, 1600, 1634, 3137; ^{1}H NMR (400 MHz, $(CD_3)_2SO$) δ 9.68 (s, 1H), 9.49 (s, 1H), 8.56 (d, J = 8.7 Hz, 2H), 8.23 (d, J = 8.7 Hz, 2H), 8.06 (d, J = 7.6 Hz, 2H), 7.98 (d, J = 7.2 Hz, 2H), 7.66 (t, J = 7.8 Hz, 2H), 7.61 – 7.49 (m, 3H), 7.41 (t, J = 7.4 Hz, 1H); ^{13}C{^{1}H} NMR (100 MHz, $(CD_3)_2SO$) δ 183.8, 147.6, 147.1, 139.7, 136.0, 132.0, 130.0, 129.5, 129.0, 128.4, 128.2, 125.4, 120.7, 119.8, 119.6. HRMS (EI) m/z: $[M]^+$ calcd for $C_{23}H_{16}N_6O$ 392.1386; found 392.1390.

***Tert*-butyl (2-acetylphenyl)carbamate (8)[15]**

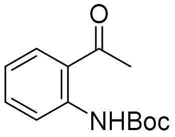

To a solution of *o*-aminoacetophenone (5.0 mmol, 1.0 equiv) in EtOH (10 mL) was added di-*tert*-butyl dicarbonate (20.0 mmol, 4.0 equiv). The reaction mixture was stirred at 50 °C (oil bath) under nitrogen atmosphere for 48 h, and concentrated in vacuo upon completion. The residue was subjected to flash silica gel column chromatography to afford *tert*-butyl(2-acetylphenyl)carbamate **8** (850.2 mg, 73%) as a white solid [by the elution of mixed petroleum ether (PE)/ethyl acetate (EA) (v/v =75:1−50:1). ^{1}H

NMR (400 MHz, $CDCl_3$) δ 10.94 (s, 1H), 8.47 (d, *J* = 8.5 Hz, 1H), 7.86 (d, *J* = 8.0 Hz, 1H), 7.51 (t, *J* = 7.9 Hz, 1H), 7.03 (t, *J* = 7.6 Hz, 1H), 2.65 (s, 3H), 1.52 (s, 9H).

(2-Aminophenyl)(1-(3-isopropylphenyl)-1H-1,2,3-triazol-4-yl)methanone (11)[16]

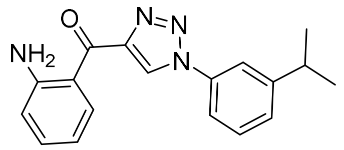

Following the General procedure (I), using *tert*-butyl(2-acetylphenyl)carbamate **8** (80.0 mg, 0.84 mmol), hexamethyldisilazane (0.29 mL, 1.36 mmol) and N,N-dimethylformamide (0.11 mL, 1.36 mmol), 4-methylbenzenesulfonate (7.5 mg, 0.03 mmol), 1-azido-3-isopropylbenzene **9** (163.0 mg, 1.02 mmol) then stirred at 100 °C (oil bath) under nitrogen atmosphere for 12 h. After the reaction was completed, the crude products were purified by flash column chromatography (DCM) to afford the desired *tert*-butyl(2-(1-(3-isopropylphenyl)-1H-1,2,3-triazole-4-carbonyl)phenyl)carbamate **10**. Subsequently, *tert*-butyl(2-(1-(3-isopropylphenyl)-1H-1,2,3-triazole-4-carbonyl)phenyl)carbamate **10** (73.8 mg, 0.18 mmol) in DCM/H_2O (9:1, 1.8 mL; 0.1M) was added 2,2,2-trifluoroacetic acid (0.21 mL, 2.72 mmol) to the reaction mixture and stirred at room temperature under nitrogen atmosphere for 9 h. After the reaction was completed, the crude was neutralized with saturated $NaHCO_{3(aq)}$ and extracted with DCM for three times and combined organic layer was washed with brine, dried over $MgSO_4$, filtered and concentrated. The crude products were purified by flash column chromatography (DCM) to afford the desired products **11** (49.2 mg, 61% yield of two steps) as a yellow solid; ^{1}H NMR (400 MHz, $(CD_3)_2SO$) δ 9.42 (s, 1H), 8.38 (d, *J* = 6.9 Hz, 1H), 7.89 (s, 1H), 7.82 (d, *J* = 8.7 Hz, 1H), 7.53 (t, *J* = 7.9 Hz, 1H), 7.42 (d, *J* = 7.8 Hz, 1H), 7.38 – 7.26 (m, 3H), 6.85 (d, *J* = 8.4 Hz, 1H), 6.60 (t, *J* = 7.6 Hz, 1H), 3.08 – 2.96 (m, 1H), 1.27 (d, *J* = 6.9 Hz, 6H); ^{13}C{^{1}H} NMR (100 MHz, $(CD_3)_2SO$) δ 186.2, 152.4, 150.7, 148.5, 136.3, 134.7, 133.8, 129.9, 127.3, 127.1, 118.6, 118.1, 116.8, 116.3, 114.5, 33.5, 23.7.

(*E*)-3-(Dimethylamino)-1-phenylprop-2-en-1-one (12)[17]

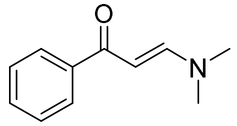

Using acetophenone **1a** (100.0 mg, 0.83 mmol), hexamethyldisilazane (0.70 mL, 3.33 mmol) and N,N-dimethylformamide (0.26 mL, 3.33 mmol), 4-methylbenzenesulfonate (20.9 mg, 0.08 mmol) and then stirred at 120 °C (oil bath) under nitrogen atmosphere for 8 hours. The crude product was then purified by column chromatography

(DCM~DCM/ EA, 1:1) to afford **12** (135.1 mg, 93% yield) as a brown solid; ^{1}H NMR (400 MHz, $CDCl_3$) δ 7.89 (d, *J* = 6.6 Hz, 2H), 7.80 (d, *J* = 12.4 Hz, 1H), 7.53-7.35 (m, 3H), 5.71 (d, *J* = 12.4 Hz, 1H), 3.14 (s, 3H), 2.92 (s, 3H); $^{13}C\{^{1}H\}$ NMR (100 MHz, $CDCl_3$) δ 188.8, 154.3, 140.6, 131.0, 128.2, 127.6, 92.2, 45.2, 37.4.

(*E*)-3-(Diethylamino)-1-phenylprop-2-en-1-one (13)[18]

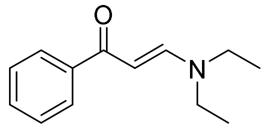

Using acetophenone **1a** (100.0 mg, 0.83 mmol), hexamethyldisilazane (0.70 mL, 3.33 mmol) and N,N-diethylformamide (0.40 mL, 3.33 mmol), 4-methylbenzenesulfonate (20.9 mg, 0.08 mmol) and then stirred at 120 °C (oil bath) under nitrogen atmosphere for 12 hours. The crude product was then purified by column chromatography (DCM~DCM/ EA, 1:1) to afford **13** (168.7 mg, 71% yield) as a yellow liquid; ^{1}H NMR (400 MHz, $CDCl_3$) δ 7.84 (d, *J* = 6.6 Hz, 2H), 7.75 (d, *J* = 12.5 Hz, 1H), 7.48 – 7.28 (m, 3H), 5.71 (d, *J* = 12.5 Hz, 1H), 3.39 – 3.08 (m, 4H), 1.14 (t, *J* = 7.1 Hz, 6H); $^{13}C\{^{1}H\}$ NMR (100 MHz, $CDCl_3$) δ 188.5, 152.2, 140.6, 130.6, 127.9, 127.3, 91.4, 50.4, 42.7, 14.7, 11.4.

(*Z*)-1-Phenyl-3-(phenylamino)prop-2-en-1-one (14)[19]

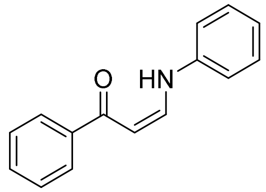

Using acetophenone **1a** (100.0 mg, 0.83 mmol), hexamethyldisilazane (0.70 mL, 3.33 mmol) and N,N-dimethylformamide (0.40 mL, 3.33 mmol), 4-methylbenzenesulfonate (20.9 mg, 0.08 mmol) and then stirred at 120 °C (oil bath) under nitrogen atmosphere for 8 hours. Thereafter, the reaction mixture was concentrated under reduced pressure. To a solution of reaction mixture in DMF (0.26 mL) was added aniline (91 μL, 0.99 mmol) and 4-methylbenzenesulfonate (188.1 mg, 0.75 mmol) and stirred at 120 °C (oil bath) under nitrogen atmosphere for another 1 hour. After the reaction was completed, the crude products were purified by flash column chromatography (hexane/ EtOAc, 8:1) to afford **14** (114.5 mg, 63% yield) as a yellow solid; ^{1}H NMR (400 MHz, $CDCl_3$) δ 12.16 (d, *J* = 10.6 Hz, 1H), 7.95 (d, *J* = 7.1 Hz, 2H), 7.58 – 7.41 (m, 4H), 7.35 (t, *J* = 7.9 Hz, 2H), 7.17 – 7.02 (m, 3H), 6.04 (d, *J* = 7.9 Hz, 1H); $^{13}C\{^{1}H\}$ NMR (100 MHz, $CDCl_3$) δ 191.0, 145.0, 140.2, 139.2, 131.6, 129.8, 128.5, 127.3, 123.7, 116.3, 93.7.

(Z)-3-Amino-1,3-diphenylprop-2-en-1-one (15)[20]

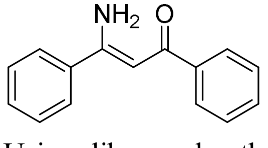

Using dibenzoylmethane **2a** (200.0 mg, 0.89 mmol), hexamethyldisilazane (0.74 mL, 3.56 mmol) and N,N-dimethylformamide (0.56 mL, 7.13 mmol) and *p*-toluenesulfonic acid monohydrate (84.6 mg, 0.45 mmol), then stirred at 100 °C (oil bath) under nitrogen atmosphere for 12 h. After the reaction completed, the crude products were purified by flash column chromatography (DCM) to afford **15** (155.2 mg, 78% yield) as a yellow oil; ^{1}H NMR (400 MHz, $CDCl_3$) δ 10.41 (s), 7.94 (dd, J = 8.0, 1.6 Hz), 7.63 (dd, J = 7.9, 1.7 Hz), 7.56 – 7.37 (m), 6.14 (s), 5.46 (s); ^{13}C{^{1}H} NMR (100 MHz, $CDCl_3$) δ 189.9, 163.1, 140.3, 137.3, 130.9, 130.6, 128.9, 128.2, 127.1, 126.3, 91.6.

(E)-3-(Dimethylamino)-1-phenylprop-2-en-1-one-3-d (Deuterated enaminone)

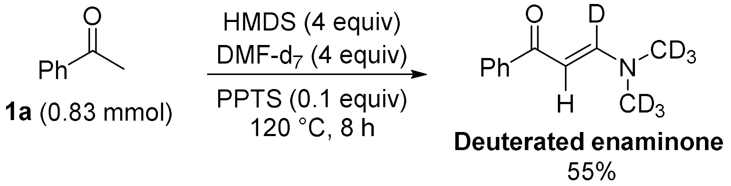

Following the aforementioned protocol (enaminone **12**), using acetophenone **1a** (100.0 mg, 0.83 mmol), hexamethyldisilazane (0.70 mL, 3.33 mmol) and N,N-dimethylformamide-d_7 (DMF-d_7, 0.27 mL, 3.33 mmol), 4-methylbenzenesulfonate (20.9 mg, 0.08 mmol) and then stirred at 120 °C (oil bath) under nitrogen atmosphere for 8 hours. The crude product was then purified by column chromatography (DCM~DCM/ EA, 1:1) to afford deuterated enaminone (83.1 mg, 55% yield) as a yellow solid; m.p. 89-90 °C; ATR-IR (cm^{-1}) v 3052, 3023, 2920, 2067, 1622, 1594, 1505, 1431, 1213, 1116; ^{1}H NMR (400 MHz, $CDCl_3$) δ 7.88 (d, J = 6.7 Hz, 2H), 7.50 – 7.34 (m, 3H), 5.69 (s, 1H); ^{13}C{1H} NMR (100 MHz, $CDCl_3$) δ 188.8, 140.7, 131.0, 128.2, 127.6, 92.1 ; HRMS (EI) m/z : $[M]^+$ calcd. for $C_{11}H_6D_7NO$: 182.1437, found: 182.1445

Reference:

1. (a) Li, Y.-S.; Liang, C.-F. Cerium(III) Triflate–Catalyzed Cycloaddition Reaction in Aqueous Conditions to Substituted Naphthotriazolediones. *J. Chin. Chem. Soc.* **2022**, *69*, 849. (b) Chen, L.-J.; Kuo, C.-J.; Liang, C.-F. Synthesis of Aryl Amides from Acyl-Bunte Salts and Aryl Azides. *J. Org. Chem.* **2023**, *88*, 10501.
2. Gao, Y.; Zhou, D.; Ma, Y., α-Csp3-H Methylenation of Diketones to Synthesize Methylene-Bridged Bis-1,3-Dicarbonyl Compounds and Polysubstituted Pyridines

Using the DMSO/Selectfluor System. *ChemistrySelect* **2018,** *3*, 9374.

3. Wan, J-P.; Cao, S.; Liu, Y. Base-Promoted Synthesis of *N*-Substituted 1,2,3-Triazoles via Enaminone-Azide Cycloaddition Involving Regitz Diazo Transfer. *Org. Lett.* **2016**, *18*, 6034.
4. Cui, X.; Zhang, X.; Wang, Wei.; Zhong, X.; Tan, Y.; Wang, Yan.; Zhang, J.; Li, Youbin.; Wang, X. Regitz Diazo Transfer Reaction for the Synthesis of 1,4,5-Trisubstituted 1,2,3-Triazoles and Subsequent Regiospecific Construction of 1,4-Disubstituted 1,2,3-Triazoles via C−C Bond Cleavage**.** *J. Org. Chem*. **2021**, *86*, 4071.
5. Thomas, J.; Goyvaerts, V.; Liekens, S.; Dehaen, W., Metal-Free Route for the Synthesis of 4-Acyl-1,2,3-Triazoles from Readily Available Building Blocks. *Chem. Eur. J.* **2016,** *22*, 9966.
6. Huang,L.; Zheng, L.; Zhou, h.; Chen, Y. Copper-catalyzed Multipe Oxidation and Cycloaddition of Aryl-Aleyl Ketones (Alcohols) for Synthesis of 4-acyl-and Diketo-1,2,3-Triazoles. *Chem. Commun*. **2022**, *58*, 3342.
7. Kumpan, K.; Nathubhai, A.; Zhang, C.; Wood, P. J.; Lloyd, M. D.; Thompson, A. S.; Haikarainen, T.; Lehtiö, L.; Threadgill, M. D., Structure-based design, synthesis and evaluation in vitro of arylnaphthyridinones, arylpyridopyrimidinones and their tetrahydro derivatives as inhibitors of the tankyrases. *Bioorg. Med. Chem.* **2015,** *23*, 3013.
8. Hu, A.; Lin, W., Ru-Catalyzed Asymmetric Hydrogenation of α-Phthalimide Ketones and 1,3-Diaryl Diketones Using 4,4‘-Substituted BINAPs. *Org. Lett.* **2005,** *7*, 455.
9. Ge, L.-S.; Wang, Z.-L.; An, X.-L.; Luo, X.; Deng, W.-P., Direct synthesis of polysubstituted 2-aminothiophenes by Cu(ii)-catalyzed addition/oxidative cyclization of alkynoates with thioamides. *Org. Biomol. Chem.* **2014,** *12*, 8473.
10. Du, H.; Rodriguez, J.; Bugaut, X.; Constantieux, T., Organocatalytic Enantio- and Diastereoselective Conjugate Addition to Nitroolefins: When β-Ketoamides Surpass β-Ketoesters. *Chem. Eur. J.* **2014,** *20*, 8458.
11. Zhang, X.; Cui, X.; Wang, W.; Zeng, T.; Wang, Y.; Tan, Y.; Liu, D.; Wang, X.; Li, Y., Base-Promoted Regiospecific Synthesis of Fully Substituted 1,2,3-Triazoles and 1,5-Disubstituted 1,2,3-Triazoles. *Asian J. Org. Chem.* **2020,** *9*, 2176.
12. Valizadeh, H.; Amiri, M.; Khalili, E., Task-specific nitrite and azide ionic liquids for the efficient one-pot synthesis of 1,2,3-triazoles from the aniline derivatives. *Mol. Diversity* **2012,** *16*, 319.
13. De Nino, A.; Algieri, V.; Tallarida, M. A.; Costanzo, P.; Pedrón, M.; Tejero, T.; Merino, P.; Maiuolo, L., Regioselective Synthesis of 1,4,5-Trisubstituted-1,2,3-Triazoles from Aryl Azides and Enaminones. *Eur. J. Org. Chem.* **2019,** *2019*, 5725.
14. Jabeen, F.; Shehzadi, S. A.; Fatmi, M. Q.; Shaheen, S.; Iqbal, L.; Afza, N.; Panda,

S. S.; Ansari, F. L. Synthesis, *in Vitro* and Computational Studies of 1,4-Disubstituted 1,2,3-Triazoles as Potential α-Glucosidase Inhibitors. *Bioorg. Med. Chem. Lett.* **2016**, *26*, 1029.

15. Lin, Y.; Wan, J.-P.; Liu, Y. Cascade *in Situ* Iodination, Chromone Annulation, and Cyanation for Site-Selective Synthesis of 2-Cyanochromones. *J. Org. Chem.* **2023**, *88*, 4017.
16. Xu, S.; Zhuang, X.; Pan, X.; Zhang, Z.; Duan, L.; Liu, Y.; Zhang, L.; Ren, X.; Ding, K. 1-Phenyl-4-benzoyl-1*H*-1,2,3-triazoles as Orally Bioavailable Transcriptional Function Suppressors of Estrogen-Related Receptor α. *J. Med. Chem.* **2013***, 56,* 4631.
17. Liang, G.; Rong, J.; Sun, W.; Chen, G.; Jiang, Y.; Loh, T.-P. Synthesis of Polyaromatic Rings: Rh(III)-Catalyzed [5+1] Annulation of Enaminones with Vinyl Esters through C–H Bond Functionalized. *Org. Lett.* **2018**, *20*, 7326.
18. Zhang, J.; Zhou, P.; Yin, A.; Zhang, S.; Liu, W. Synthetic Route to Enaminones via Metal-Free Four-Component Sequential Reactions of Aryl Olefins with $CHCl_3$, Et_3N, and TBHP. *J. Org. Chem*. **2021**, *86*, 8980.
19. Zhou, S.; Liu, D.-Y.; Wang, S.; Tian, J.-S.; Loh, T.-P. An Efficient Method for the Synthesis of 2-Pyridones via C–H Bond Functionalization. *Chem. Commun*. **2020**, *56*, 15020.
20. Wan, C.; Pang, J.-Y., Jiang, W.; Zhang, X.-W.; Hu X.-G. Copper-Catalyzed Reductive-Cleavage of Isoxazoles: Synthesis of Fluoroalkylated Enaminones and Application for the Preparation of Celecoxib, Deracoxib, and Mavacoxib. *J. Org. Chem*. **2021**, *86*, 4557.

Parameter	Value
Origin	Varian
Spectrometer	vnmrs
Solvent	"$CDCl_3$"
Temperature	25.0
Pulse Sequence	s2pul
Experiment	1D
Number of Scans	8
Receiver Gain	52
Relaxation Delay	1.0000
Pulse Width	0.0000
Acquisition Time	2.5559
Modification Date	2023-03-03T17:18:00
Spectrometer Frequency	399.76
Spectral Width	6410.3
Lowest Frequency	-799.4
Nucleus	1H

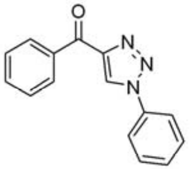

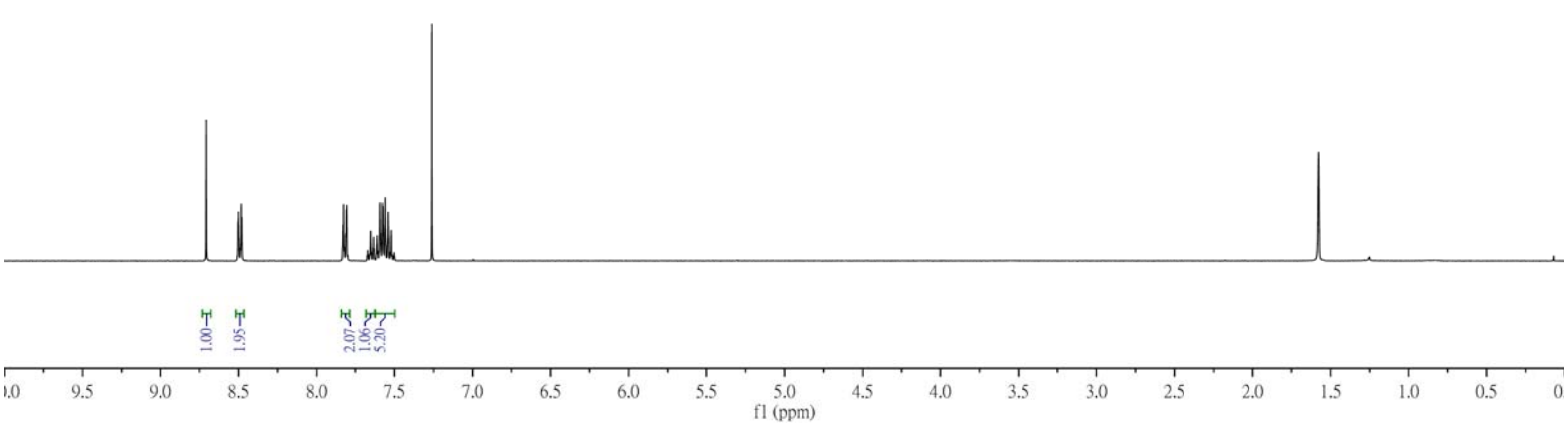

4aa ^{1}H NMR spectrum (400 MHz in $CDCl_3$)

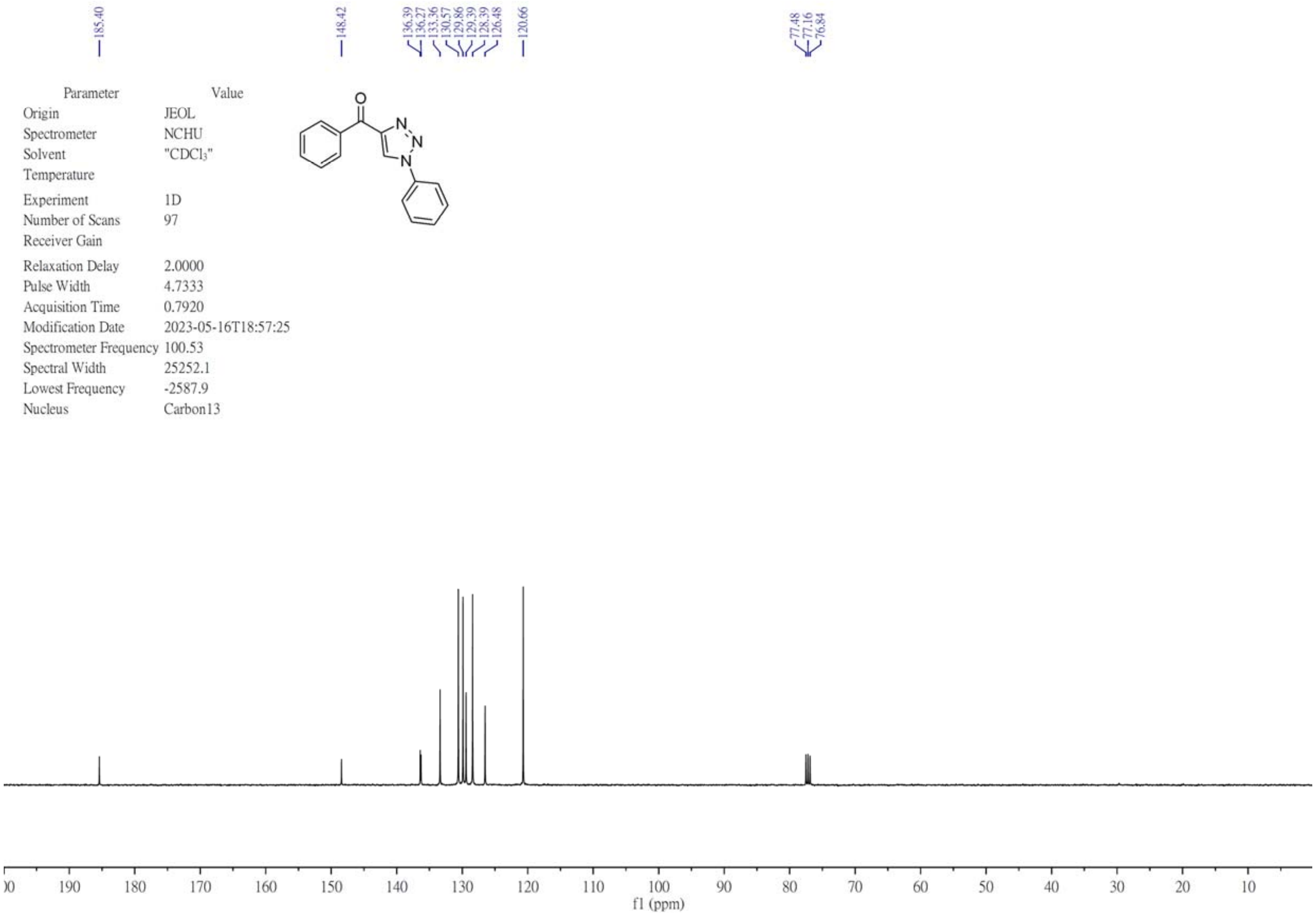

4aa $^{13}C\{^{1}H\}$ NMR spectrum (100 MHz in $CDCl_3$)

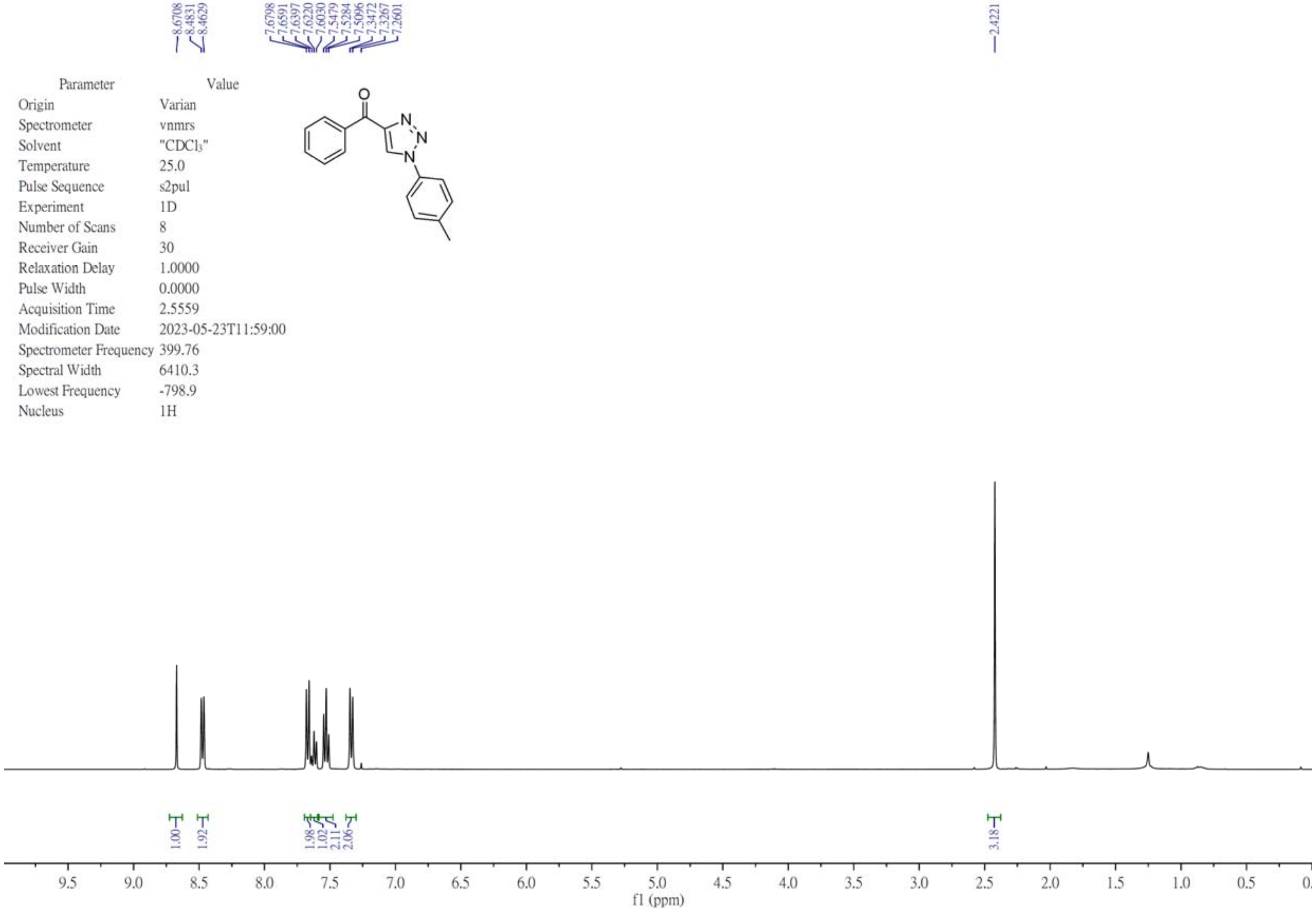

4ab ^{1}H NMR spectrum (400 MHz in $CDCl_3$)

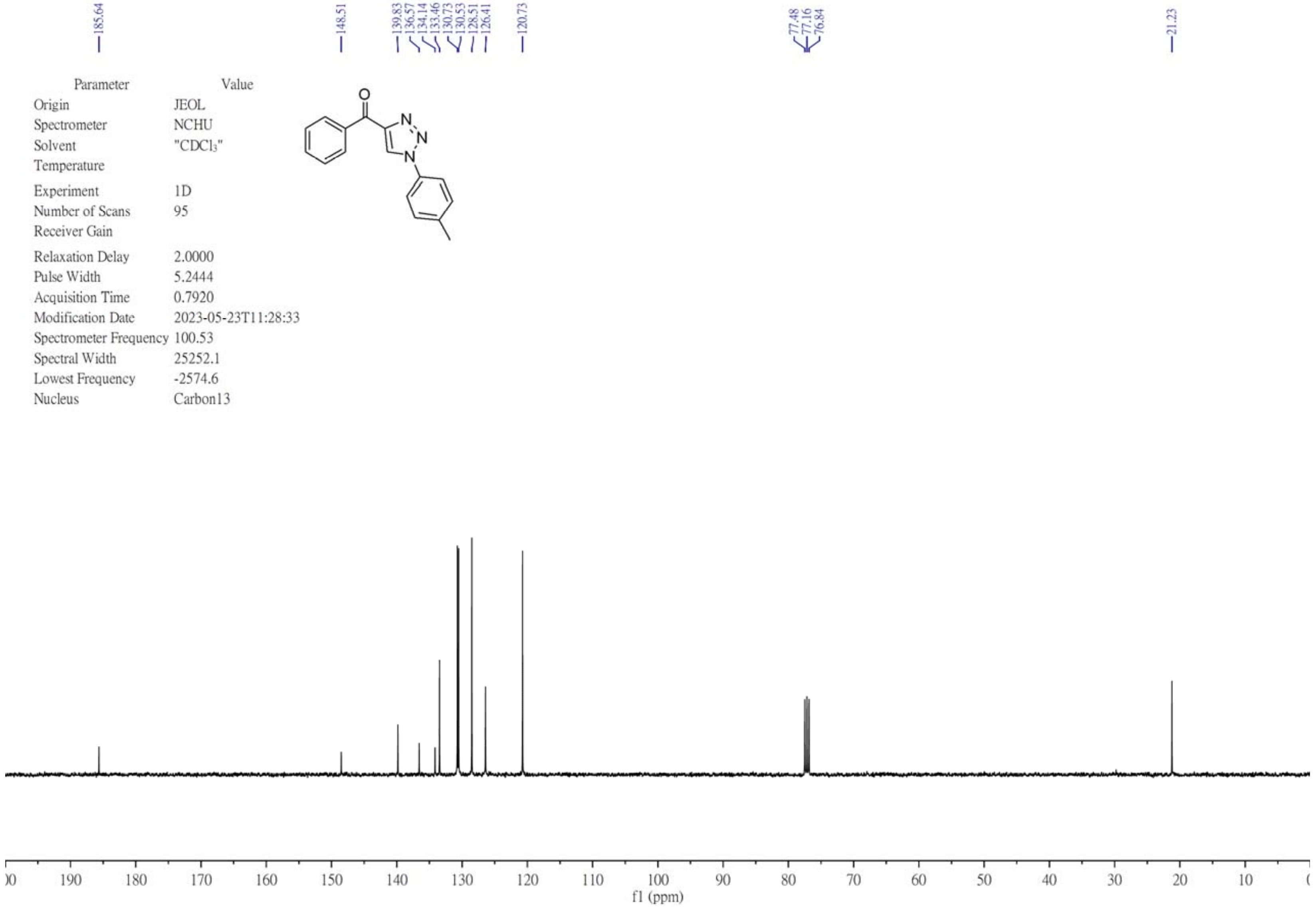

4ab $^{13}C\{^{1}H\}$ NMR spectrum (100 MHz in $CDCl_3$)

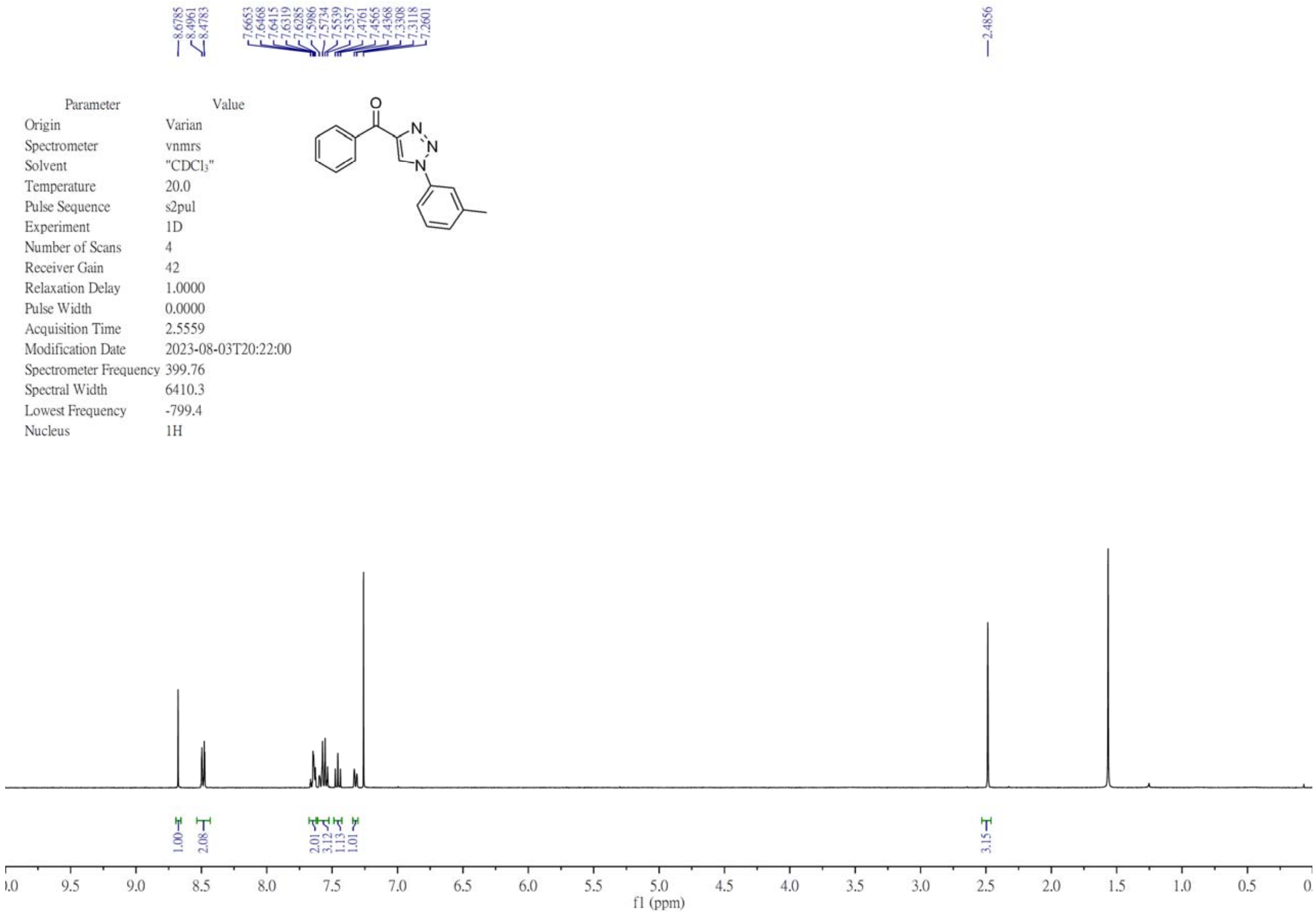

4ac ^{1}H NMR spectrum (400 MHz in $CDCl_3$)

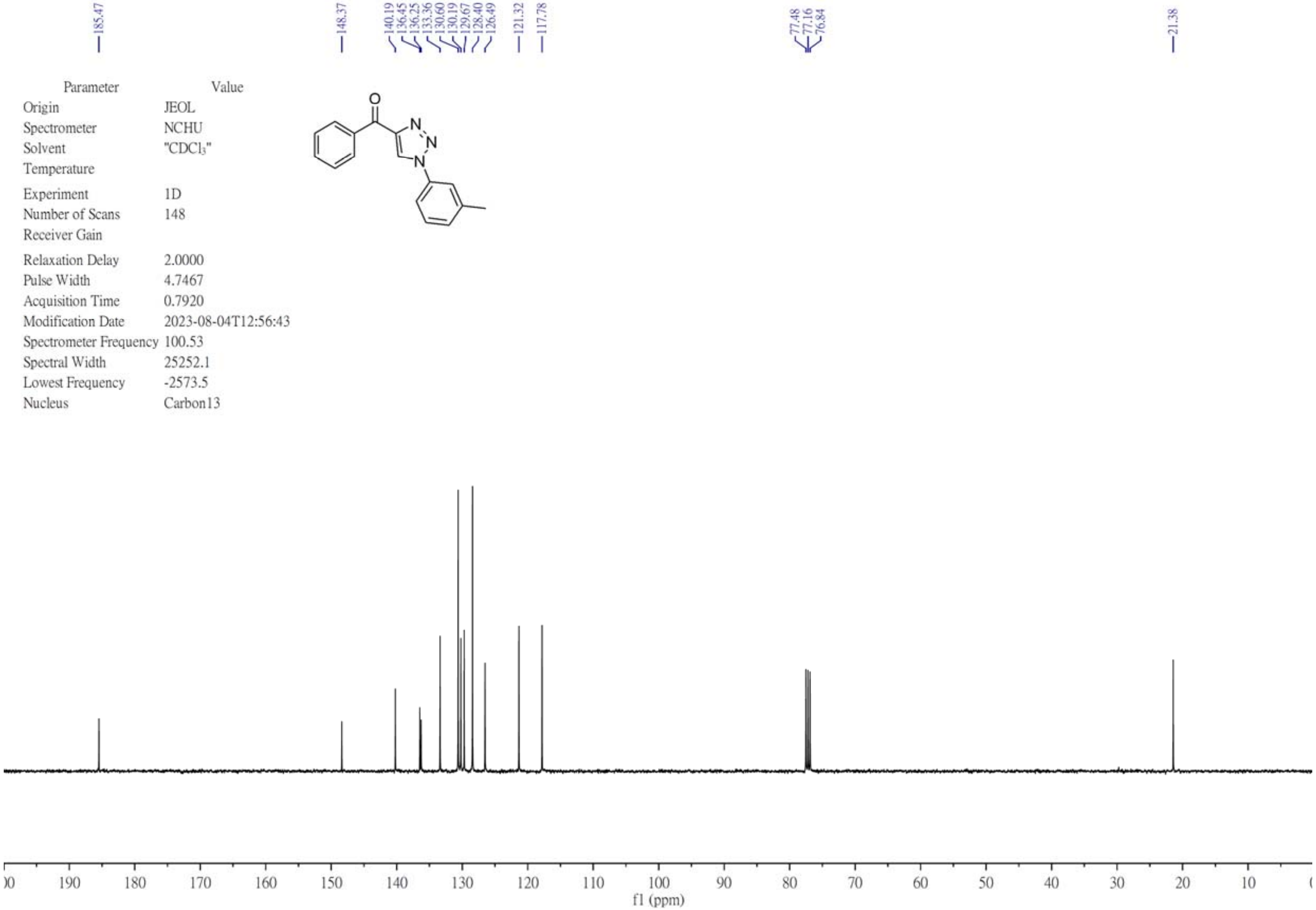

4ac $^{13}C\{^{1}H\}$ NMR spectrum (100 MHz in $CDCl_3$)

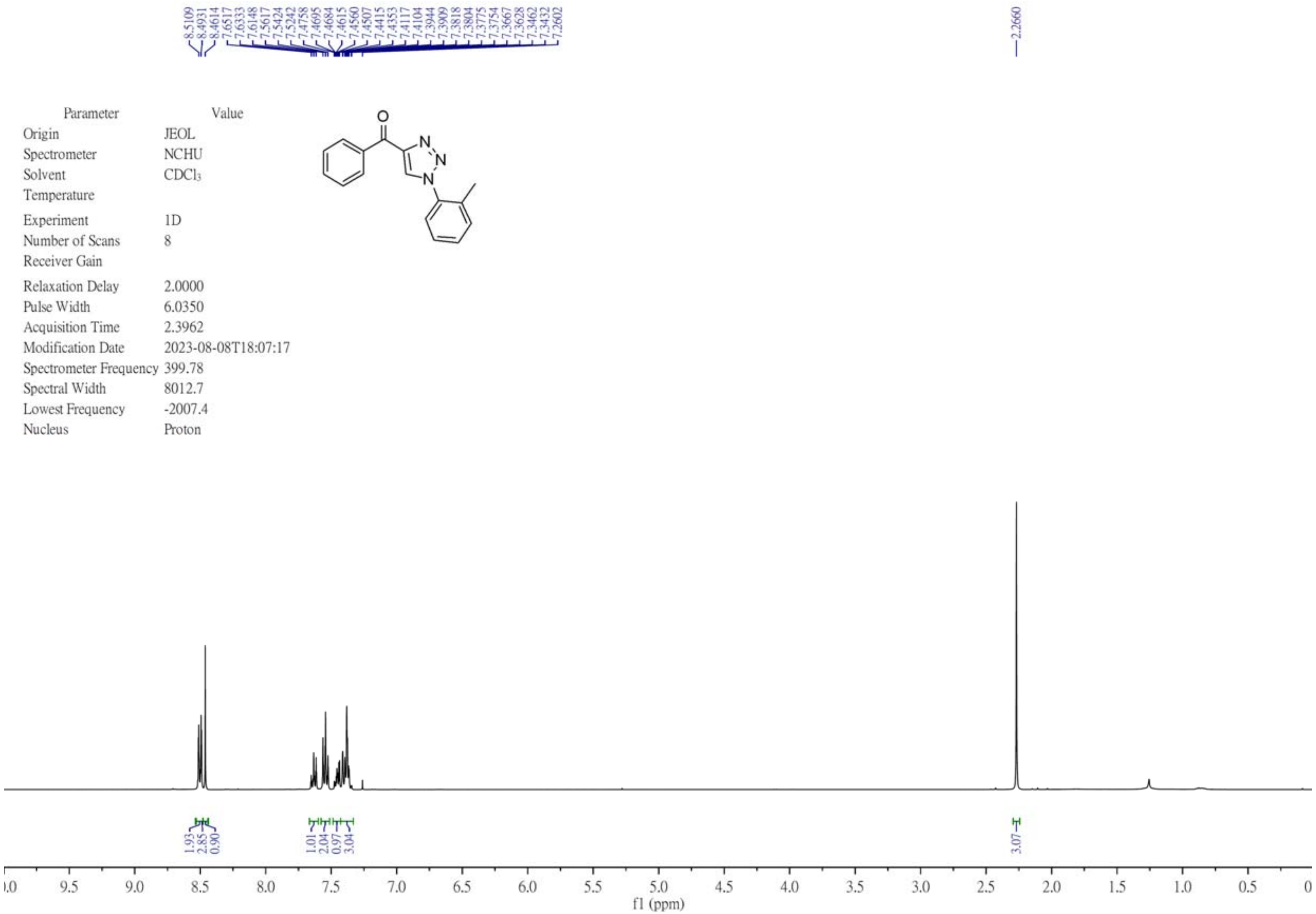

4ad ^{1}H NMR spectrum (400 MHz in $CDCl_3$)

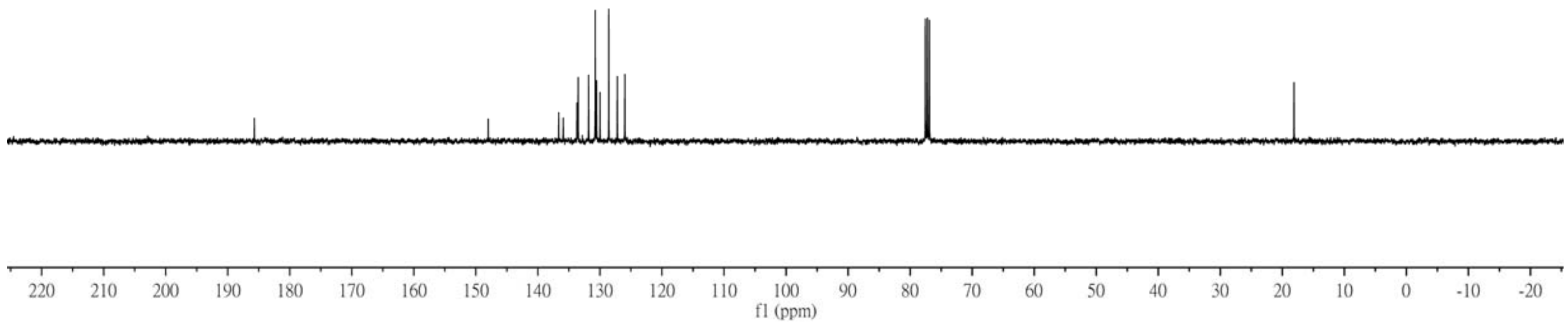

Parameter	Value
Origin	JEOL
Spectrometer	NCHU
Solvent	$CDCl_3$
Temperature	
Experiment	1D
Number of Scans	89
Receiver Gain	
Relaxation Delay	2.0000
Pulse Width	4.7467
Acquisition Time	0.7920
Modification Date	2023-08-08T18:12:06
Spectrometer Frequency	100.53
Spectral Width	25252.1
Lowest Frequency	-2574.1
Nucleus	Carbon13

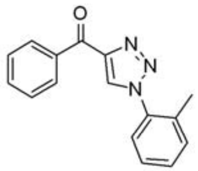

4ad $^{13}C\{^1H\}$ NMR spectrum (100 MHz in $CDCl_3$)

Parameter	Value
Origin	Varian
Spectrometer	vnmrs
Solvent	"$CDCl_3$"
Temperature	25.0
Pulse Sequence	s2pul
Experiment	1D
Number of Scans	12
Receiver Gain	18
Relaxation Delay	1.0000
Pulse Width	0.0000
Acquisition Time	2.5559
Modification Date	2023-05-15T20:29:00
Spectrometer Frequency	399.76
Spectral Width	6410.3
Lowest Frequency	-798.6
Nucleus	1H

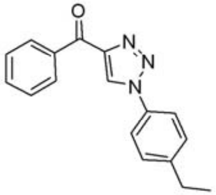

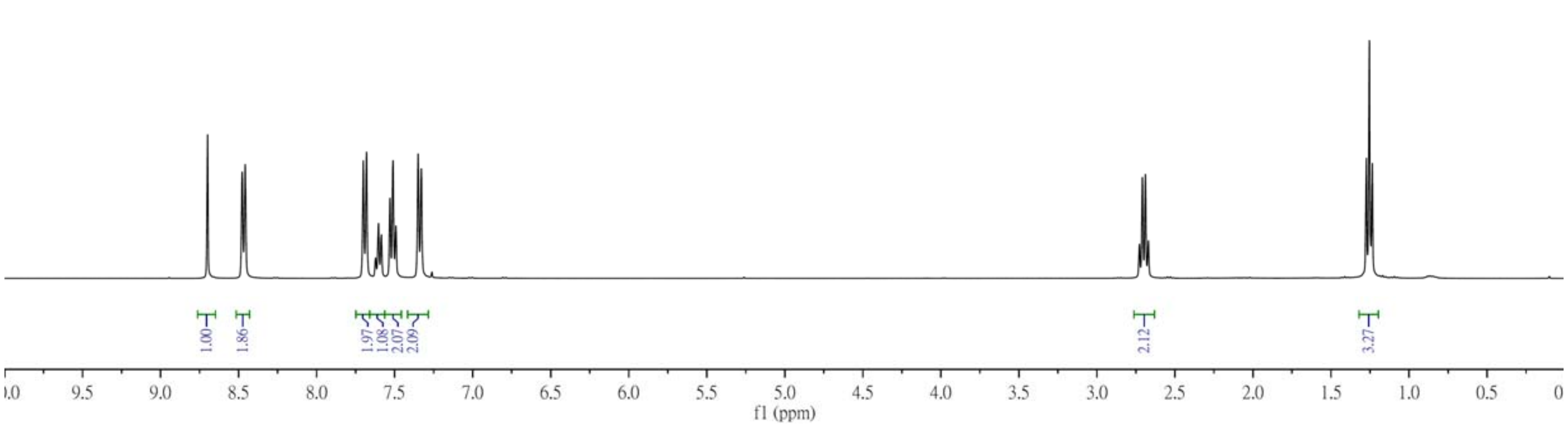

4ae ^1H NMR spectrum (400 MHz in $CDCl_3$)

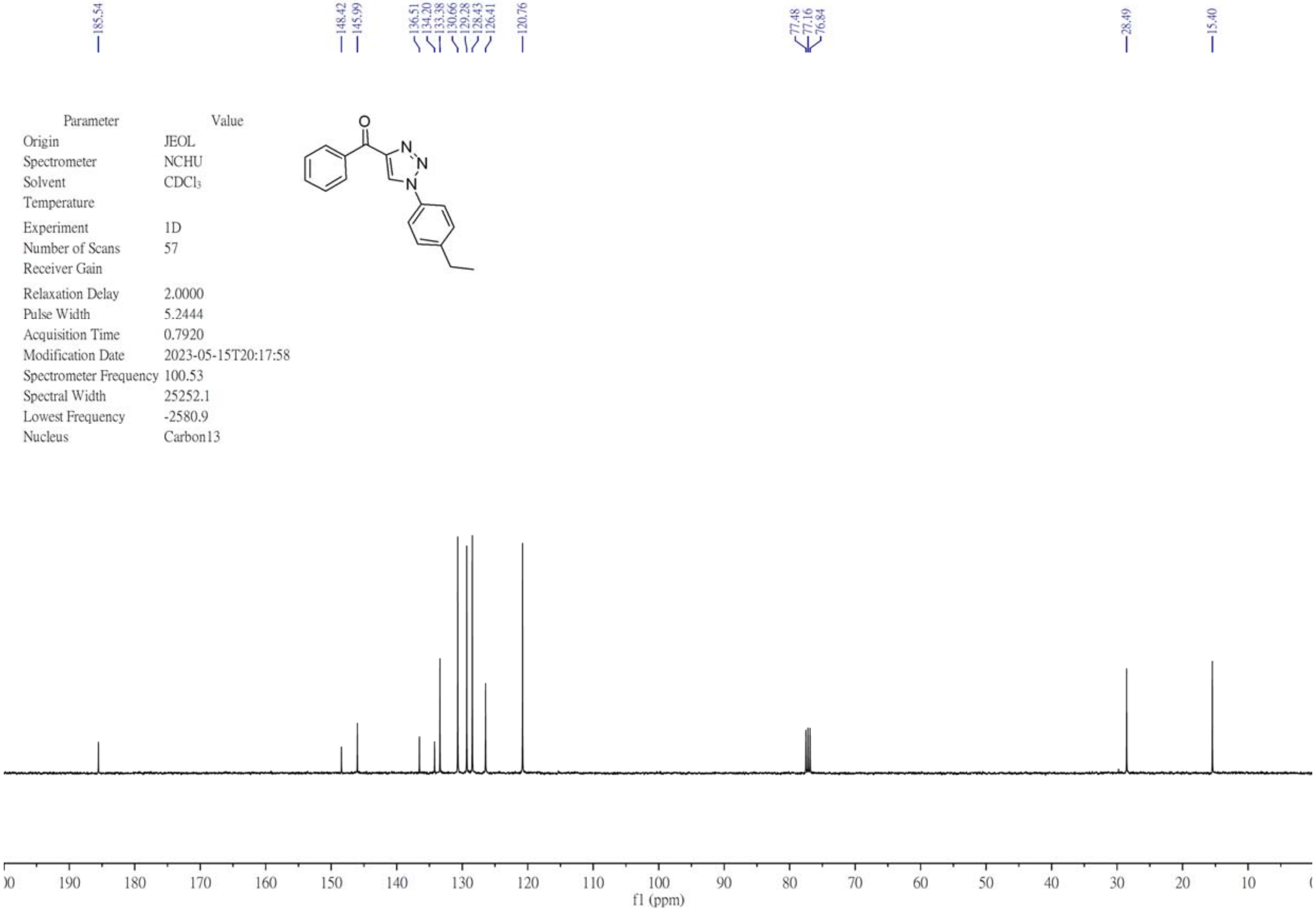

4ae $^{13}C\{^{1}H\}$ NMR spectrum (100 MHz in $CDCl_3$)

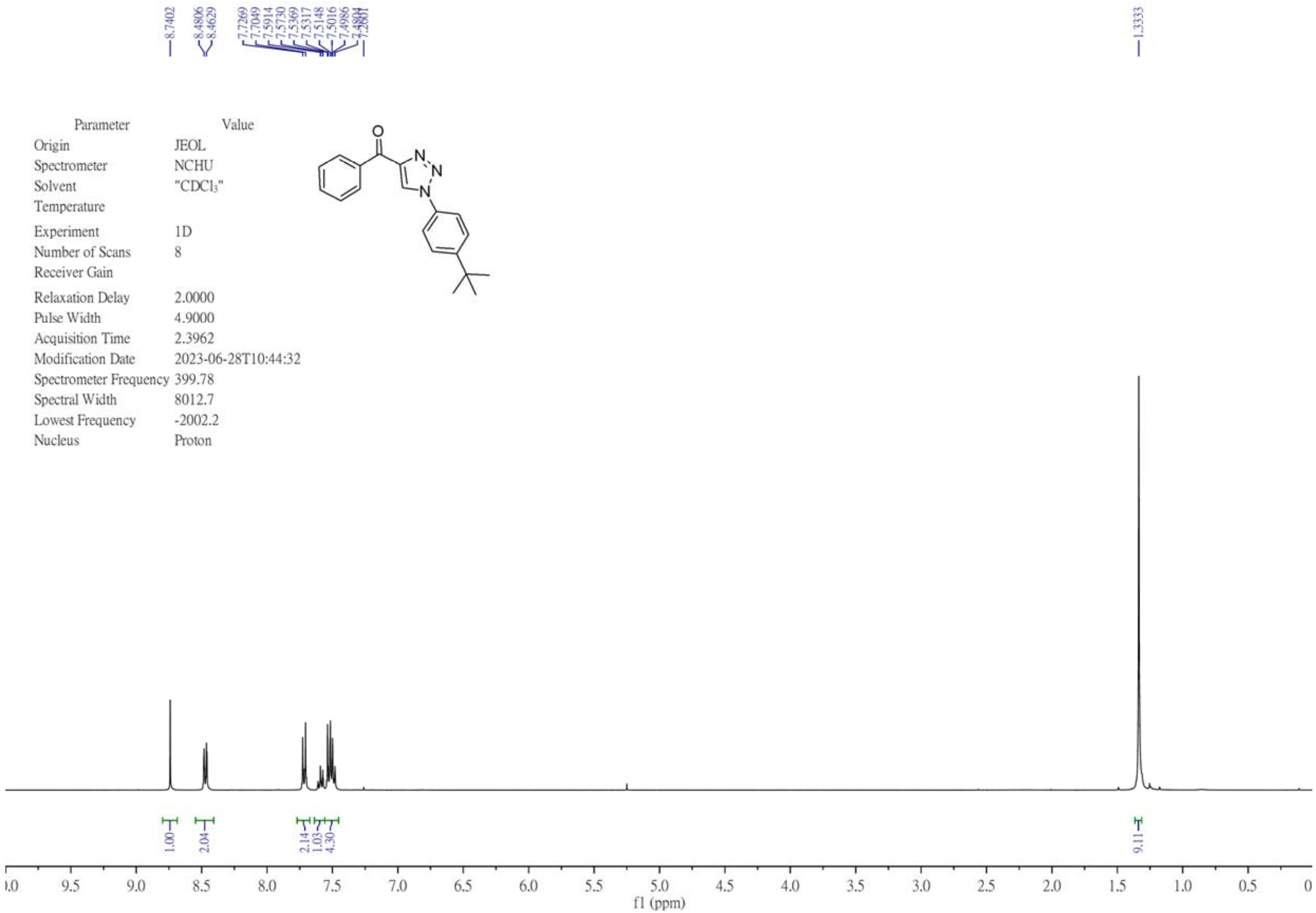

4af ^{1}H NMR spectrum (400 MHz in $CDCl_3$)

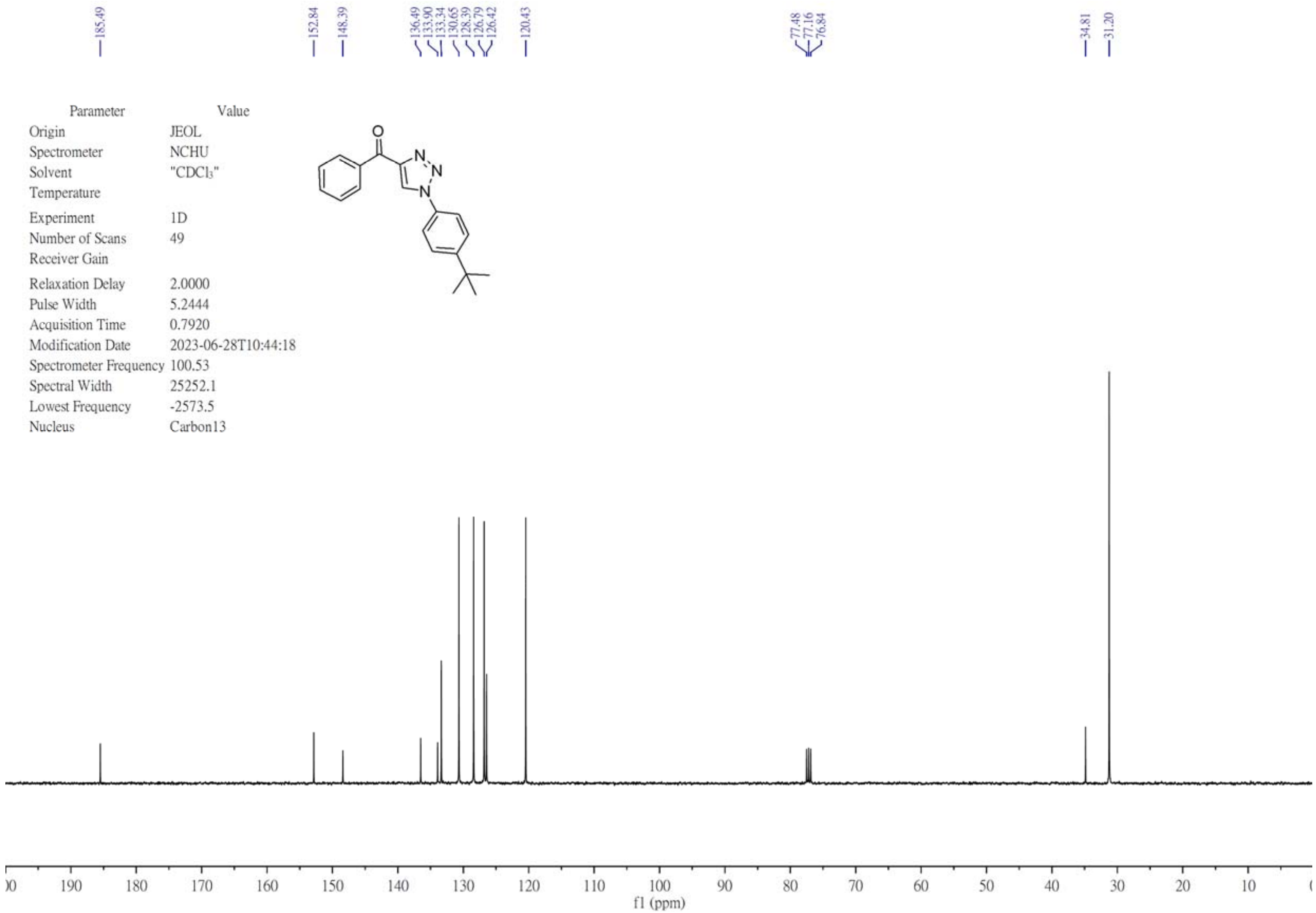

4af $^{13}C\{^{1}H\}$ NMR spectrum (100 MHz in $CDCl_3$)

Parameter	Value
Origin	JEOL
Spectrometer	NCHU
Solvent	"$CDCl_3$"
Temperature	
Pulse Sequence	
Experiment	1D
Number of Scans	8
Receiver Gain	
Relaxation Delay	2.0000
Pulse Width	4.9000
Acquisition Time	2.3962
Modification Date	2023-06-16T13:54:19
Spectrometer Frequency	399.78
Spectral Width	8012.7
Lowest Frequency	-2002.2
Nucleus	Proton

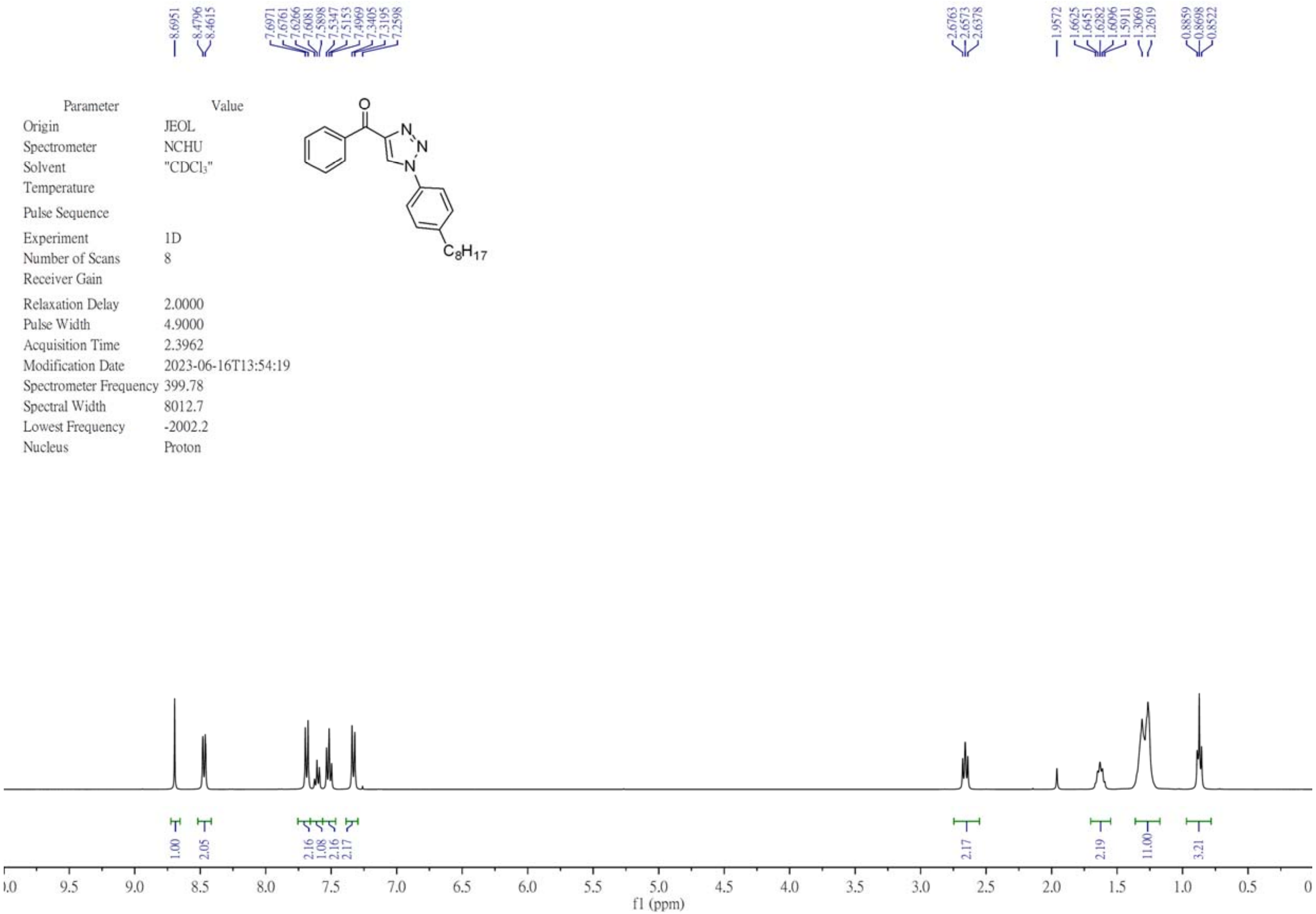

4ag ^{1}H NMR spectrum (400 MHz in $CDCl_3$)

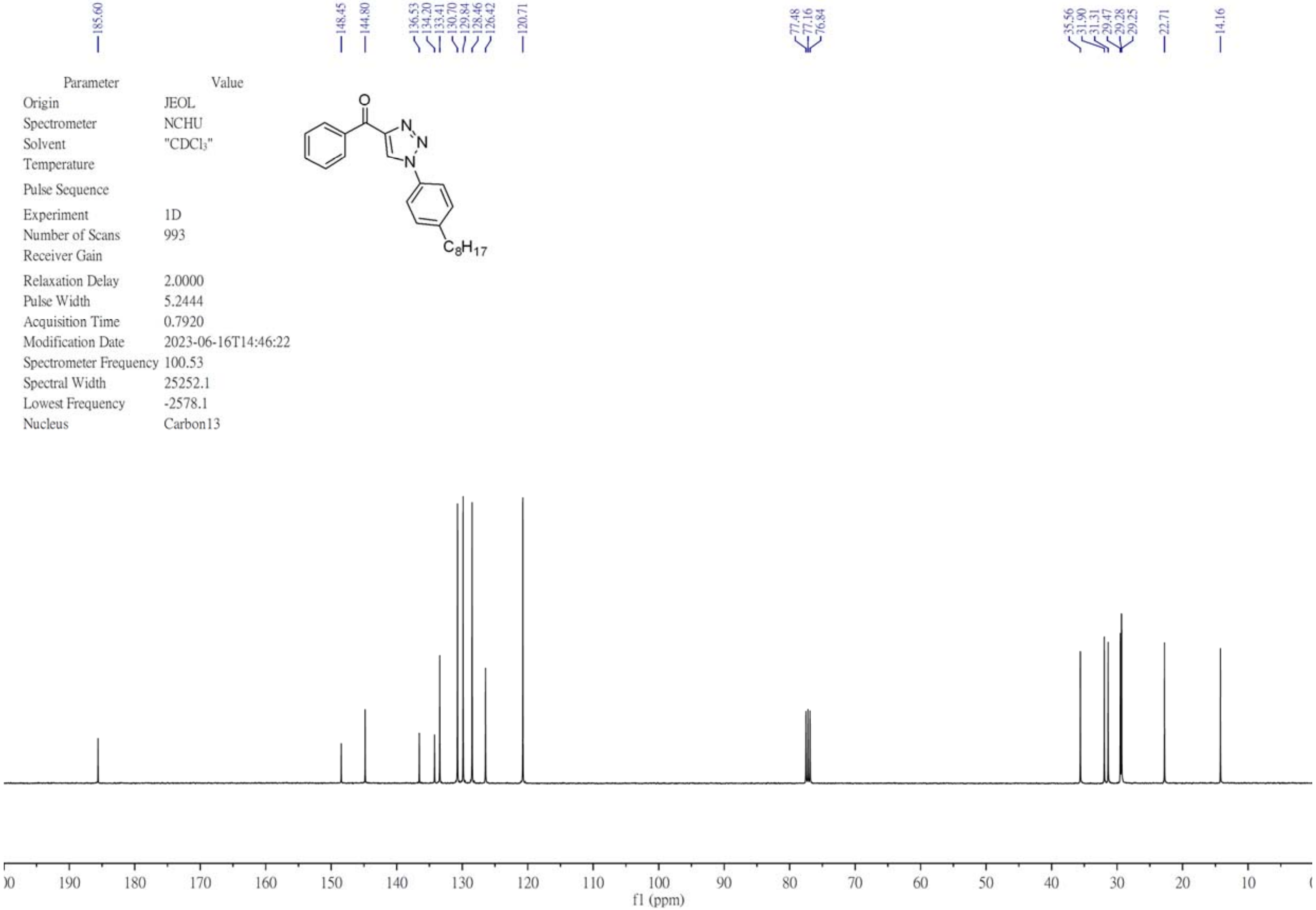

4ag $^{13}C\{^{1}H\}$ NMR spectrum (100 MHz in $CDCl_3$)

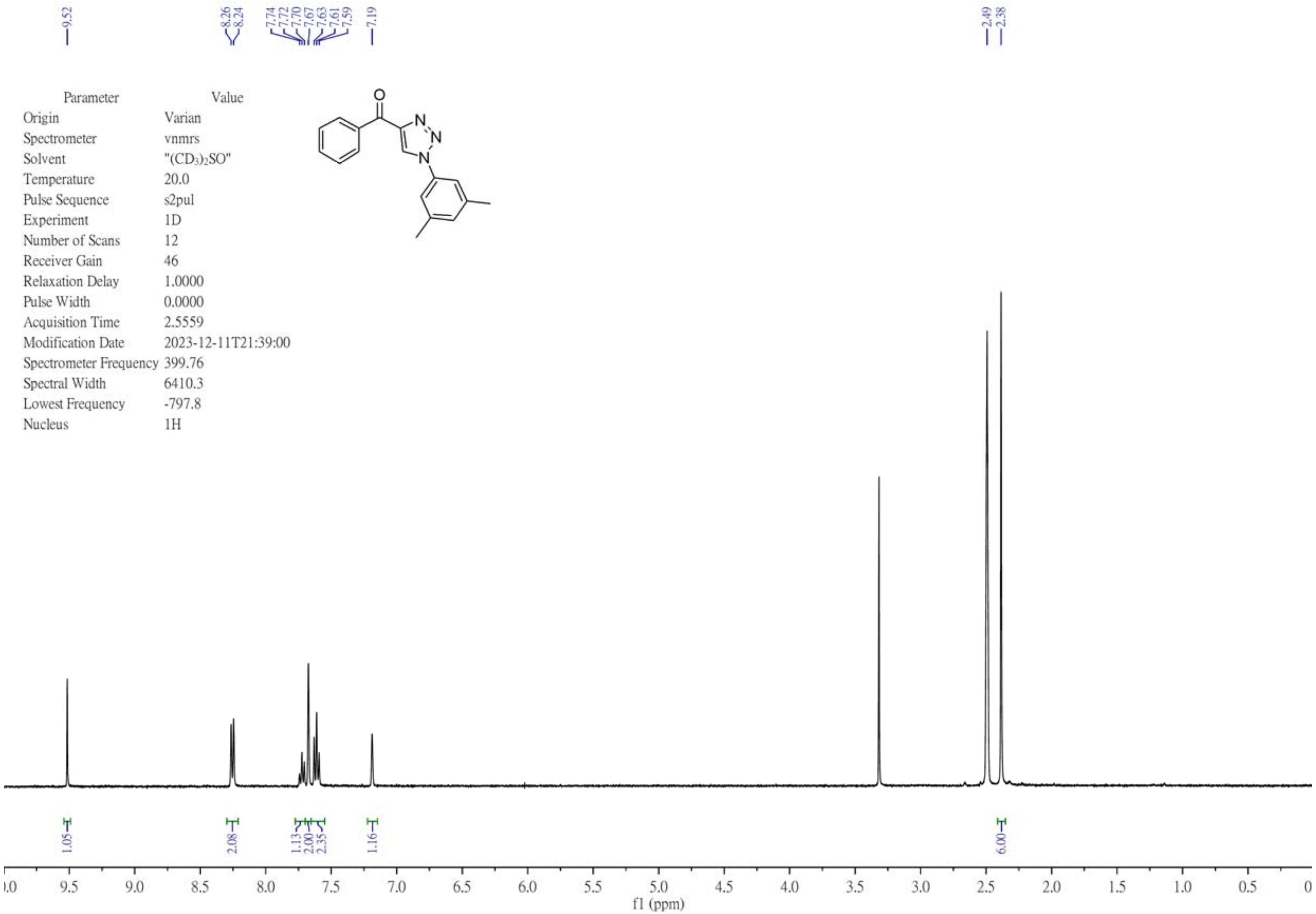

4ah ^{1}H NMR spectrum (400 MHz in $(CD_3)_2SO$)

Parameter	Value
Origin	JEOL
Spectrometer	NCHU
Solvent	"$CDCl_3$"
Temperature	
Experiment	1D
Number of Scans	45
Receiver Gain	
Relaxation Delay	2.0000
Pulse Width	5.2444
Acquisition Time	0.7920
Modification Date	2023-05-26T16:43:49
Spectrometer Frequency	100.53
Spectral Width	25252.1
Lowest Frequency	-2585.1
Nucleus	Carbon13

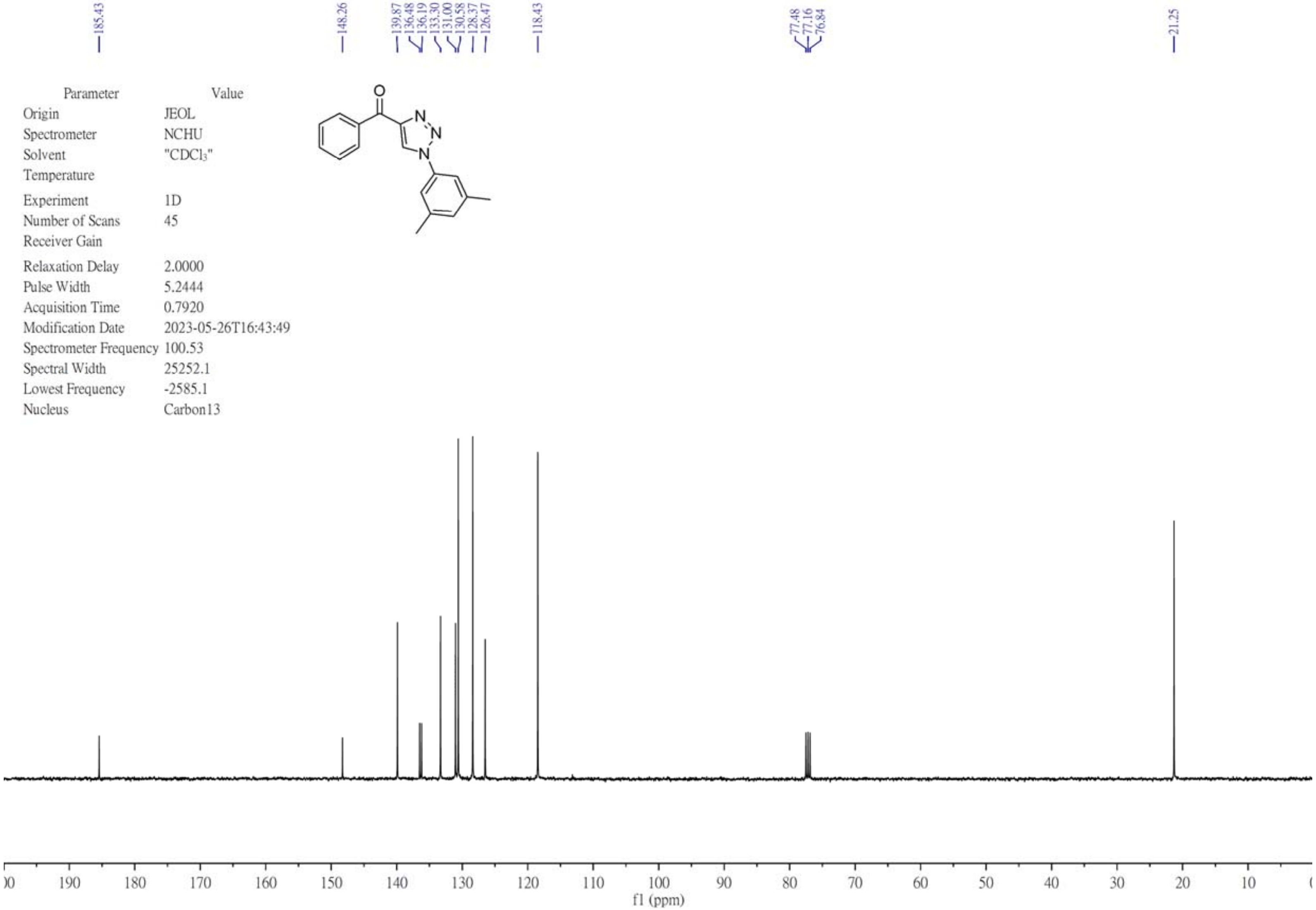

4ah $^{13}C\{^1H\}$ NMR spectrum (100 MHz in $CDCl_3$)

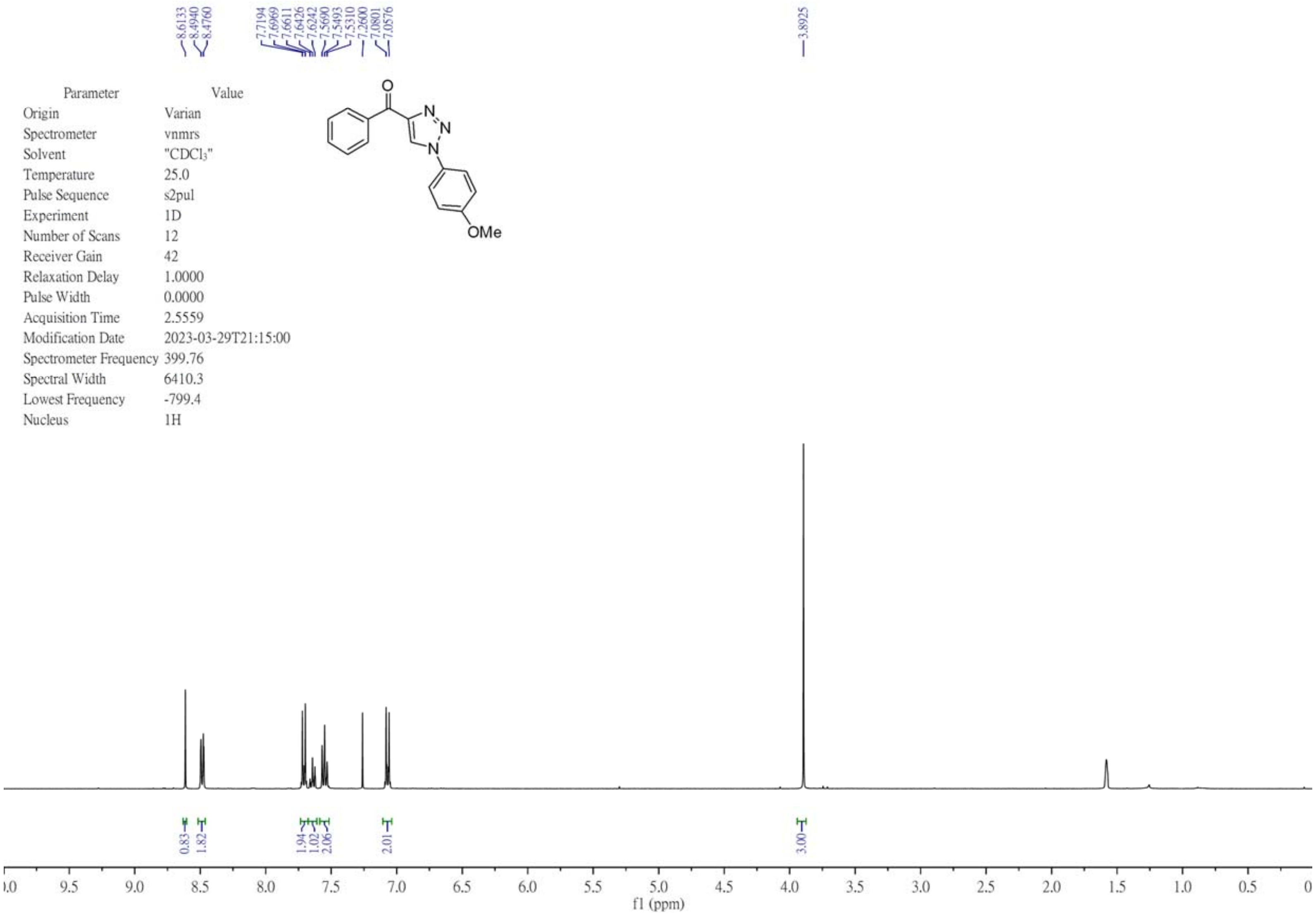

4ai ^{1}H NMR spectrum (400 MHz in $CDCl_3$)

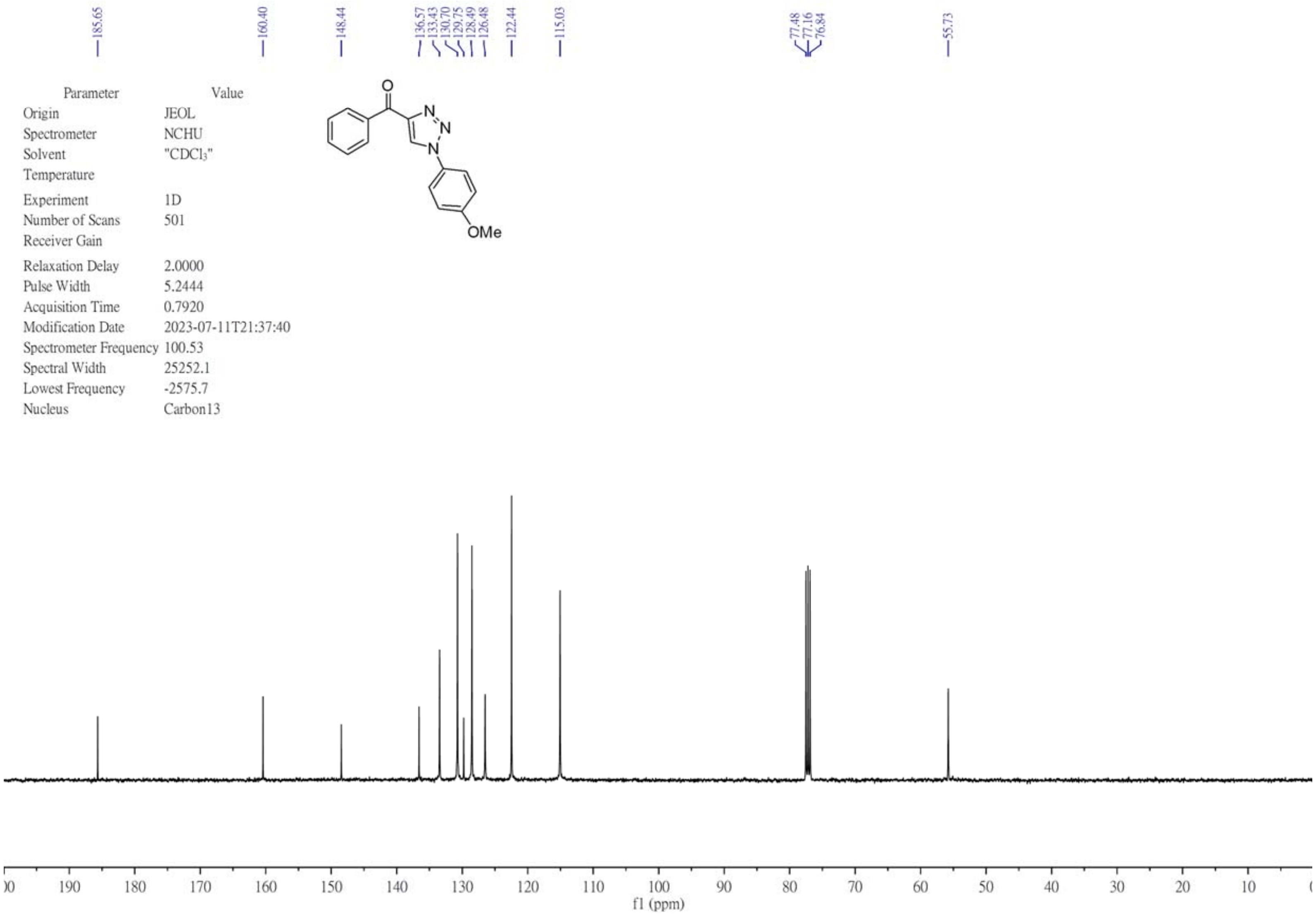

4ai $^{13}C\{^{1}H\}$ NMR spectrum (100 MHz in $CDCl_3$)

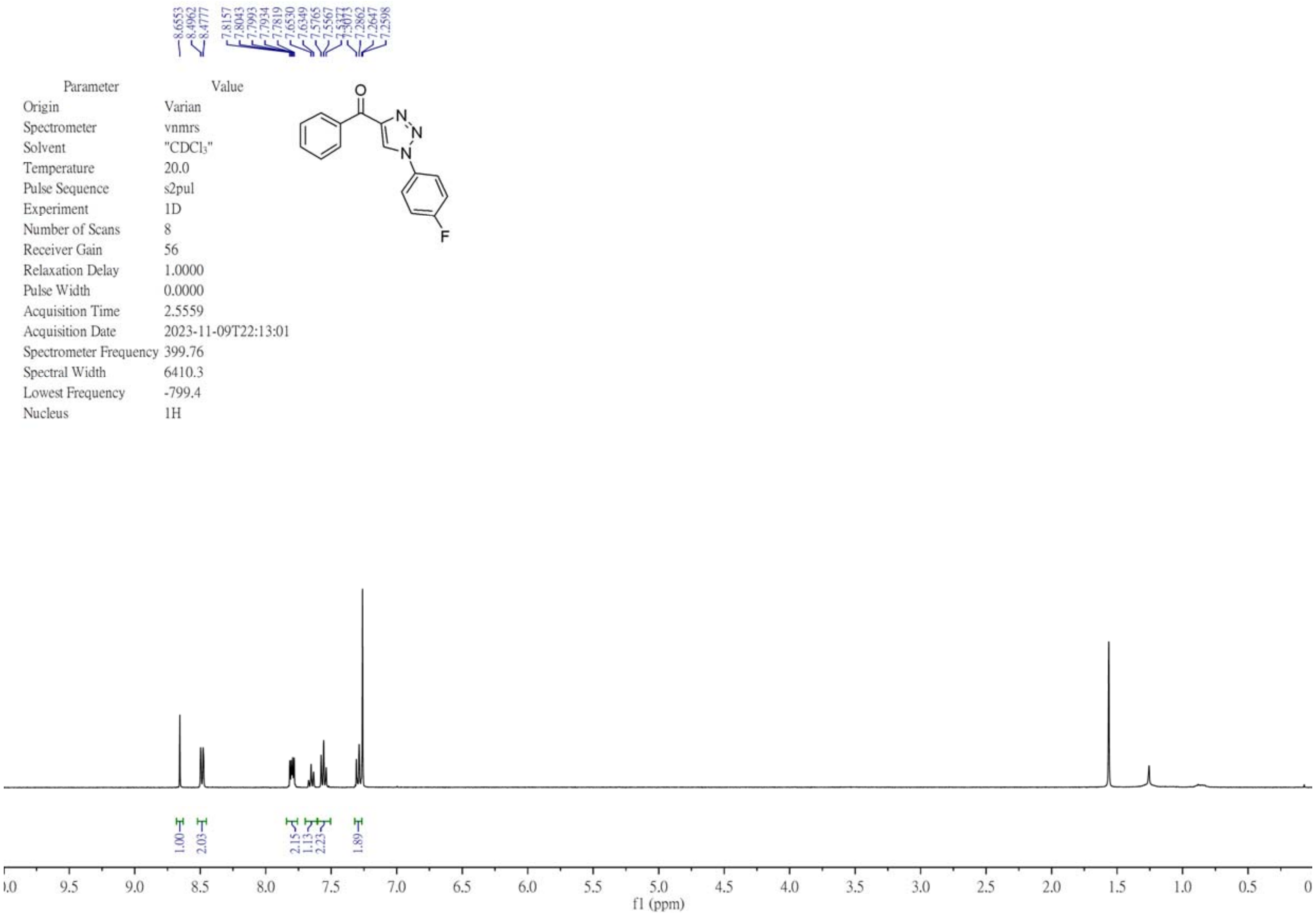

4aj ^{1}H NMR spectrum (400 MHz in $CDCl_3$)

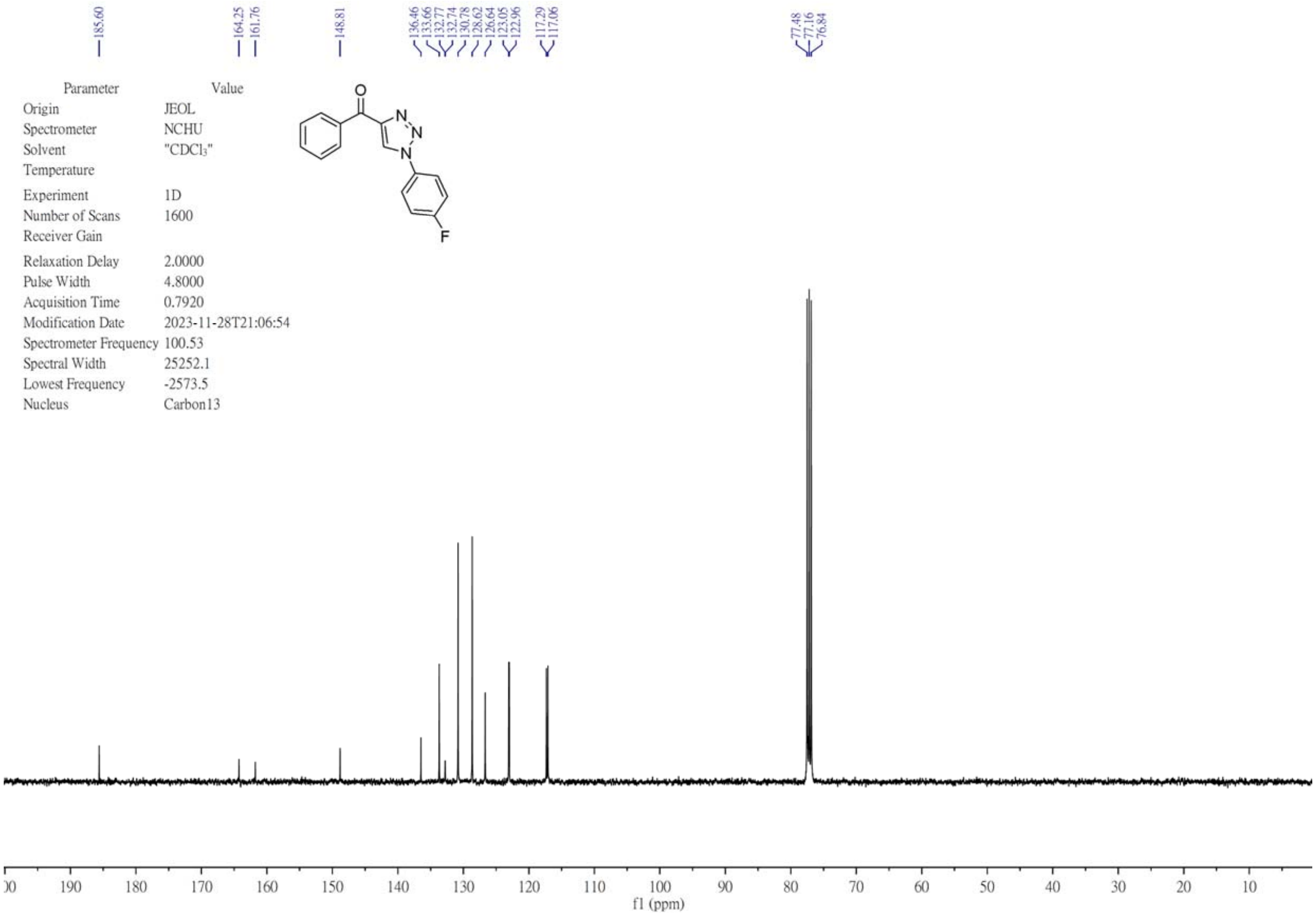

4aj $^{13}C\{^{1}H\}$ NMR spectrum (100 MHz in $CDCl_3$)

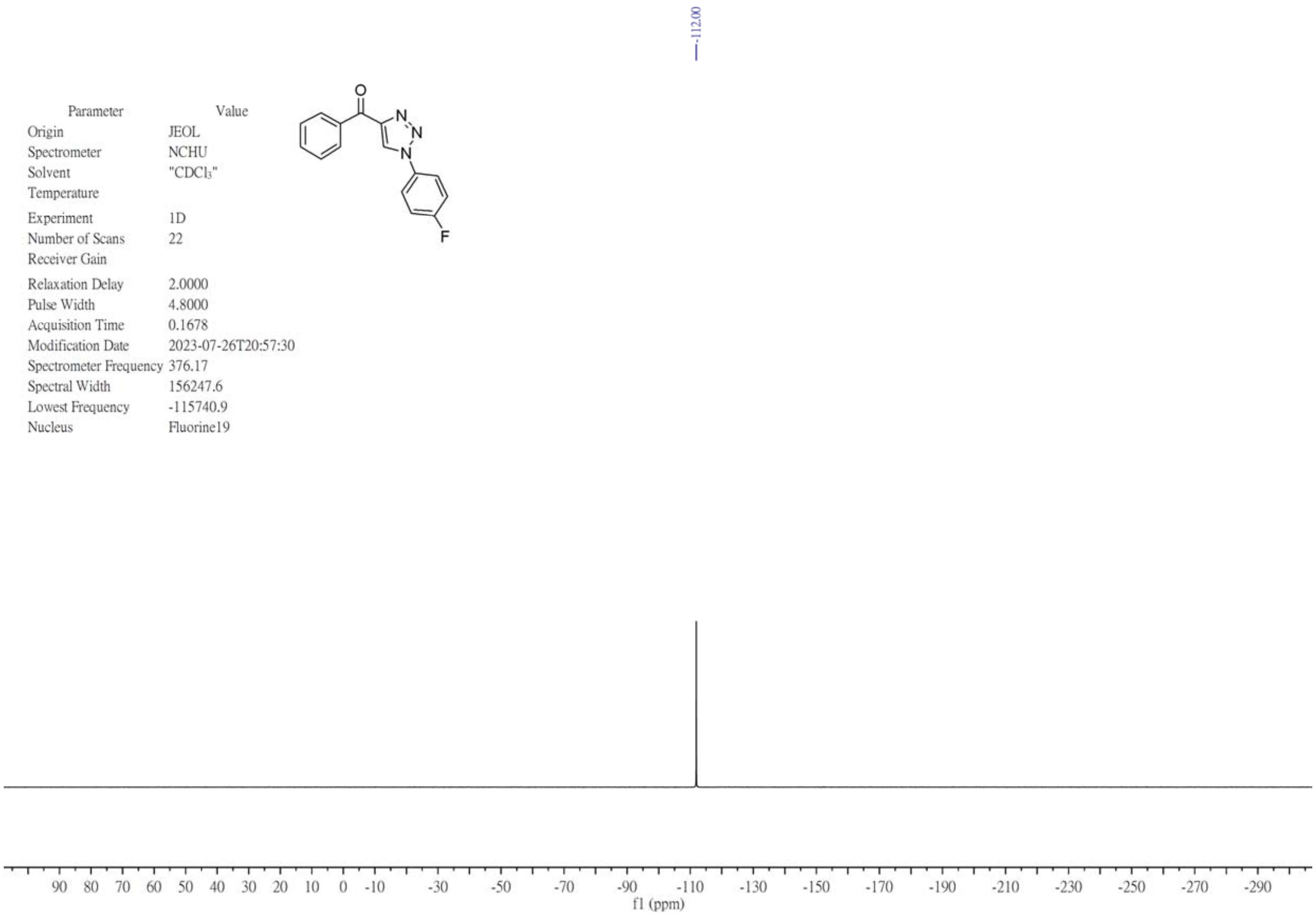

4aj ^{19}F NMR spectrum (376 MHz, $CDCl_3$)

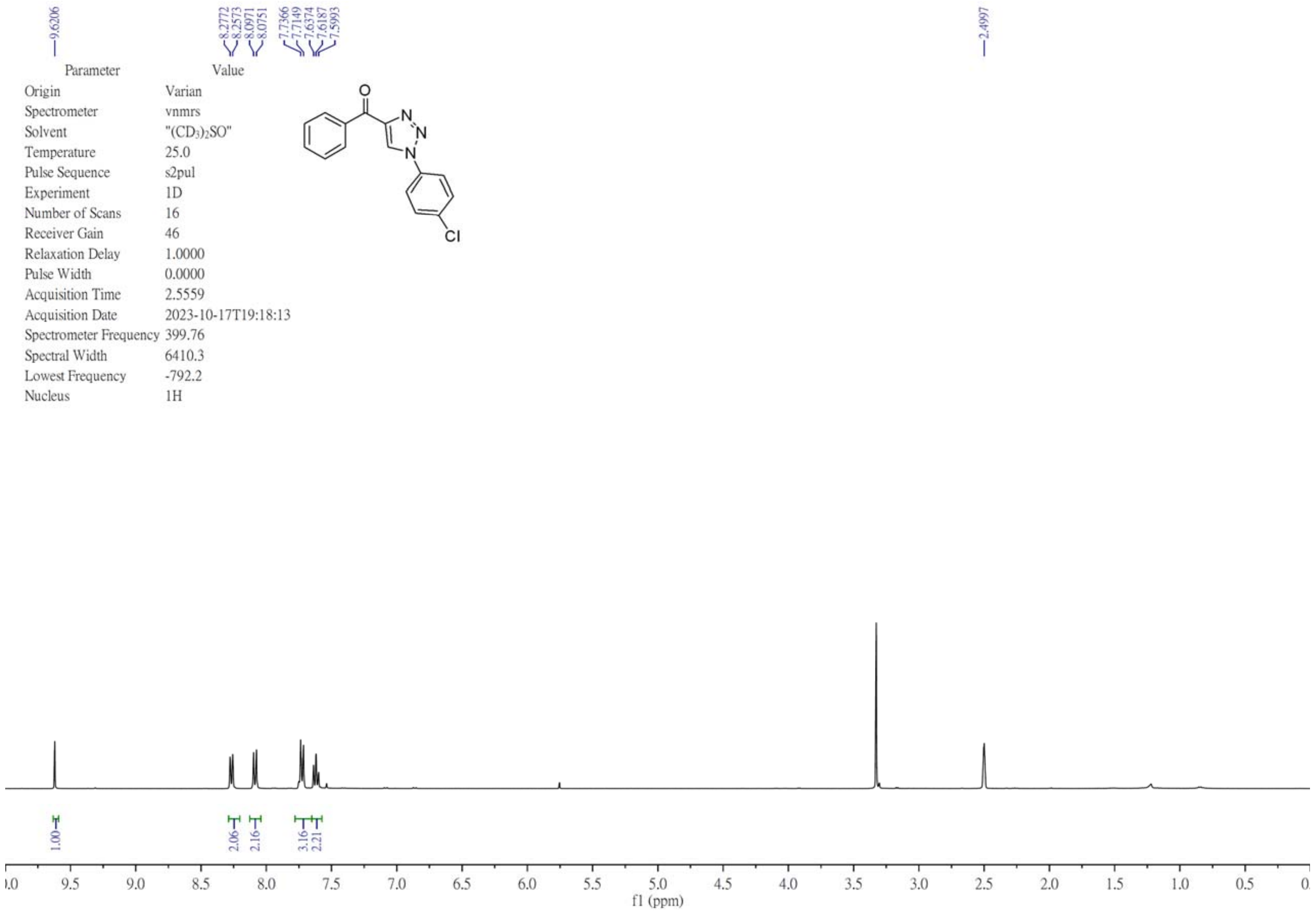

4ak ^{1}H NMR spectrum (400 MHz in $(CD_3)_2SO$)

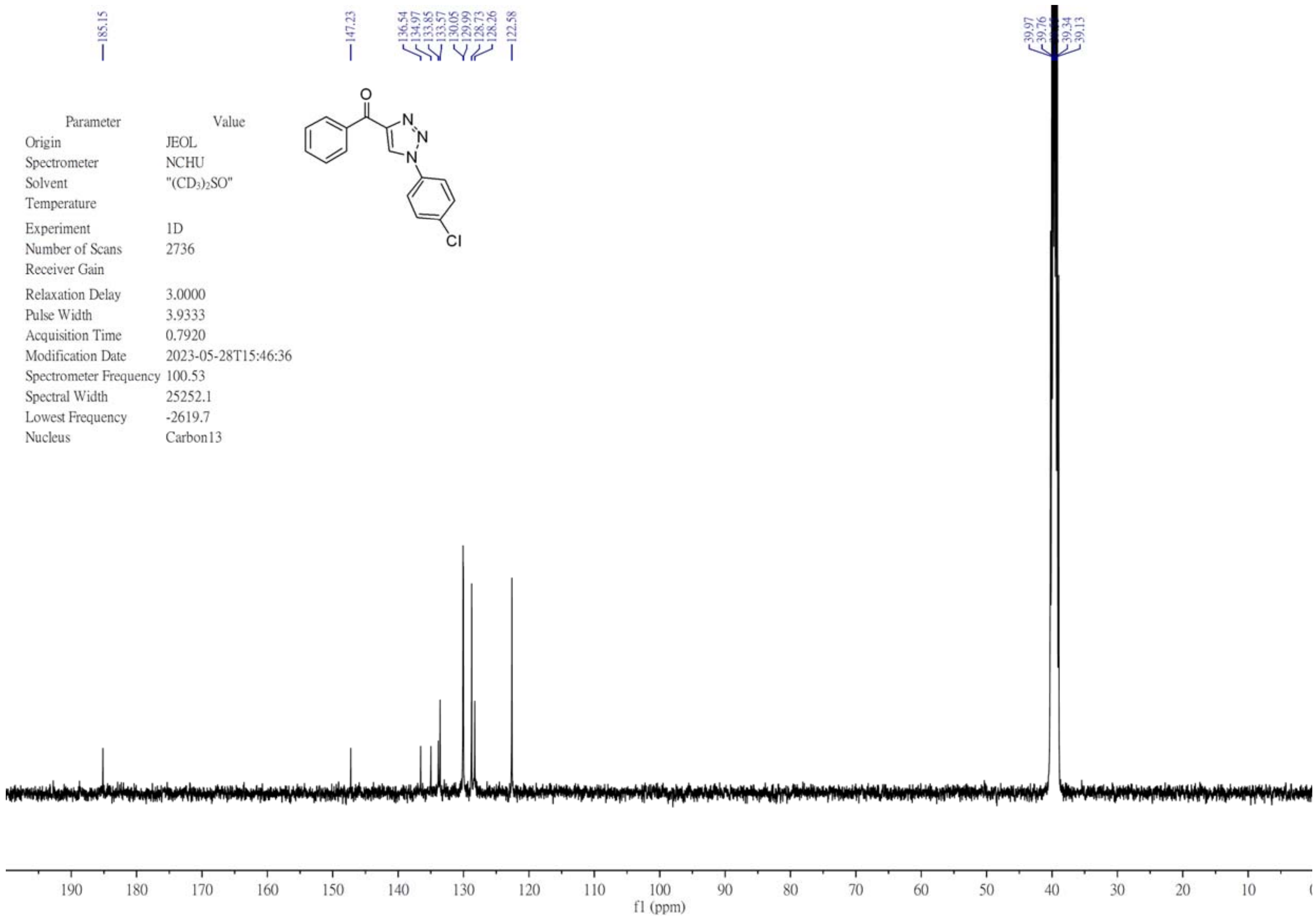

4ak $^{13}C\{^1H\}$ NMR spectrum (100 MHz in $(CD_3)_2SO$)

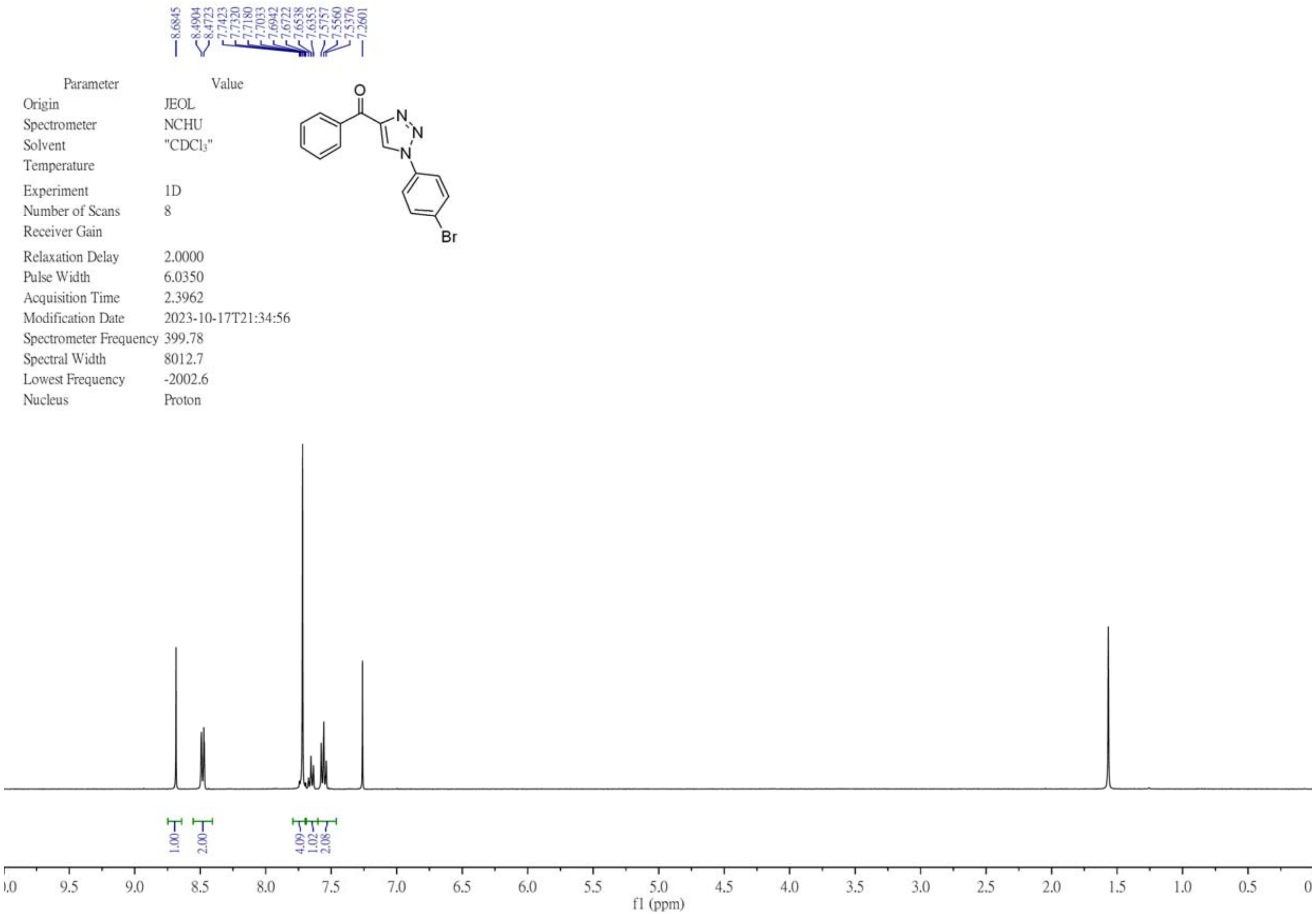

4al ^{1}H NMR spectrum (400 MHz in $CDCl_3$)

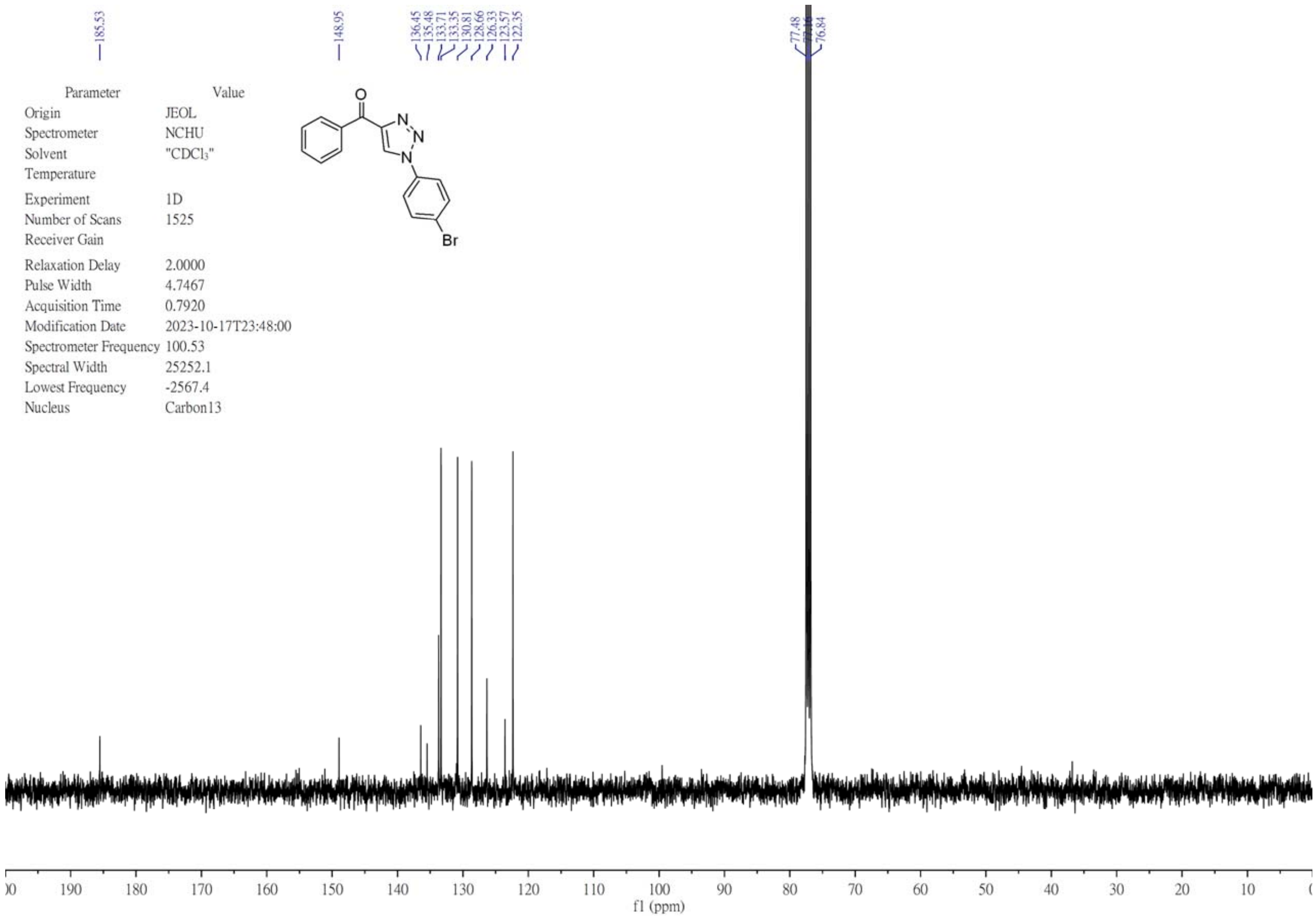

4al $^{13}C\{^{1}H\}$ NMR spectrum (100 MHz in $CDCl_3$)

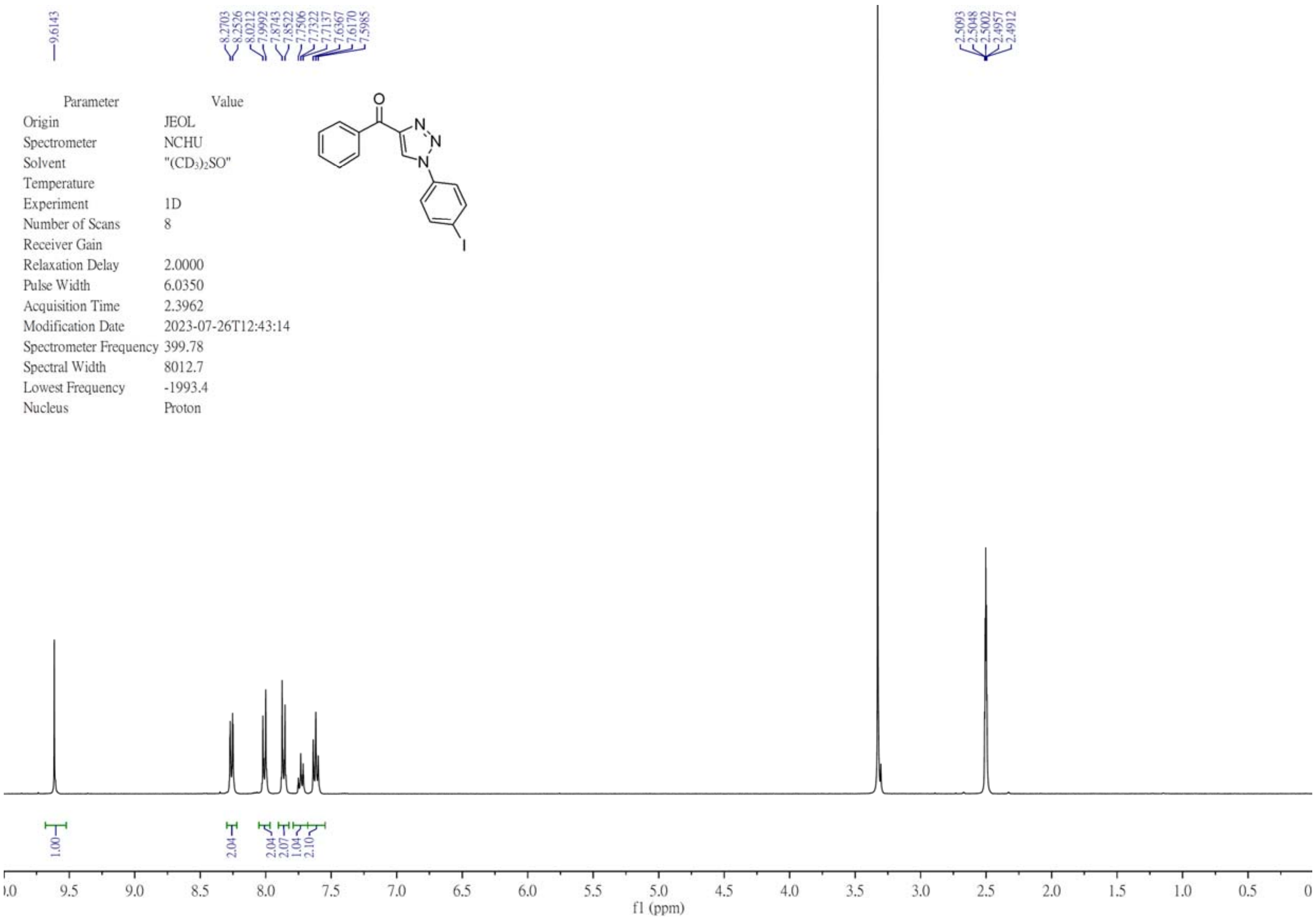

4am ^{1}H NMR spectrum (400 MHz in $(CD_3)_2SO$)

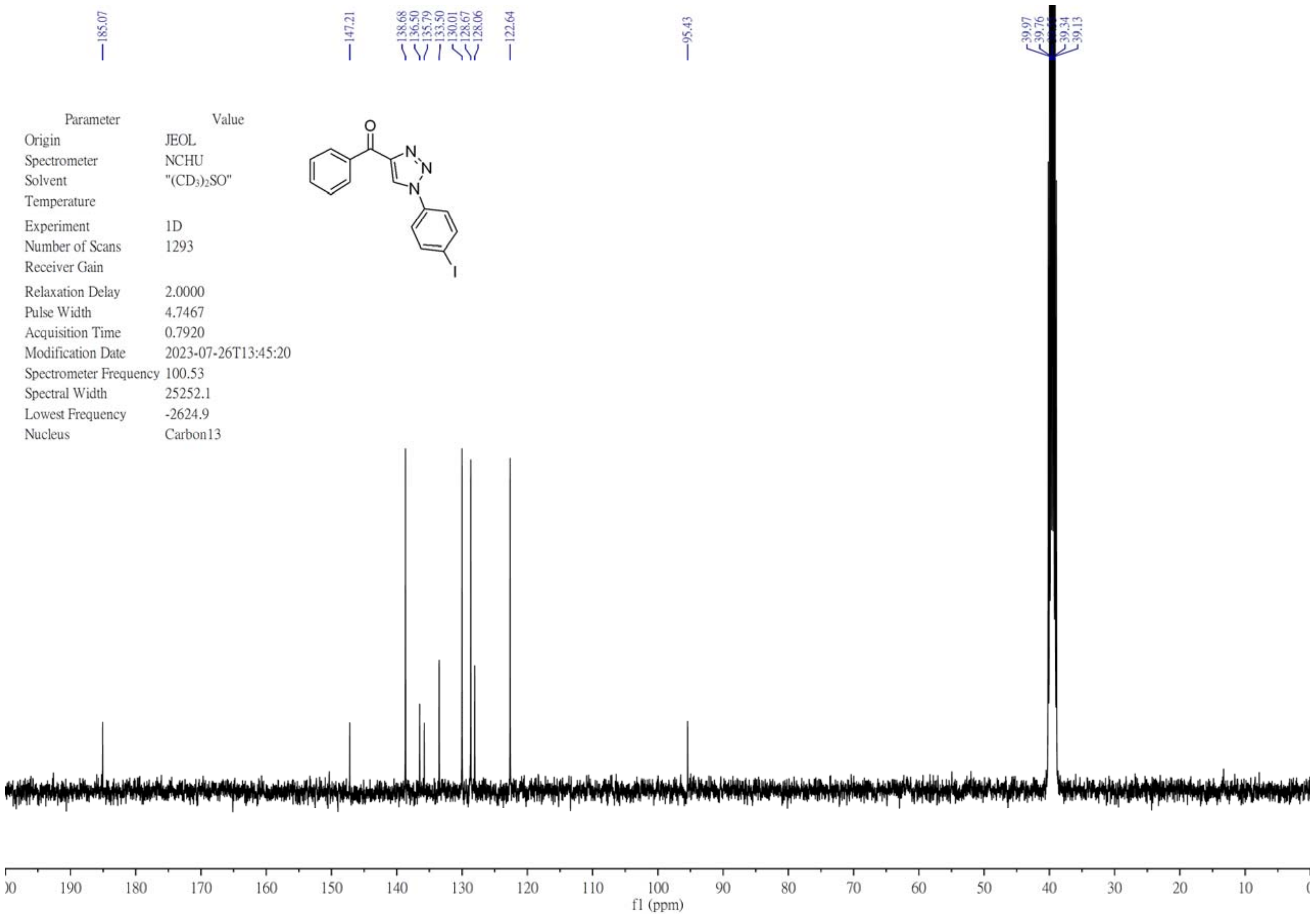

4am $^{13}C\{^{1}H\}$ NMR spectrum (100 MHz in $(CD_3)_2SO$)

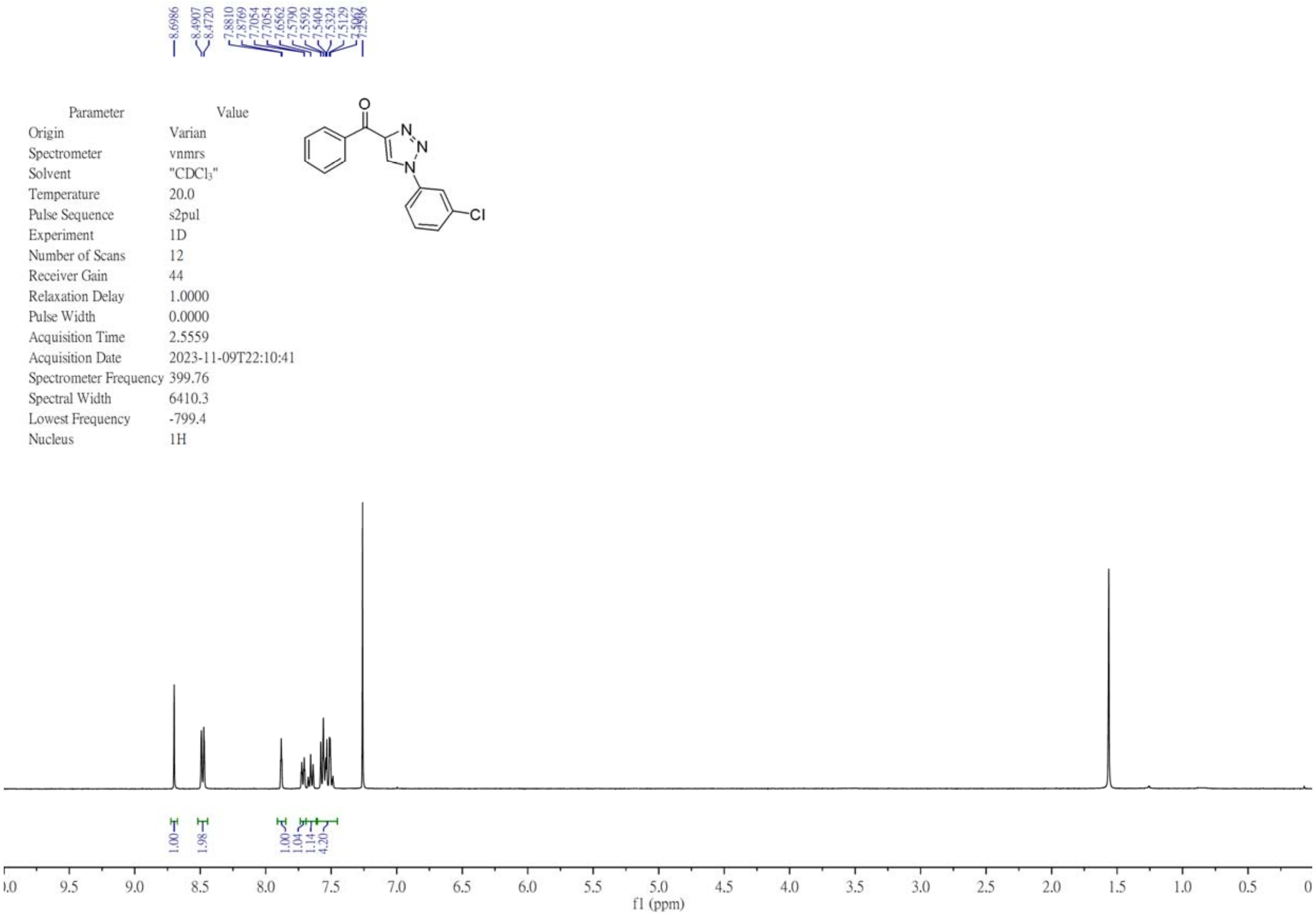

4an ^{1}H NMR spectrum (400 MHz in $CDCl_3$)

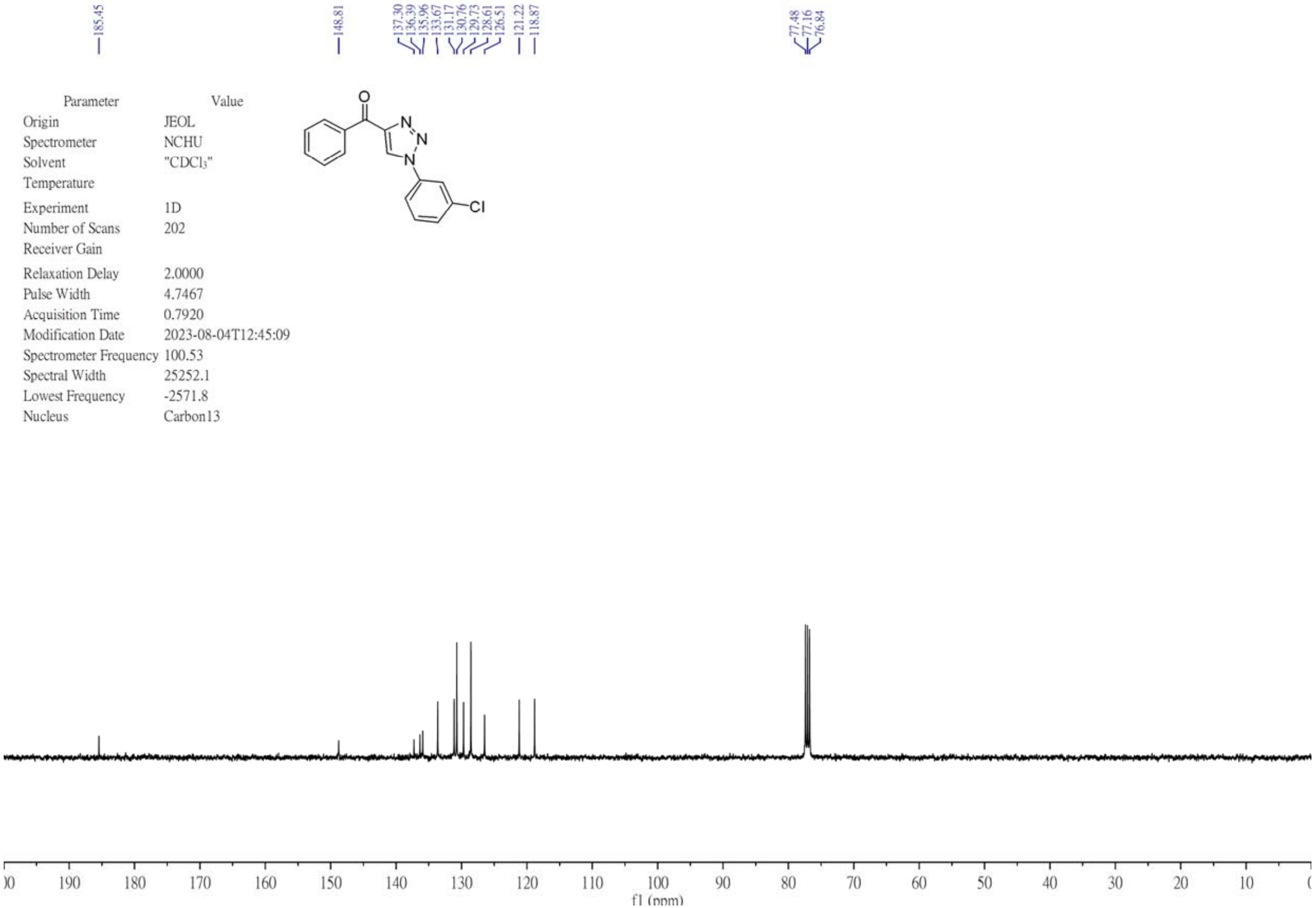

4an $^{13}C\{^{1}H\}$ NMR spectrum (100 MHz in $CDCl_3$)

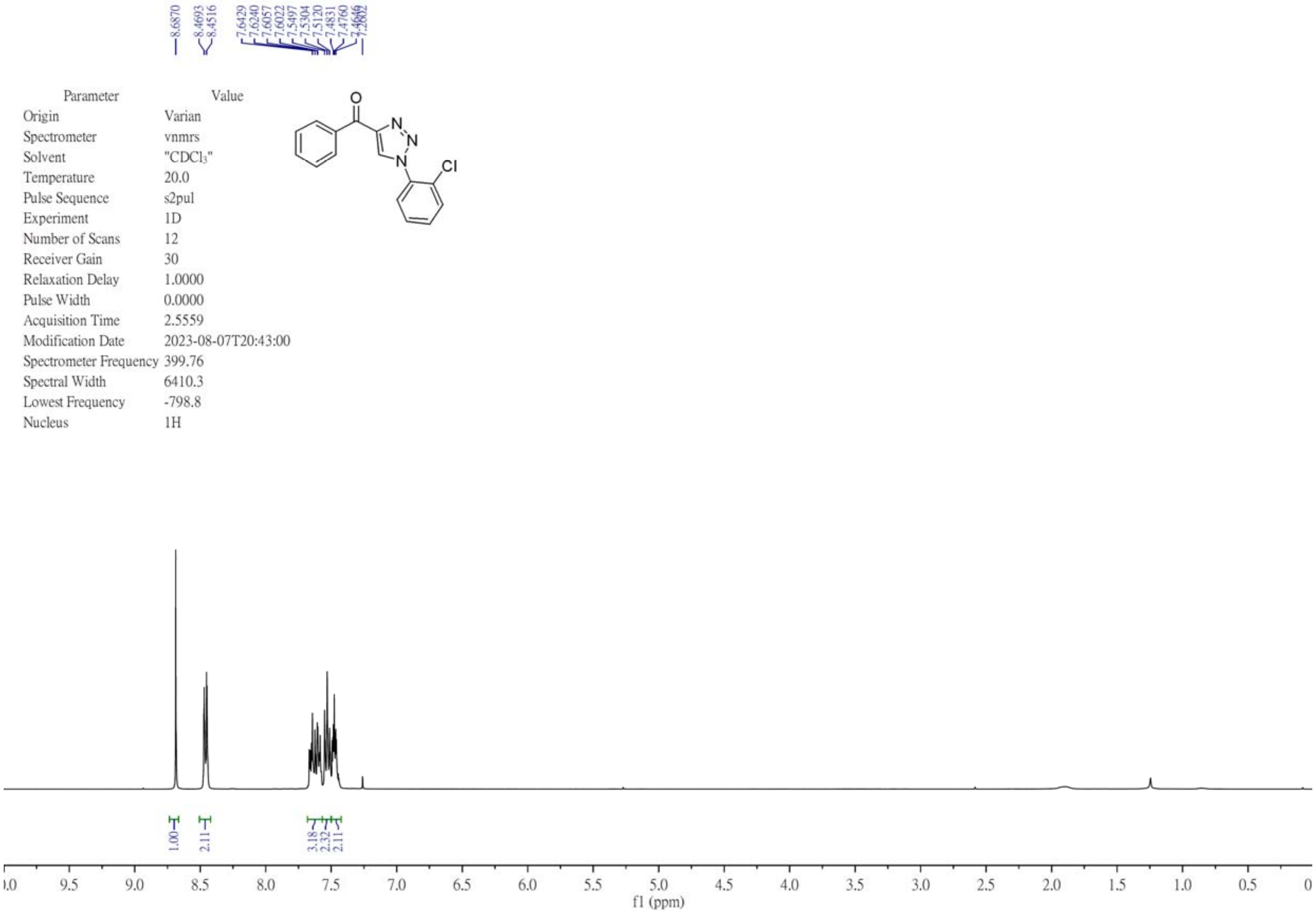

4ao ^{1}H NMR spectrum (400 MHz in $CDCl_3$)

Parameter	Value
Origin	JEOL
Spectrometer	NCHU
Solvent	"$CDCl_3$"
Temperature	
Experiment	1D
Number of Scans	29
Receiver Gain	
Relaxation Delay	2.0000
Pulse Width	4.7467
Acquisition Time	0.7920
Modification Date	2023-10-05T15:27:25
Spectrometer Frequency	100.53
Spectral Width	25252.1
Lowest Frequency	-2582.2
Nucleus	Carbon13

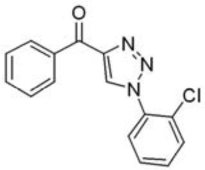

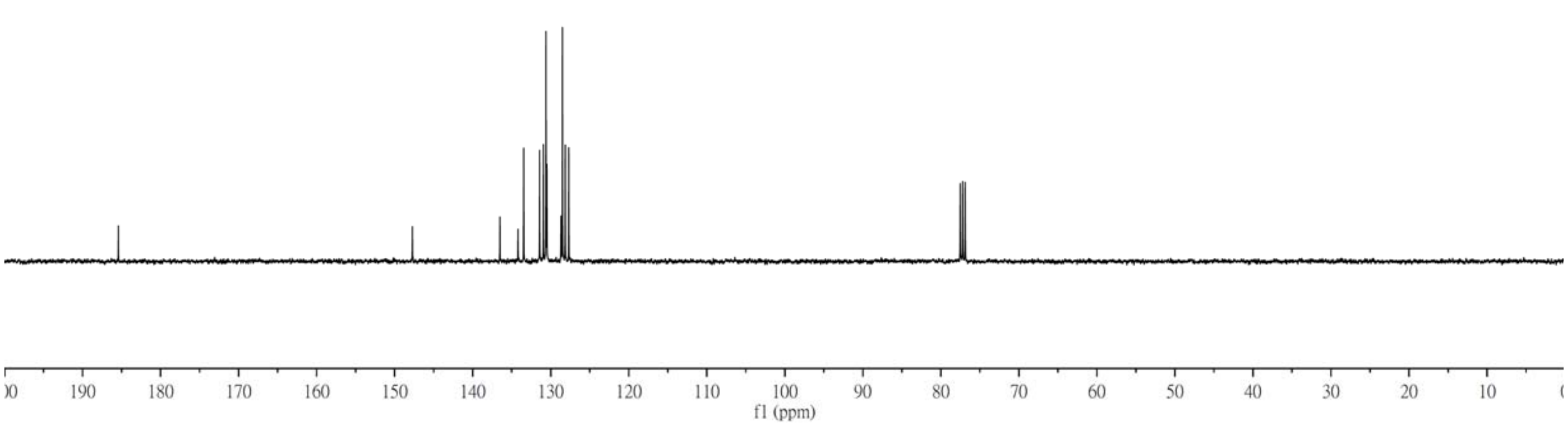

4ao $^{13}C\{^1H\}$ NMR spectrum (100 MHz in $CDCl_3$)

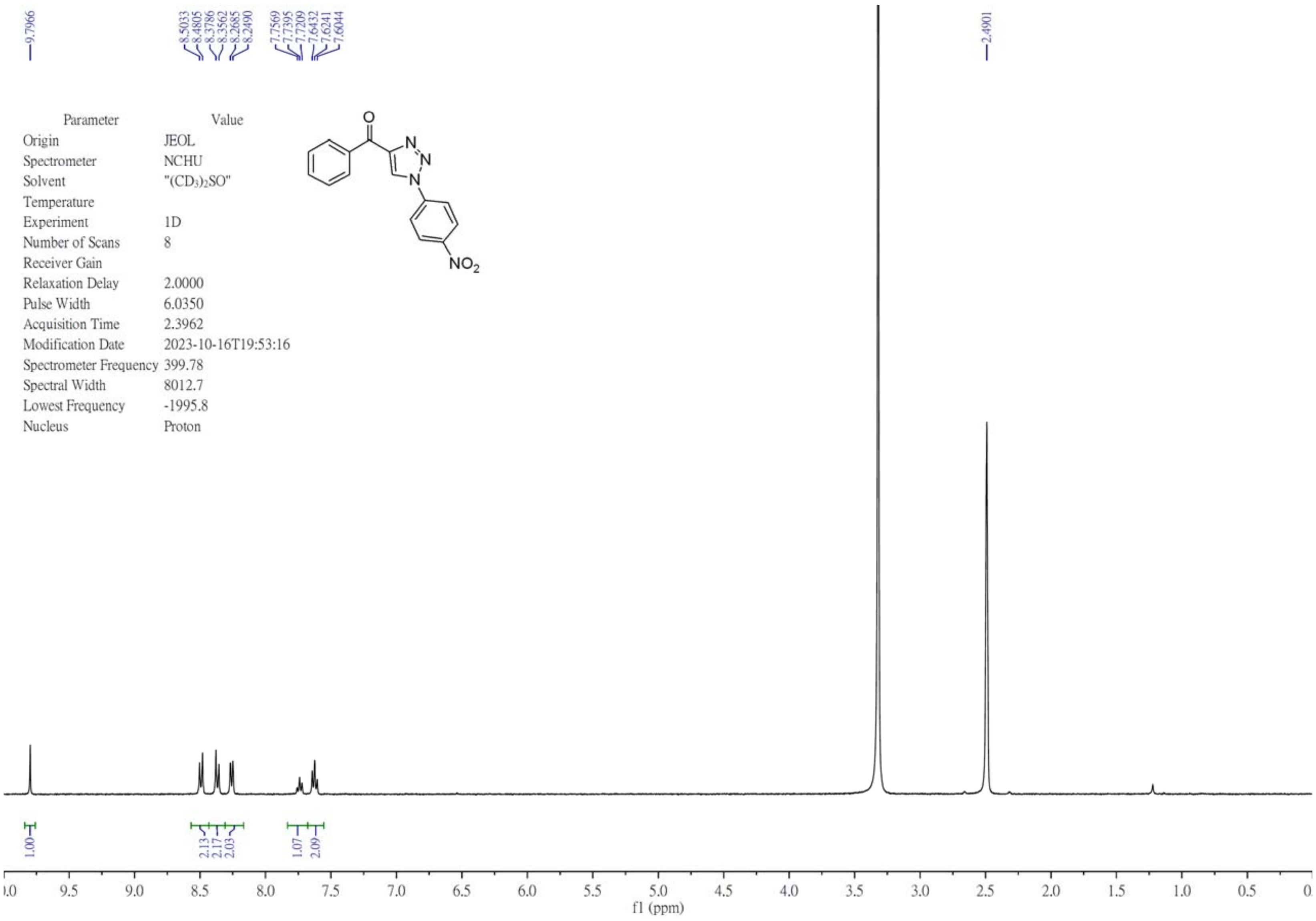

4ap ^{1}H NMR spectrum (400 MHz in $(CD_3)_2SO$)

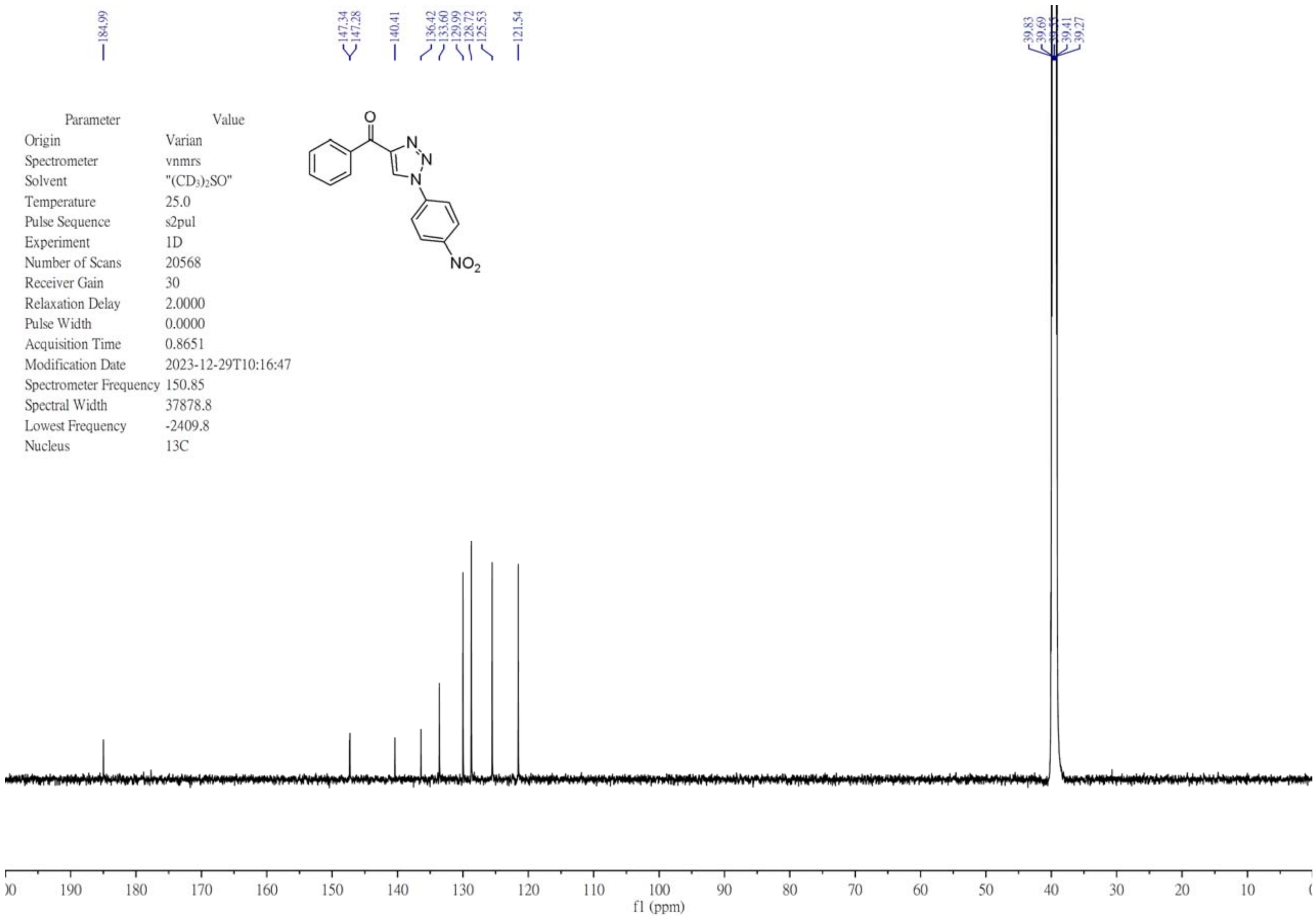

4ap $^{13}C\{^{1}H\}$ NMR spectrum (100 MHz in $(CD_3)_2SO$)

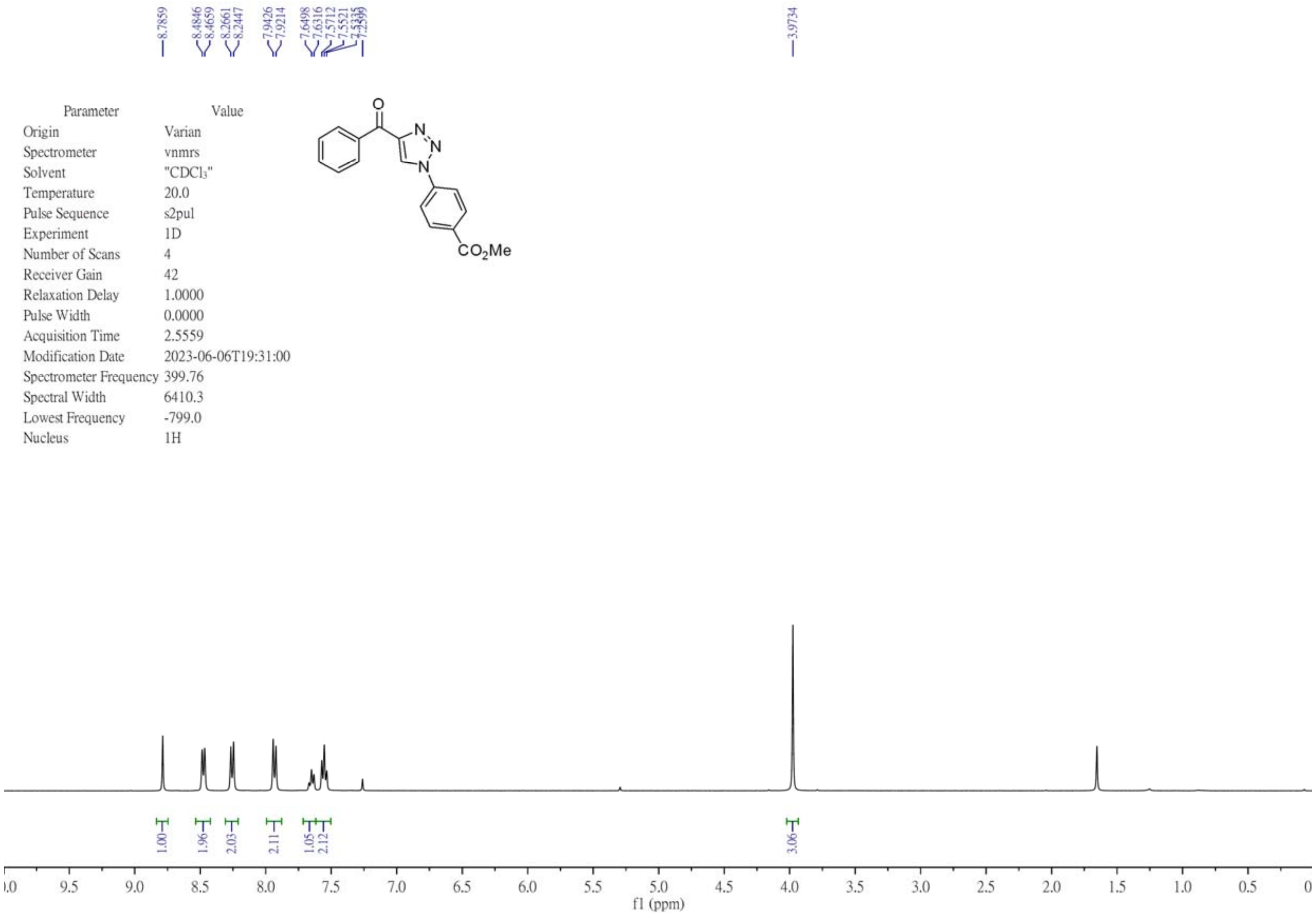

4aq ^{1}H NMR spectrum (400 MHz in $CDCl_3$)

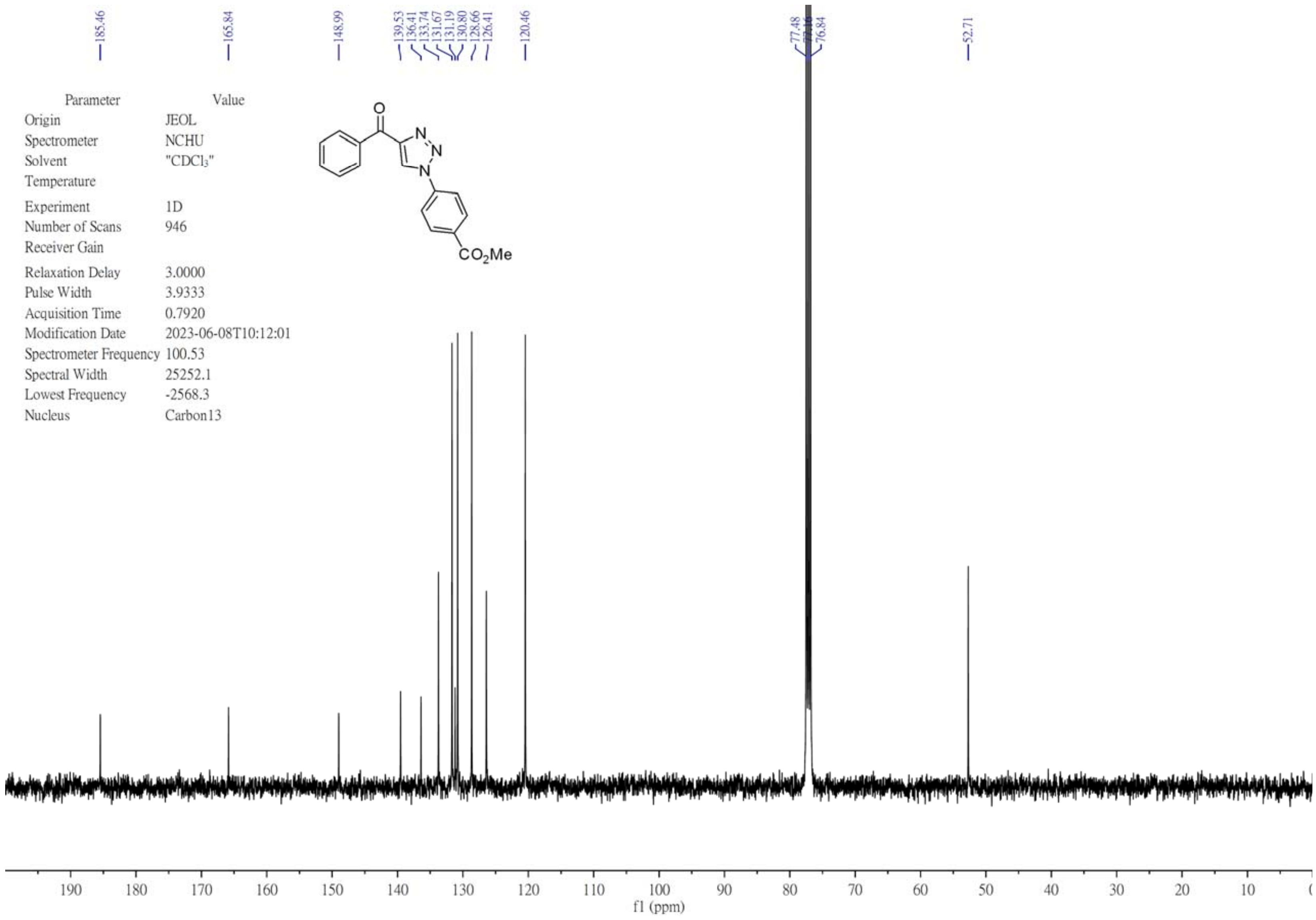

4aq $^{13}C\{^{1}H\}$ NMR spectrum (100 MHz in $CDCl_3$)

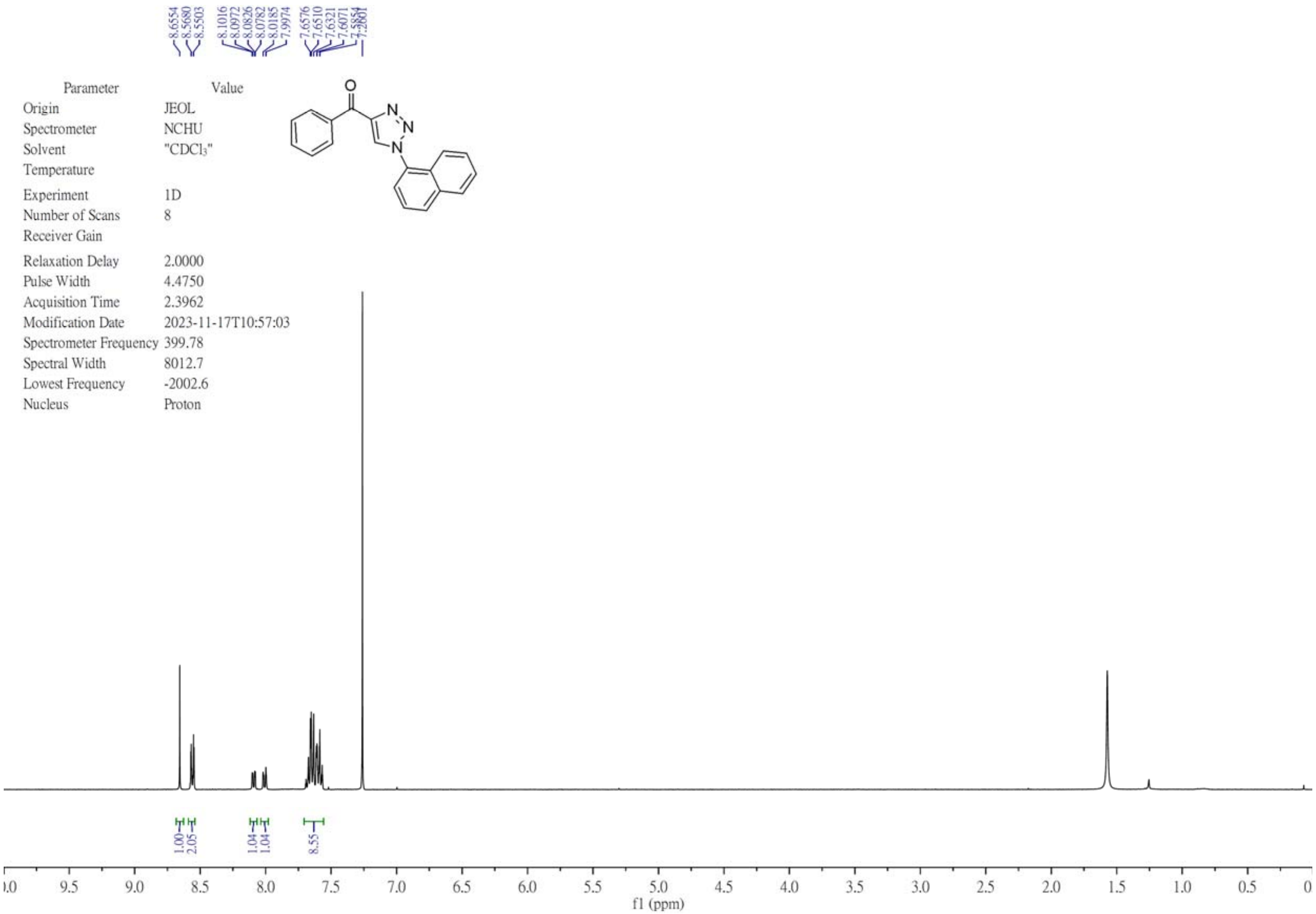

4ar ^{1}H NMR spectrum (400 MHz in $CDCl_3$)

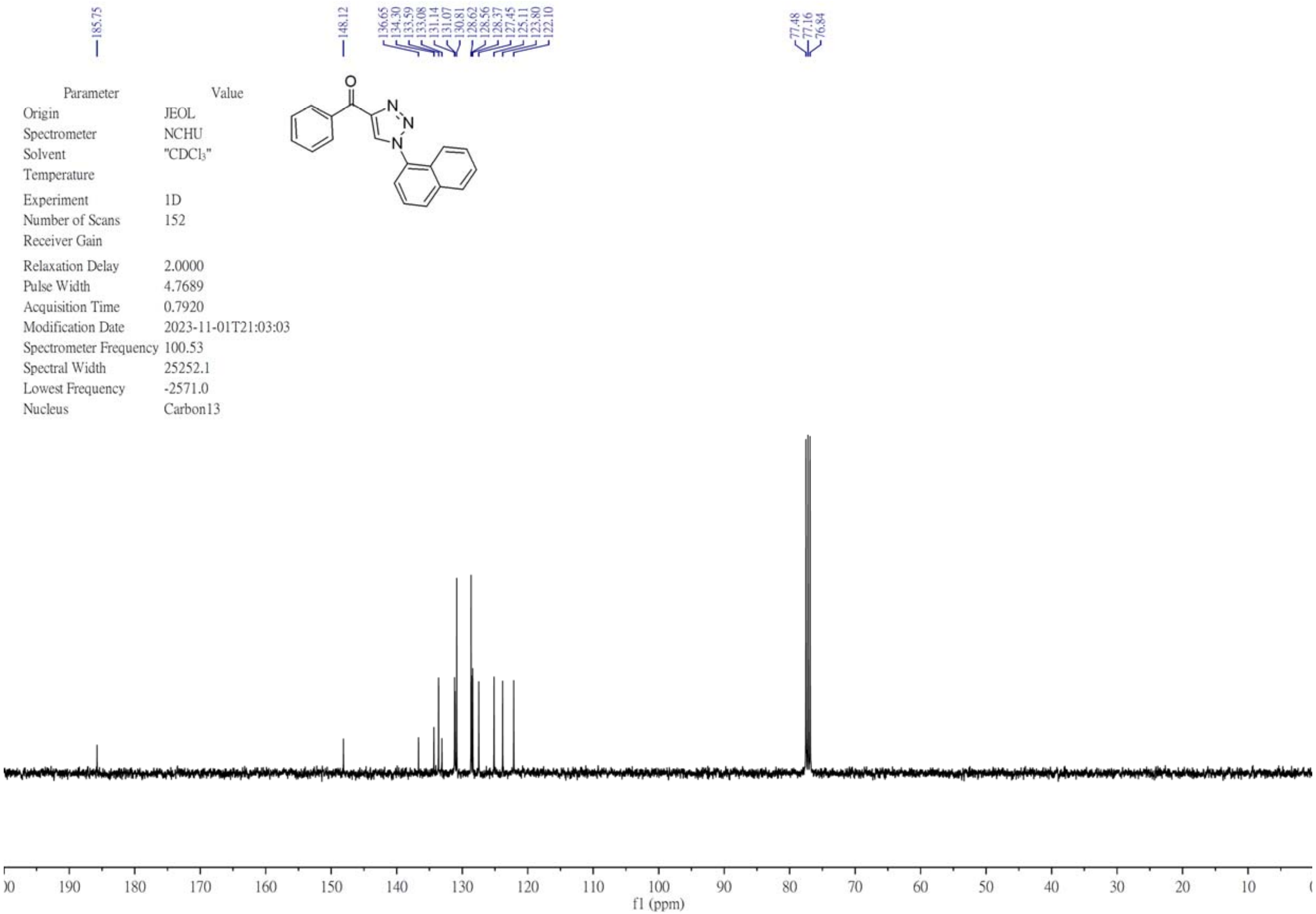

4ar $^{13}C\{^{1}H\}$ NMR spectrum (100 MHz in $CDCl_3$)

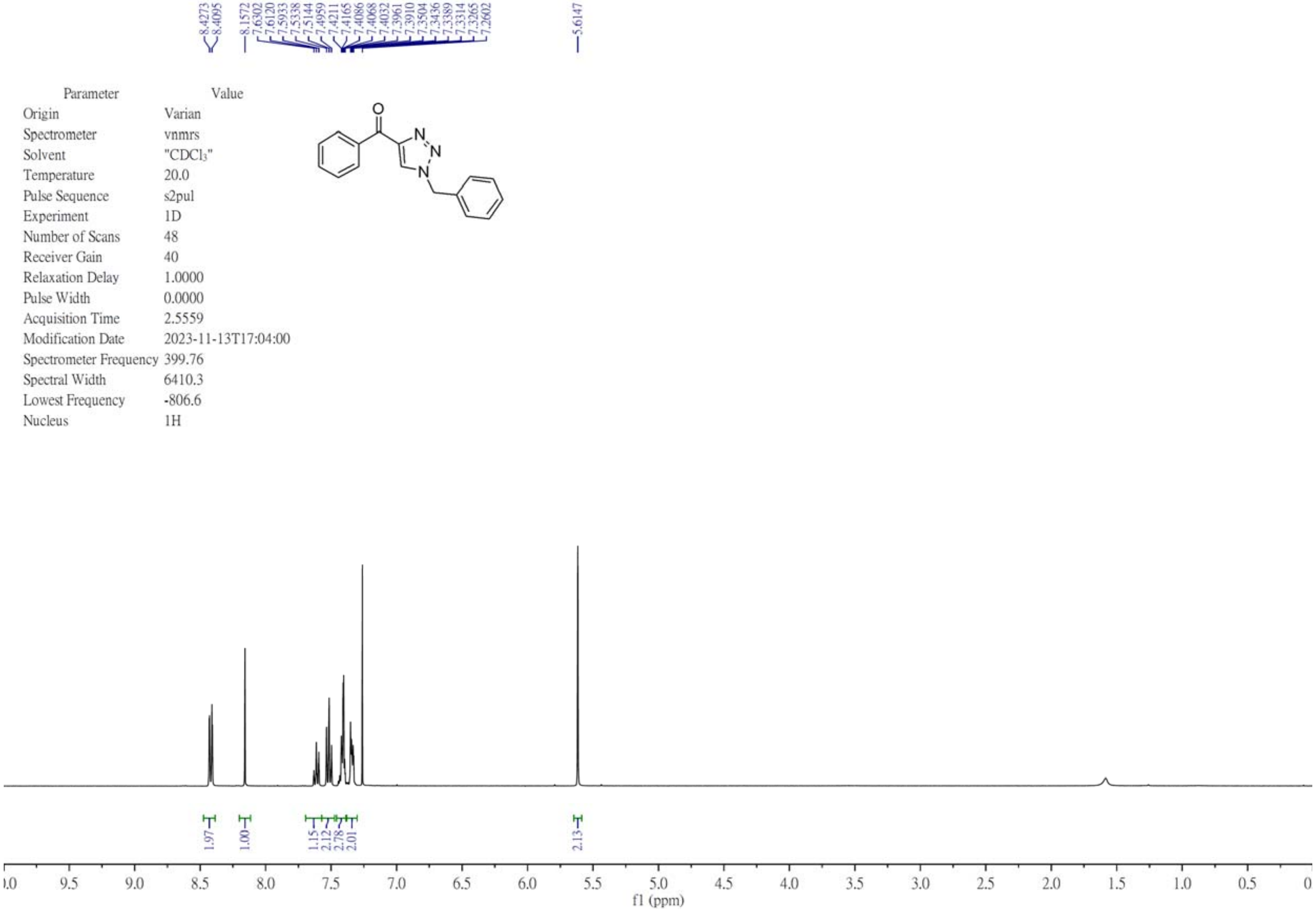

4as ^{1}H NMR spectrum (400 MHz in $CDCl_3$)

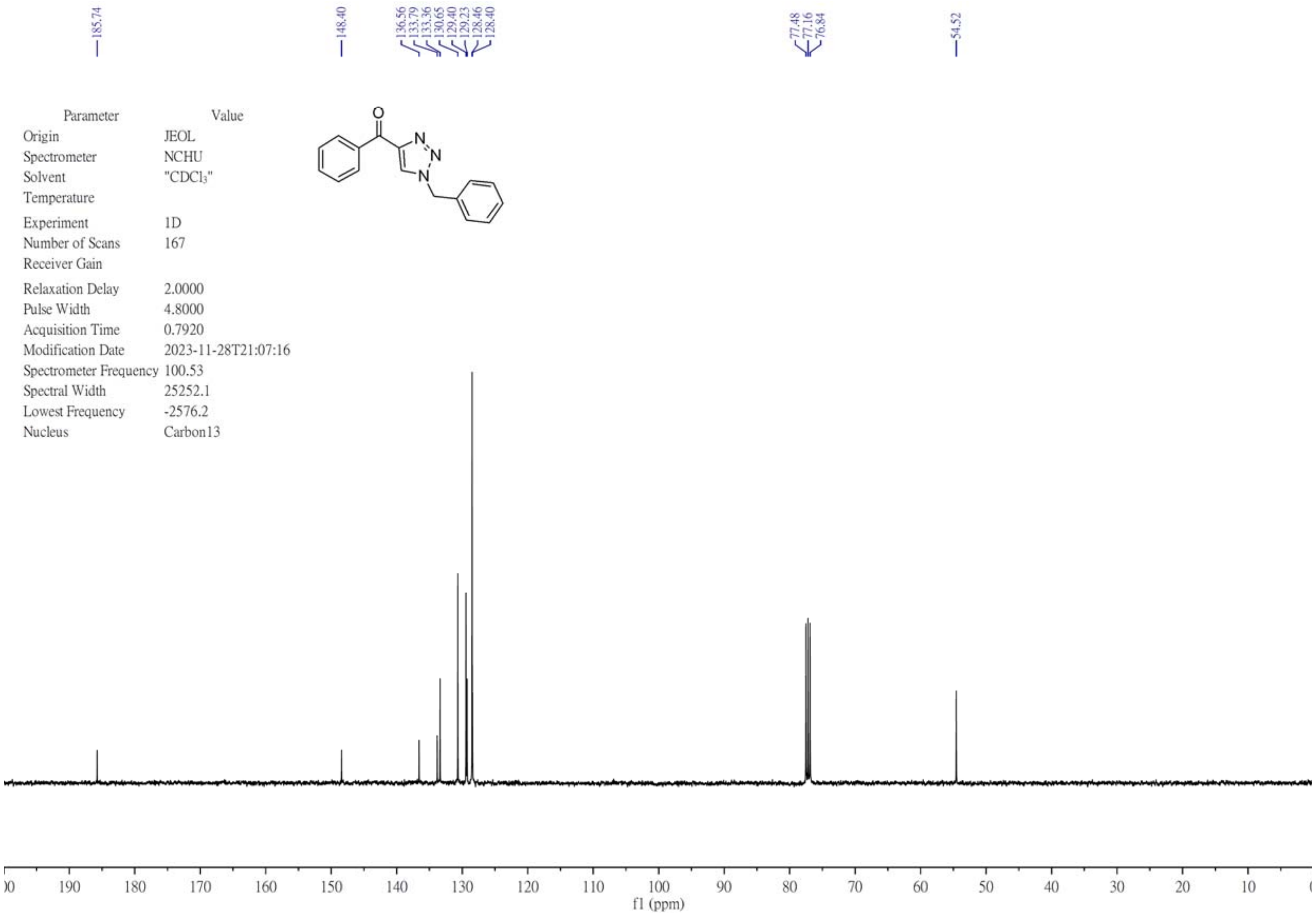

4as $^{13}C\{^{1}H\}$ NMR spectrum (100 MHz in $CDCl_3$)

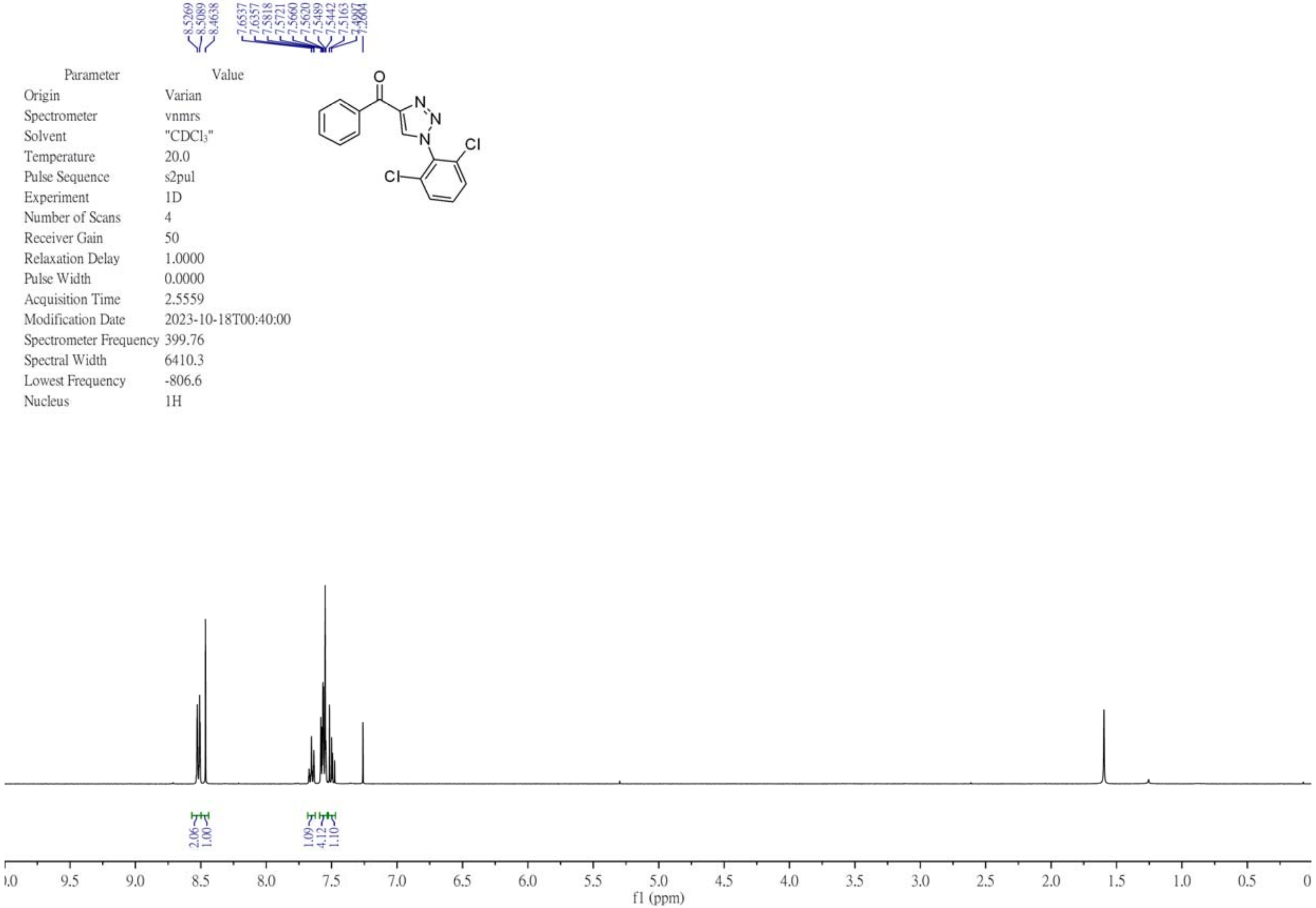

4at ^{1}H NMR spectrum (400 MHz in $CDCl_3$)

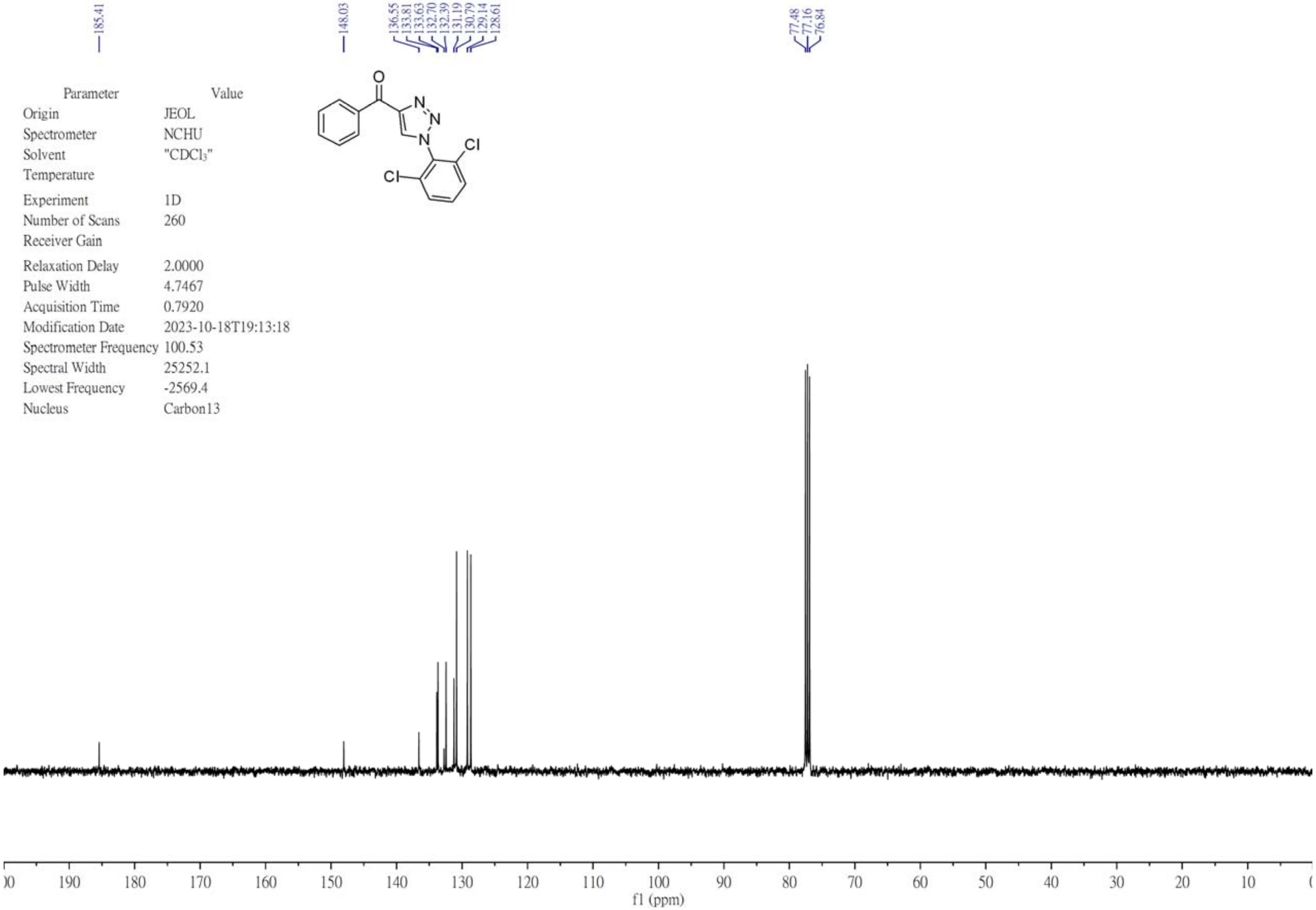

4at $^{13}C\{^{1}H\}$ NMR spectrum (100 MHz in $CDCl_3$)

Parameter	Value
Origin	Varian
Spectrometer	vnmrs
Solvent	"CDCl3"
Temperature	25.0
Pulse Sequence	s2pul
Experiment	1D
Number of Scans	4
Receiver Gain	52
Relaxation Delay	1.0000
Pulse Width	0.0000
Acquisition Time	2.5559
Modification Date	2023-03-20T19:44:00
Spectrometer Frequency	399.76
Spectral Width	6410.3
Lowest Frequency	-799.6
Nucleus	1H

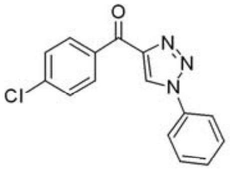

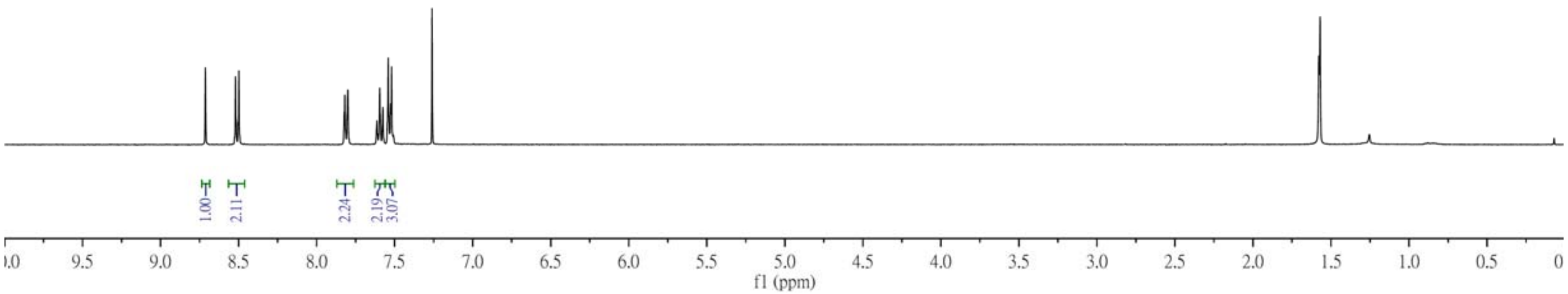

4ba ^{1}H NMR spectrum (400 MHz in $CDCl_3$)

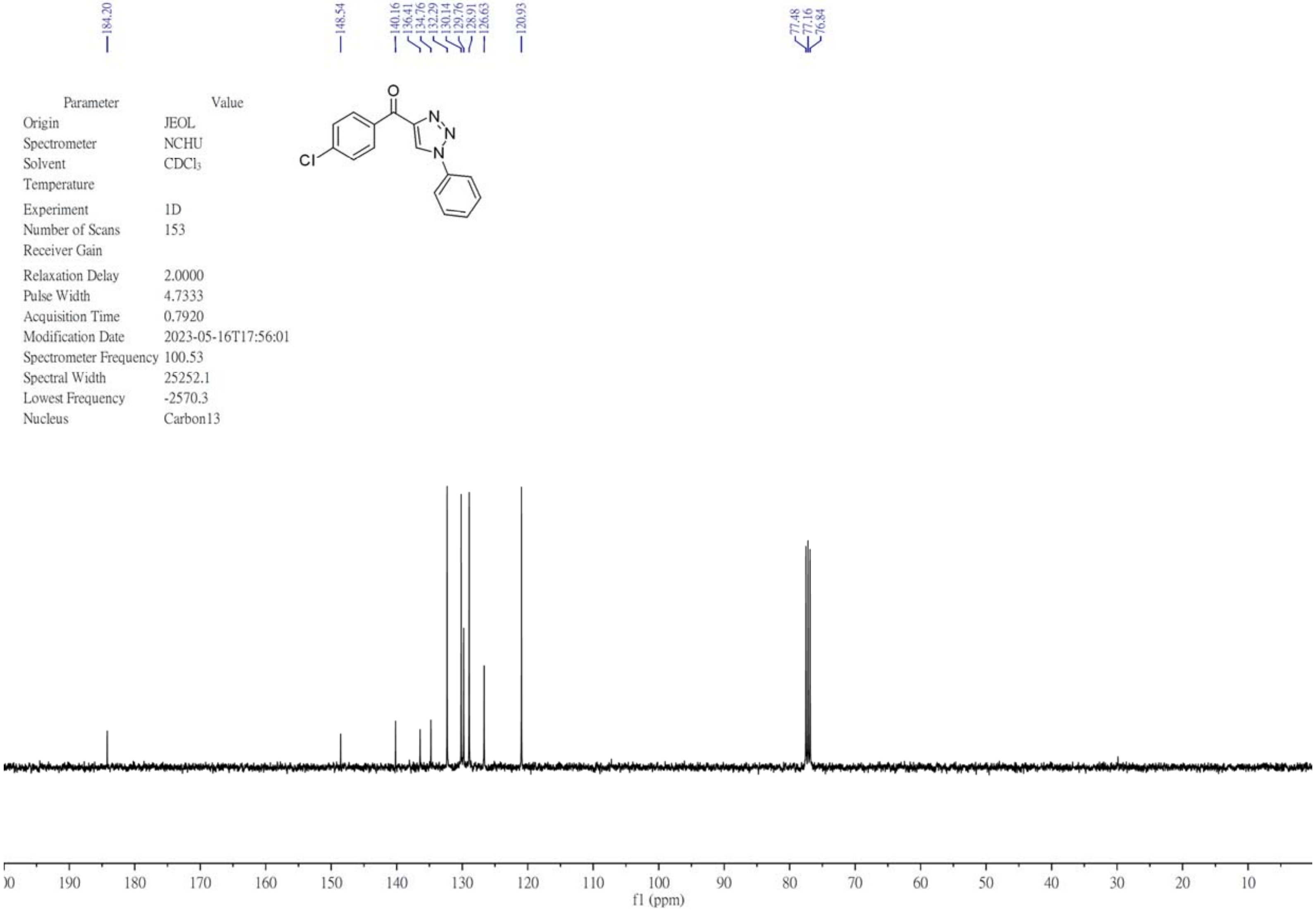

4ba $^{13}C\{^{1}H\}$ NMR spectrum (100 MHz in $CDCl_3$)

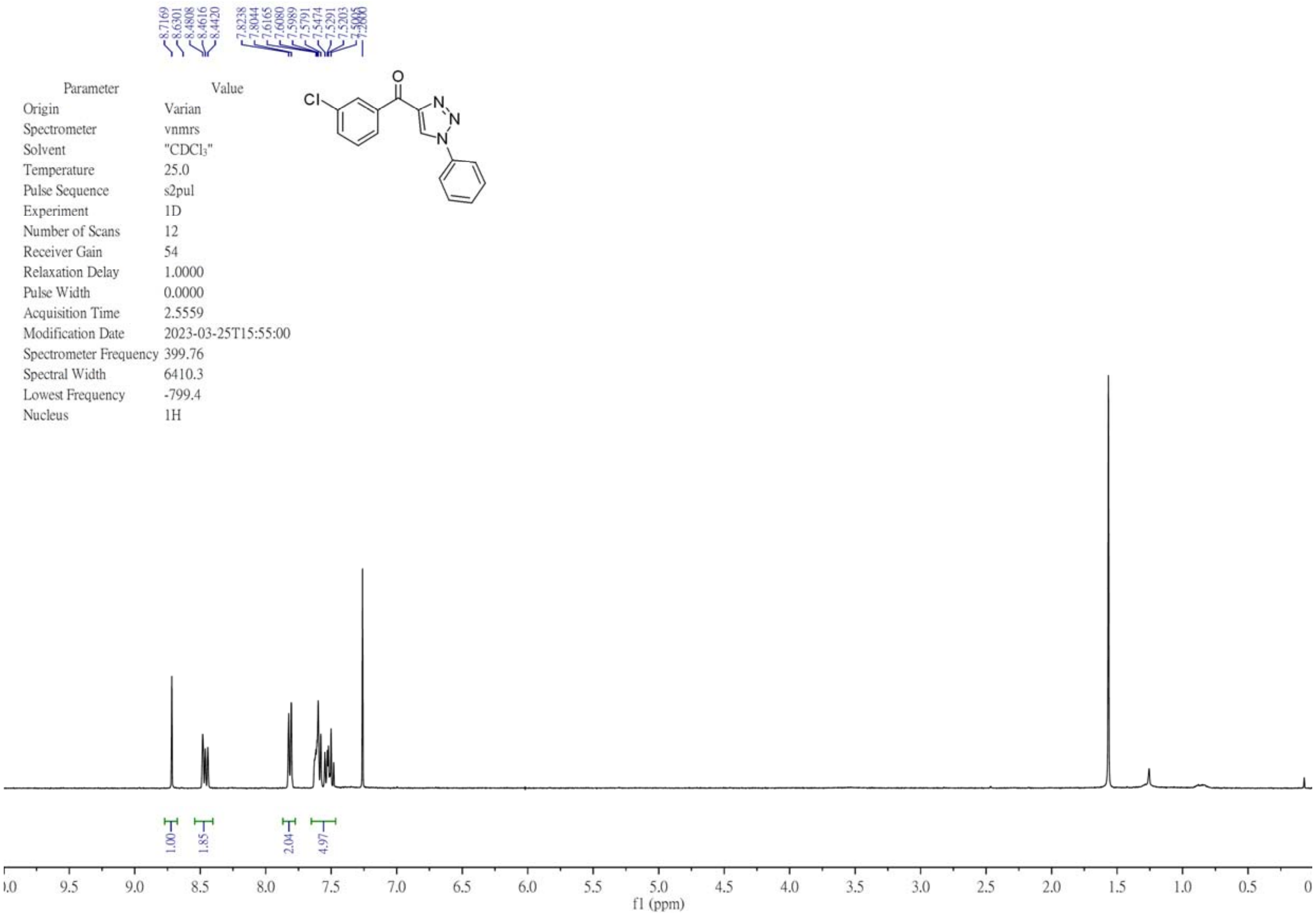

4ca ^{1}H NMR spectrum (400 MHz in $CDCl_3$)

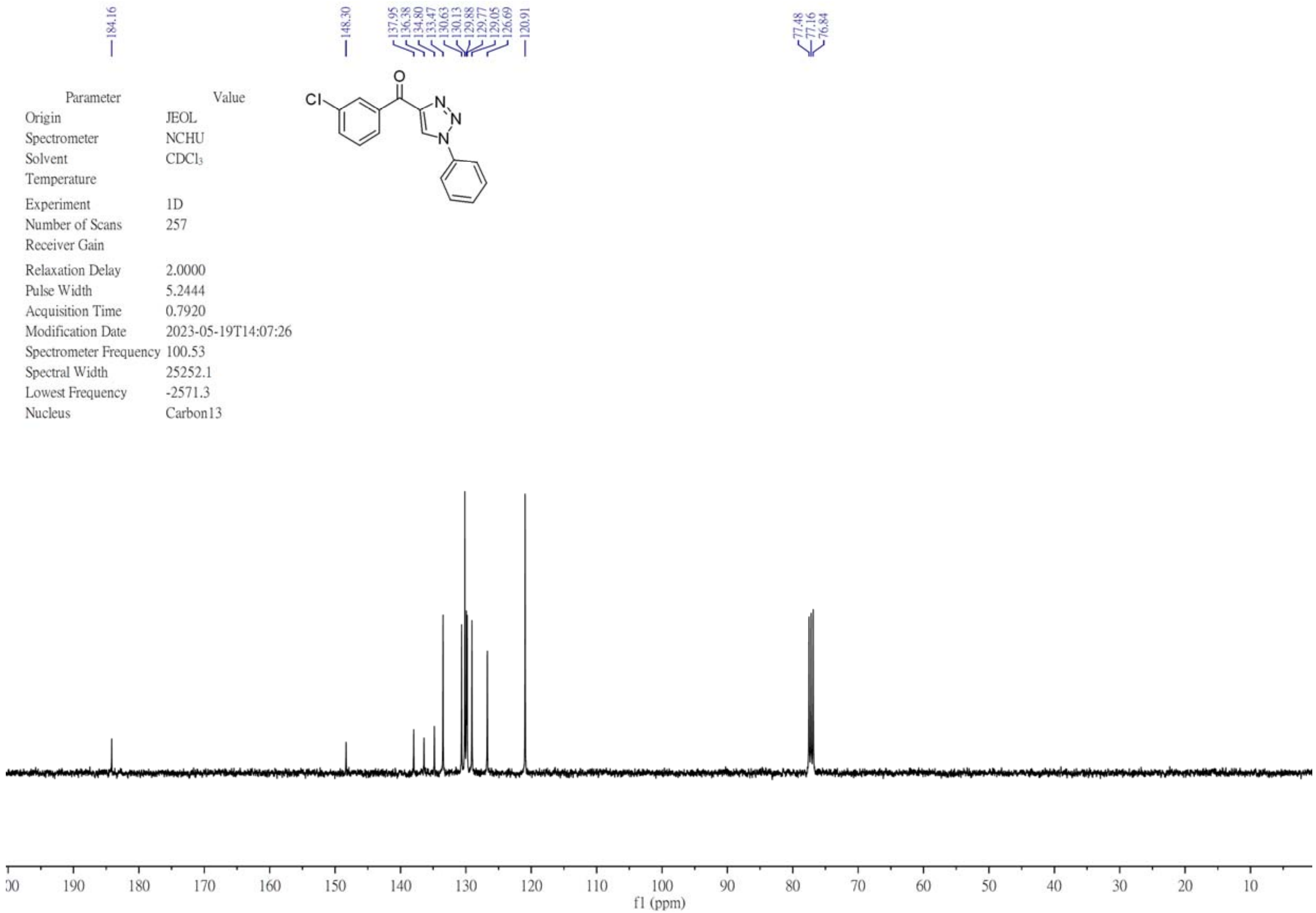

4ca $^{13}C\{^{1}H\}$ NMR spectrum (100 MHz in $CDCl_3$)

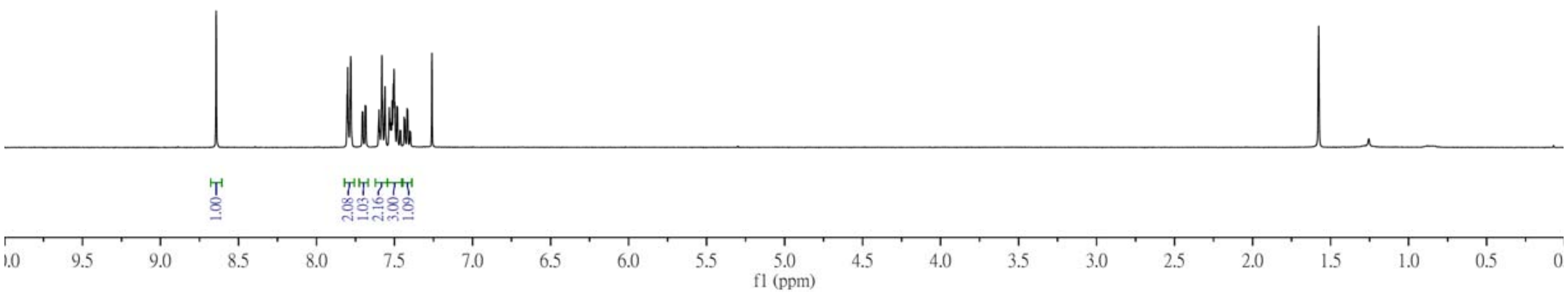

Parameter	Value
Origin	Varian
Spectrometer	vnmrs
Solvent	"$CDCl_3$"
Temperature	25.0
Pulse Sequence	s2pul
Experiment	1D
Number of Scans	4
Receiver Gain	50
Relaxation Delay	1.0000
Pulse Width	0.0000
Acquisition Time	2.5559
Modification Date	2023-03-29T21:09:00
Spectrometer Frequency	399.76
Spectral Width	6410.3
Lowest Frequency	-799.6
Nucleus	1H

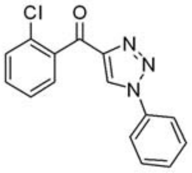

4da ^{1}H NMR spectrum (400 MHz in $CDCl_3$)

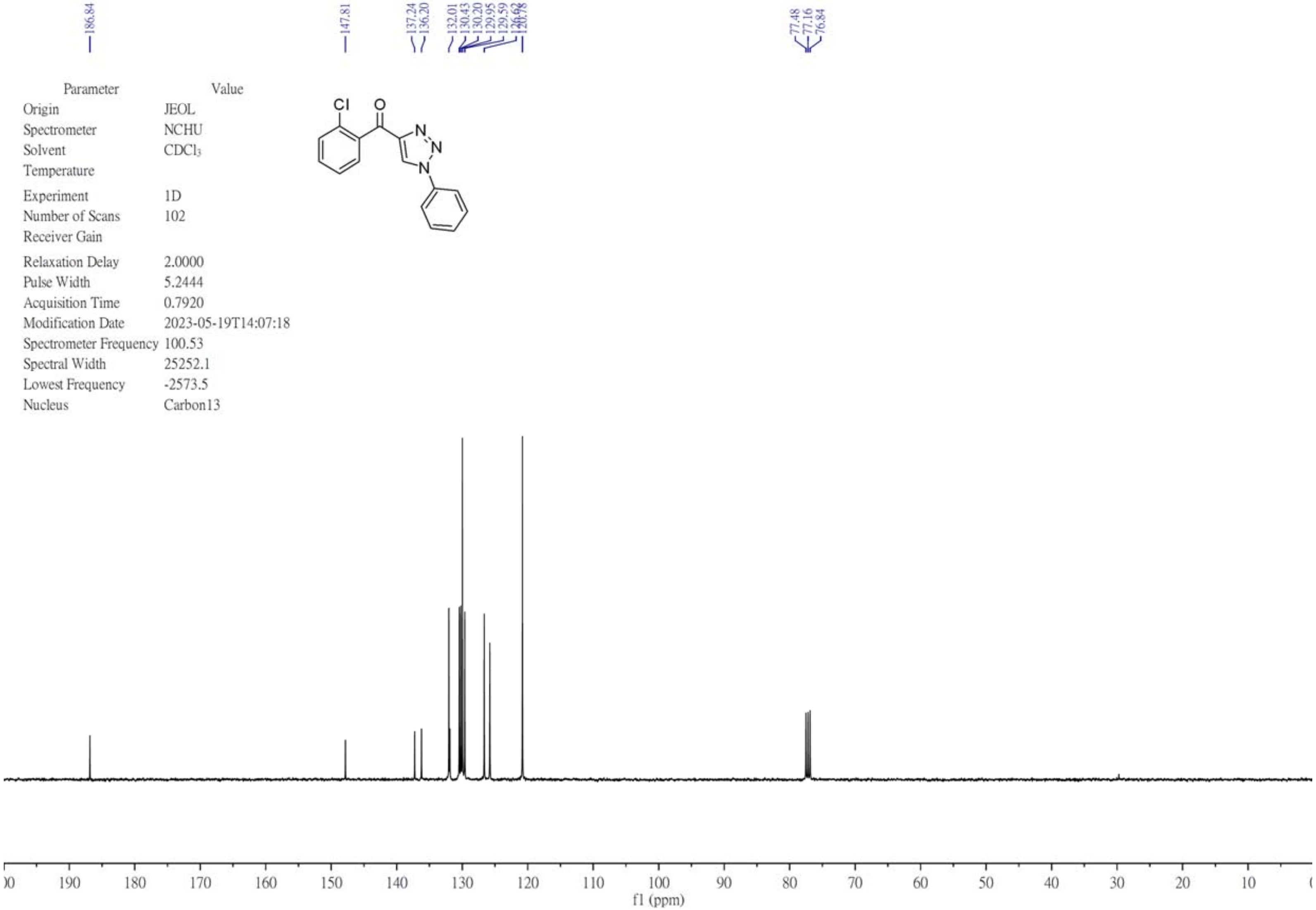

4da $^{13}C\{^{1}H\}$ NMR spectrum (100 MHz in $CDCl_3$)

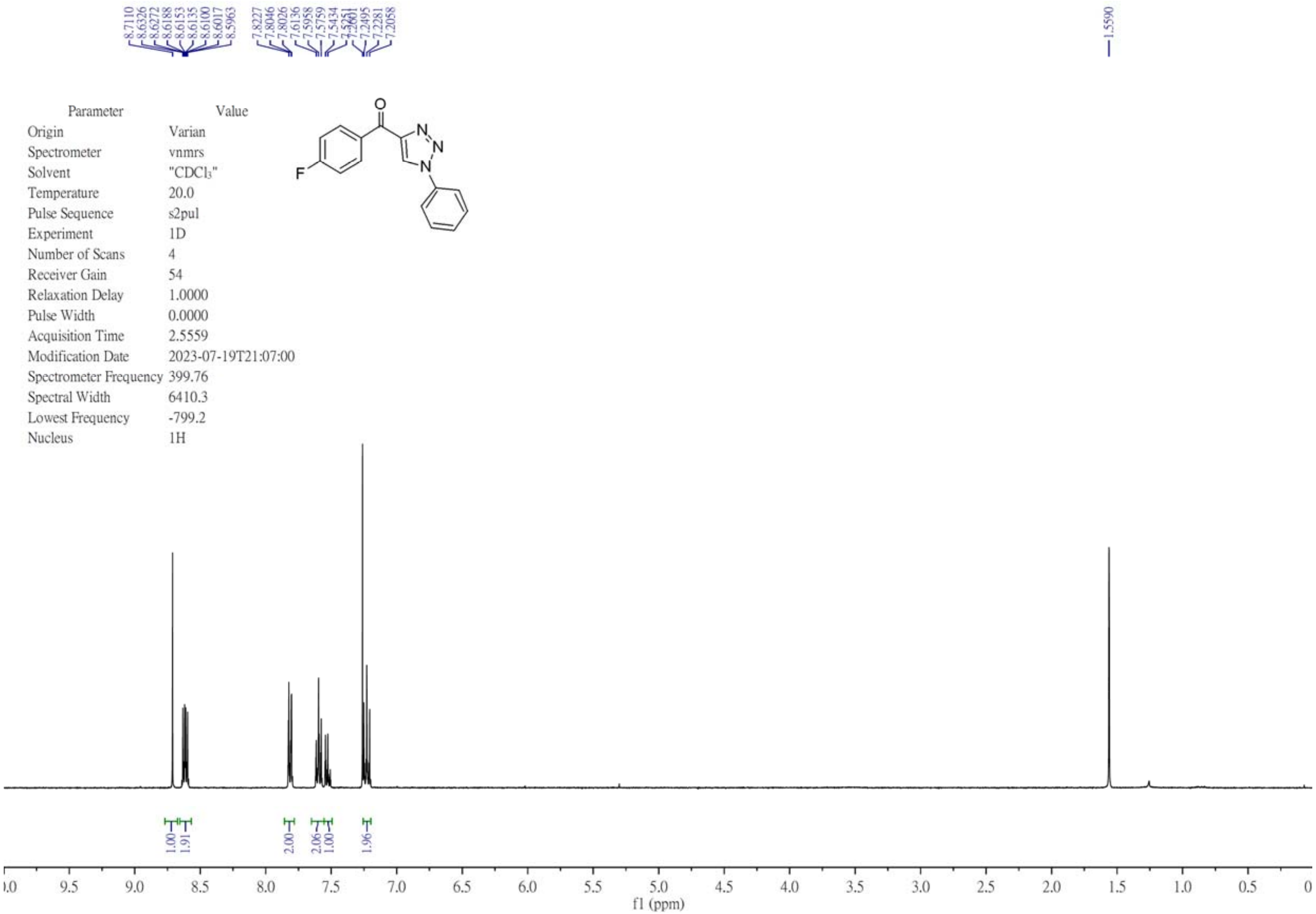

4ea ^{1}H NMR spectrum (400 MHz in $CDCl_3$)

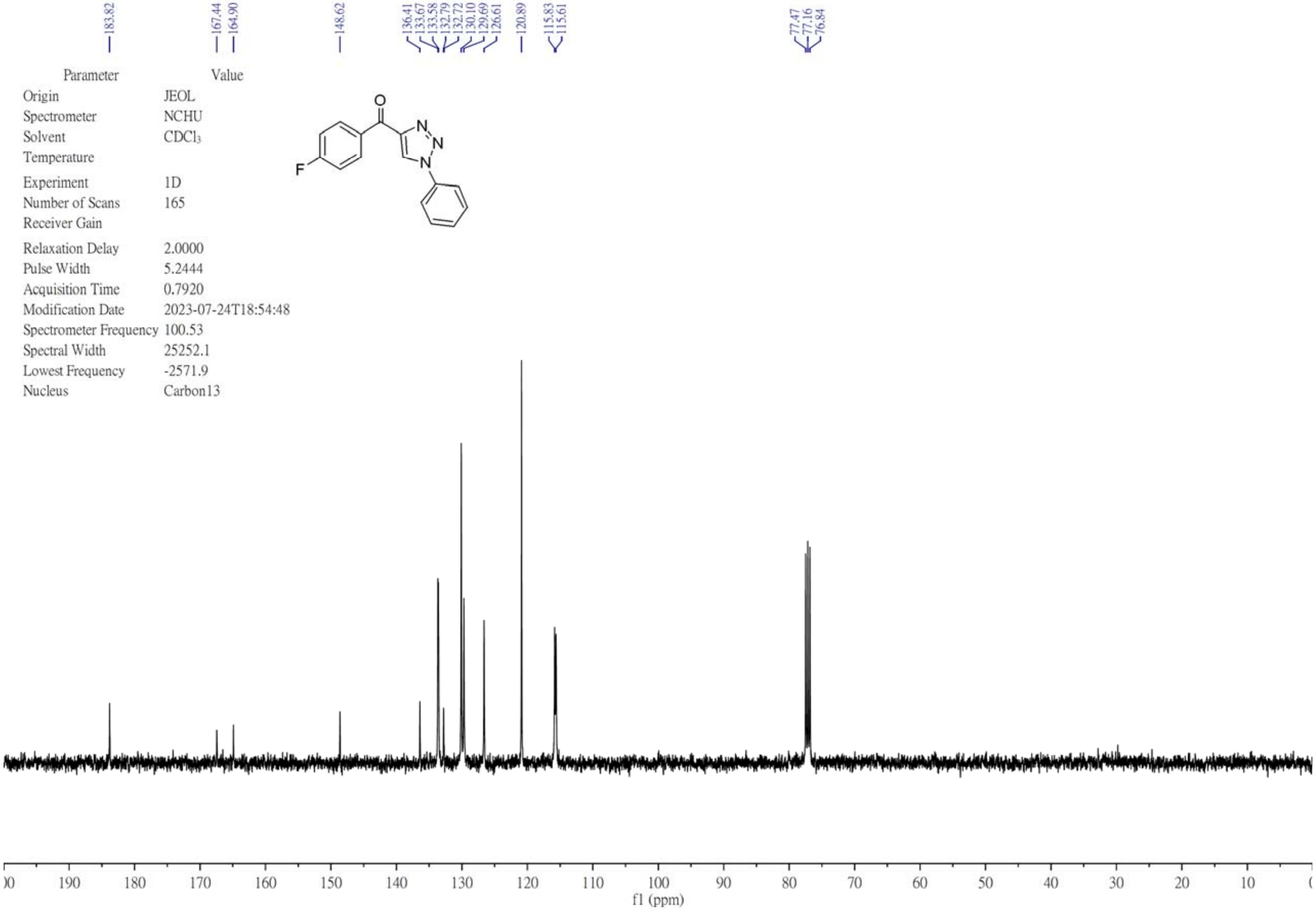

4ea $^{13}C\{^{1}H\}$ NMR spectrum (100 MHz in $CDCl_3$)

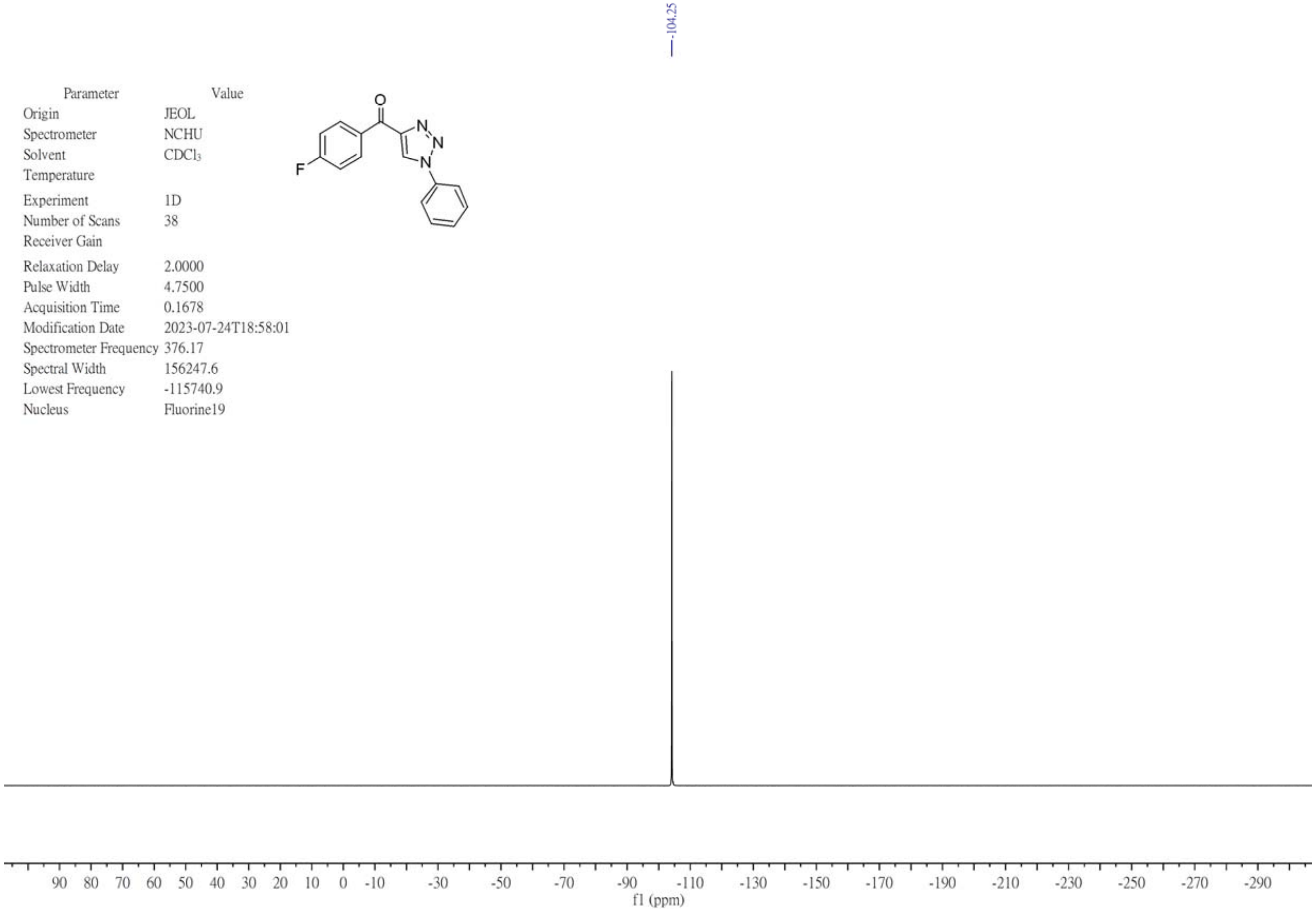

4ea ^{19}F NMR spectrum (376 MHz in ($CDCl_3$)

Parameter	Value
Origin	JEOL
Spectrometer	NCHU
Solvent	"(CD3)2SO"
Temperature	
Experiment	1D
Number of Scans	8
Receiver Gain	
Relaxation Delay	2.0000
Pulse Width	4.4750
Acquisition Time	2.3962
Modification Date	2023-11-13T15:19:32
Spectrometer Frequency	399.78
Spectral Width	8012.7
Lowest Frequency	-1992.2
Nucleus	Proton

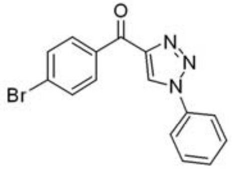

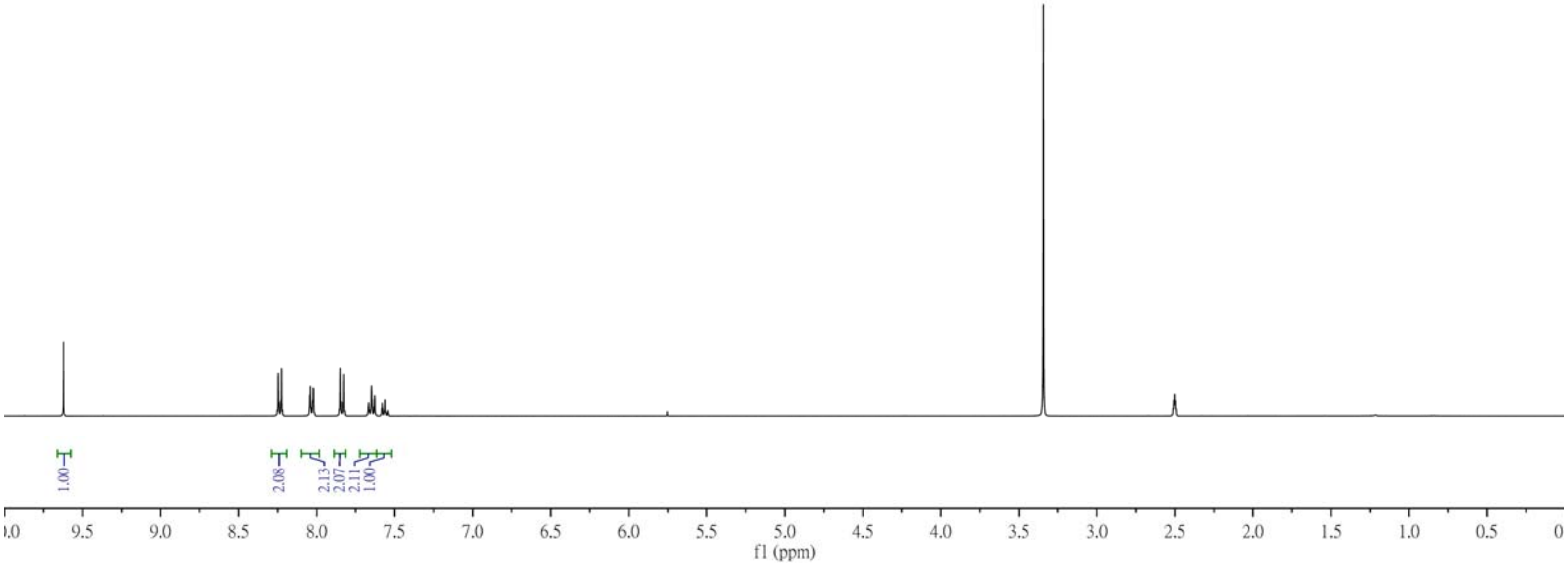

4fa ^{1}H NMR spectrum (400 MHz in $(CD_3)_2SO$)

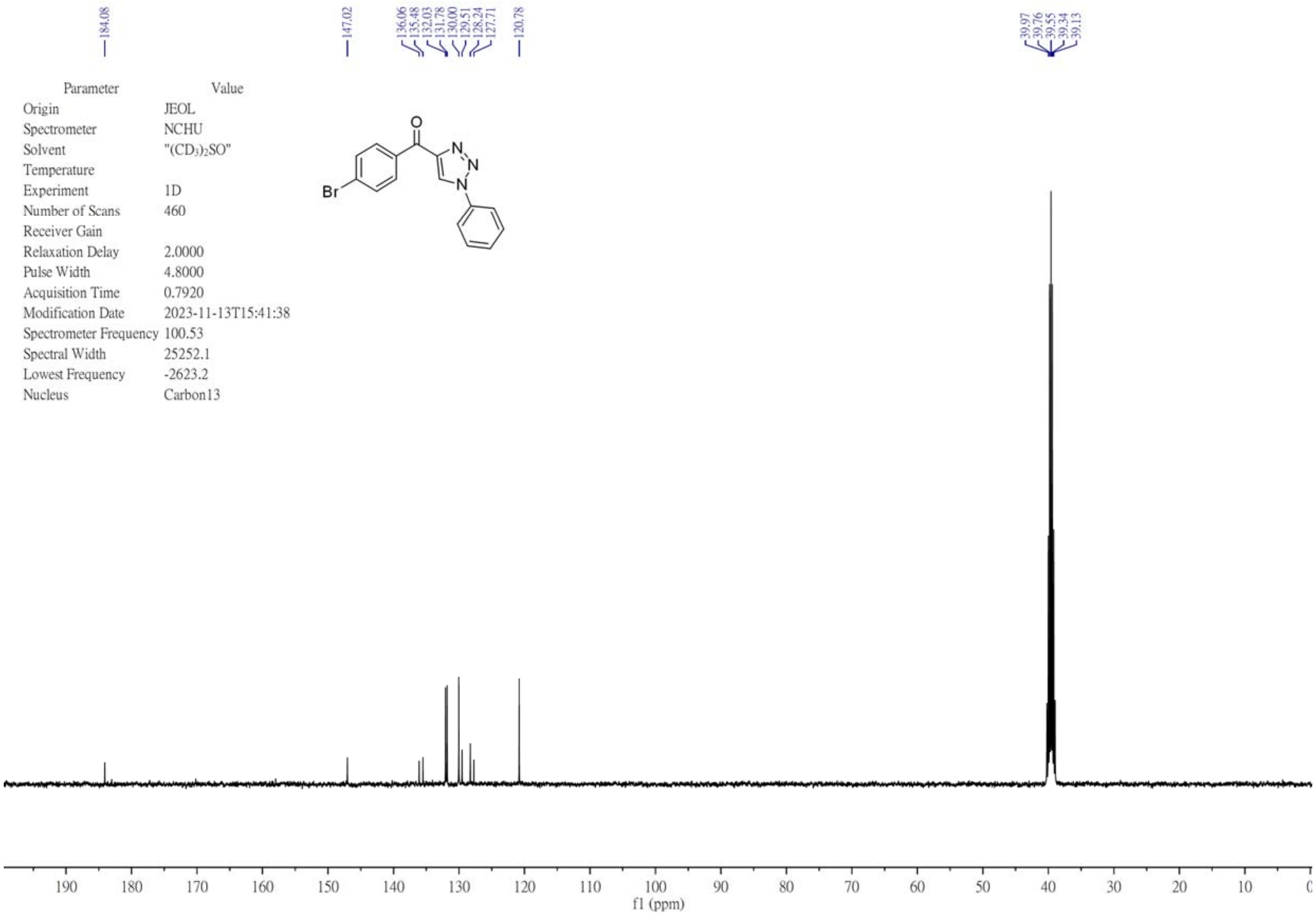

4fa $^{13}C\{^1H\}$ NMR spectrum (100 MHz in $(CD_3)_2SO$)

Parameter	Value
Origin	Varian
Spectrometer	vnmrs
Solvent	"$CDCl_3$"
Temperature	20.0
Pulse Sequence	s2pul
Experiment	1D
Number of Scans	20
Receiver Gain	56
Relaxation Delay	1.0000
Pulse Width	0.0000
Acquisition Time	2.5559
Modification Date	2023-07-19T21:17:00
Spectrometer Frequency	399.76
Spectral Width	6410.3
Lowest Frequency	-799.4
Nucleus	1H

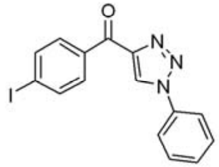

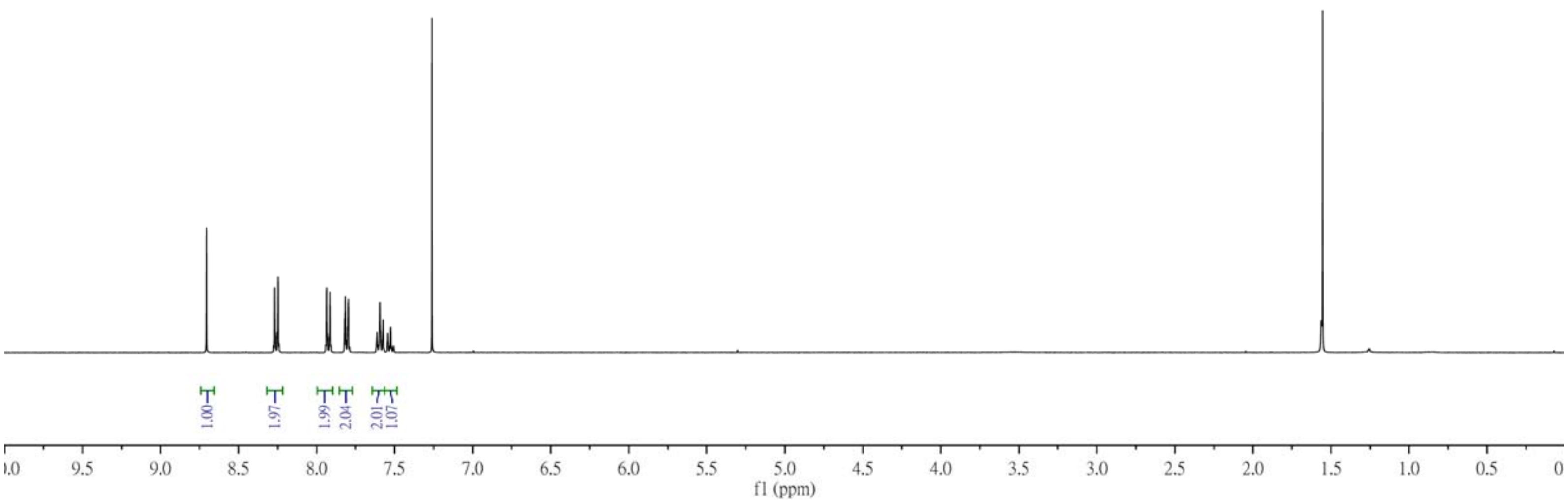

4ga ^{1}H NMR spectrum (400 MHz in $CDCl_3$)

Parameter	Value
Origin	JEOL
Spectrometer	NCHU
Solvent	"$CDCl_3$"
Temperature	
Experiment	1D
Number of Scans	102
Receiver Gain	
Relaxation Delay	2.0000
Pulse Width	5.2444
Acquisition Time	0.7920
Modification Date	2023-07-24T18:43:58
Spectrometer Frequency	100.53
Spectral Width	25252.1
Lowest Frequency	-2572.1
Nucleus	Carbon13

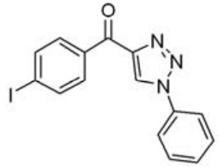

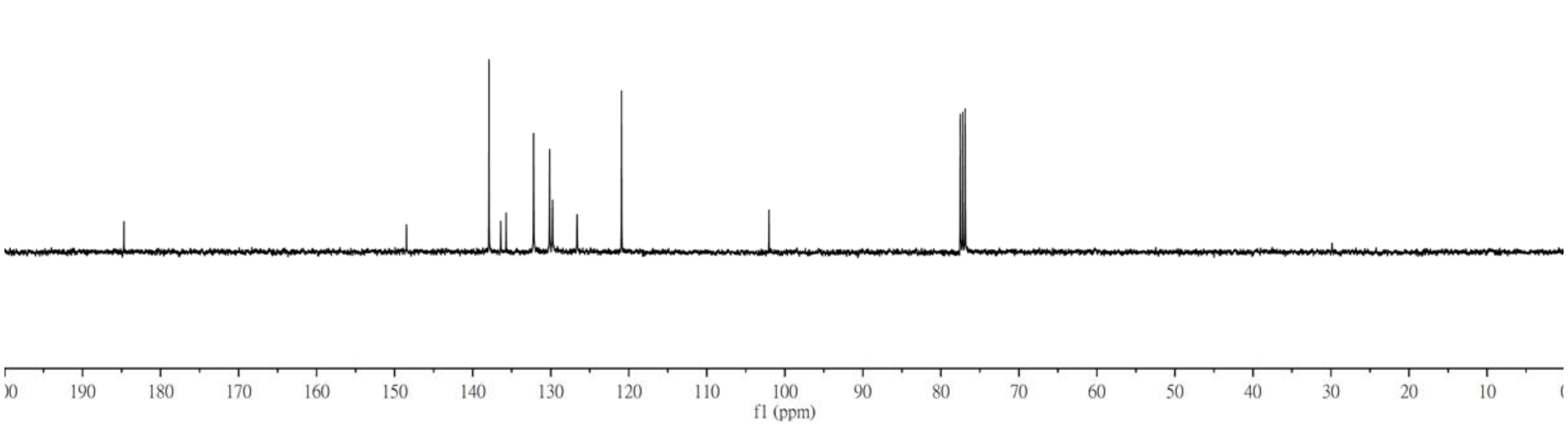

4ga $^{13}C\{^{1}H\}$ NMR spectrum (100 MHz in $CDCl_3$)

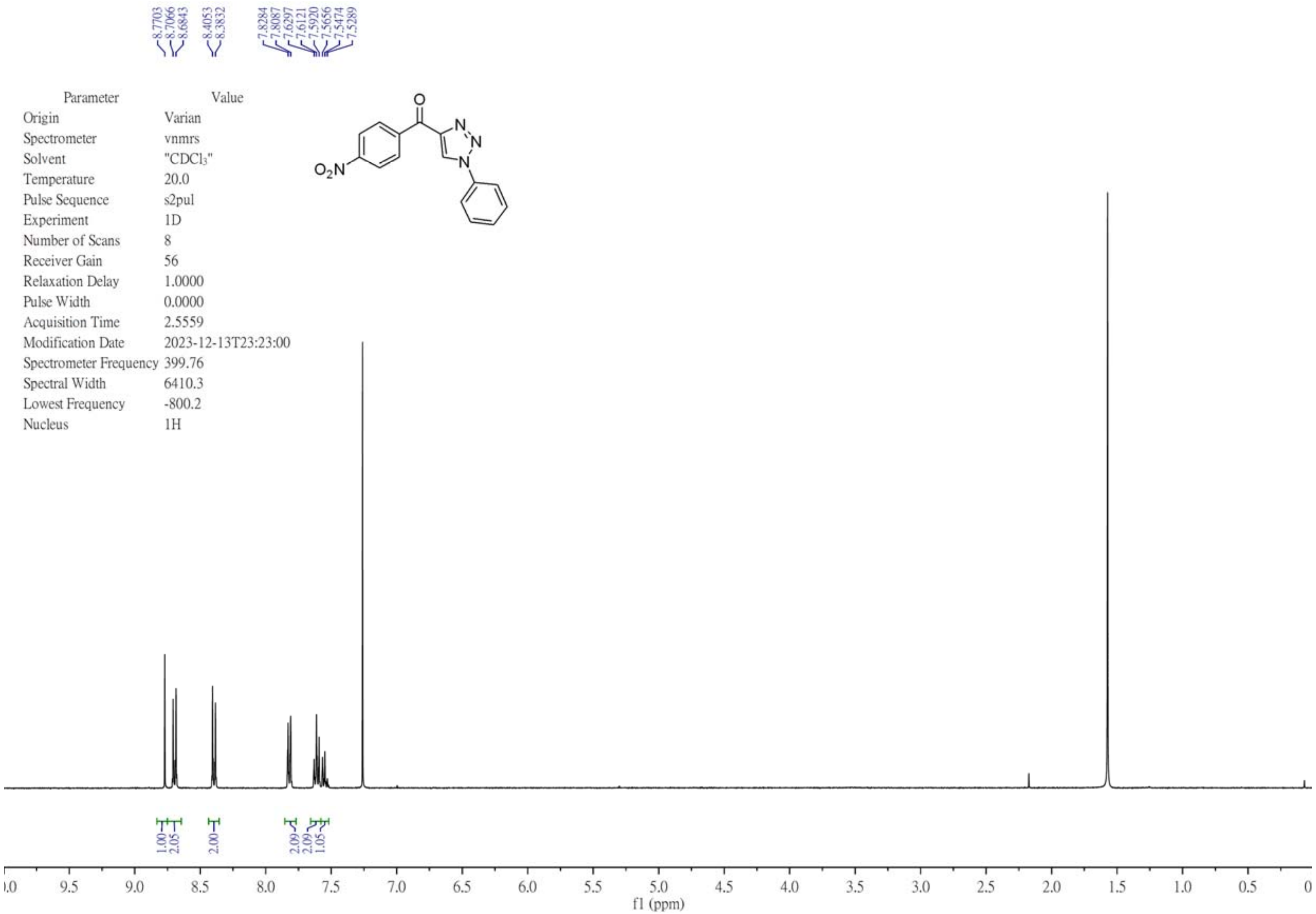

4ha ^{1}H NMR spectrum (400 MHz in $CDCl_3$)

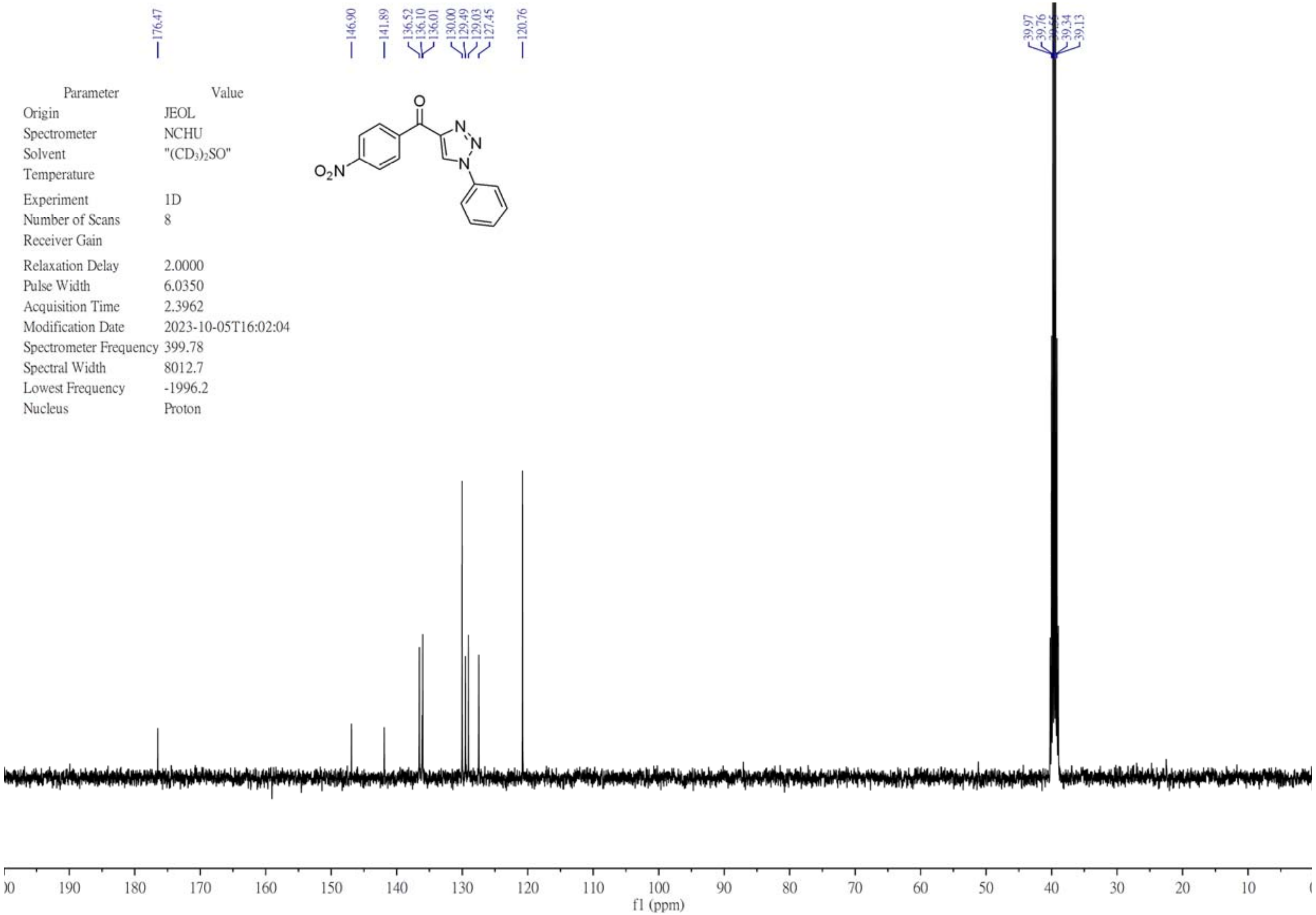

4ha $^{13}C\{^{1}H\}$ NMR spectrum (100 MHz in $(CD_3)_2SO$)

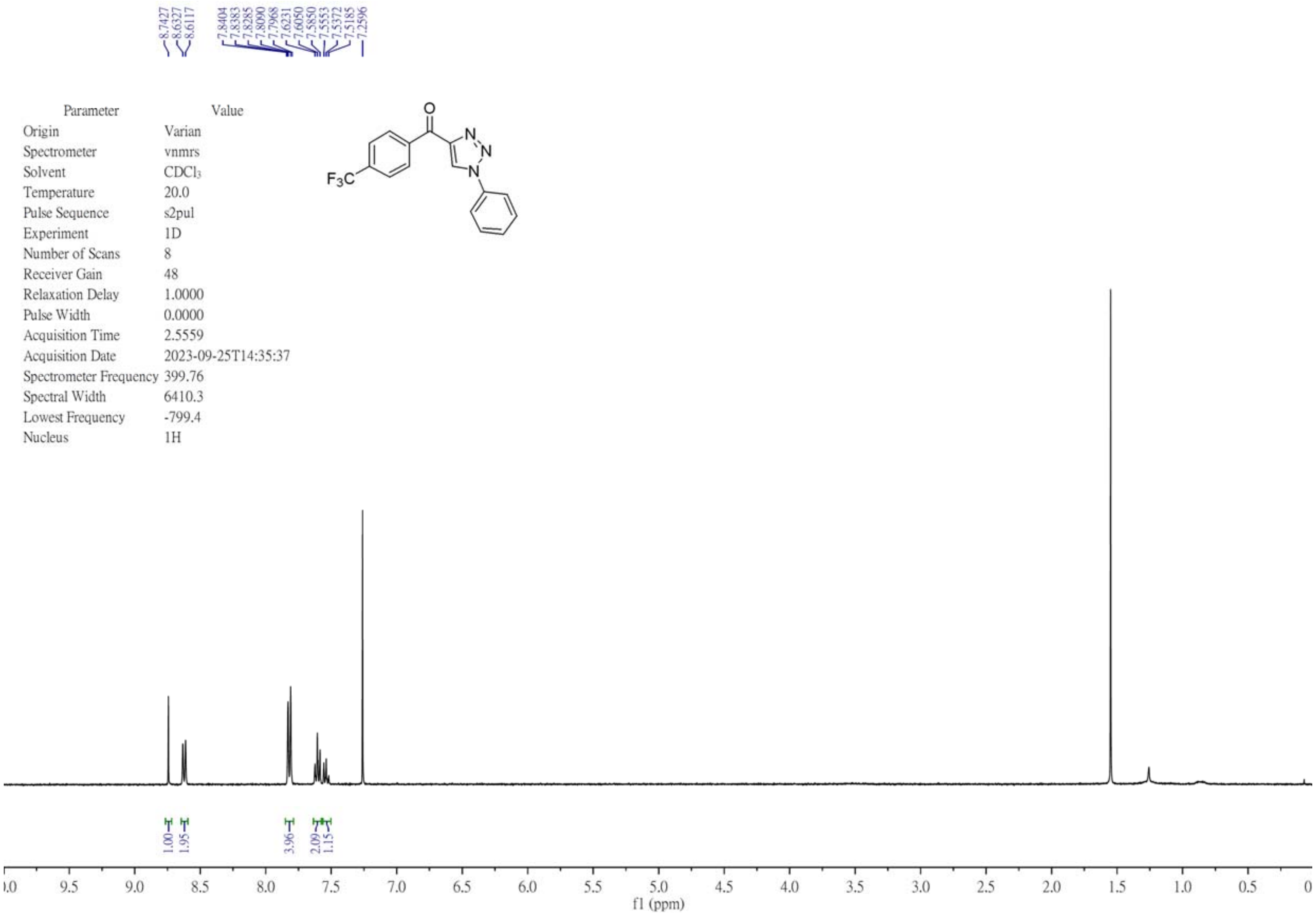

4ia ^{1}H NMR spectrum (400 MHz in $CDCl_3$)

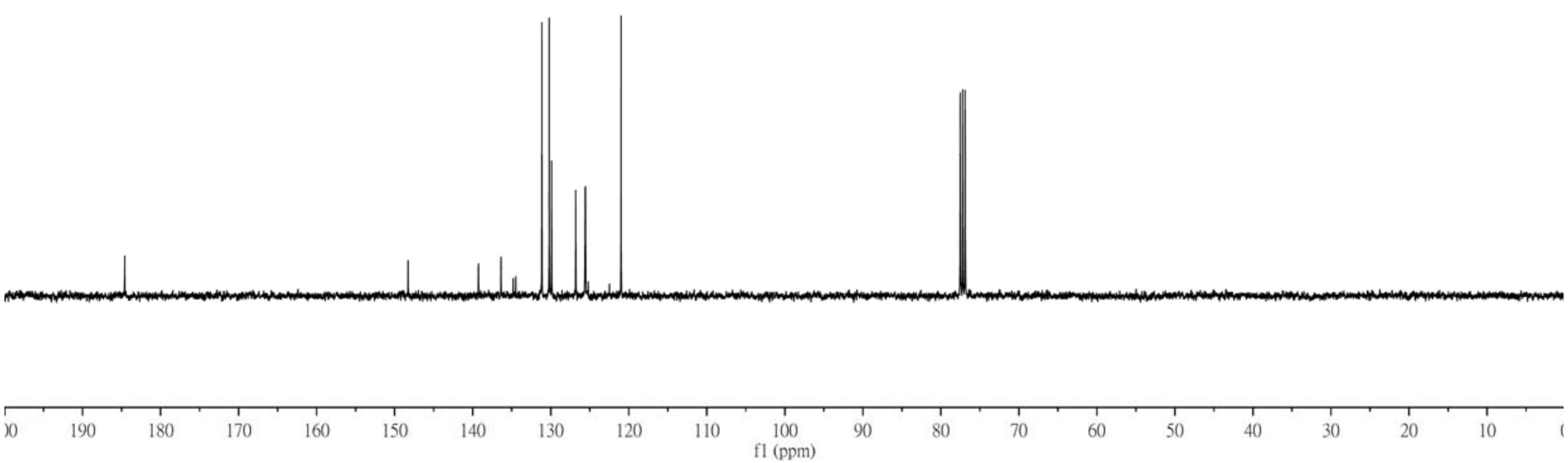

Parameter	Value
Origin	JEOL
Spectrometer	NCHU
Solvent	$CDCl_3$
Temperature	
Experiment	1D
Number of Scans	94
Receiver Gain	
Relaxation Delay	2.0000
Pulse Width	4.7467
Acquisition Time	0.7920
Modification Date	2023-10-05T15:08:30
Spectrometer Frequency	100.53
Spectral Width	25252.1
Lowest Frequency	-2569.6
Nucleus	Carbon13

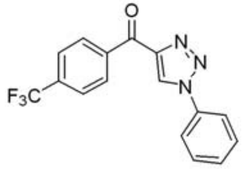

4ia $^{13}C\{^1H\}$ NMR spectrum (100 MHz in $CDCl_3$)

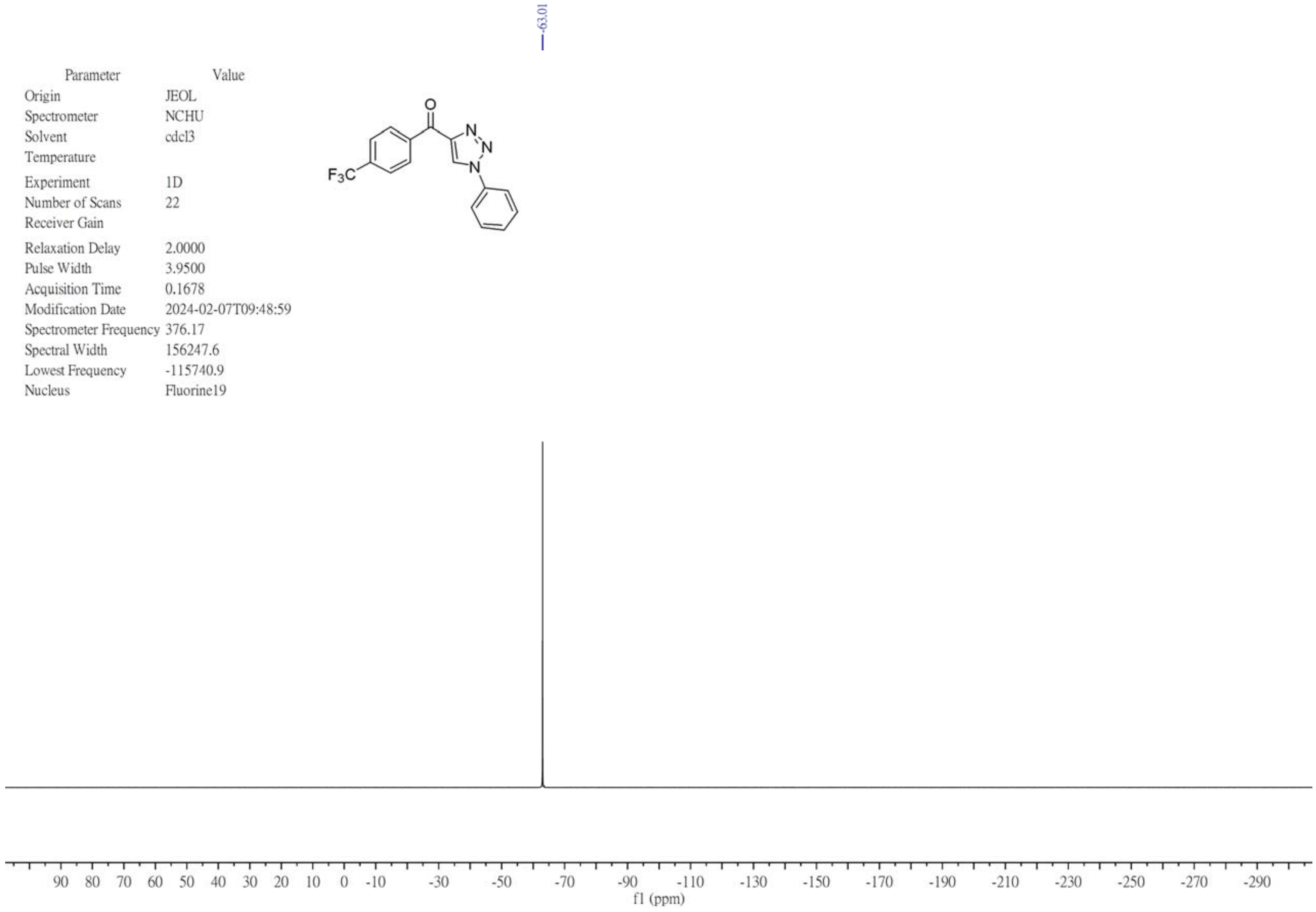

4ia ^{19}F NMR spectrum (376 MHz in $CDCl_3$)

Parameter	Value
Origin	Varian
Spectrometer	vnmrs
Solvent	"CDCl3"
Temperature	25.0
Pulse Sequence	s2pul
Experiment	1D
Number of Scans	8
Receiver Gain	48
Relaxation Delay	1.0000
Pulse Width	0.0000
Acquisition Time	2.5559
Modification Date	2023-03-25T15:50:00
Spectrometer Frequency	399.76
Spectral Width	6410.3
Lowest Frequency	-799.4
Nucleus	1H

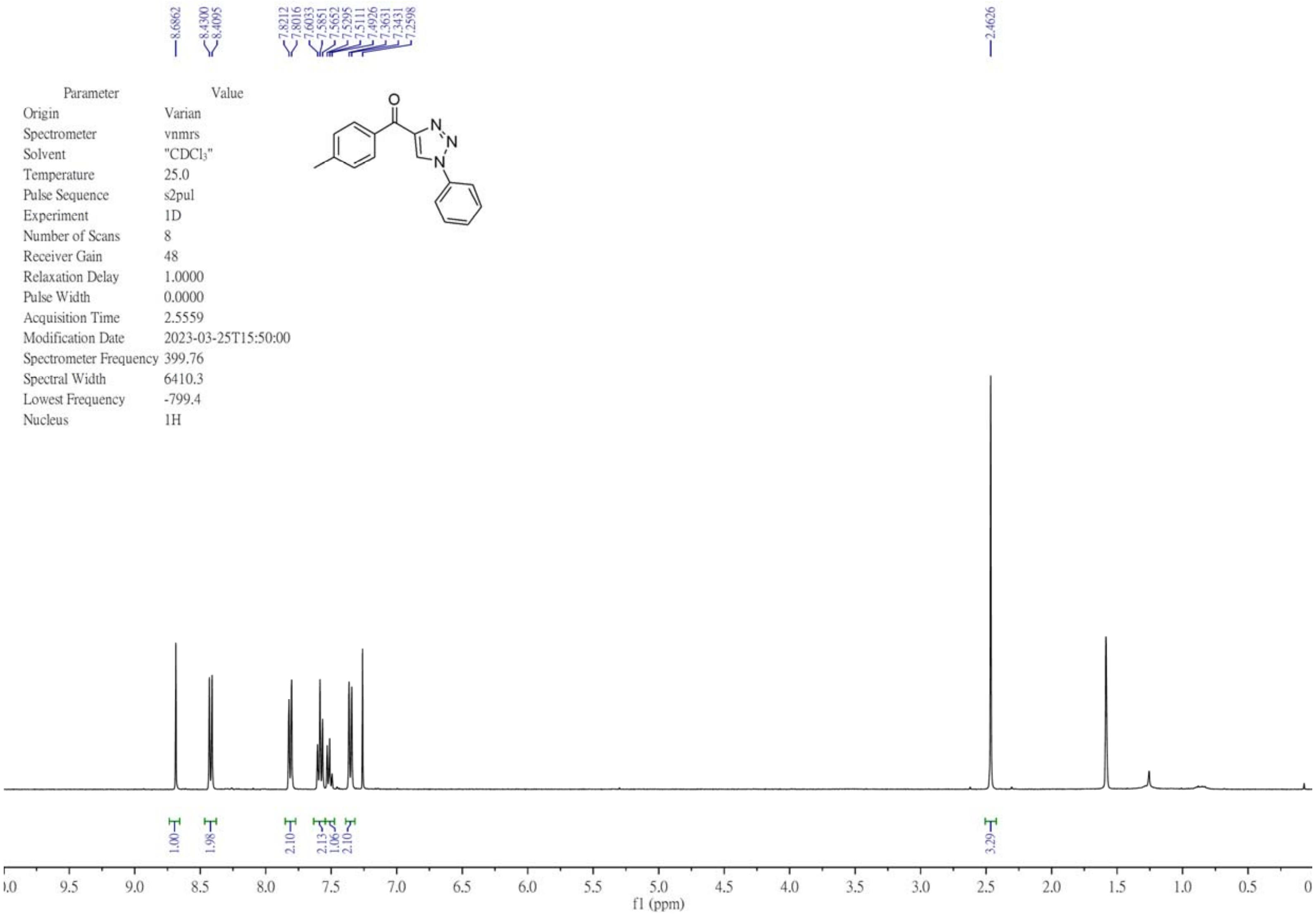

4ja ^{1}H NMR spectrum (400 MHz in $CDCl_3$)

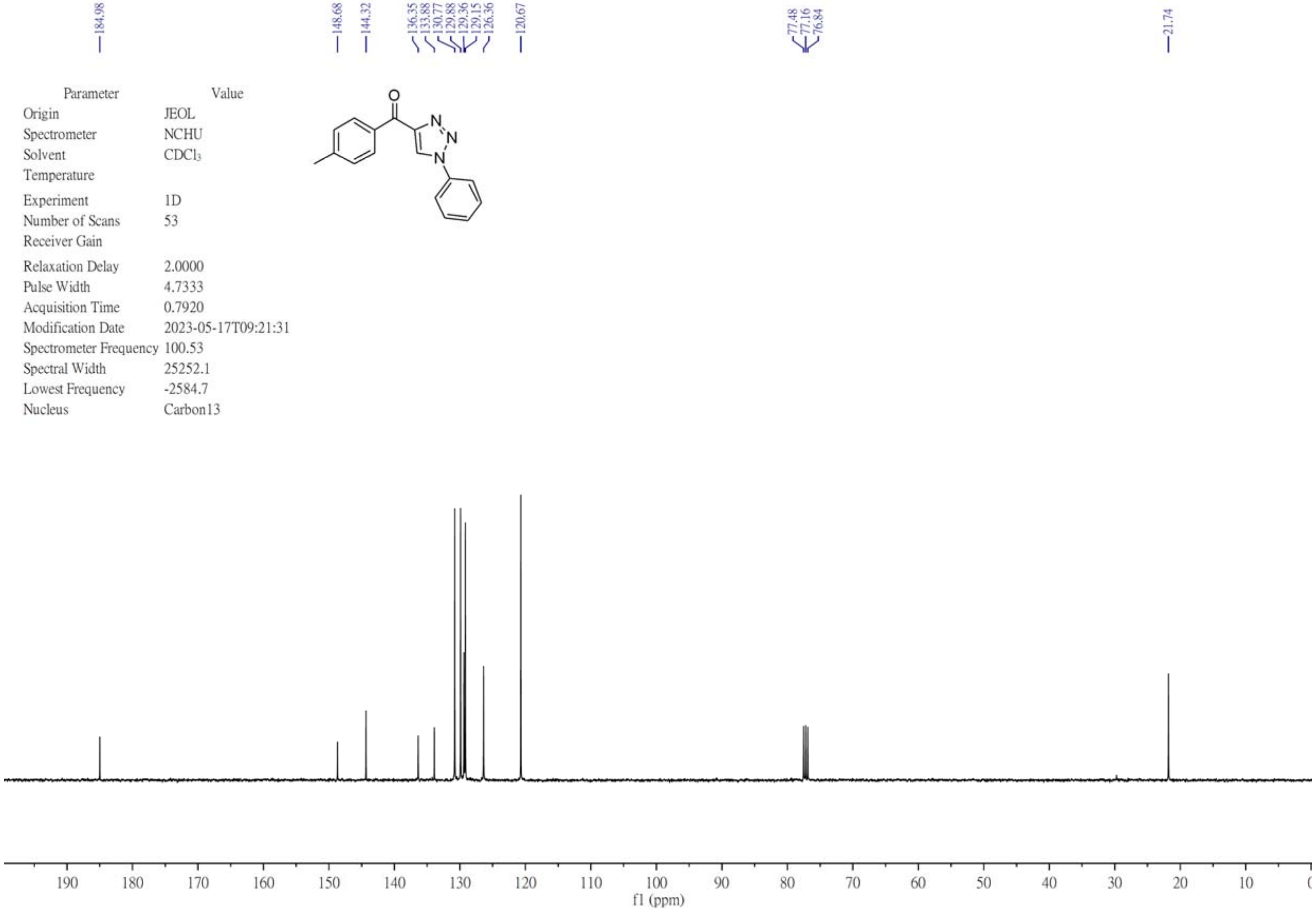

4ja $^{13}C\{^{1}H\}$ NMR spectrum (100 MHz in $CDCl_3$)

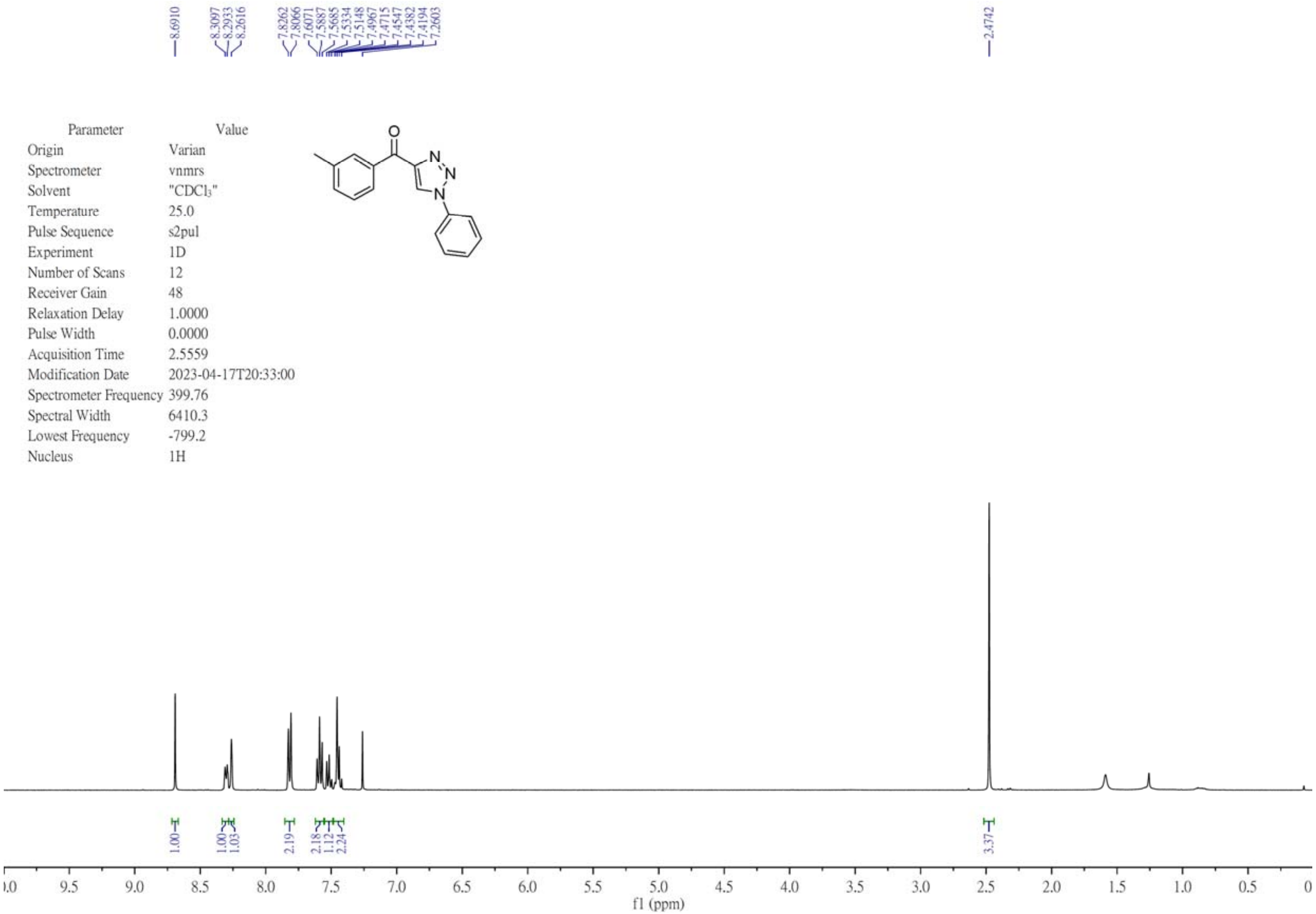

4ka ^{1}H NMR spectrum (400 MHz in $CDCl_3$)

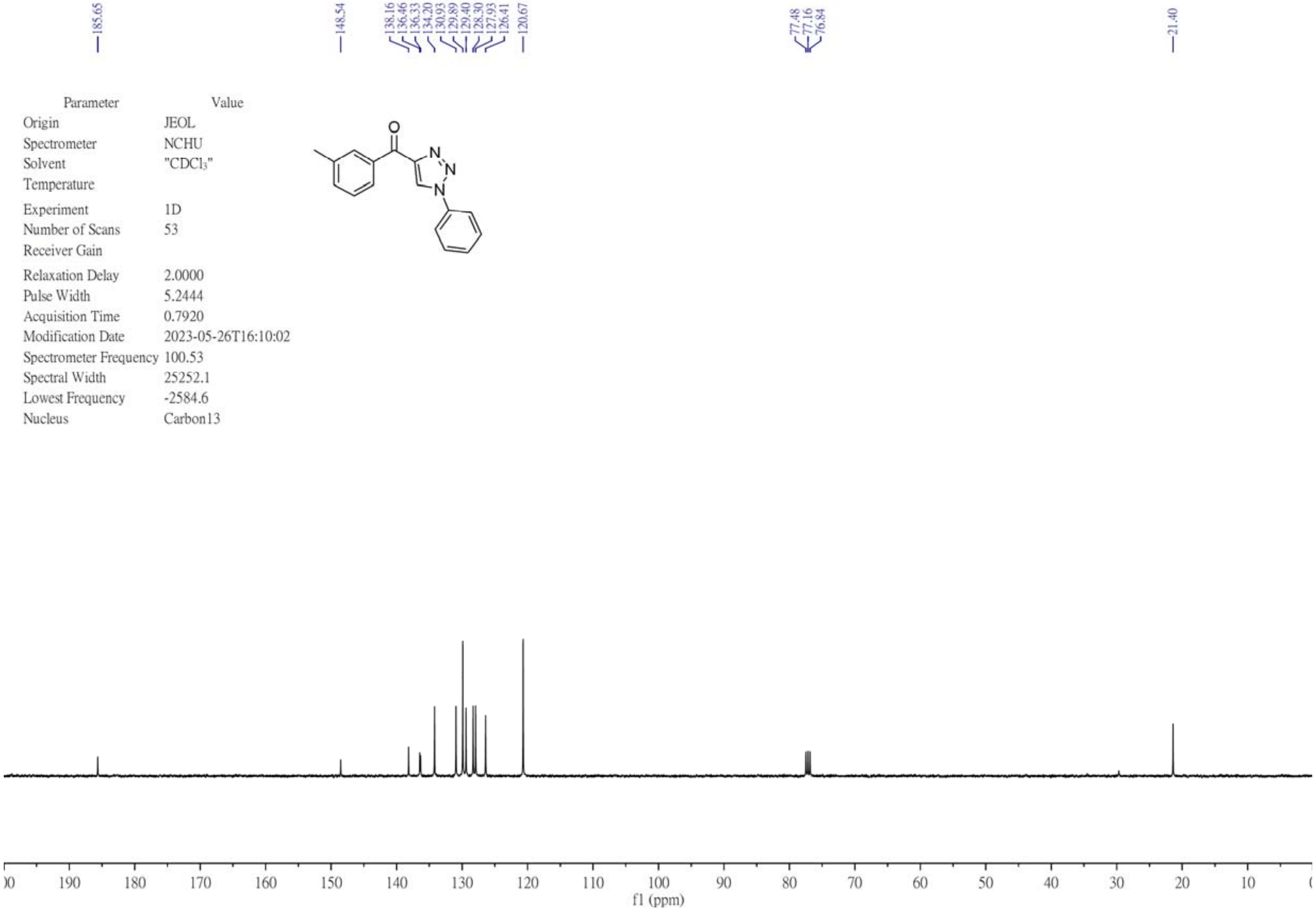

4ka $^{13}C\{^{1}H\}$ NMR spectrum (100 MHz in $CDCl_3$)

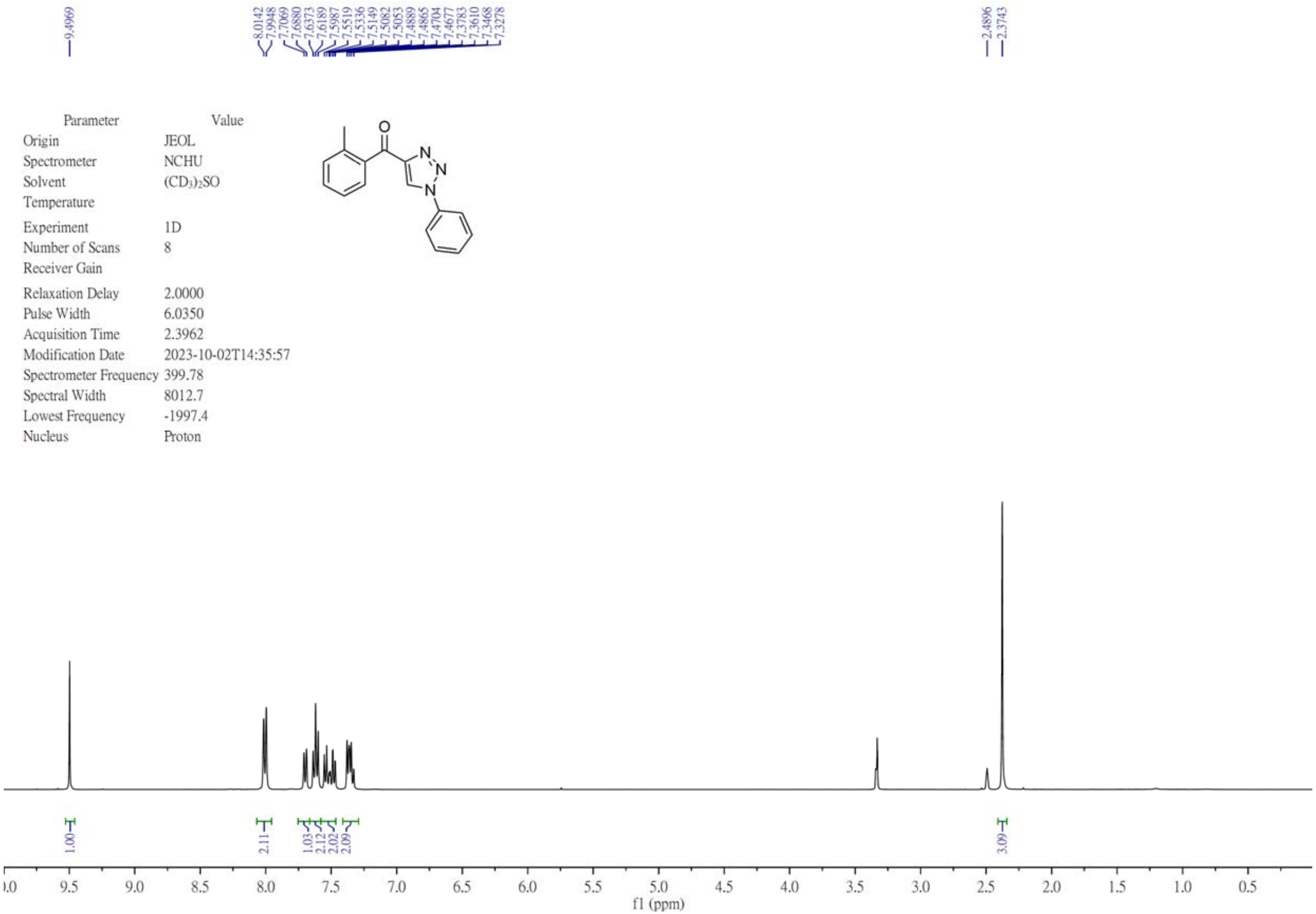

4la ^{1}H NMR spectrum (400 MHz in $(CD_3)_2SO$)

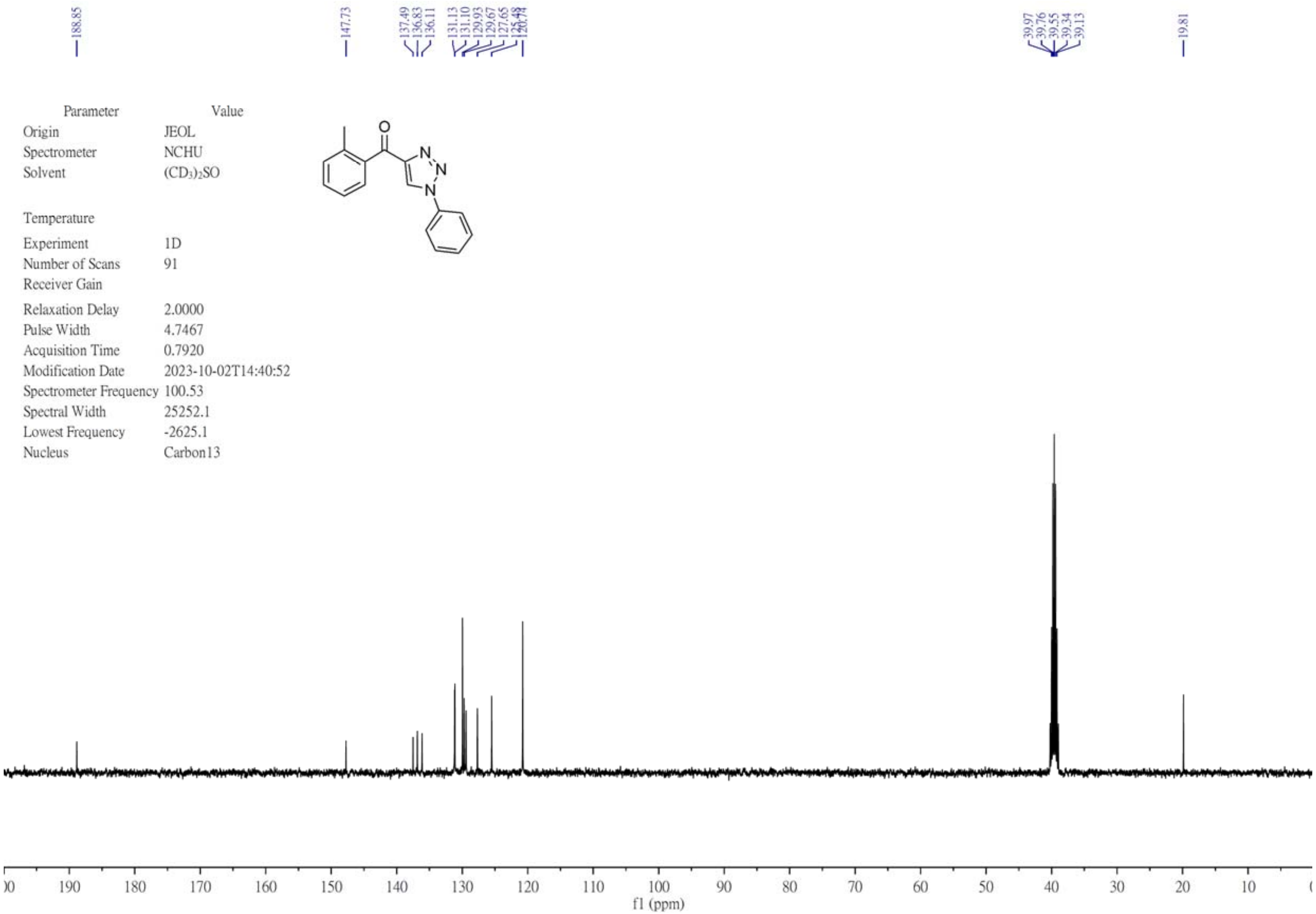

4la $^{13}C\{^{1}H\}$ NMR spectrum (100 MHz in $(CD_3)_2SO$)

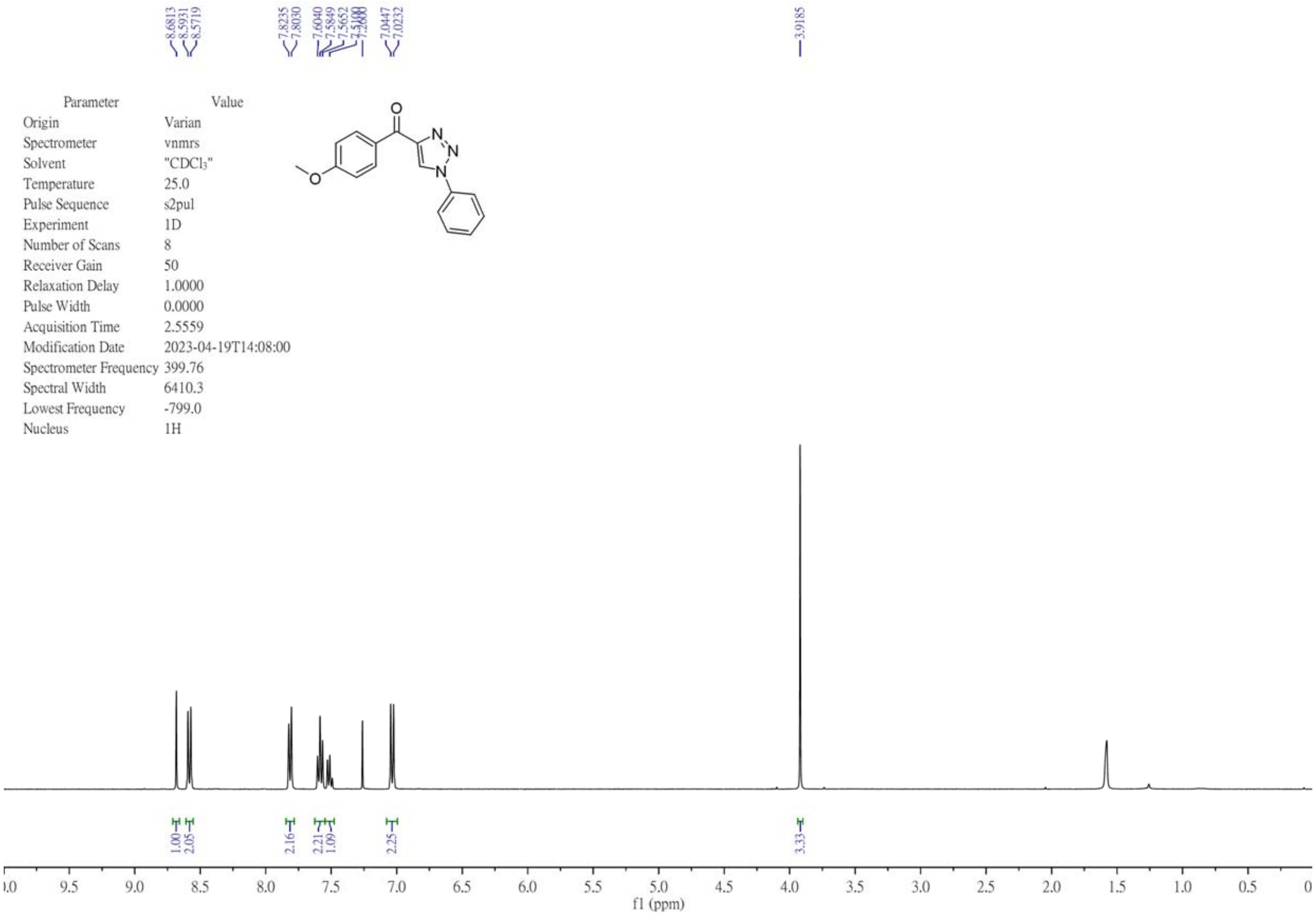

4ma ^{1}H NMR spectrum (400 MHz in $CDCl_3$)

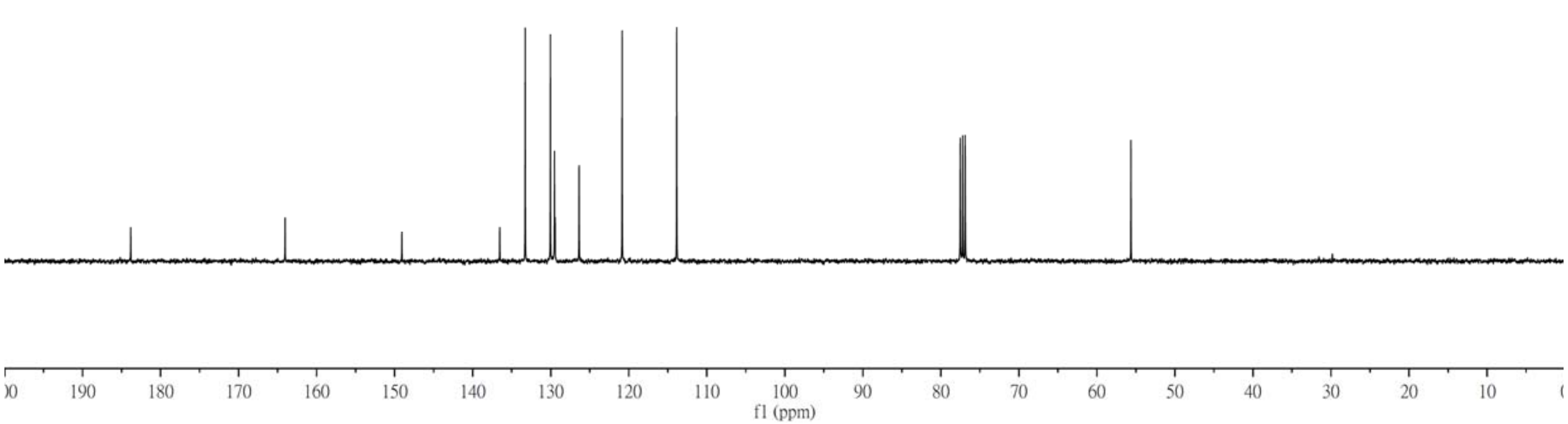

Parameter	Value
Origin	JEOL
Spectrometer	NCHU
Solvent	"$CDCl_3$"
Temperature	
Experiment	1D
Number of Scans	152
Receiver Gain	
Relaxation Delay	2.0000
Pulse Width	5.3333
Acquisition Time	0.7920
Modification Date	2023-05-16T19:11:24
Spectrometer Frequency	100.53
Spectral Width	25252.1
Lowest Frequency	-2575.1
Nucleus	Carbon13

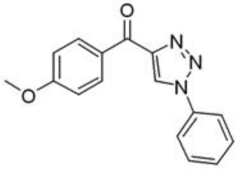

4ma $^{13}C\{^1H\}$ NMR spectrum (100 MHz in $CDCl_3$)

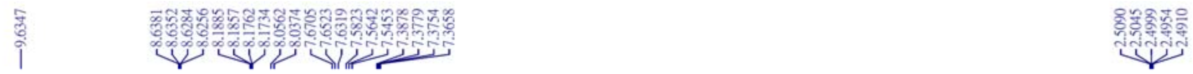

Parameter	Value
Origin	Varian
Spectrometer	vnmrs
Solvent	"$(CD_3)_2SO$"
Temperature	20.0
Pulse Sequence	s2pul
Experiment	1D
Number of Scans	4
Receiver Gain	52
Relaxation Delay	1.0000
Pulse Width	0.0000
Acquisition Time	2.5559
Modification Date	2023-12-19T12:19:00
Spectrometer Frequency	399.76
Spectral Width	6410.3
Lowest Frequency	-793.8
Nucleus	1H

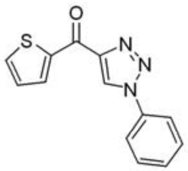

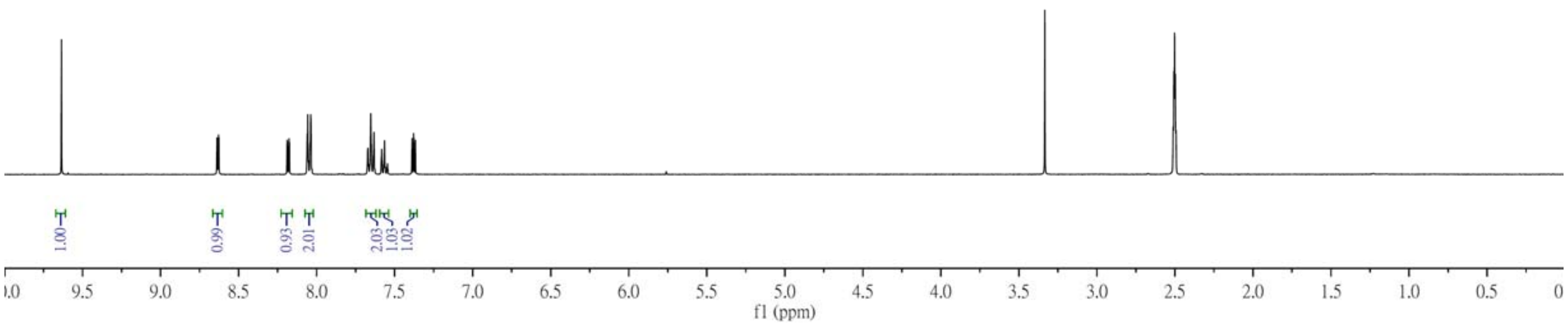

4na ^{1}H NMR spectrum (400 MHz in $CDCl_3$)

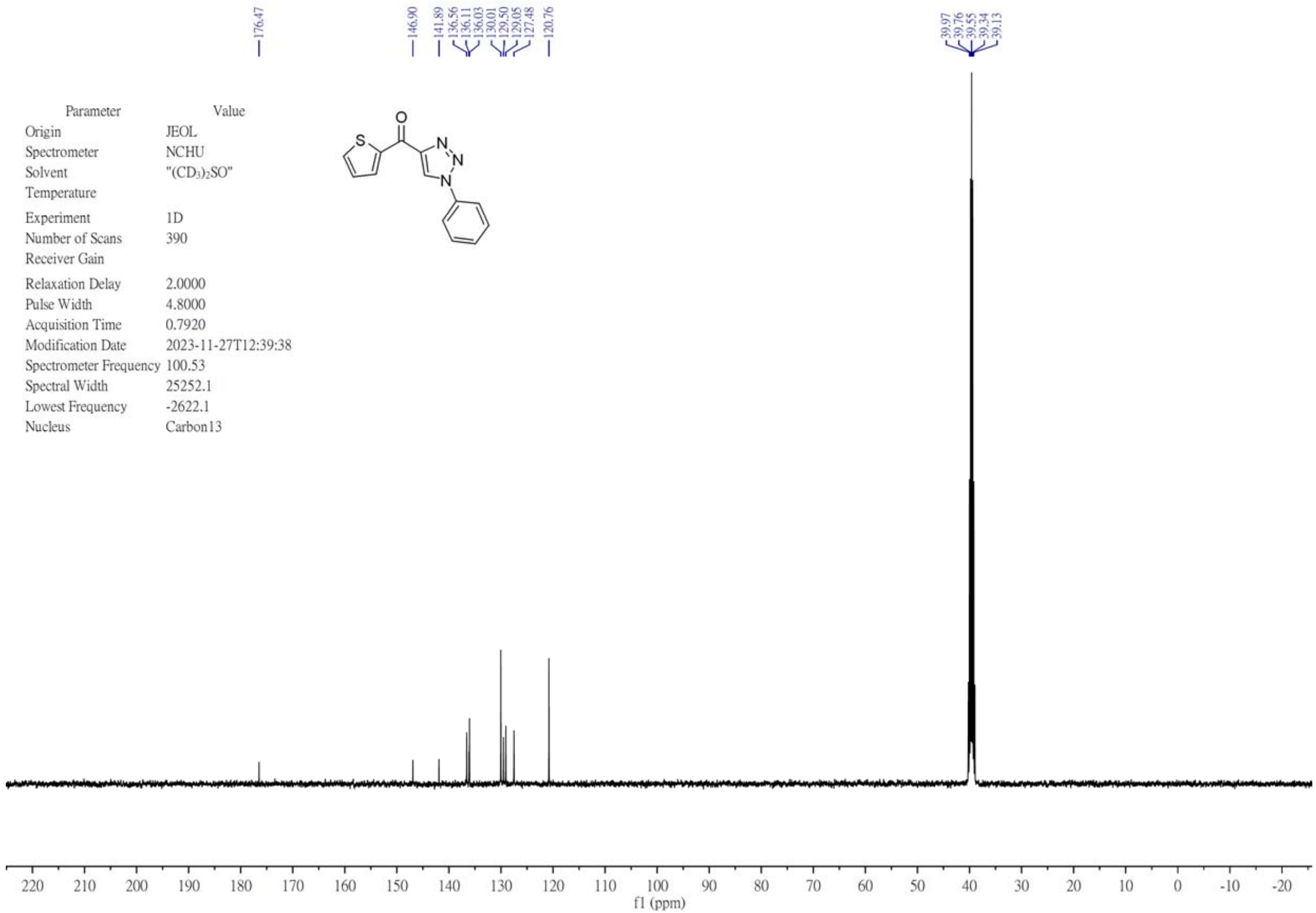

4na $^{13}C\{^1H\}$ NMR spectrum (100 MHz in $CDCl_3$)

Parameter	Value
Origin	Varian
Spectrometer	vnmrs
Solvent	"$CDCl_3$"
Temperature	25.0
Pulse Sequence	s2pul
Experiment	1D
Number of Scans	8
Receiver Gain	52
Relaxation Delay	1.0000
Pulse Width	0.0000
Acquisition Time	2.5559
Modification Date	2023-05-08T13:34:00
Spectrometer Frequency	399.76
Spectral Width	6410.3
Lowest Frequency	-799.2
Nucleus	1H

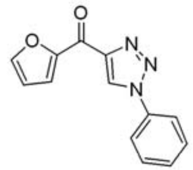

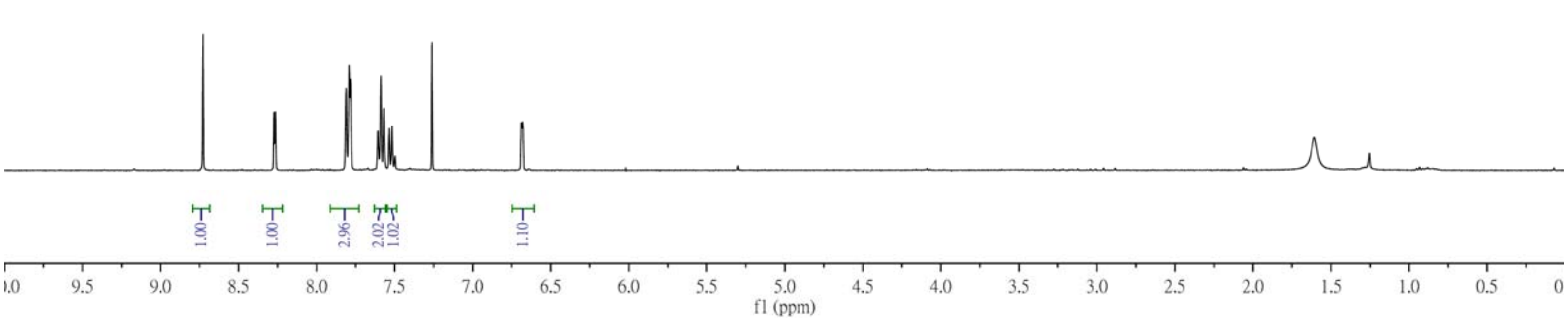

4oa ^{1}H NMR spectrum (400 MHz in $CDCl_3$)

Parameter	Value
Origin	JEOL
Spectrometer	NCHU
Solvent	"$(CD_3)_2SO$"
Temperature	
Experiment	1D
Number of Scans	159
Receiver Gain	
Relaxation Delay	2.0000
Pulse Width	5.2444
Acquisition Time	0.7920
Modification Date	2023-05-25T16:16:41
Spectrometer Frequency	100.53
Spectral Width	25252.1
Lowest Frequency	-2624.7
Nucleus	Carbon13

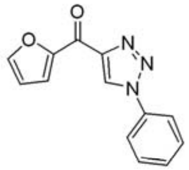

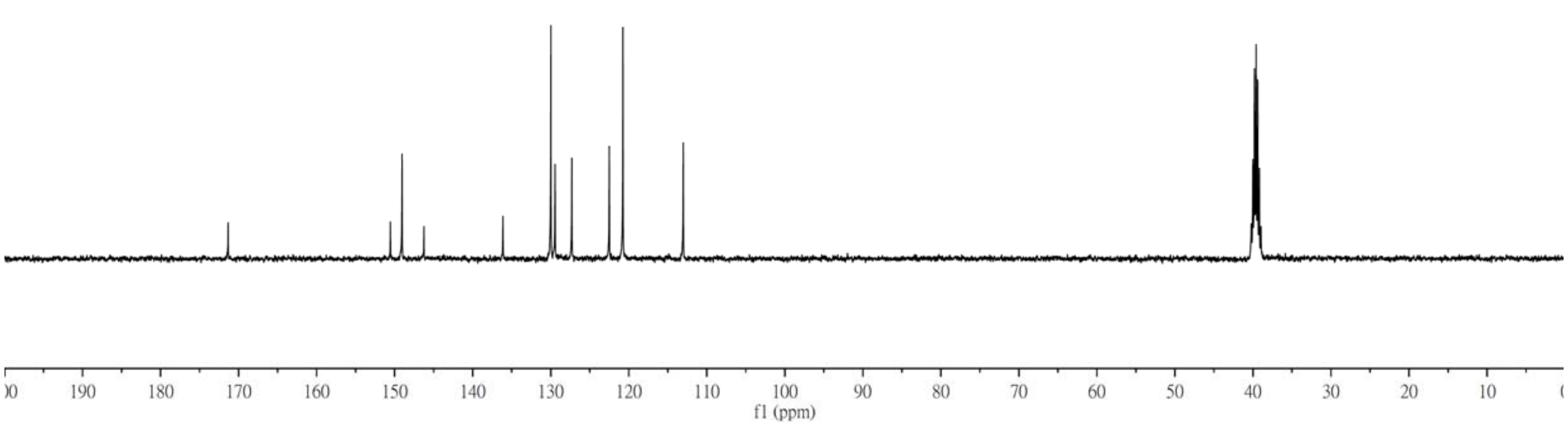

4oa $^{13}C\{^{1}H\}$ NMR spectrum (100 MHz in $(CD_3)_2SO$)

Parameter	Value
Origin	Varian
Spectrometer	vnmrs
Solvent	"$CDCl_3$"
Temperature	25.0
Pulse Sequence	s2pul
Experiment	1D
Number of Scans	8
Receiver Gain	52
Relaxation Delay	1.0000
Pulse Width	0.0000
Acquisition Time	2.5559
Modification Date	2023-04-14T12:26:00
Spectrometer Frequency	399.76
Spectral Width	6410.3
Lowest Frequency	-799.4
Nucleus	1H

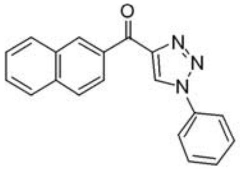

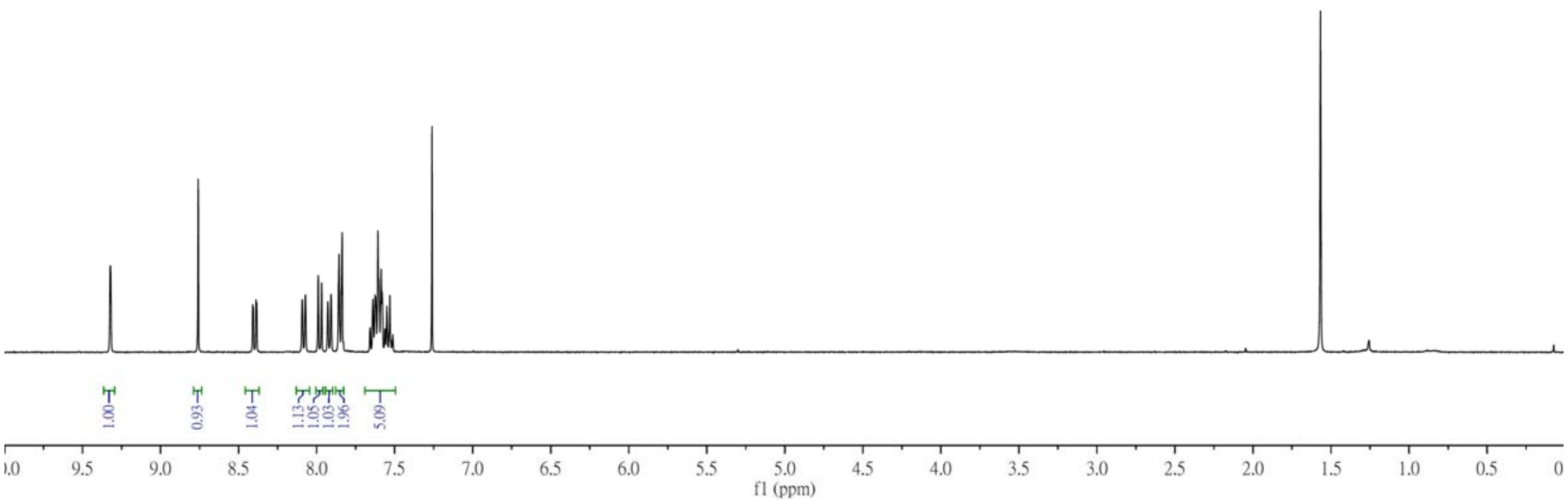

4pa ^1H NMR spectrum (400 MHz in $CDCl_3$)

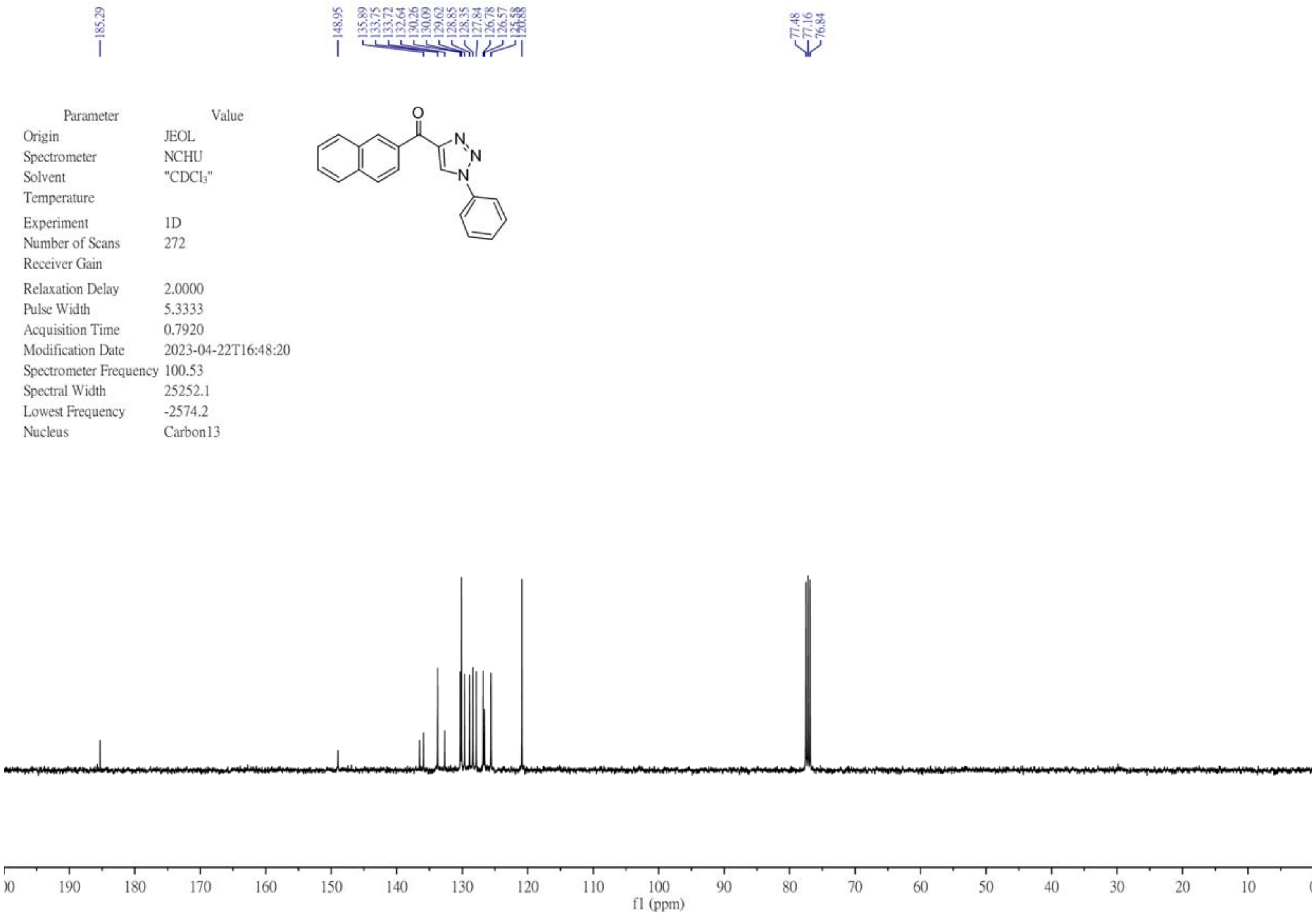

4pa $^{13}C\{^1H\}$ NMR spectrum (100 MHz in $CDCl_3$)

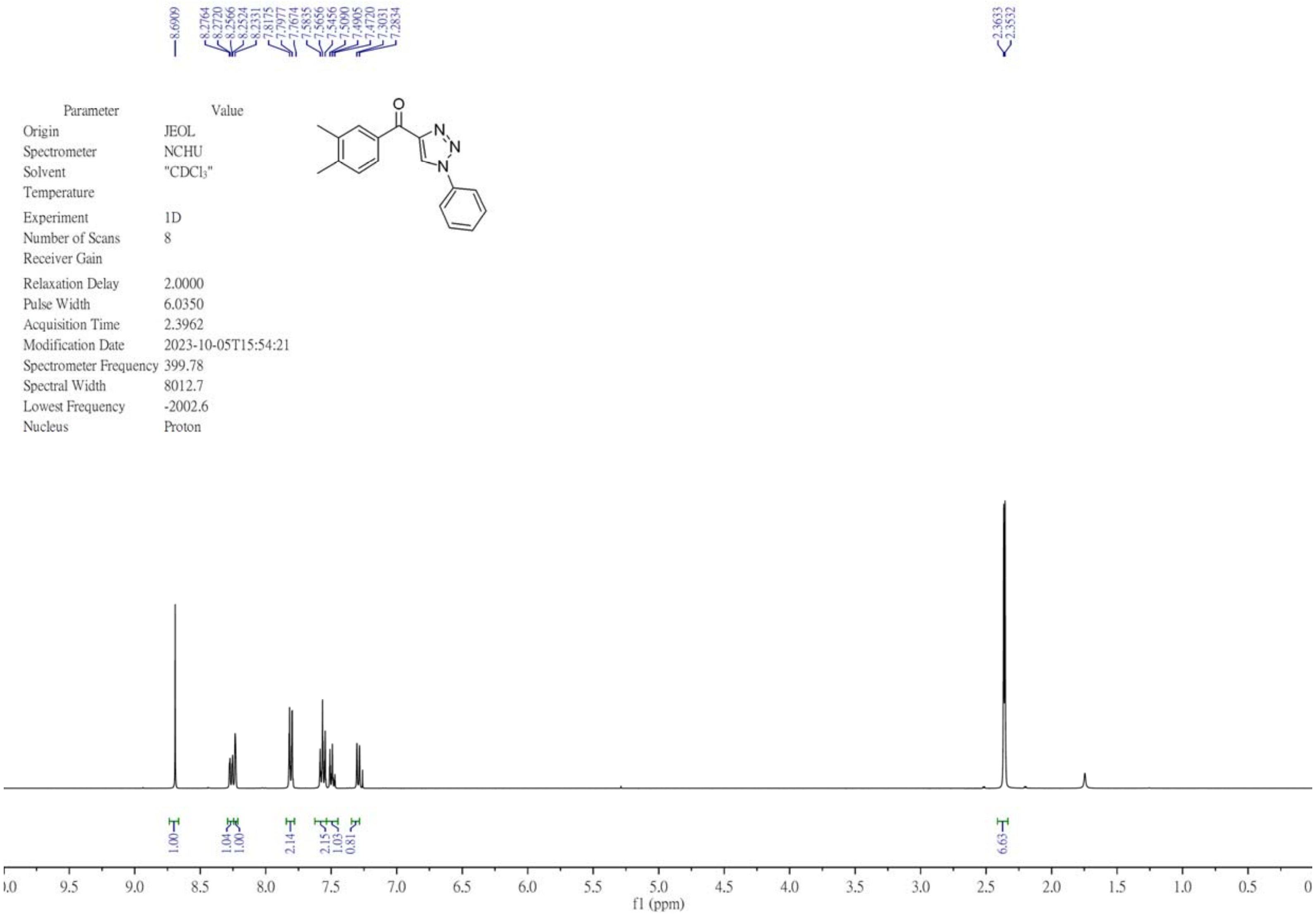

4qa ^{1}H NMR spectrum (400 MHz in $CDCl_3$)

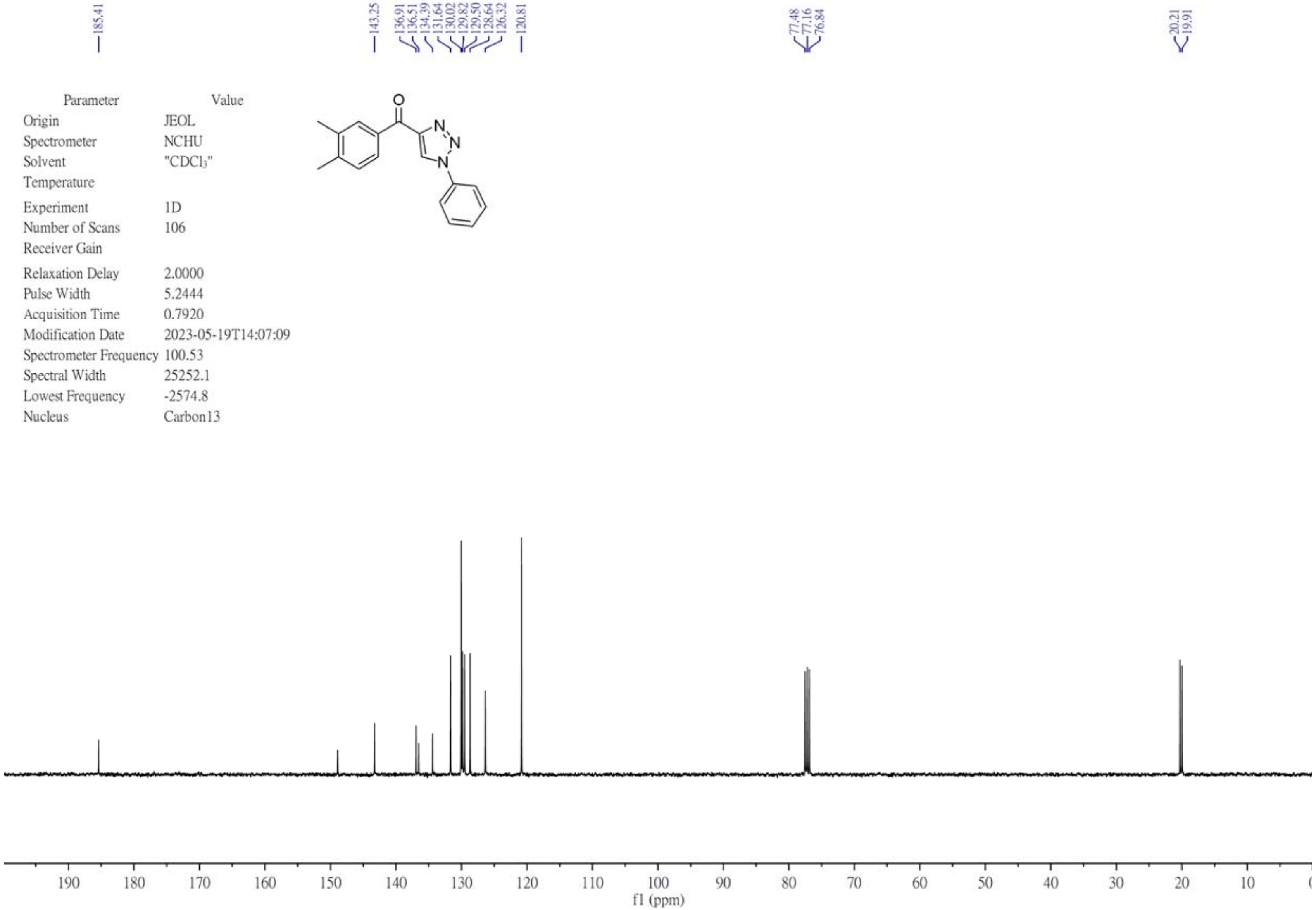

4qa $^{13}C\{^1H\}$ NMR spectrum (100 MHz in $CDCl_3$)

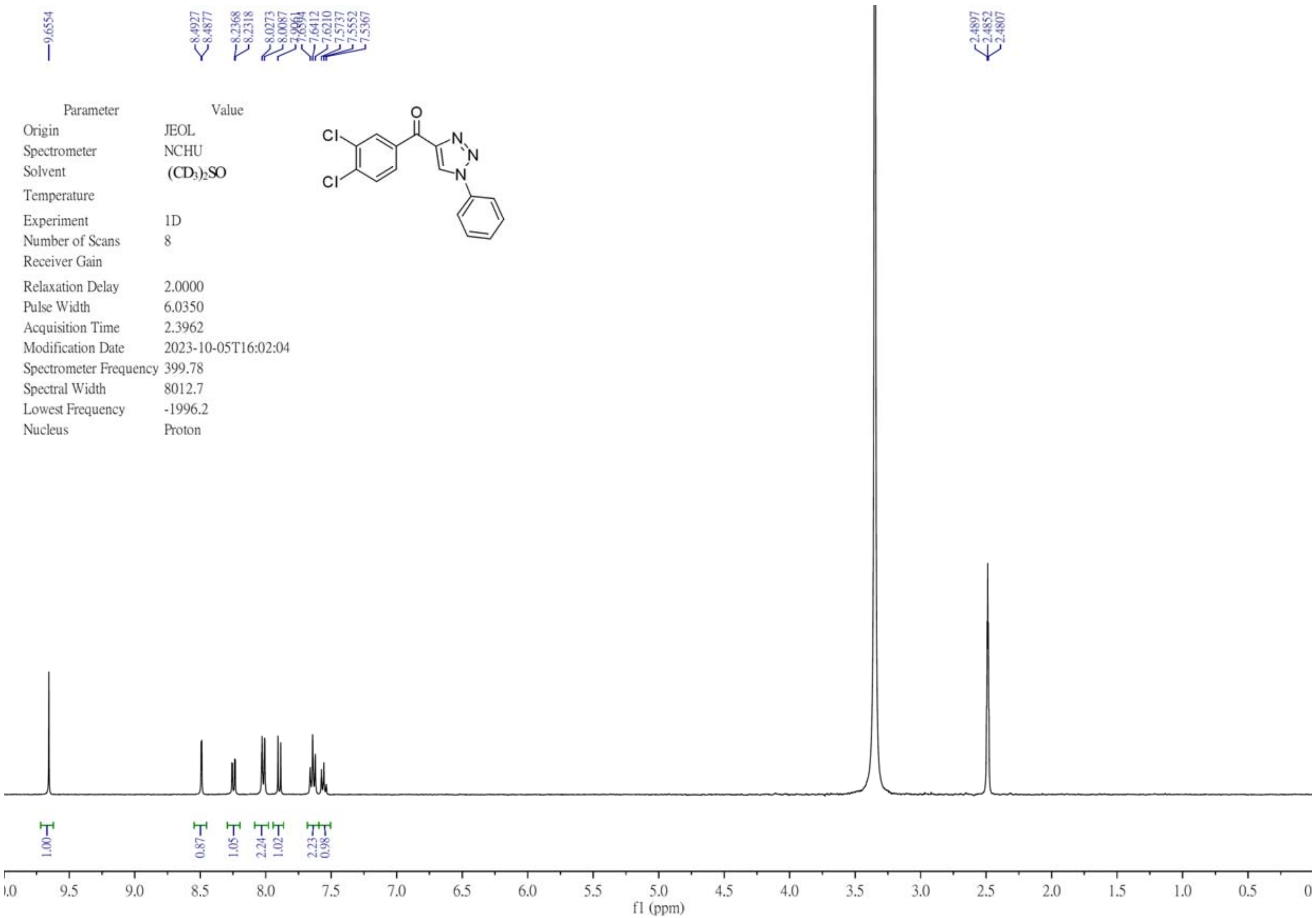

4ra ^{1}H NMR spectrum (400 MHz in $(CD_3)_2SO$)

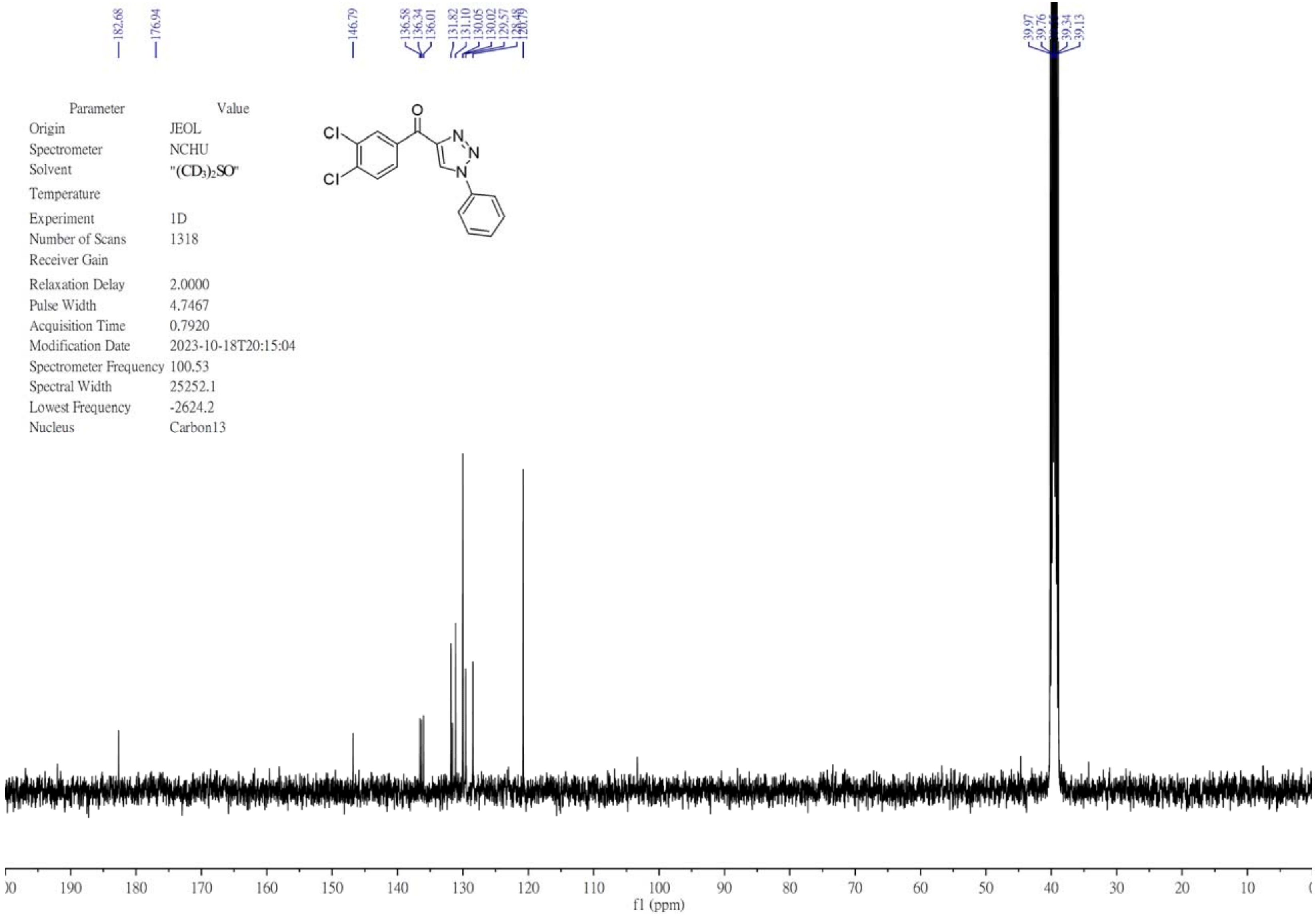

4ra $^{13}C\{^{1}H\}$ NMR spectrum (100 MHz in $(CD_3)_2SO$)

Parameter	Value
Origin	Varian
Spectrometer	vnmrs
Solvent	"cdcl3"
Temperature	30.0
Pulse Sequence	s2pul
Experiment	1D
Number of Scans	8
Receiver Gain	56
Relaxation Delay	1.0000
Pulse Width	0.0000
Acquisition Time	2.5559
Spectrometer Frequency	399.76
Spectral Width	6410.3
Lowest Frequency	-799.8
Nucleus	1H

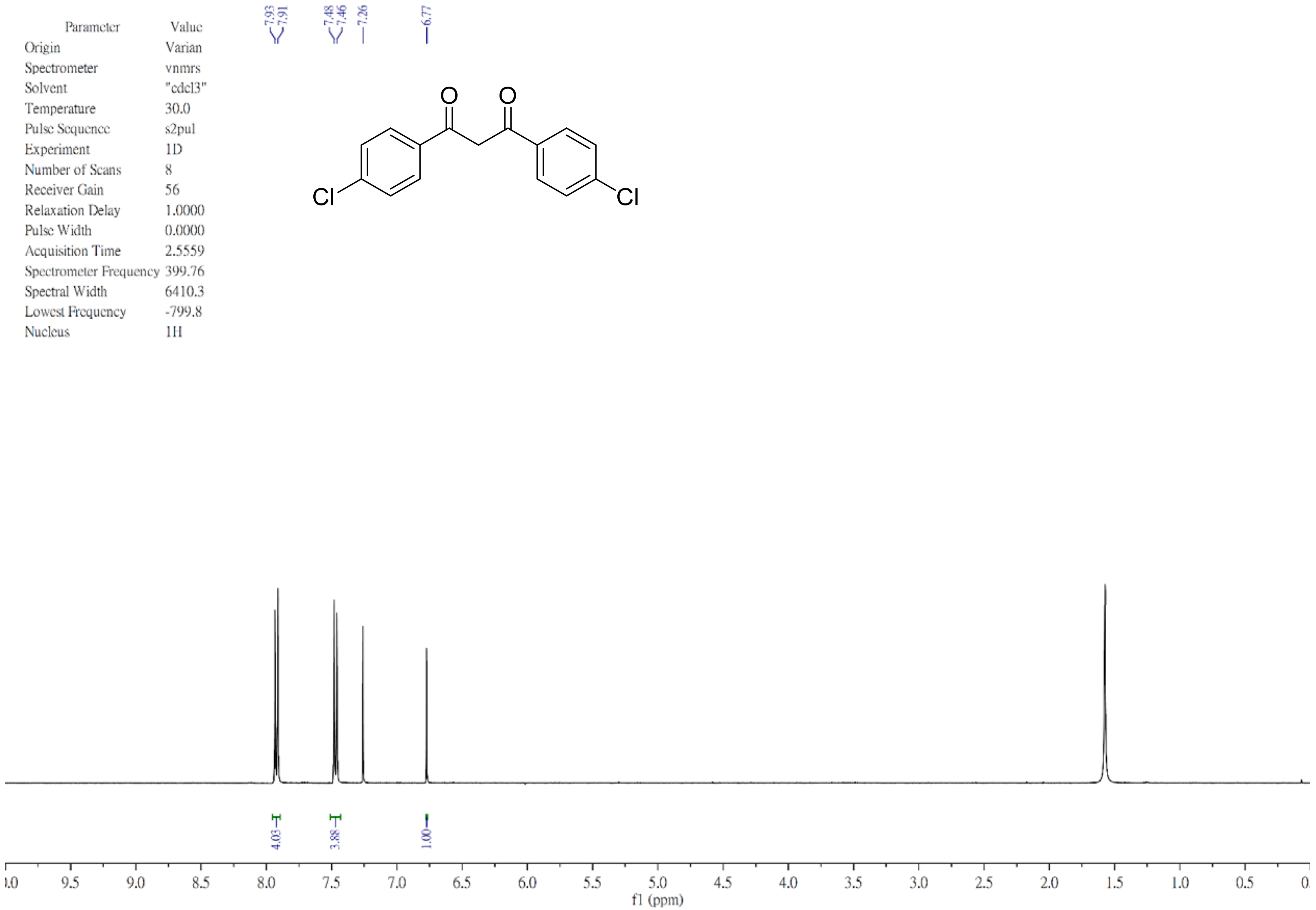

2b ^{1}H NMR spectrum (400 MHz in ($CDCl_3$))

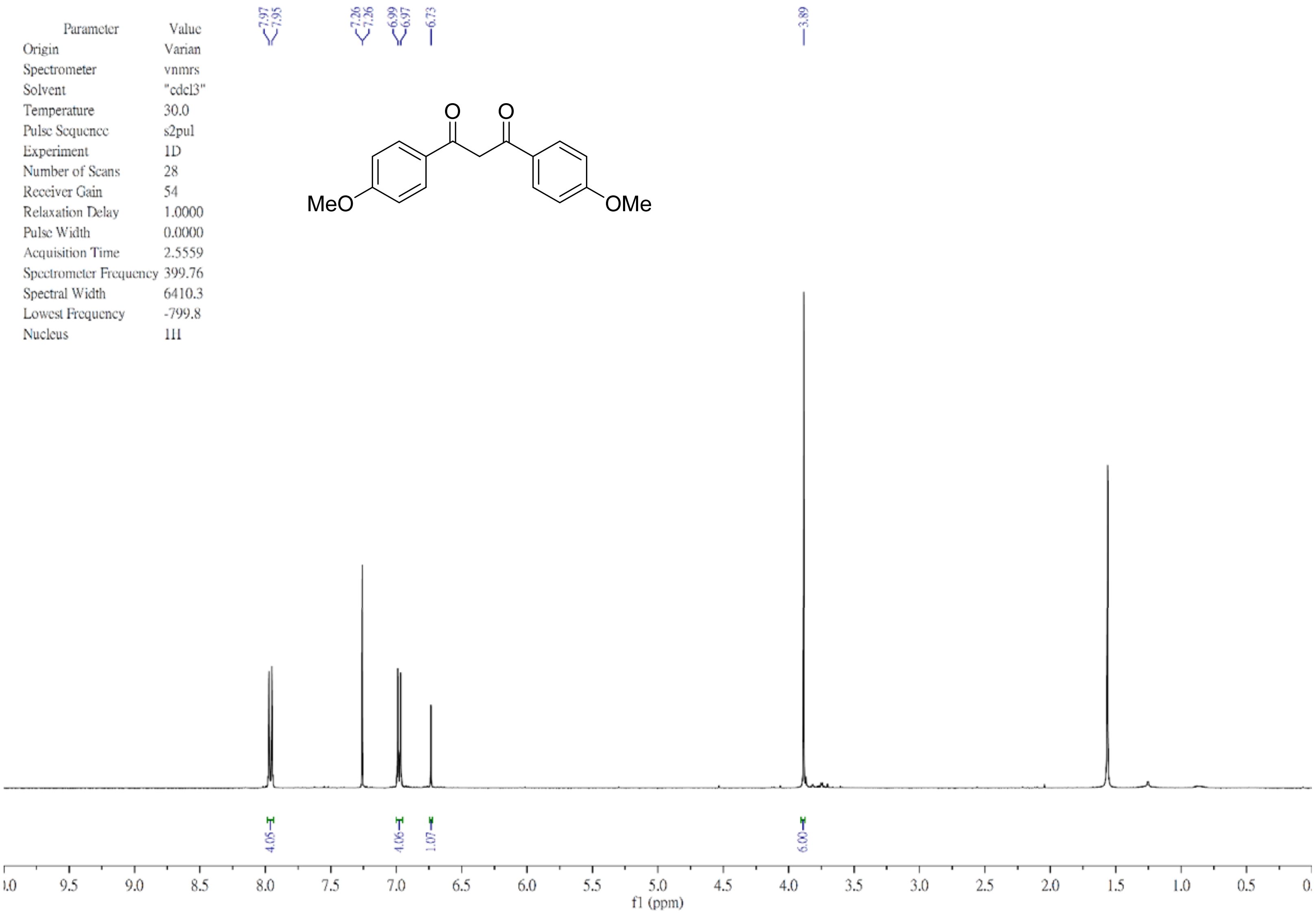

2c ^{1}H NMR spectrum (400 MHz in ($CDCl_3$))

Parameter	Value
Origin	Varian
Spectrometer	vnmrs
Solvent	"cdcl3"
Temperature	30.0
Pulse Sequence	s2pul
Experiment	1D
Number of Scans	16
Receiver Gain	42
Relaxation Delay	1.0000
Pulse Width	0.0000
Acquisition Time	2.5559
Spectrometer Frequency	399.76
Spectral Width	6410.3
Lowest Frequency	-799.8
Nucleus	1H

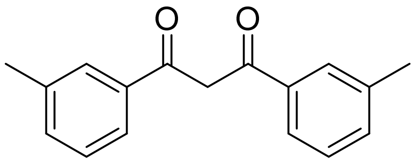

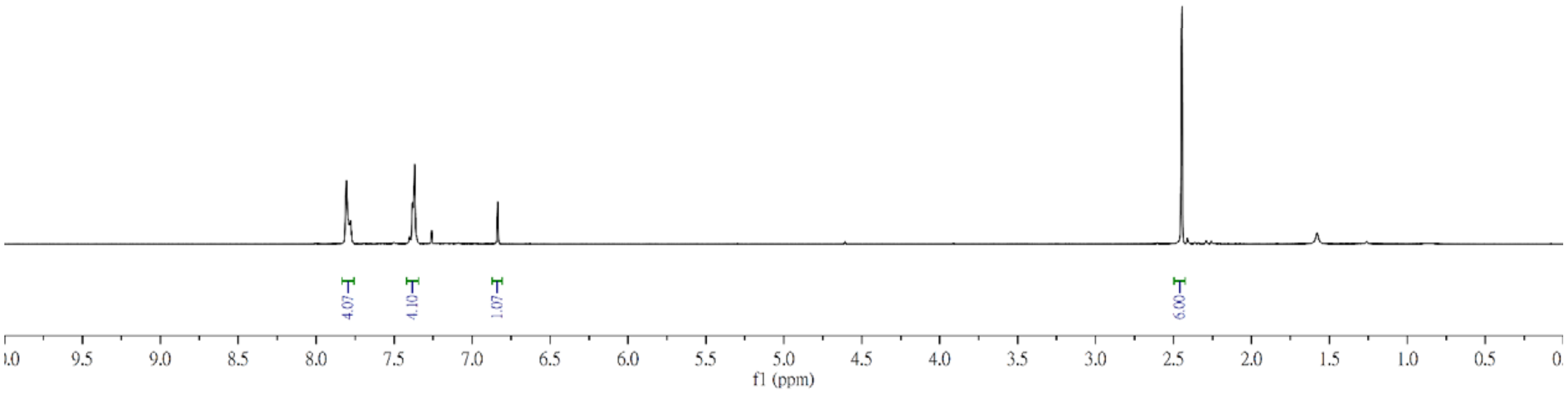

2d ^{1}H NMR spectrum (400 MHz in ($CDCl_3$)

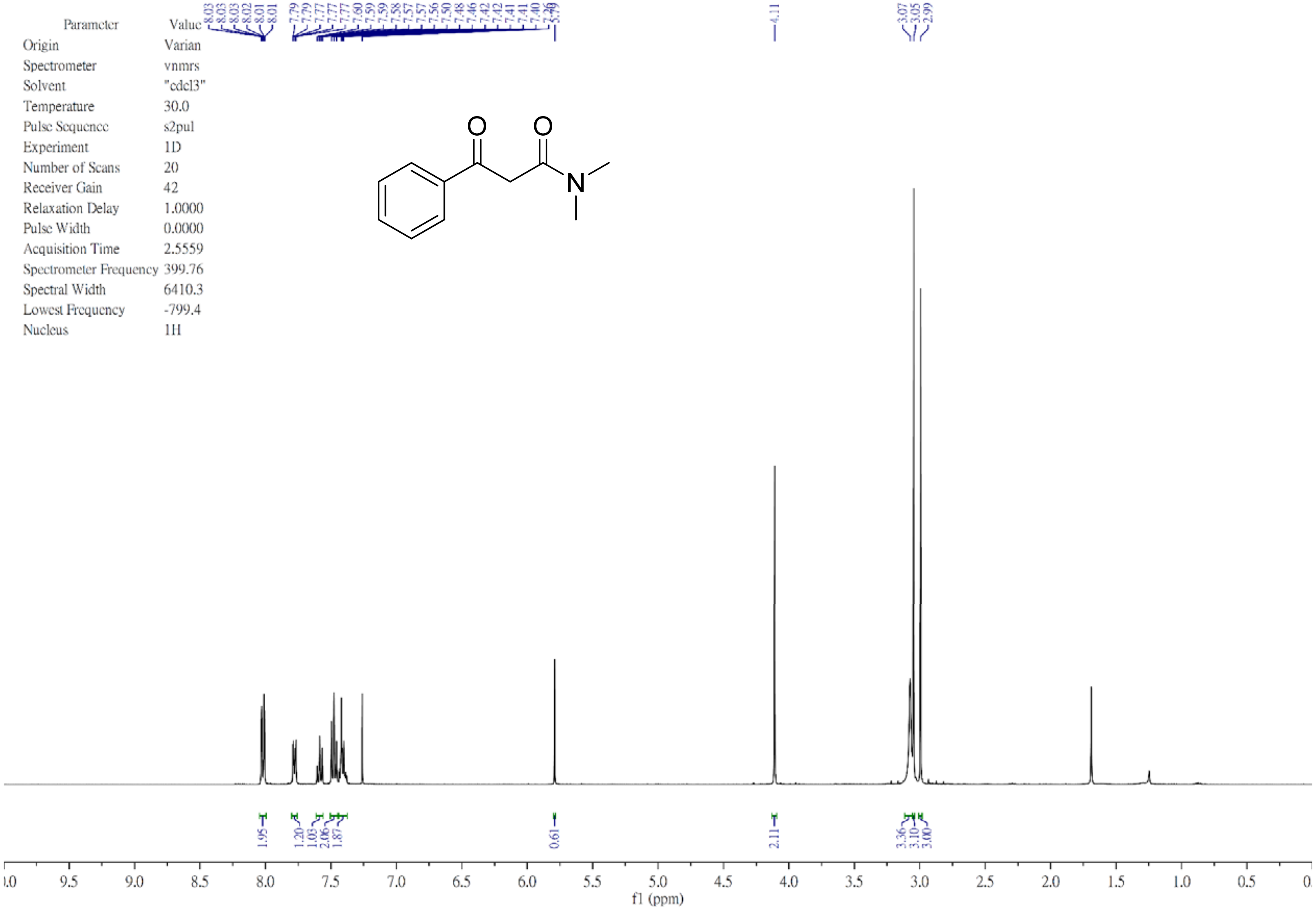

2g ^{1}H NMR spectrum (400 MHz in ($CDCl_3$)

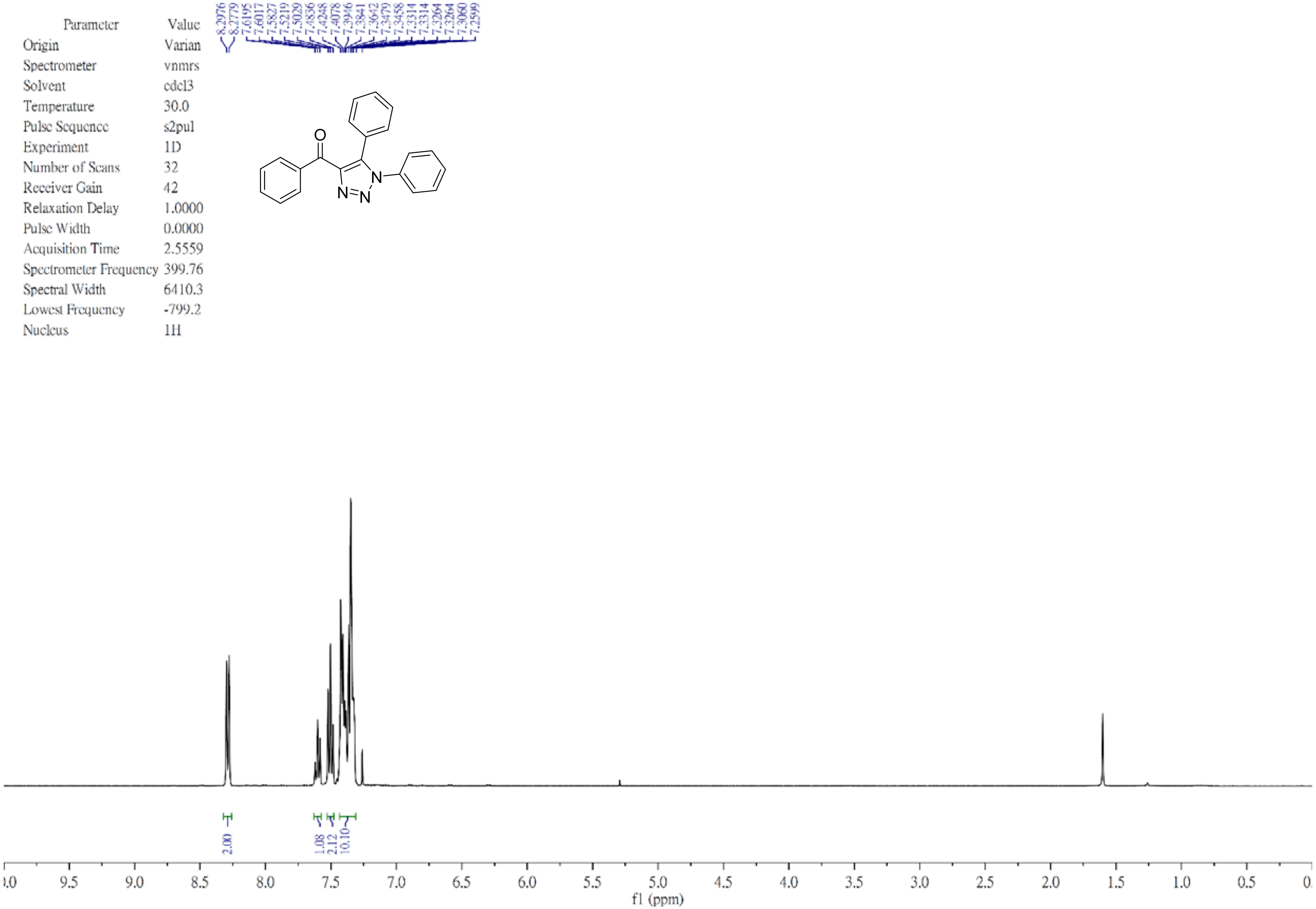

5aa ^{1}H NMR spectrum (400 MHz in $CDCl_3$)

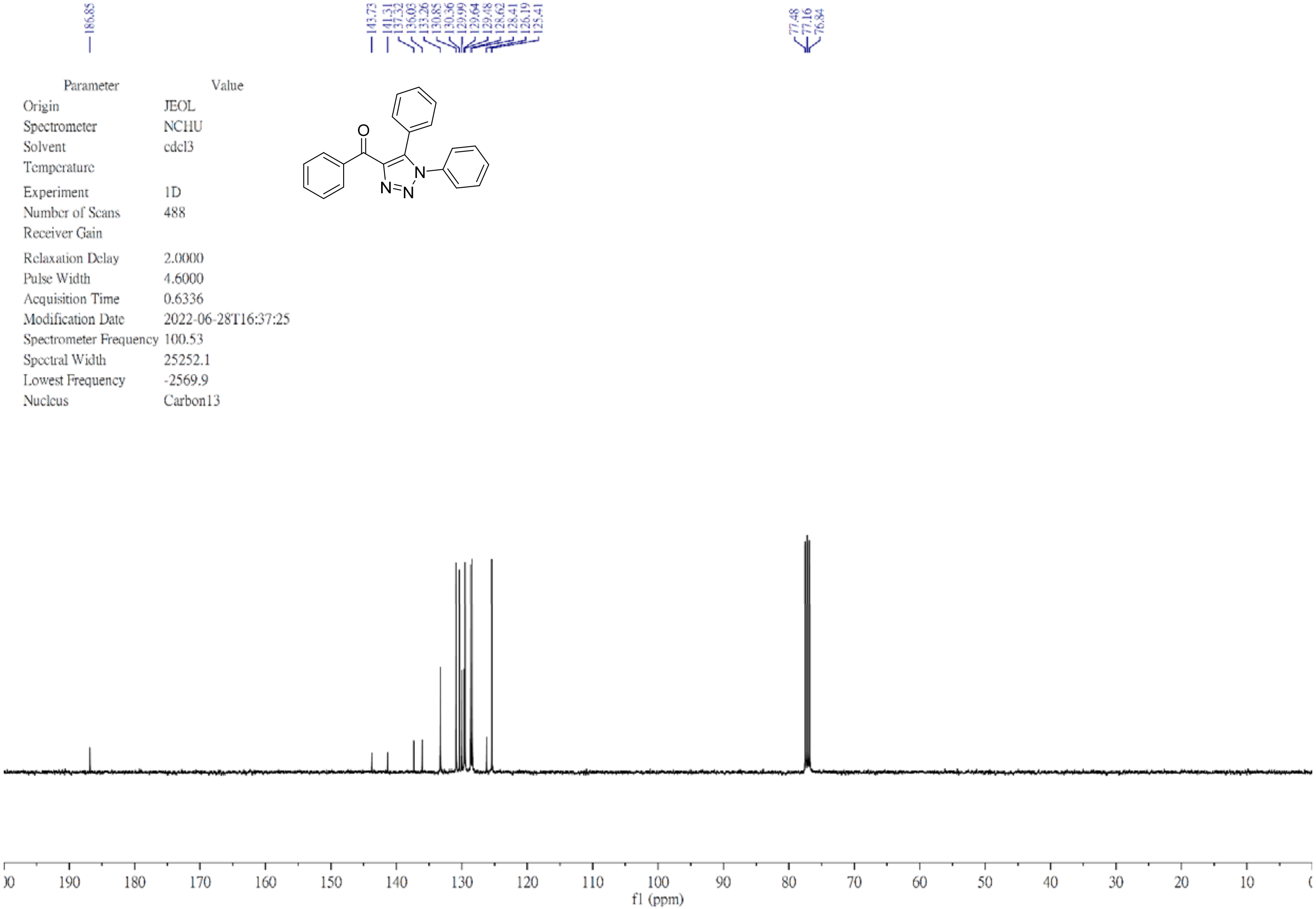

5aa $^{13}C\{^{1}H\}$ NMR spectrum (100 MHz in $CDCl_3$)

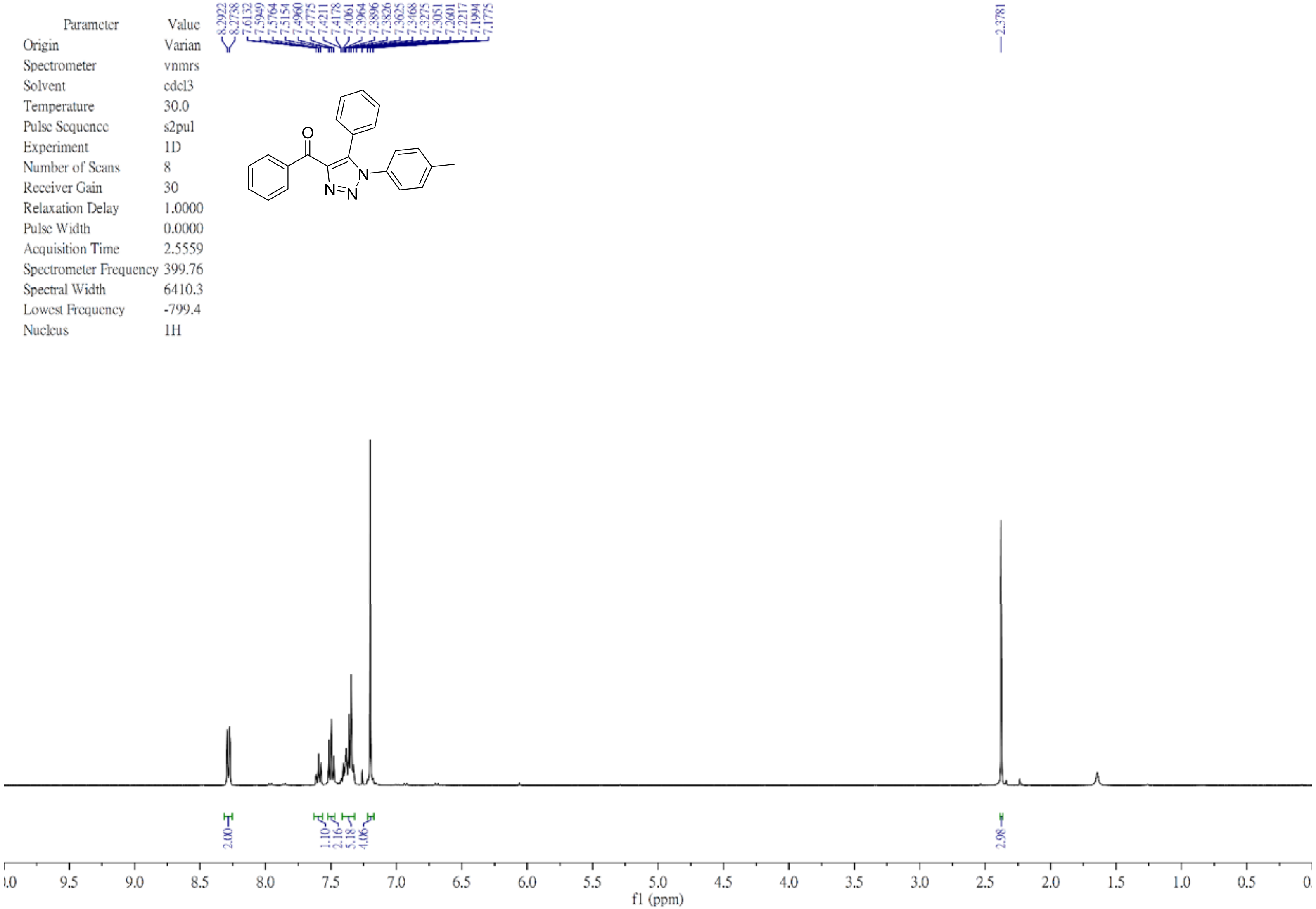

5ab ^{1}H NMR spectrum (400 MHz in $CDCl_3$)

Parameter	Value
Origin	JEOL
Spectrometer	NCHU
Solvent	cdcl3
Temperature	
Experiment	1D
Number of Scans	191
Receiver Gain	
Relaxation Delay	2.0000
Pulse Width	4.6000
Acquisition Time	0.6336
Modification Date	2022-06-28T16:39:01
Spectrometer Frequency	100.53
Spectral Width	25252.1
Lowest Frequency	-2571.5
Nucleus	Carbon13

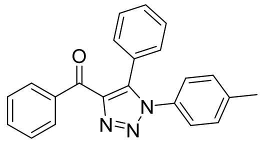

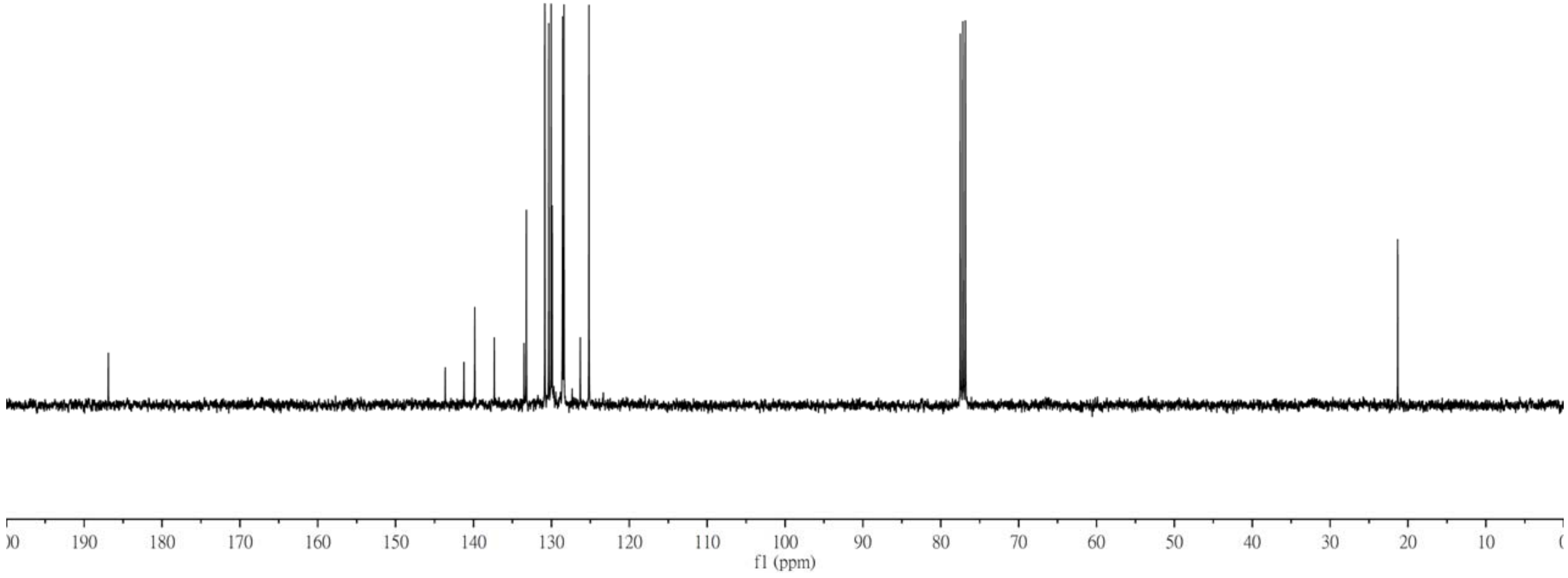

5ab $^{13}C\{^{1}H\}$ NMR spectrum (100 MHz in $CDCl_3$)

Parameter	Value
Origin	Varian
Spectrometer	vnmrs
Solvent	cdcl3
Temperature	30.0
Pulse Sequence	s2pul
Experiment	1D
Number of Scans	20
Receiver Gain	44
Relaxation Delay	1.0000
Pulse Width	0.0000
Acquisition Time	2.5559
Spectrometer Frequency	399.76
Spectral Width	6410.3
Lowest Frequency	-799.6
Nucleus	1H

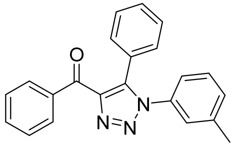

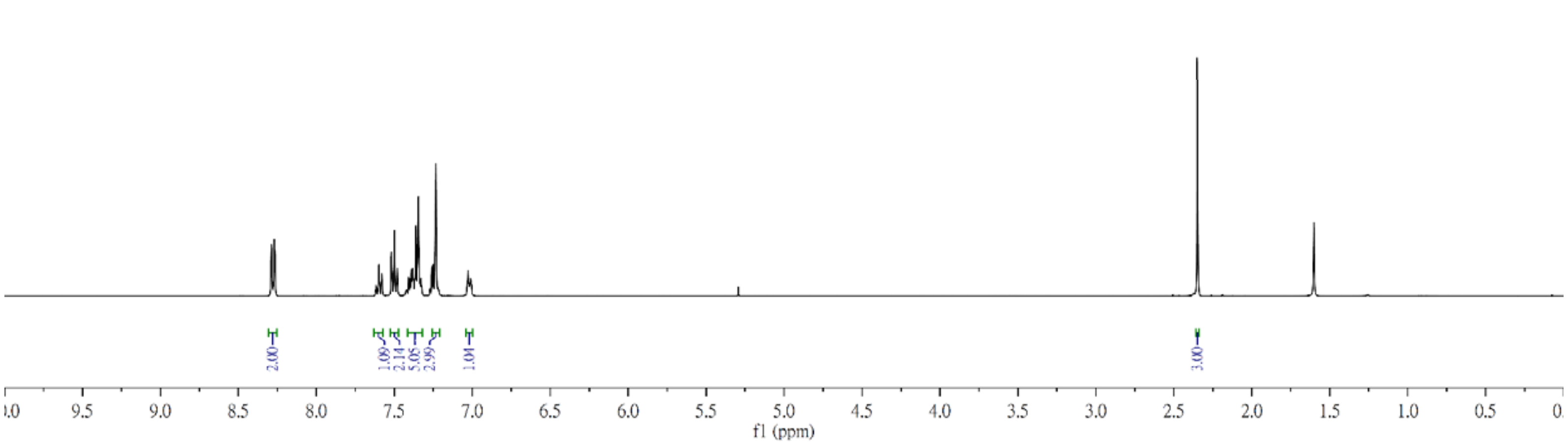

5ac ^{1}H NMR spectrum (400 MHz in $CDCl_3$)

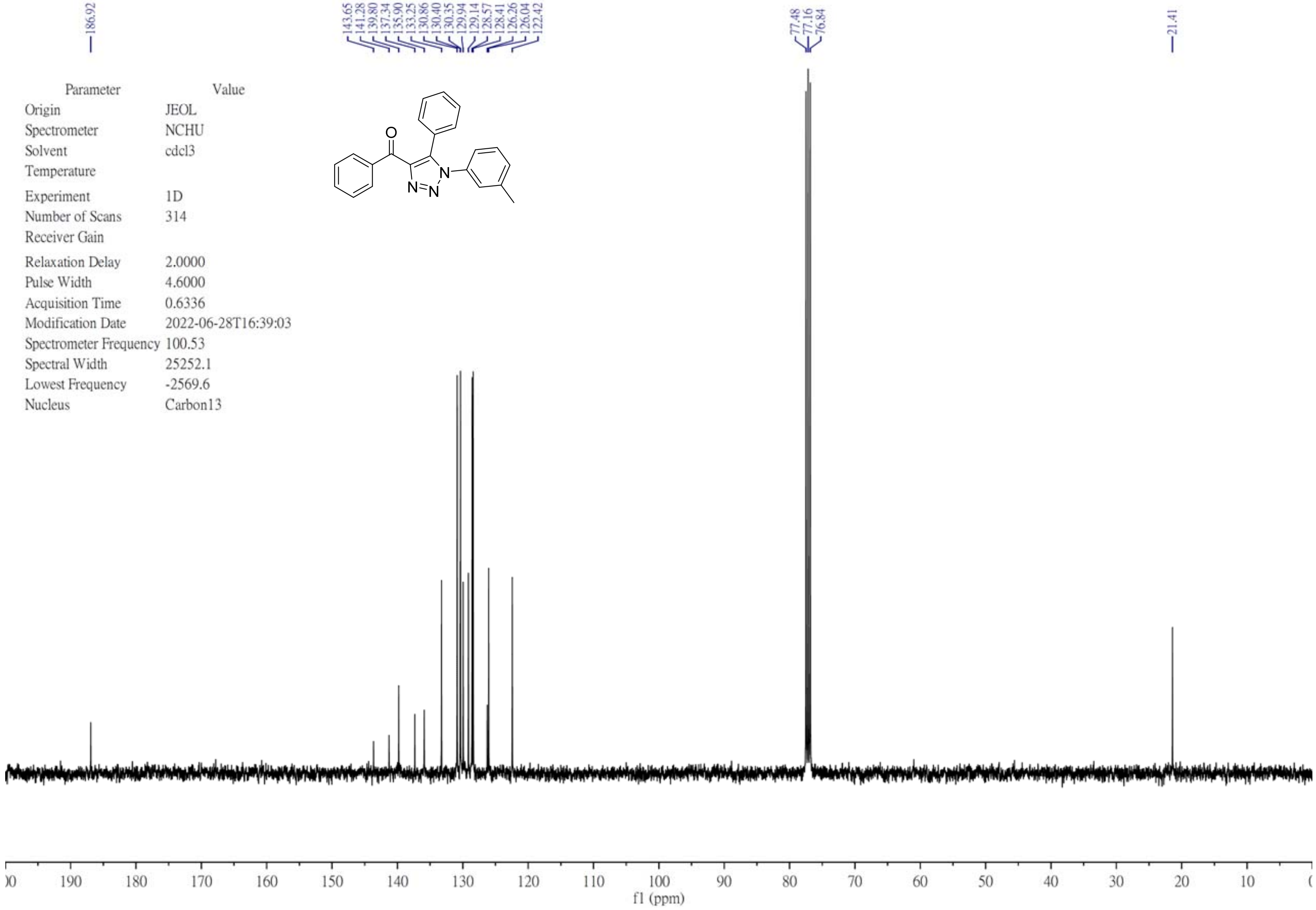

5ac $^{13}C\{^{1}H\}$ NMR spectrum (100 MHz in $CDCl_3$)

Parameter	Value
Origin	Varian
Spectrometer	vnmrs
Solvent	"cdcl3"
Temperature	20.0
Pulse Sequence	s2pul
Experiment	1D
Number of Scans	4
Receiver Gain	30
Relaxation Delay	1.0000
Pulse Width	0.0000
Acquisition Time	2.5559
Modification Date	2024-01-12T15:03:00
Spectrometer Frequency	399.76
Spectral Width	6410.3
Lowest Frequency	-815.4
Nucleus	1H

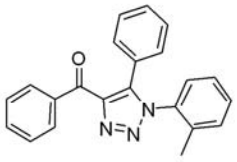

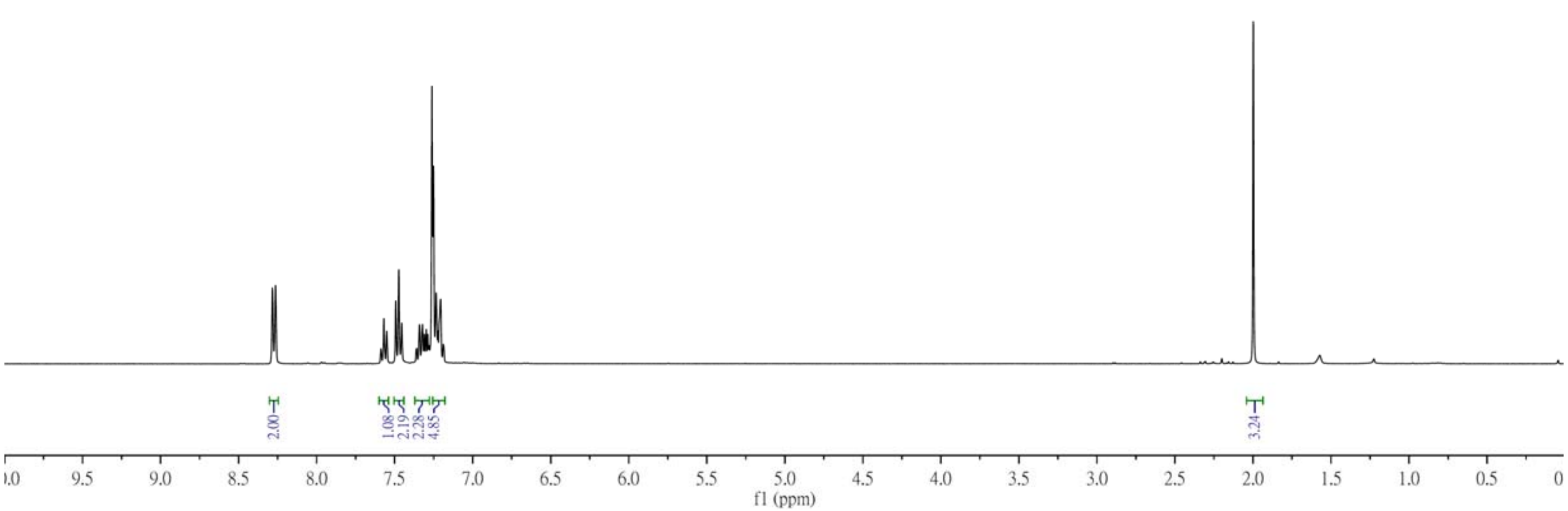

5ad ^{1}H NMR spectrum (400 MHz in $CDCl_3$)

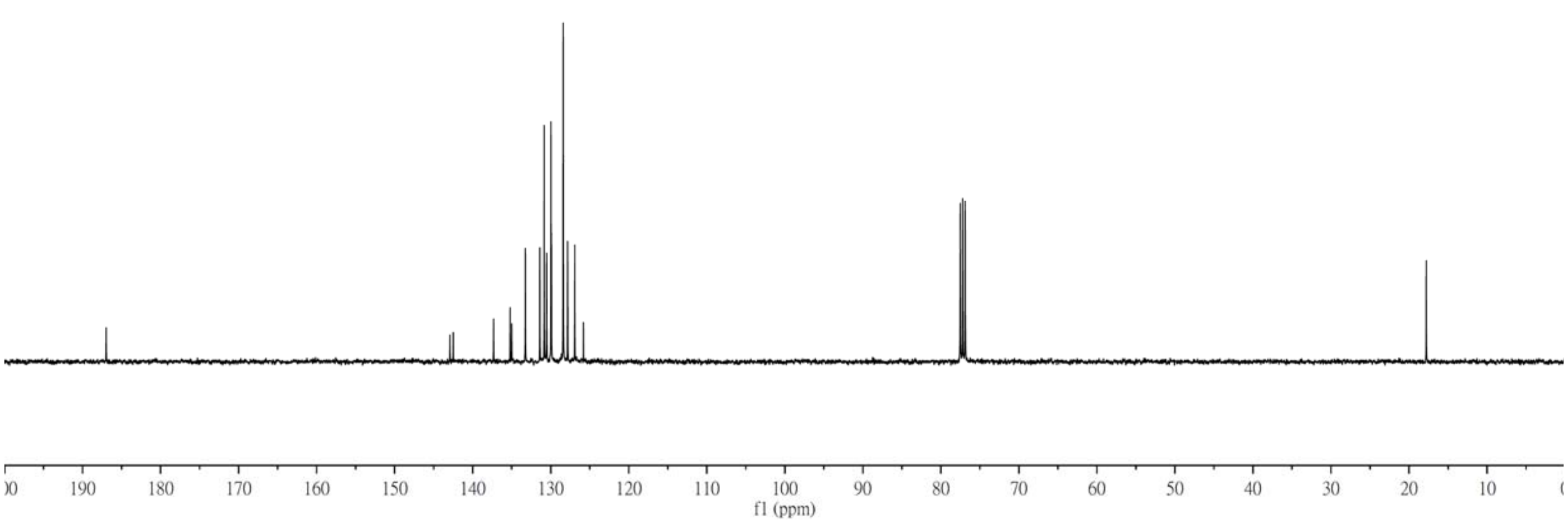

Parameter	Value
Origin	JEOL
Spectrometer	NCHU
Solvent	"$CDCl_3$"
Temperature	
Experiment	1D
Number of Scans	83
Receiver Gain	
Relaxation Delay	2.0000
Pulse Width	4.8000
Acquisition Time	0.7920
Modification Date	2024-01-15T10:27:28
Spectrometer Frequency	100.53
Spectral Width	25252.1
Lowest Frequency	-2576.7
Nucleus	Carbon13

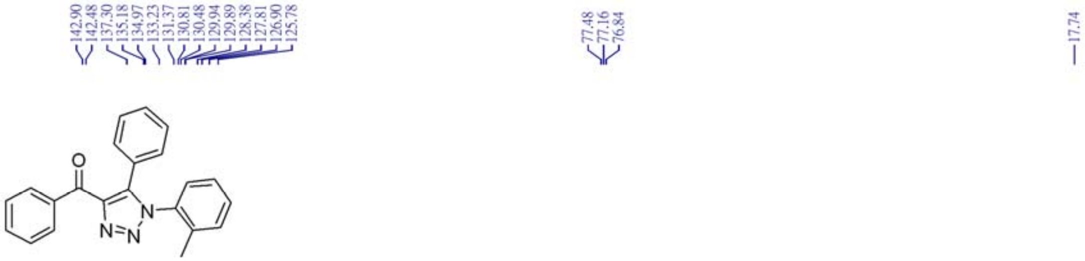

5ad $^{13}C\{^1H\}$ NMR spectrum (100 MHz in $CDCl_3$)

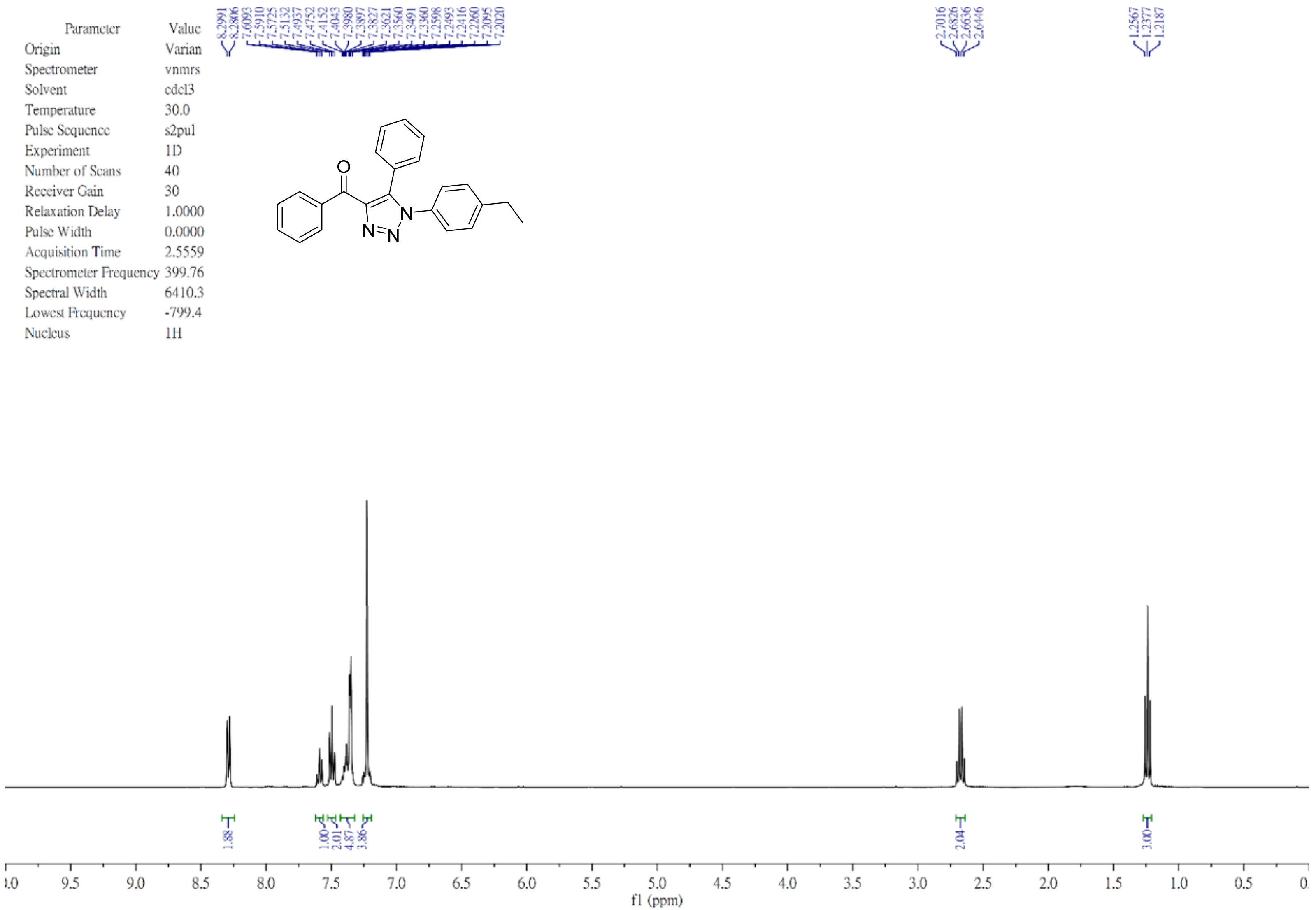

5ae 1H NMR spectrum (400 MHz in $CDCl_3$)

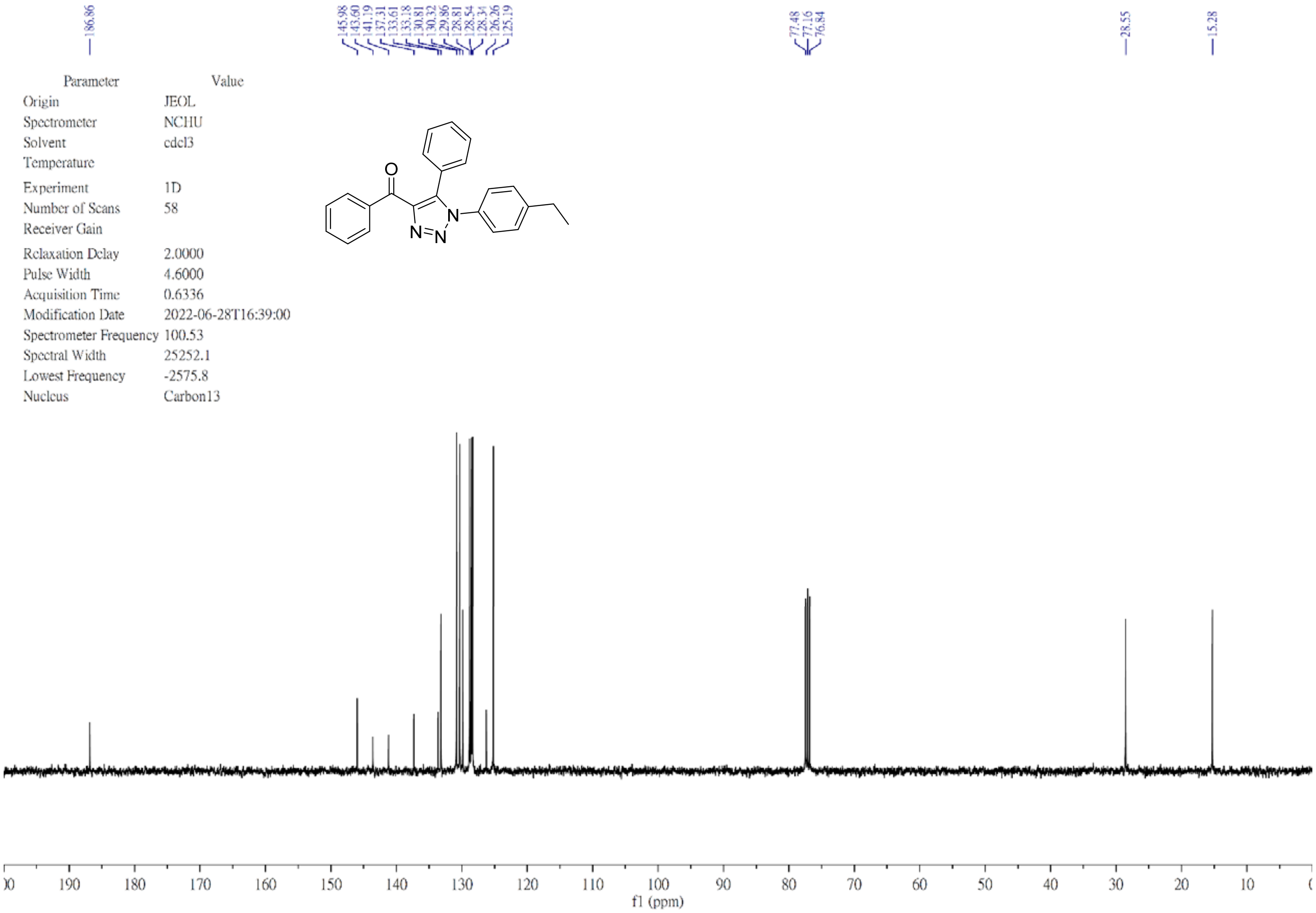

5ae $^{13}C\{^{1}H\}$ NMR spectrum (100 MHz in $CDCl_3$)

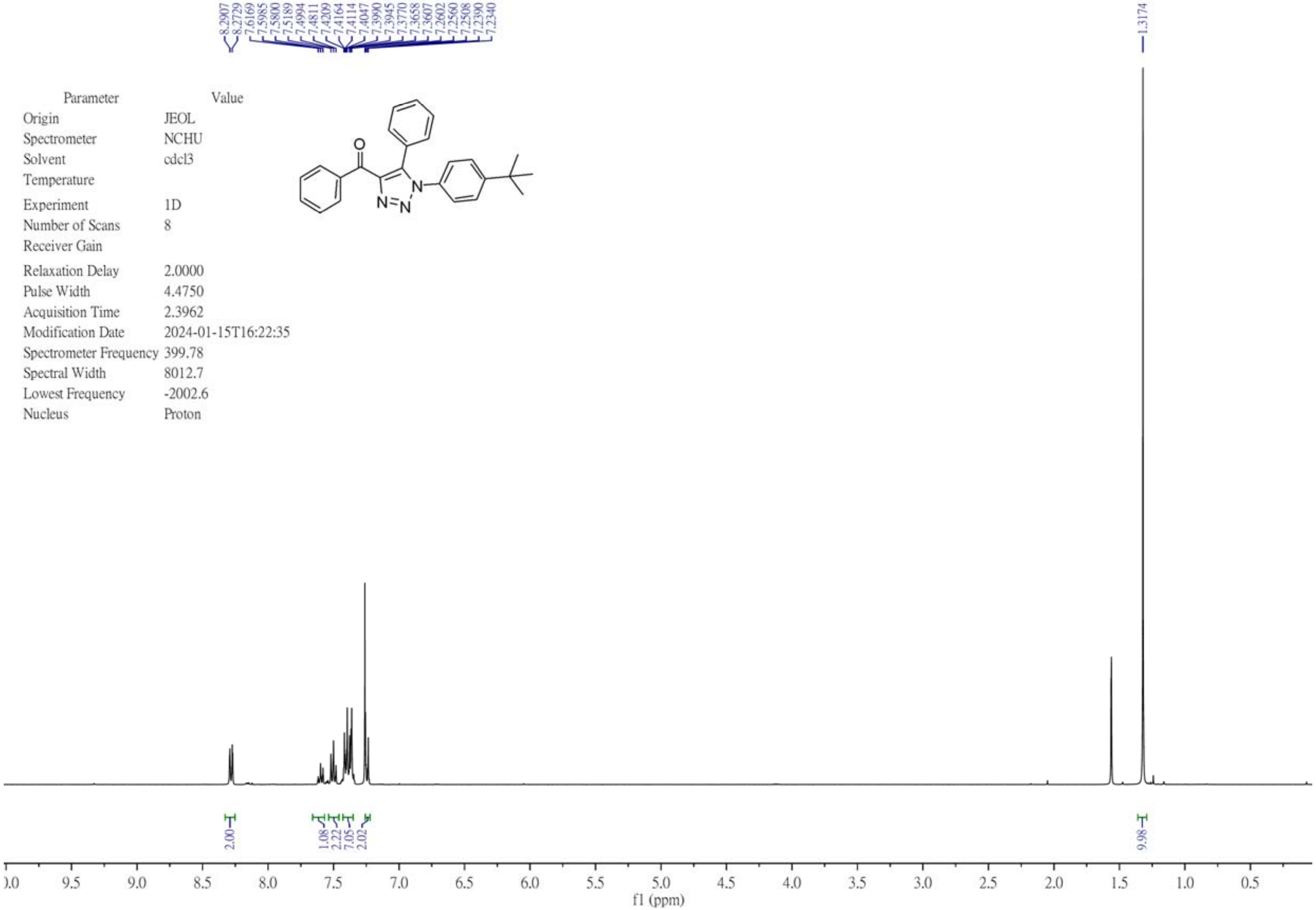

5af ^{1}H NMR spectrum (400 MHz in $CDCl_3$)

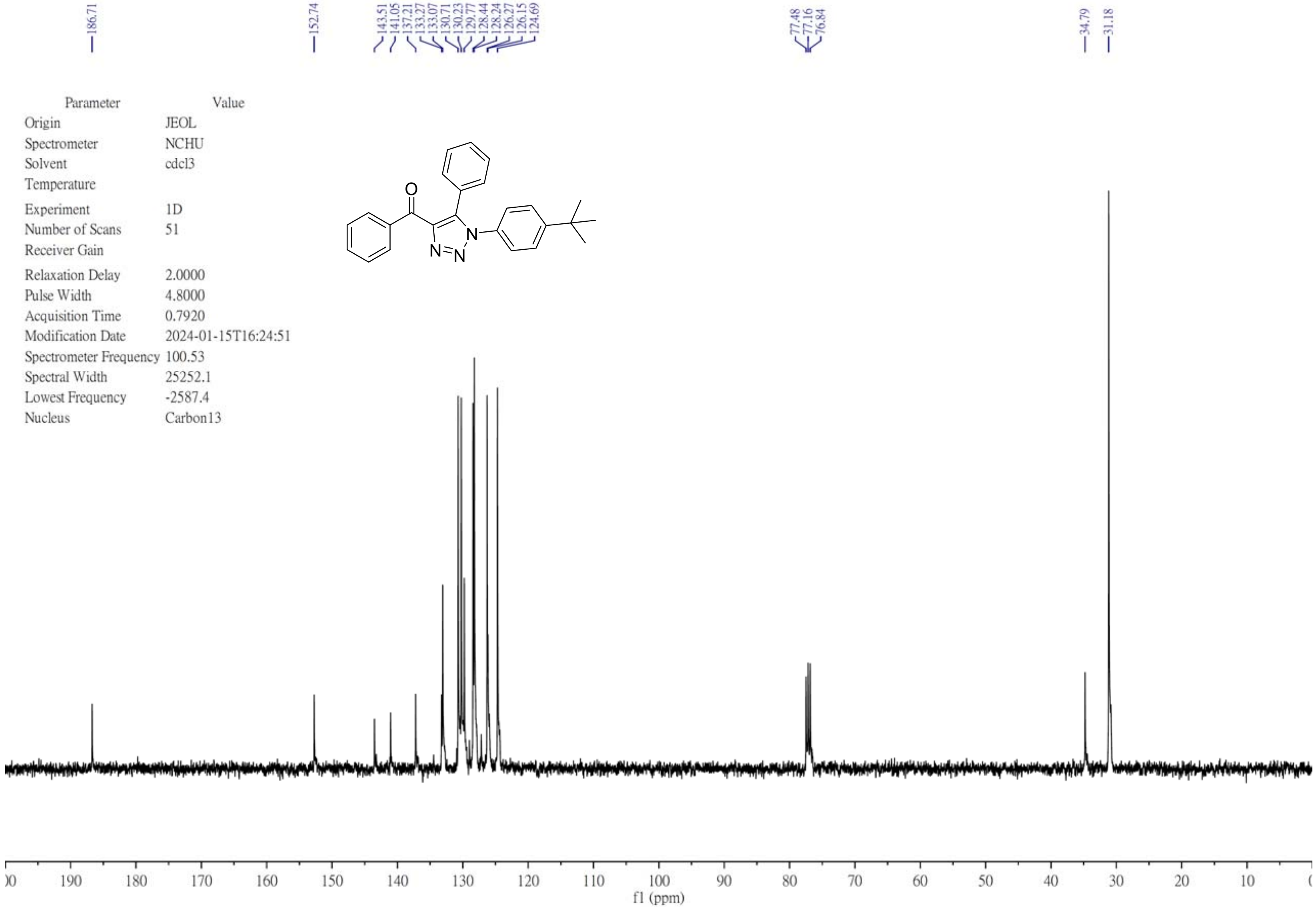

5af $^{13}C\{^{1}H\}$ NMR spectrum (100 MHz in $CDCl_3$)

Parameter	Value
Origin	Varian
Spectrometer	vnmrs
Solvent	cdcl3
Temperature	20.0
Pulse Sequence	s2pul
Experiment	1D
Number of Scans	12
Receiver Gain	46
Relaxation Delay	1.0000
Pulse Width	0.0000
Acquisition Time	2.5559
Spectrometer Frequency	399.76
Spectral Width	6410.3
Lowest Frequency	-799.6
Nucleus	1H

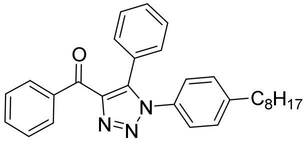

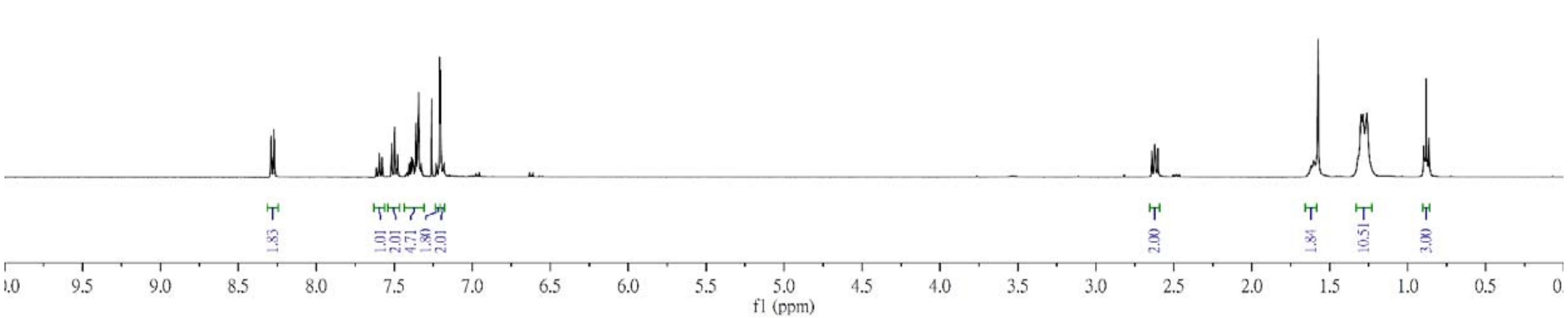

5ag ^{1}H NMR spectrum (400 MHz in $CDCl_3$)

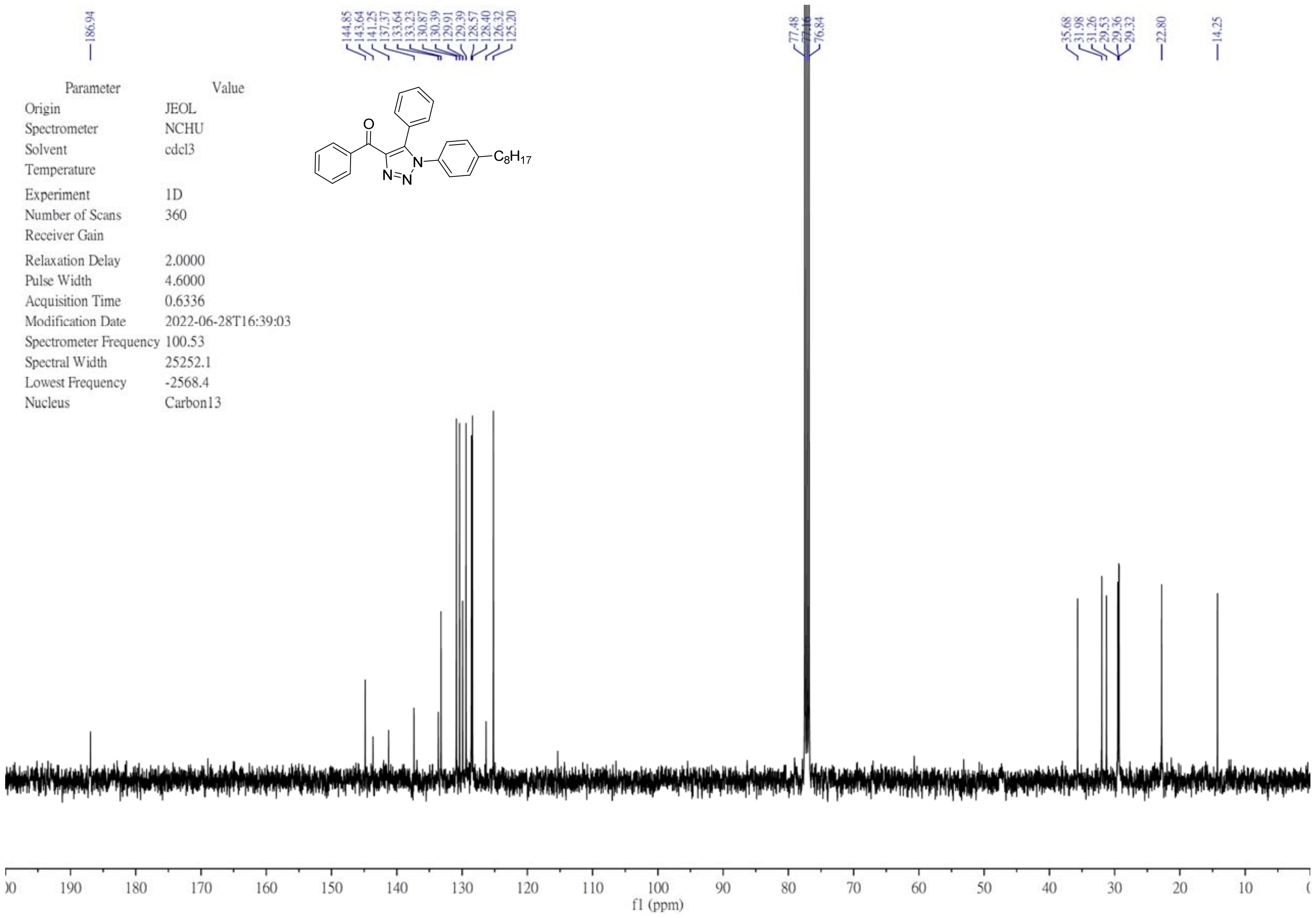

5ag $^{13}C\{^{1}H\}$ NMR spectrum (100 MHz in $CDCl_3$)

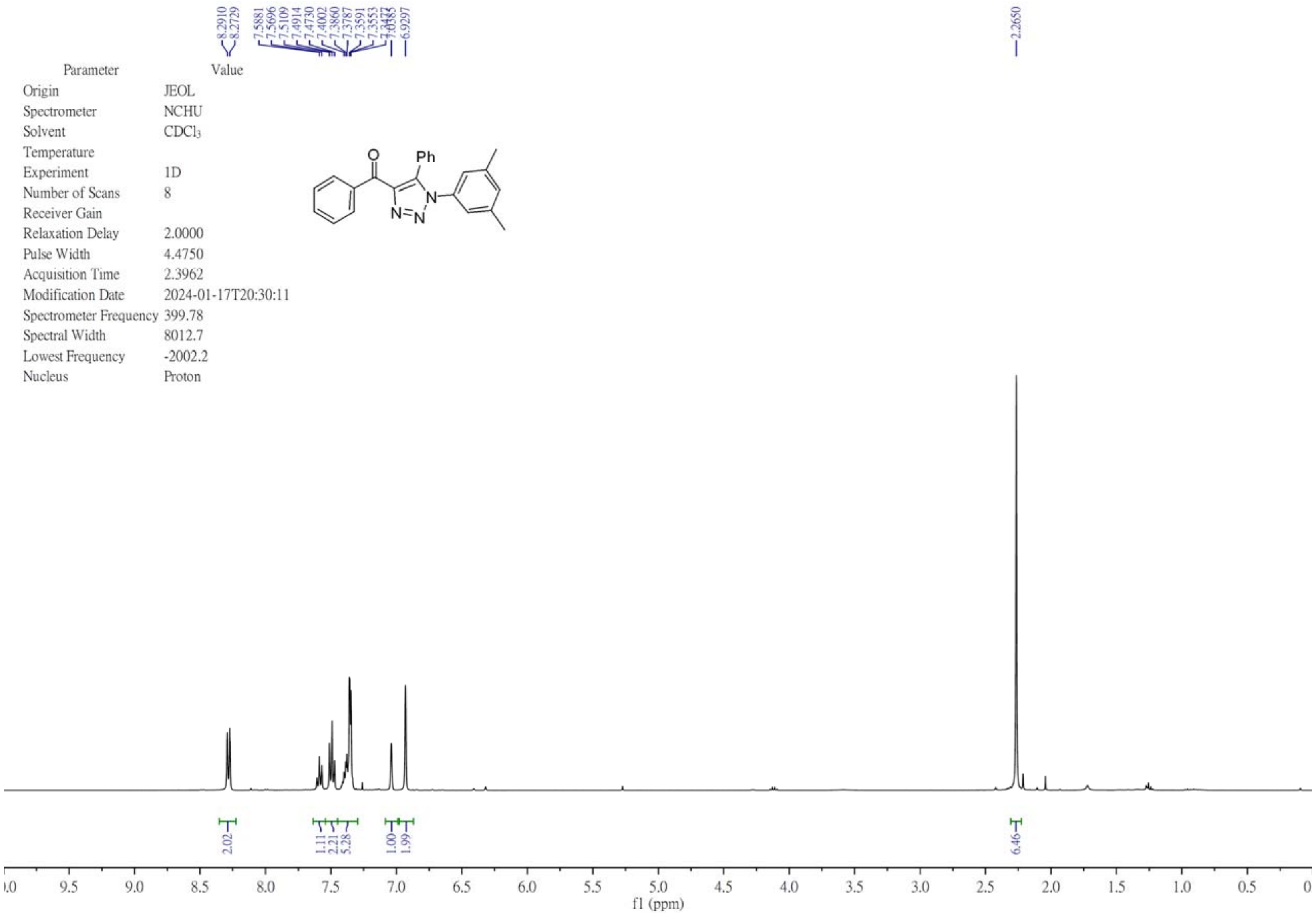

5ah ^{1}H NMR spectrum (400 MHz in $CDCl_3$)

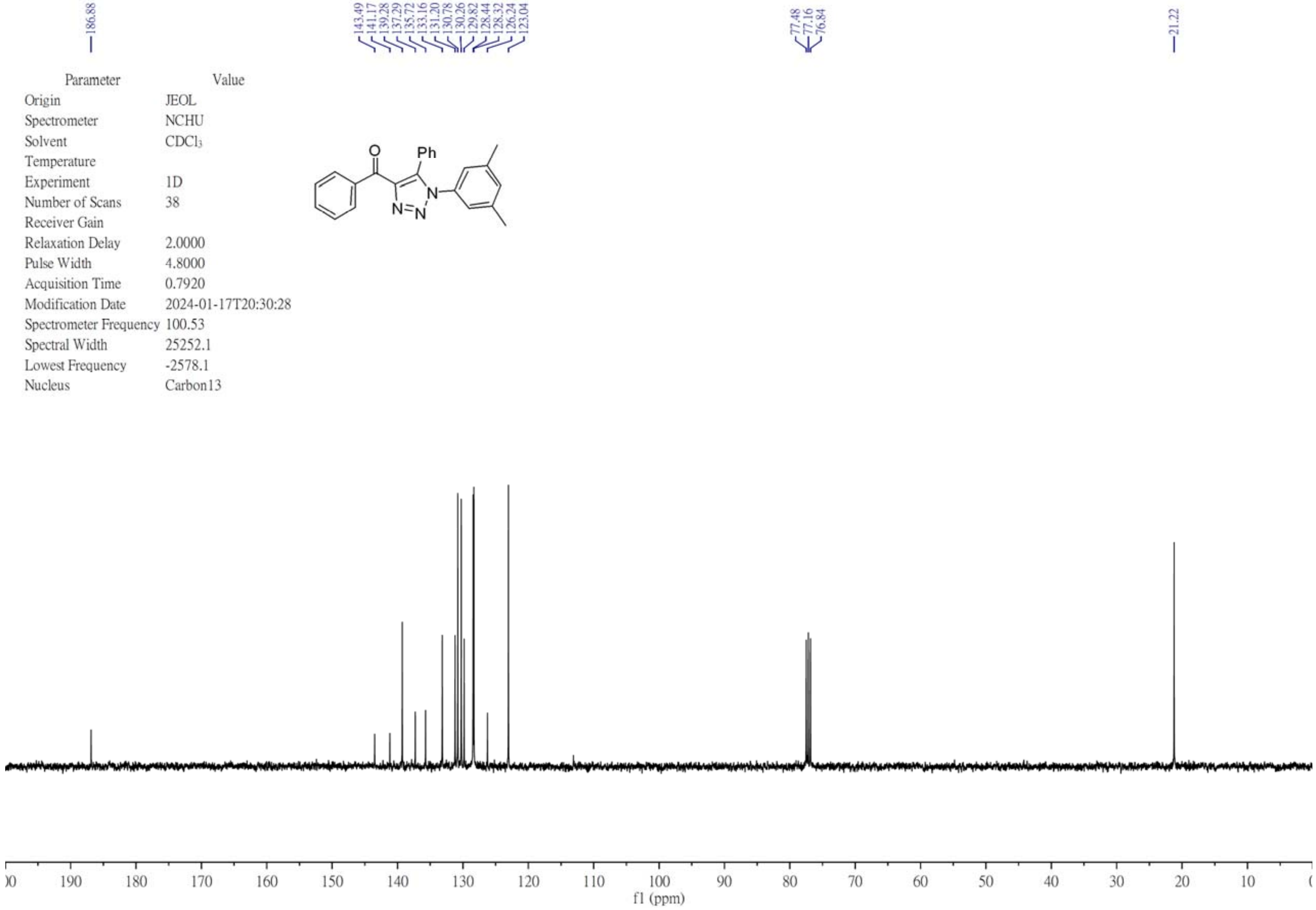

5ah $^{13}C\{^{1}H\}$ NMR spectrum (100 MHz in $CDCl_3$)

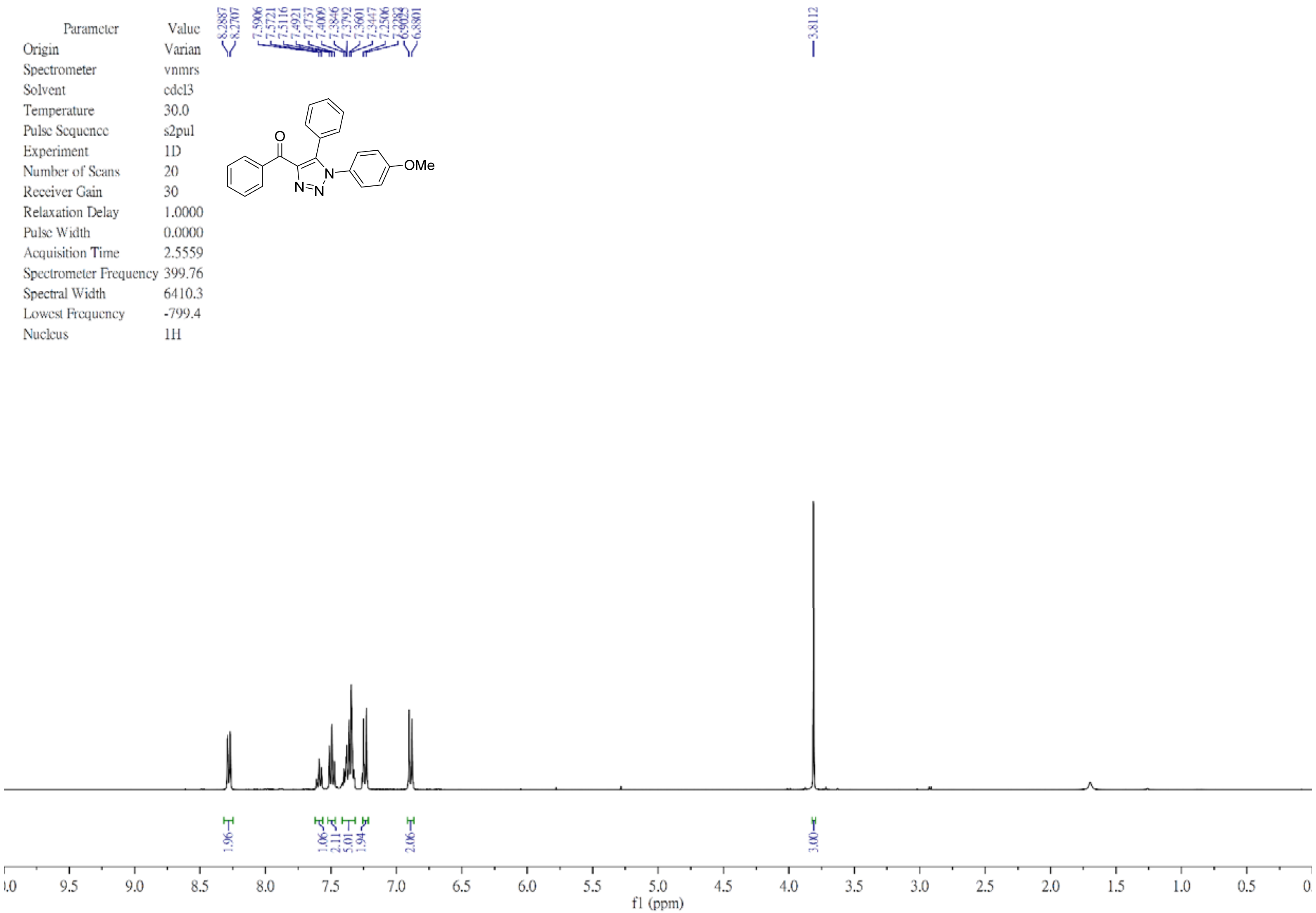

5ai ^{1}H NMR spectrum (400 MHz in $CDCl_3$)

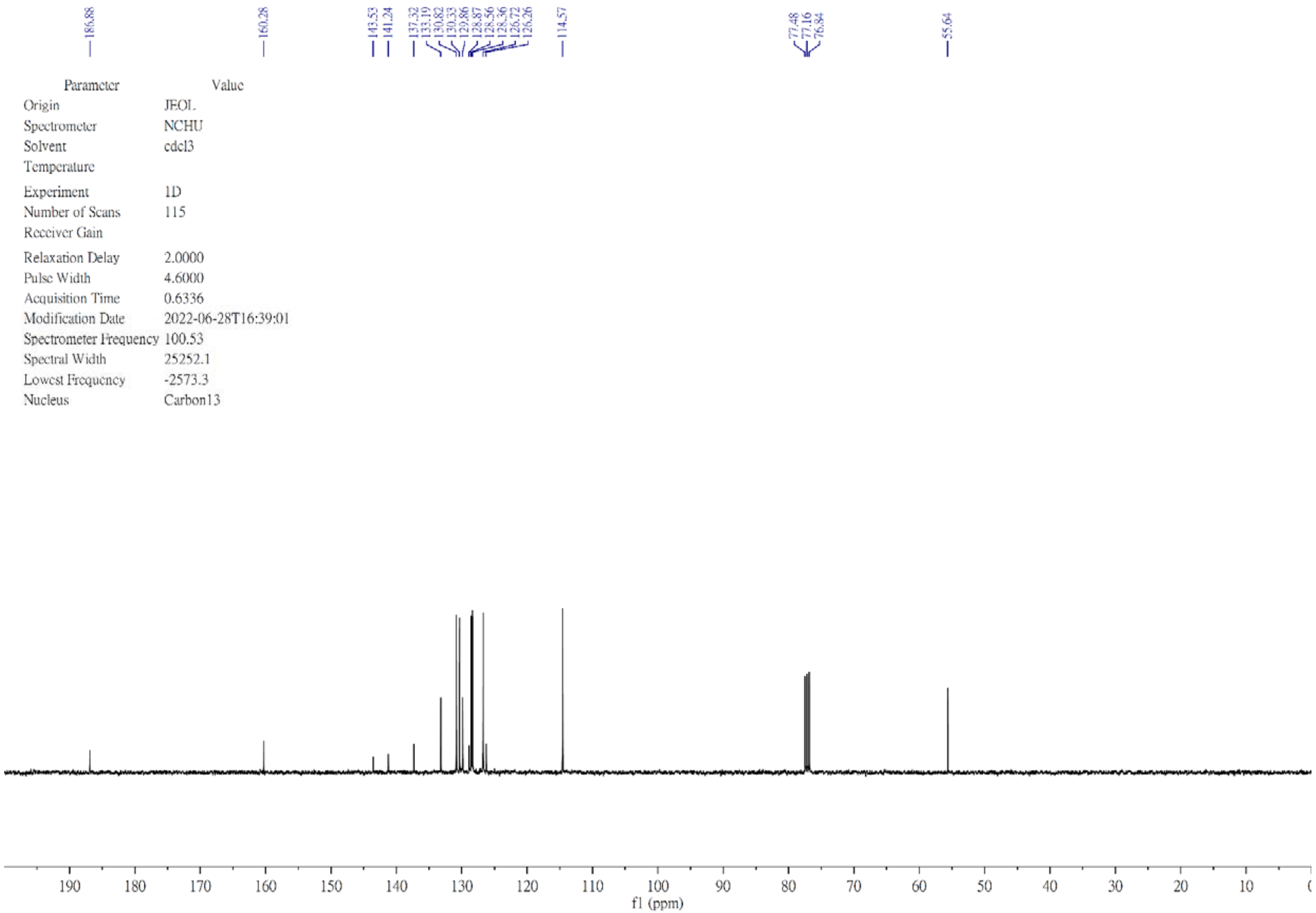

5ai $^{13}C\{^{1}H\}$ NMR spectrum (100 MHz in $CDCl_3$)

Parameter	Value
Origin	Varian
Spectrometer	vnmrs
Solvent	cdcl3
Temperature	30.0
Pulse Sequence	s2pul
Experiment	1D
Number of Scans	16
Receiver Gain	30
Relaxation Delay	1.0000
Pulse Width	0.0000
Acquisition Time	2.5559
Spectrometer Frequency	399.76
Spectral Width	6410.3
Lowest Frequency	-799.2
Nucleus	1H

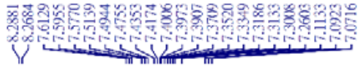

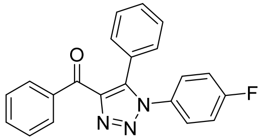

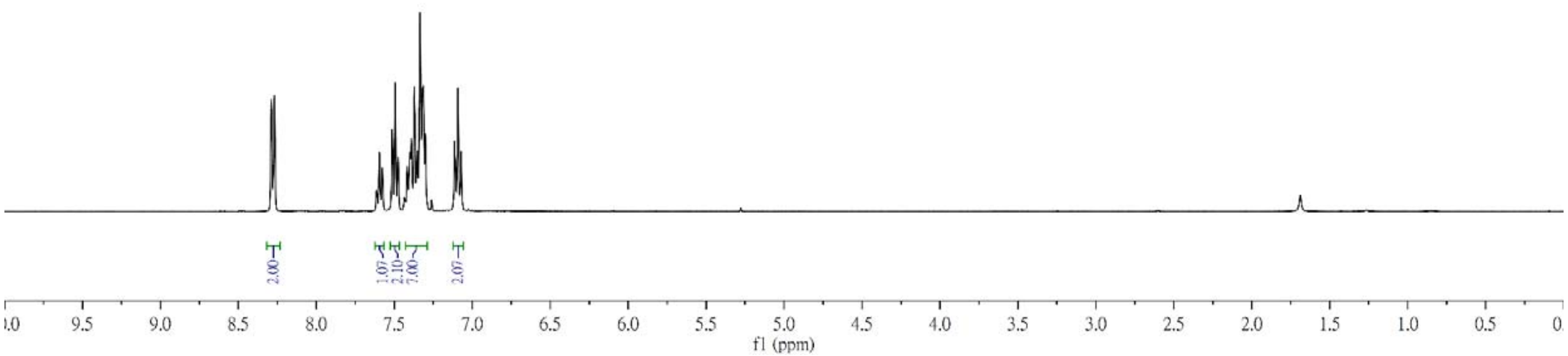

5aj ^{1}H NMR spectrum (400 MHz in $CDCl_3$)

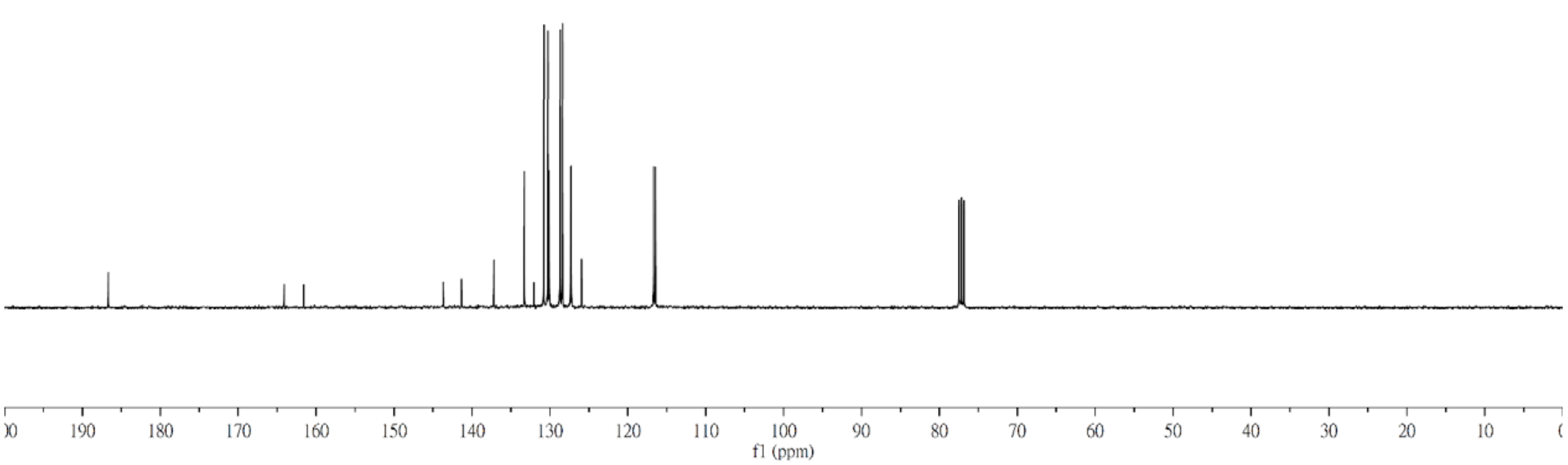

Parameter	Value
Origin	JEOL
Spectrometer	NCHU
Solvent	cdcl3
Temperature	
Experiment	1D
Number of Scans	300
Receiver Gain	
Relaxation Delay	2.0000
Pulse Width	4.6000
Acquisition Time	0.6336
Modification Date	2022-06-28T16:39:00
Spectrometer Frequency	100.53
Spectral Width	25252.1
Lowest Frequency	-2575.1
Nucleus	Carbon13

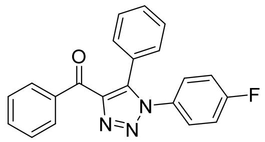

5aj $^{13}C\{^1H\}$ NMR spectrum (100 MHz in $CDCl_3$)

Parameter	Value
Origin	Varian
Spectrometer	vnmrs
Solvent	cdcl3
Temperature	30.0
Pulse Sequence	s2pul
Experiment	1D
Number of Scans	20
Receiver Gain	54
Relaxation Delay	1.0000
Pulse Width	0.0000
Acquisition Time	2.5559
Spectrometer Frequency	399.76
Spectral Width	6410.3
Lowest Frequency	-799.6
Nucleus	1H

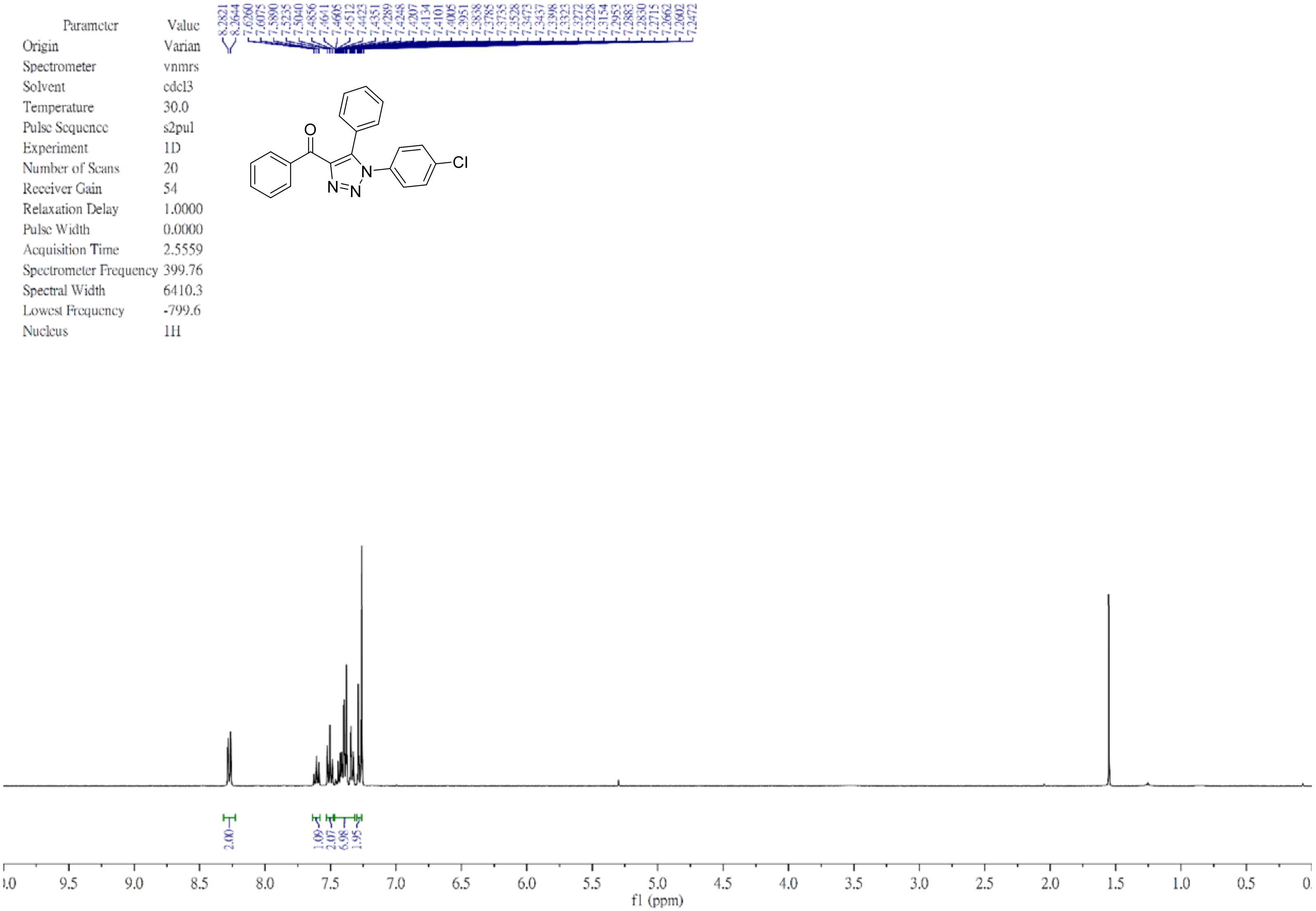

5ak ^{1}H NMR spectrum (400 MHz in $CDCl_3$)

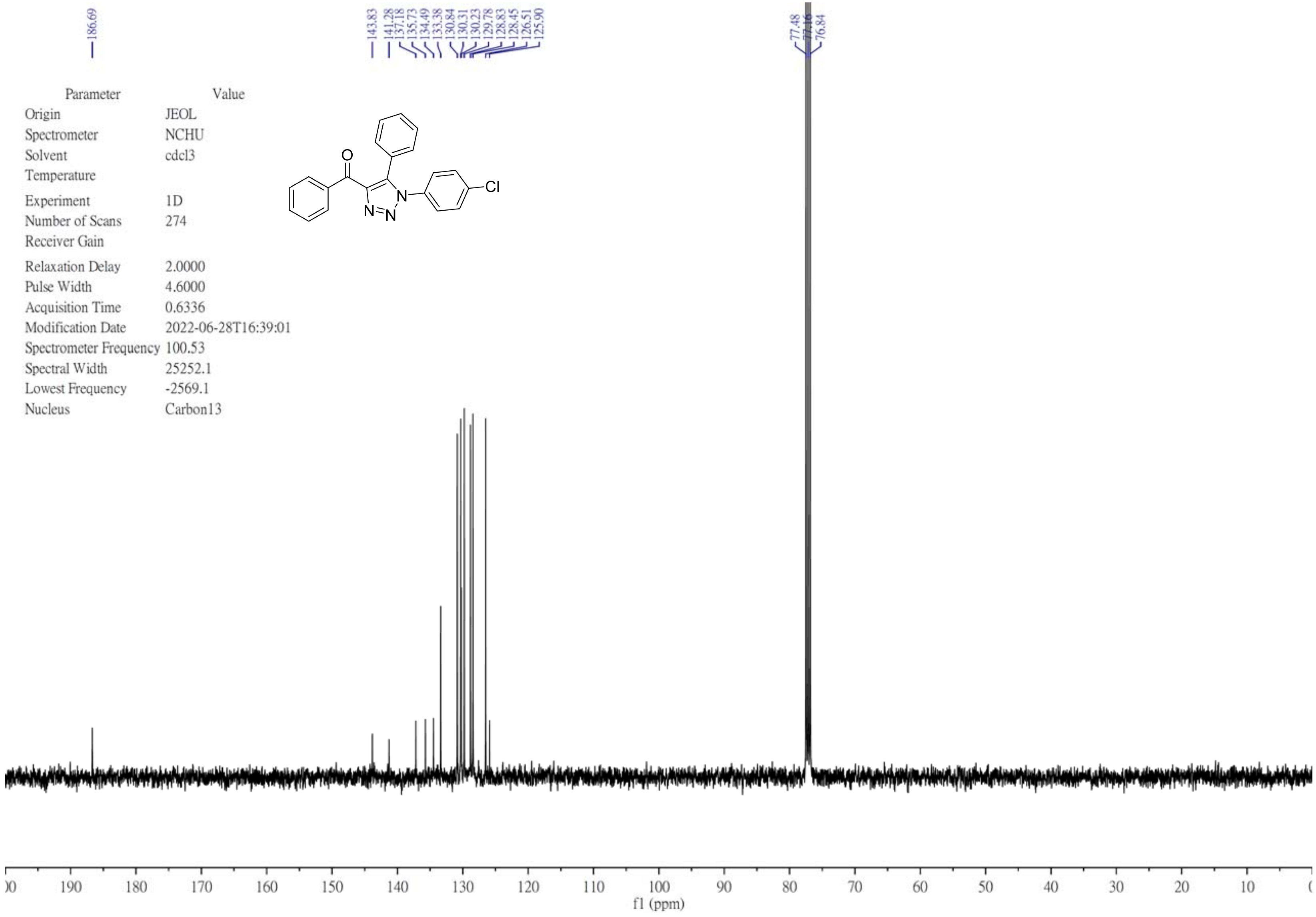

5ak $^{13}C\{^1H\}$ NMR spectrum (100 MHz in $CDCl_3$)

Parameter	Value
Origin	Varian
Spectrometer	vnmrs
Solvent	cdcl3
Temperature	30.0
Pulse Sequence	s2pul
Experiment	1D
Number of Scans	12
Receiver Gain	30
Relaxation Delay	1.0000
Pulse Width	0.0000
Acquisition Time	2.5559
Spectrometer Frequency	399.76
Spectral Width	6410.3
Lowest Frequency	-800.2
Nucleus	1H

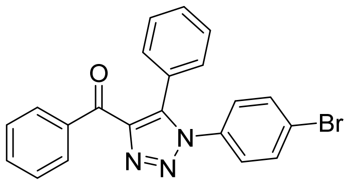

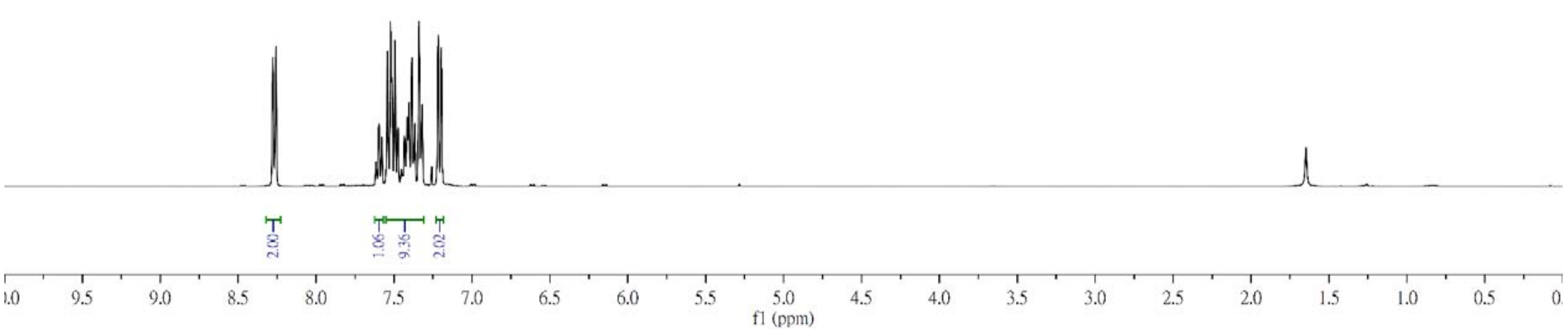

5al ^{1}H NMR spectrum (400 MHz in $CDCl_3$)

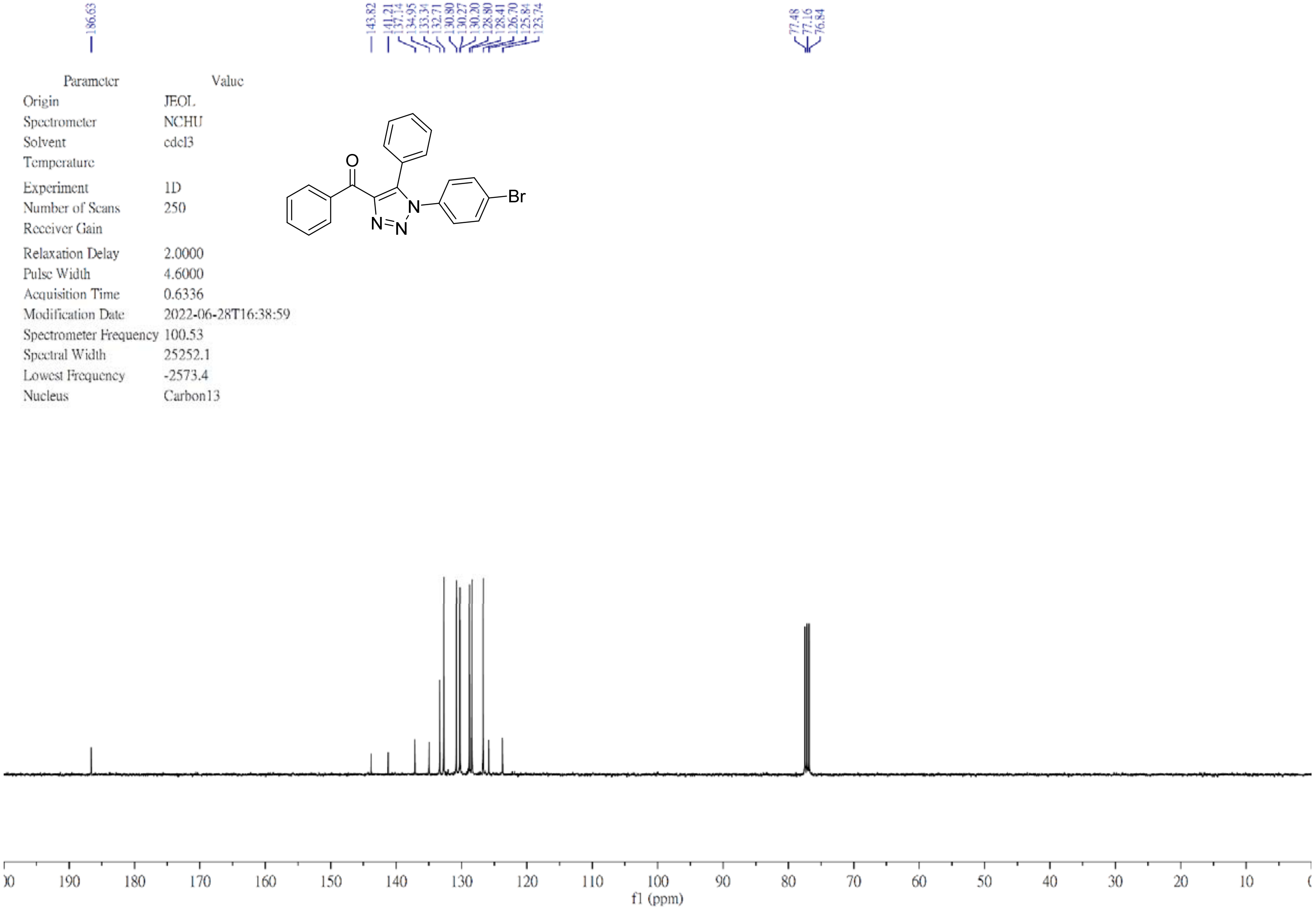

5al $^{13}C\{^{1}H\}$ NMR spectrum (100 MHz in $CDCl_3$)

Parameter	Value
Origin	Varian
Spectrometer	vnmrs
Solvent	cdcl3
Temperature	30.0
Pulse Sequence	s2pul
Experiment	1D
Number of Scans	12
Receiver Gain	30
Relaxation Delay	1.0000
Pulse Width	0.0000
Acquisition Time	2.5559
Spectrometer Frequency	399.76
Spectral Width	6410.3
Lowest Frequency	-798.4
Nucleus	1H

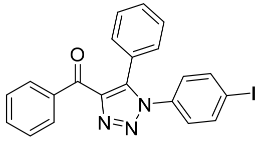

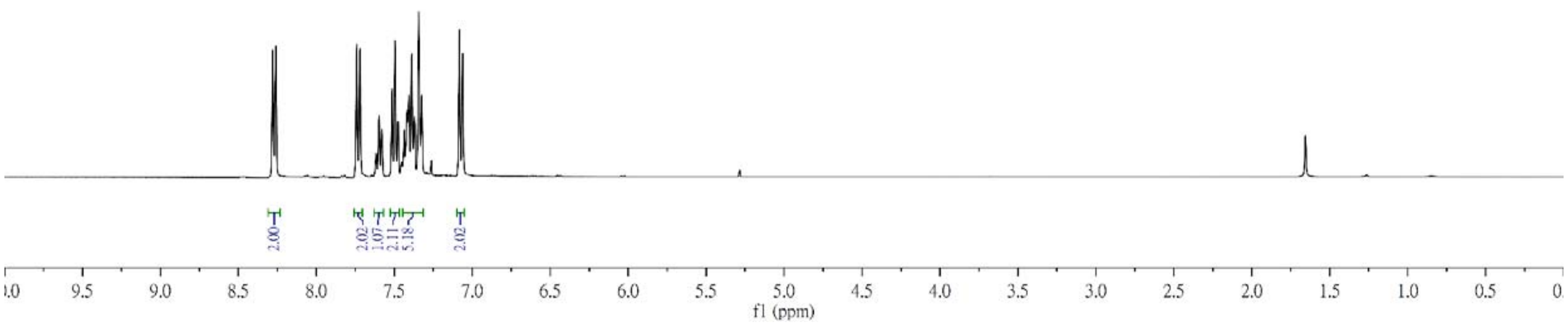

5am ^{1}H NMR spectrum (400 MHz in $CDCl_3$)

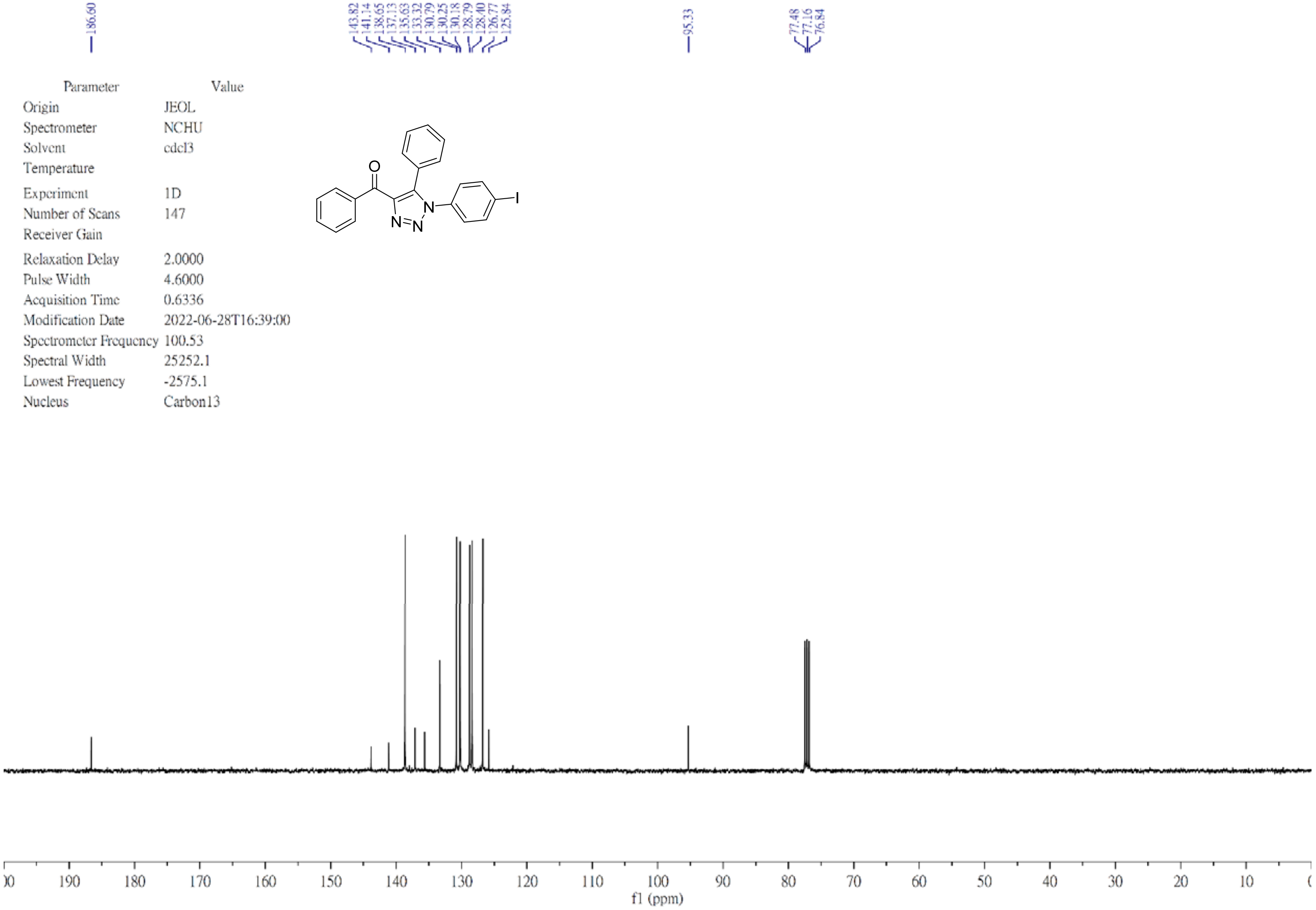

5am $^{13}C\{^{1}H\}$ NMR spectrum (100 MHz in $CDCl_3$)

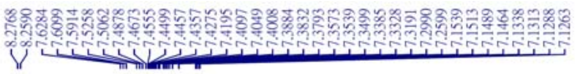

Parameter	Value
Origin	Varian
Spectrometer	vnmrs
Solvent	"cdcl3"
Temperature	20.0
Pulse Sequence	s2pul
Experiment	1D
Number of Scans	28
Receiver Gain	48
Relaxation Delay	1.0000
Pulse Width	0.0000
Acquisition Time	2.5559
Modification Date	2024-01-04T17:56:00
Spectrometer Frequency	399.76
Spectral Width	6410.3
Lowest Frequency	-800.0
Nucleus	1H

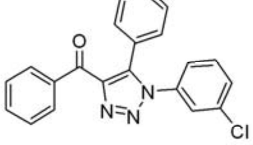

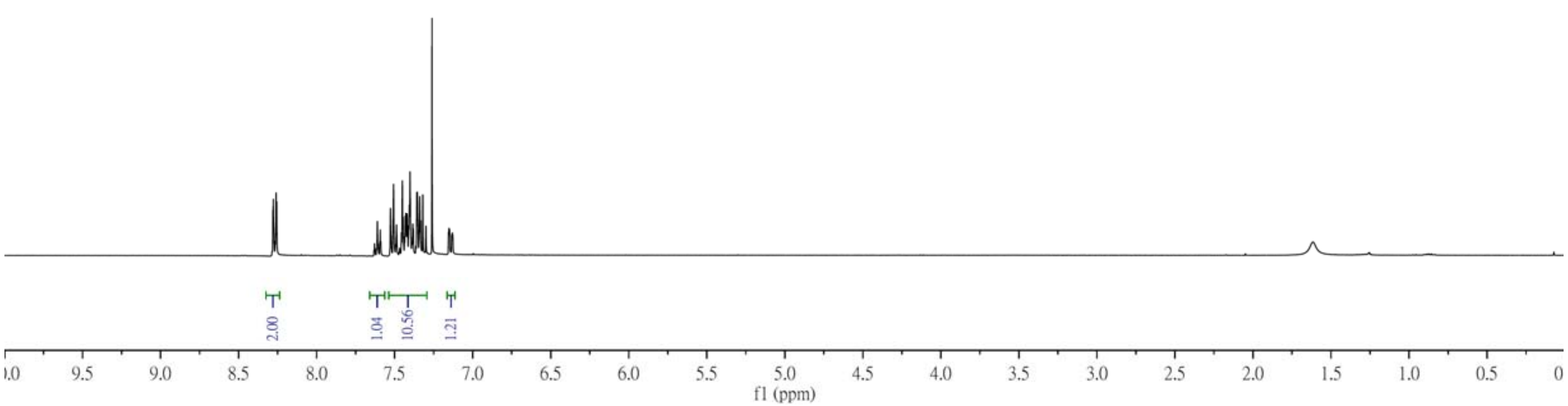

5an ^{1}H NMR spectrum (400 MHz in $CDCl_3$)

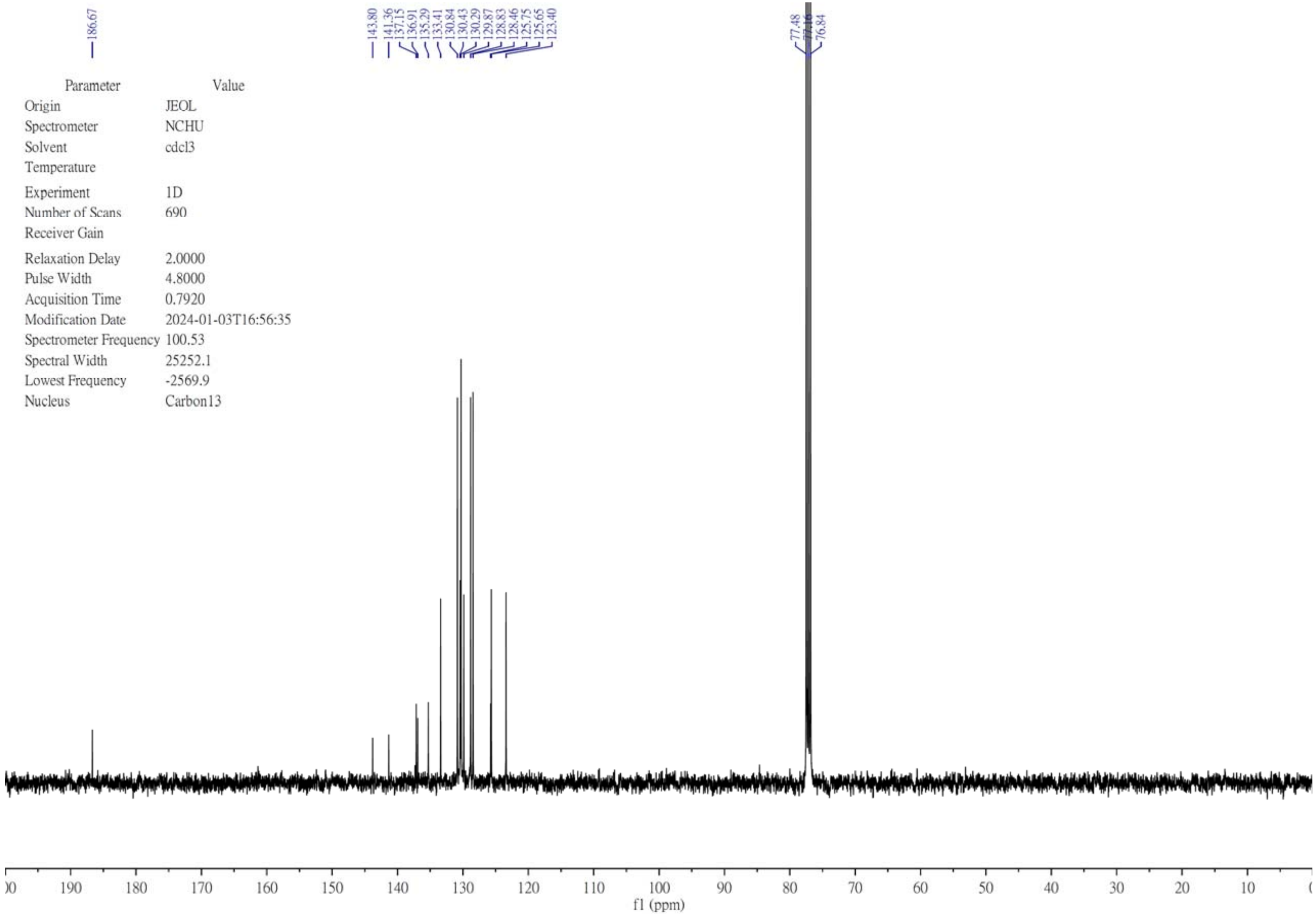

5an $^{13}C\{^{1}H\}$ NMR spectrum (100 MHz in $CDCl_3$)

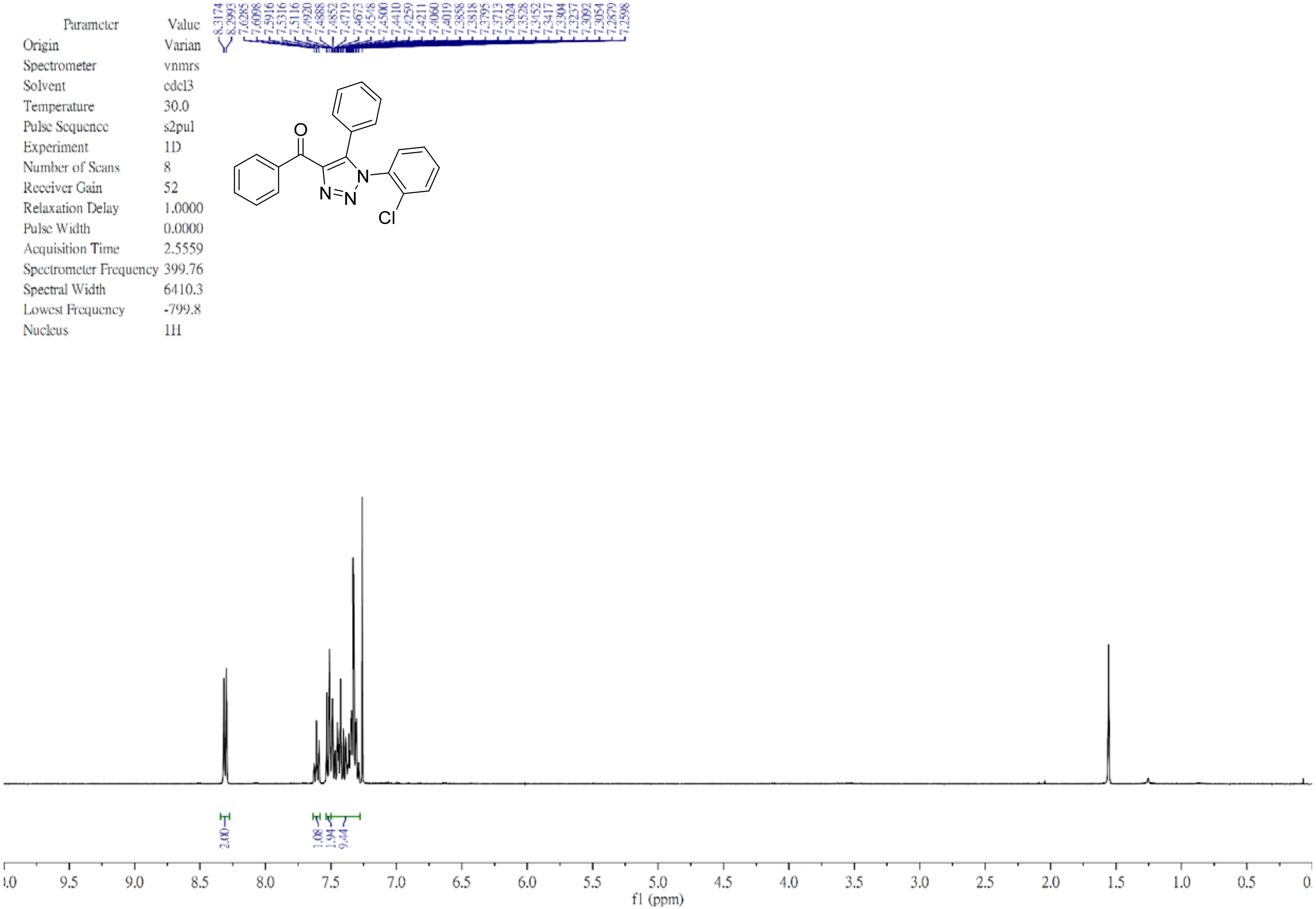

5ao ^{1}H NMR spectrum (400 MHz in $CDCl_3$)

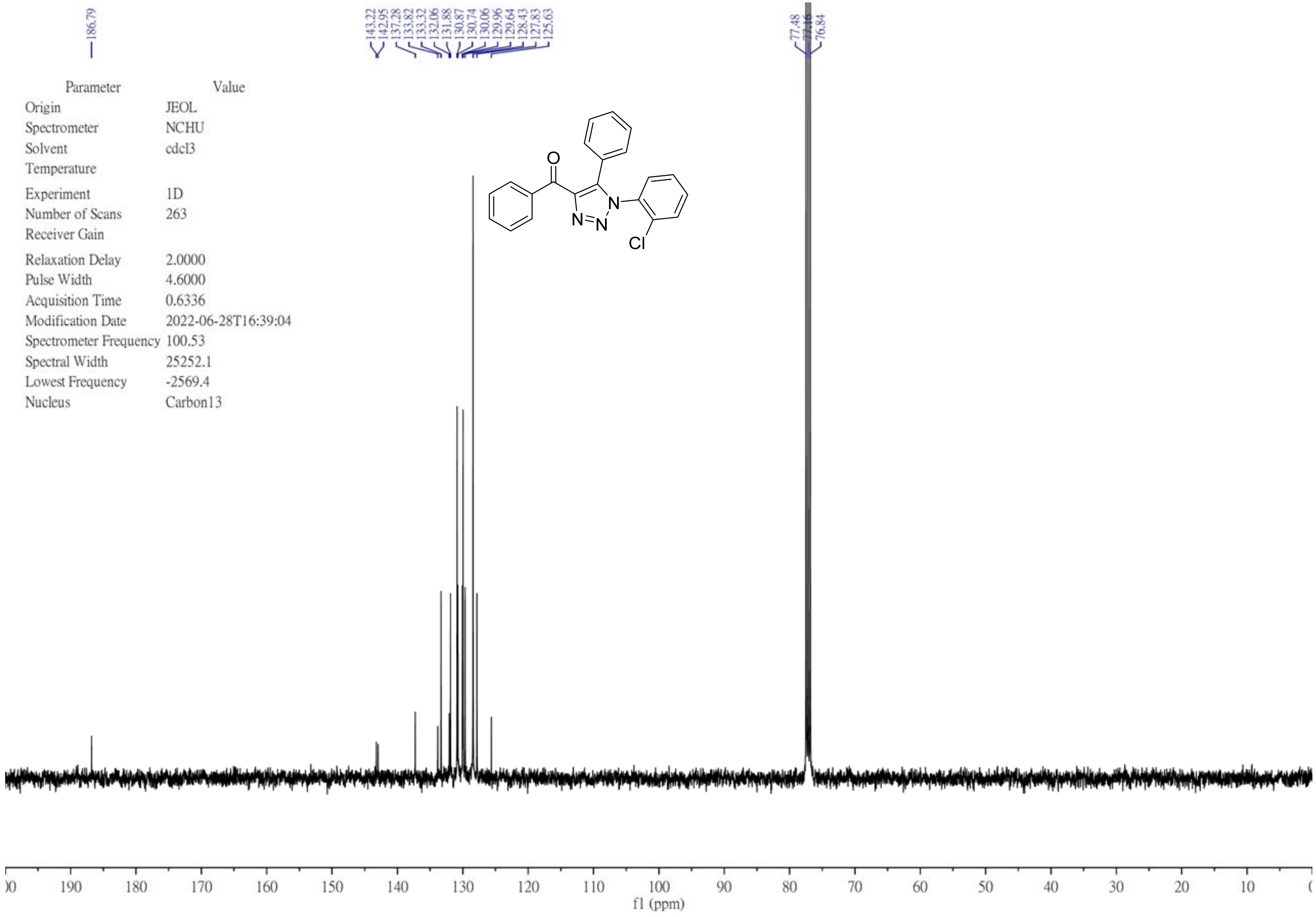

5ao $^{13}C\{^{1}H\}$ NMR spectrum (100 MHz in $CDCl_3$)

Parameter	Value
Origin	Varian
Spectrometer	vnmrs
Solvent	cdcl3
Temperature	30.0
Pulse Sequence	s2pul
Experiment	1D
Number of Scans	16
Receiver Gain	52
Relaxation Delay	1.0000
Pulse Width	0.0000
Acquisition Time	2.5559
Spectrometer Frequency	399.76
Spectral Width	6410.3
Lowest Frequency	-799.0
Nucleus	1H

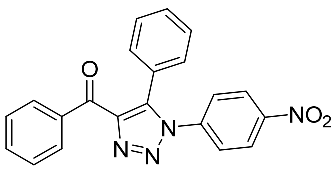

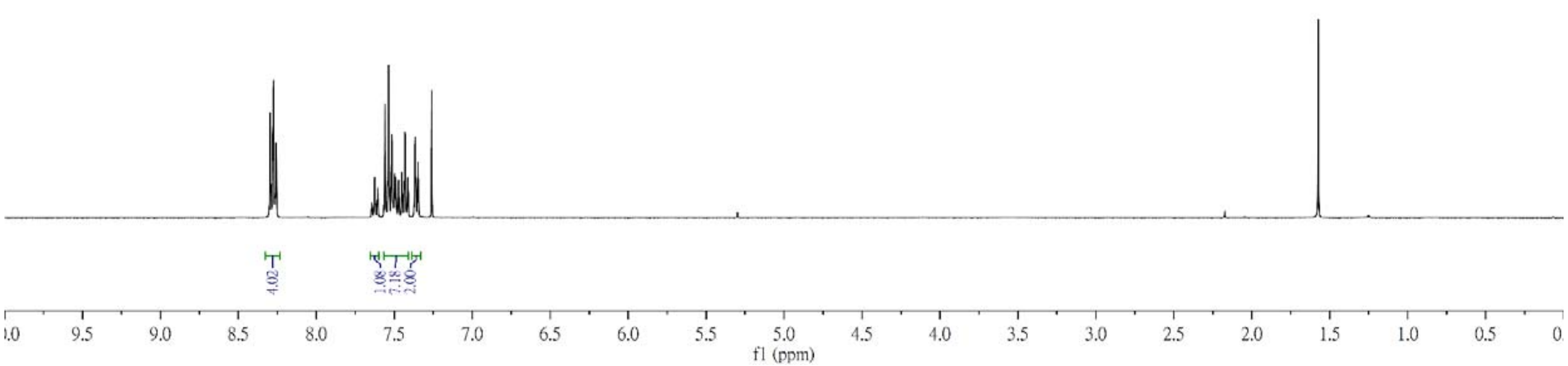

5ap ^{1}H NMR spectrum (400 MHz in $CDCl_3$)

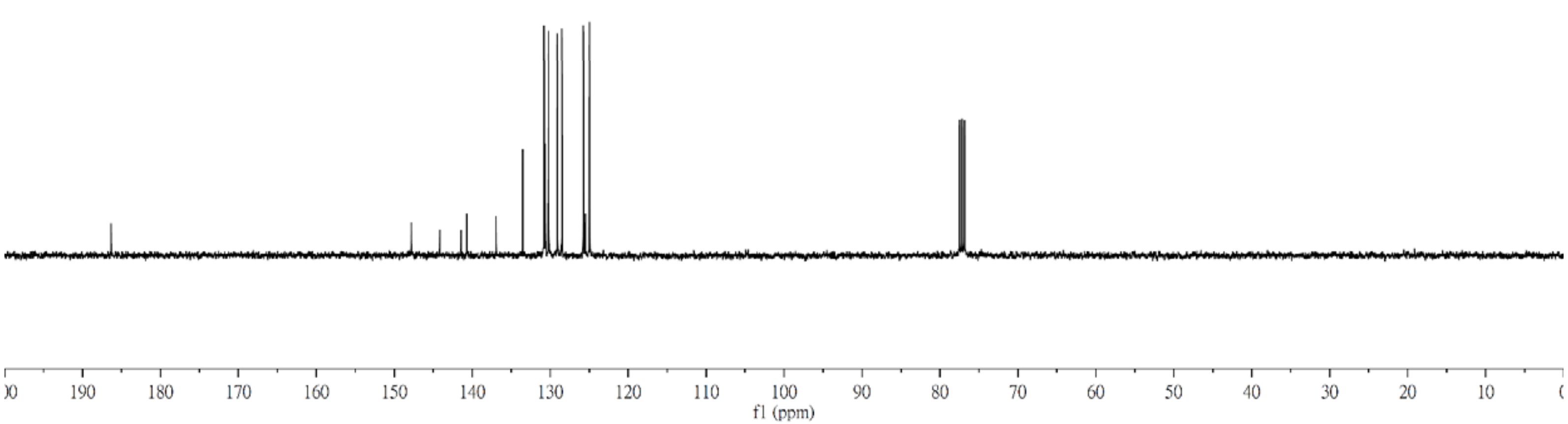

Parameter	Value
Origin	JEOL
Spectrometer	NCHU
Solvent	cdcl3
Temperature	
Experiment	1D
Number of Scans	63
Receiver Gain	
Relaxation Delay	2.0000
Pulse Width	4.6000
Acquisition Time	0.6336
Modification Date	2022-06-30T09:43:52
Spectrometer Frequency	100.53
Spectral Width	25252.1
Lowest Frequency	-2573.2
Nucleus	Carbon13

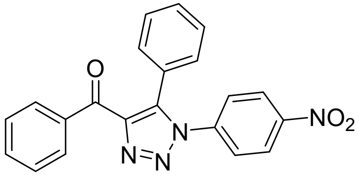

5ap $^{13}C\{^1H\}$ NMR spectrum (100 MHz in $CDCl_3$)

Parameter	Value
Origin	Varian
Spectrometer	vnmrs
Solvent	cdcl3
Temperature	30.0
Pulse Sequence	s2pul
Experiment	1D
Number of Scans	12
Receiver Gain	42
Relaxation Delay	1.0000
Pulse Width	0.0000
Acquisition Time	2.5559
Spectrometer Frequency	399.76
Spectral Width	6410.3
Lowest Frequency	-799.6
Nucleus	1H

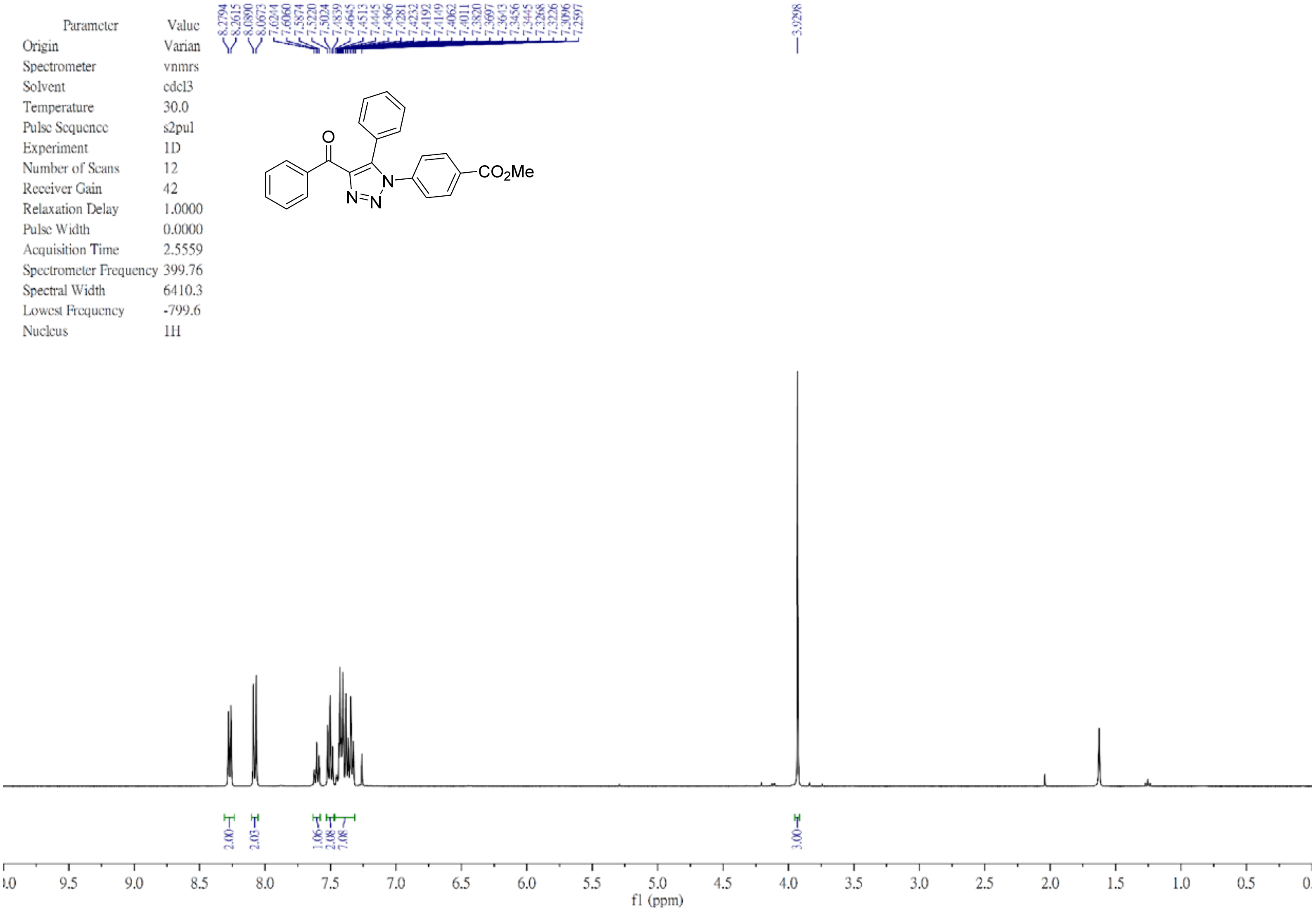

5aq ^{1}H NMR spectrum (400 MHz in $CDCl_3$)

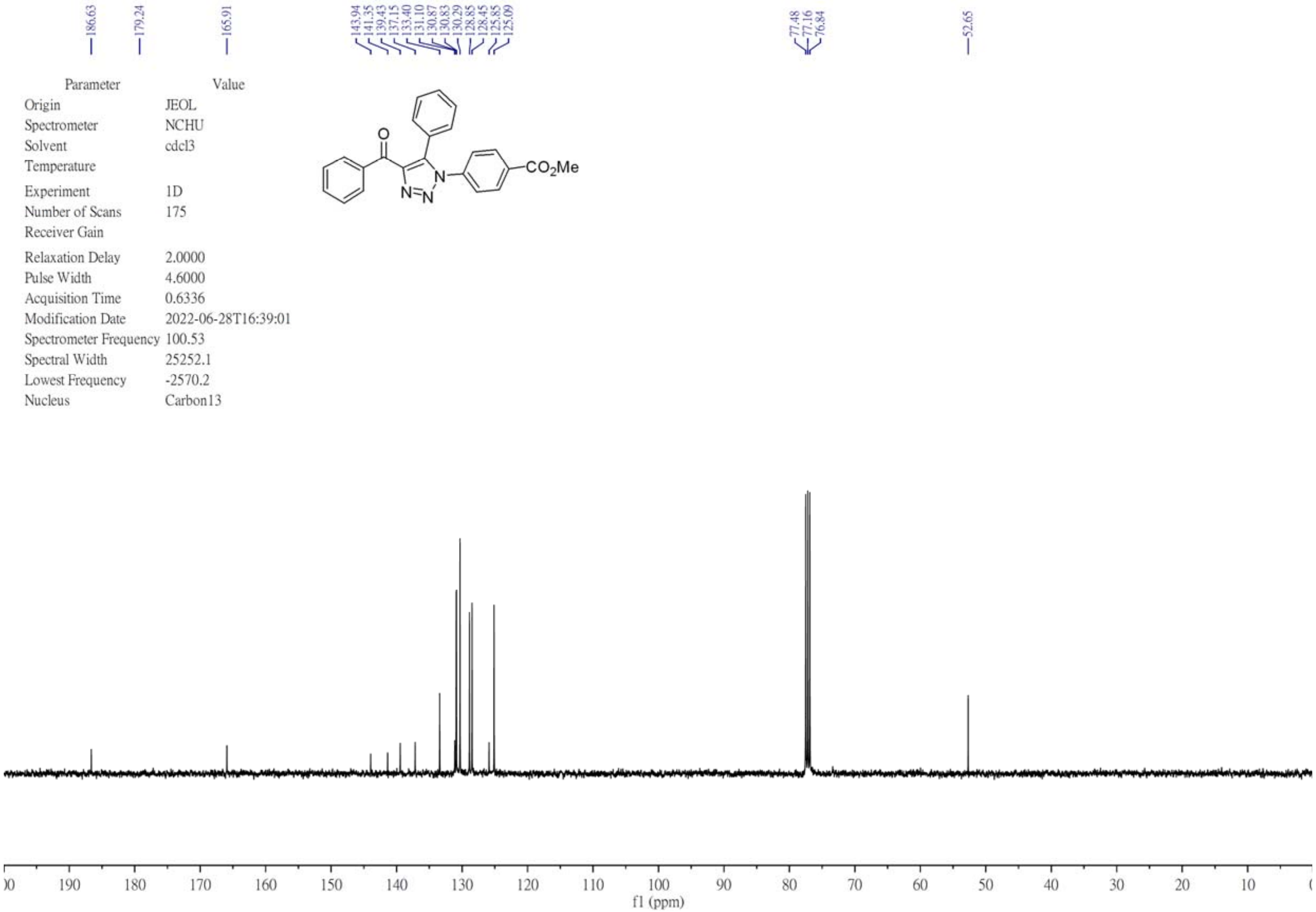

5aq $^{13}C\{^{1}H\}$ NMR spectrum (100 MHz in $CDCl_3$)

Parameter	Value
Origin	Varian
Spectrometer	vnmrs
Solvent	cdcl3
Temperature	30.0
Pulse Sequence	s2pul
Experiment	1D
Number of Scans	12
Receiver Gain	54
Relaxation Delay	1.0000
Pulse Width	0.0000
Acquisition Time	2.5559
Spectrometer Frequency	399.76
Spectral Width	6410.3
Lowest Frequency	-799.4
Nucleus	1H

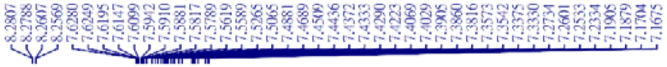

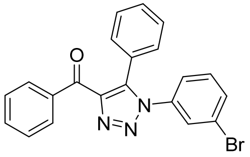

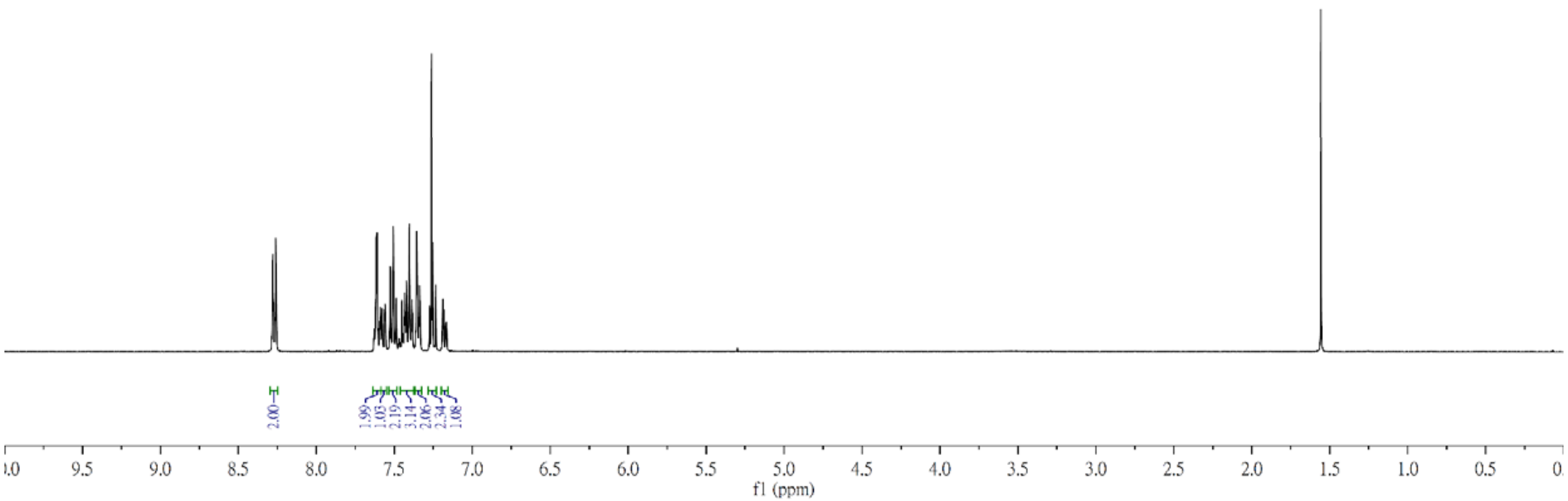

5au ^{1}H NMR spectrum (400 MHz in $CDCl_3$)

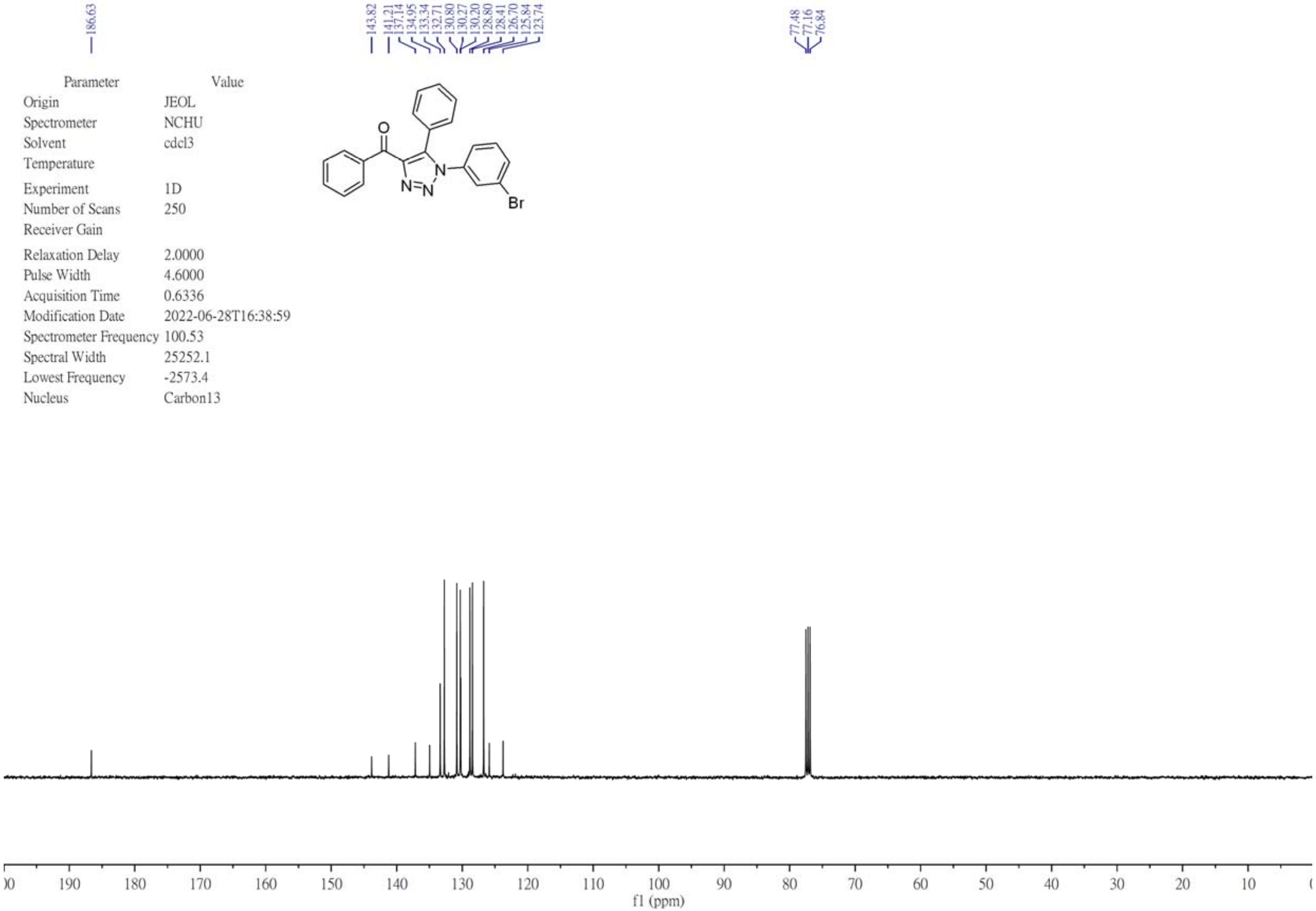

Parameter	Value
Origin	JEOL
Spectrometer	NCHU
Solvent	cdcl3
Temperature	
Experiment	1D
Number of Scans	250
Receiver Gain	
Relaxation Delay	2.0000
Pulse Width	4.6000
Acquisition Time	0.6336
Modification Date	2022-06-28T16:38:59
Spectrometer Frequency	100.53
Spectral Width	25252.1
Lowest Frequency	-2573.4
Nucleus	Carbon13

5au $^{13}C\{^{1}H\}$ NMR spectrum (100 MHz in $CDCl_3$)

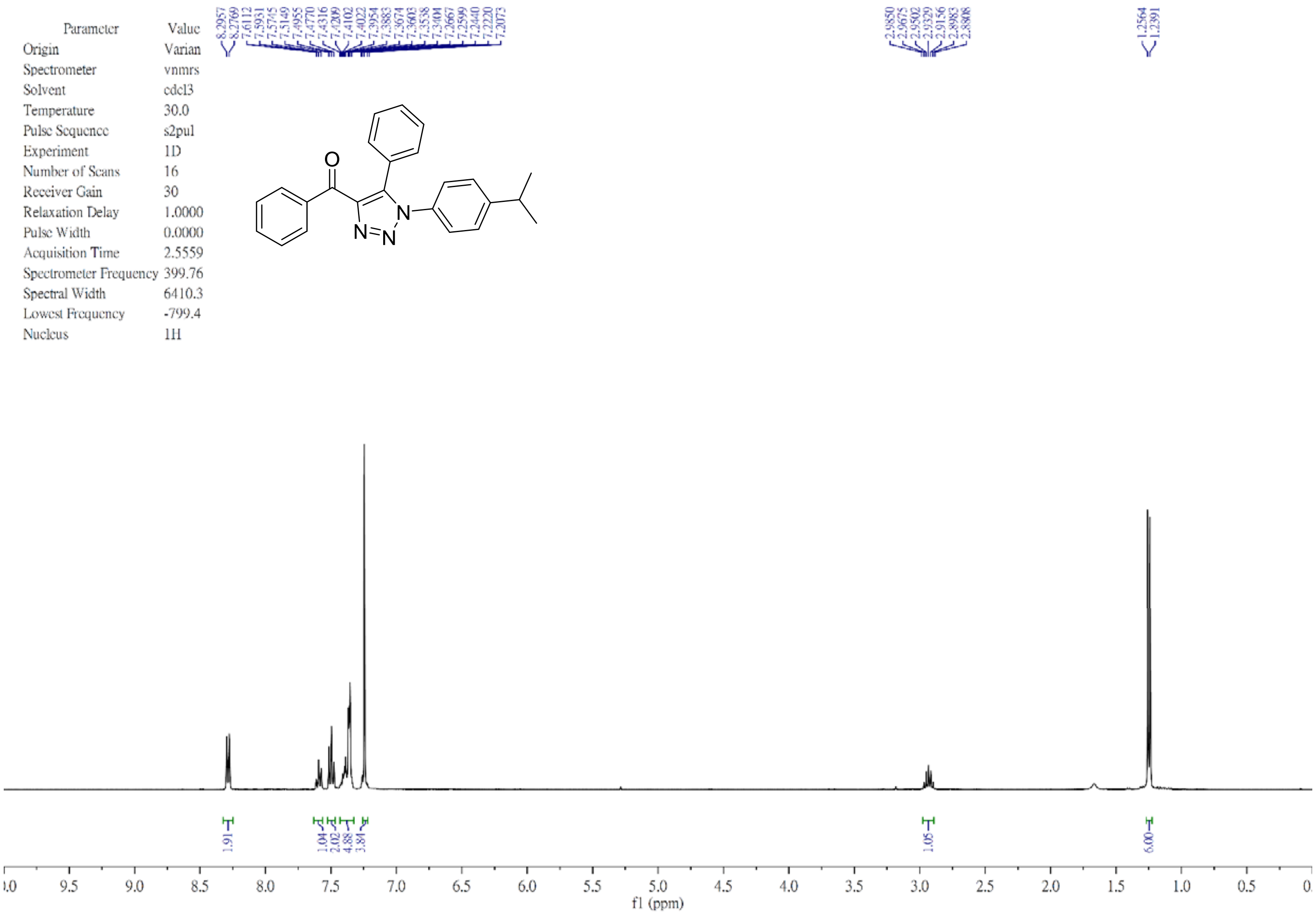

5av ^{1}H NMR spectrum (400 MHz in $CDCl_3$)

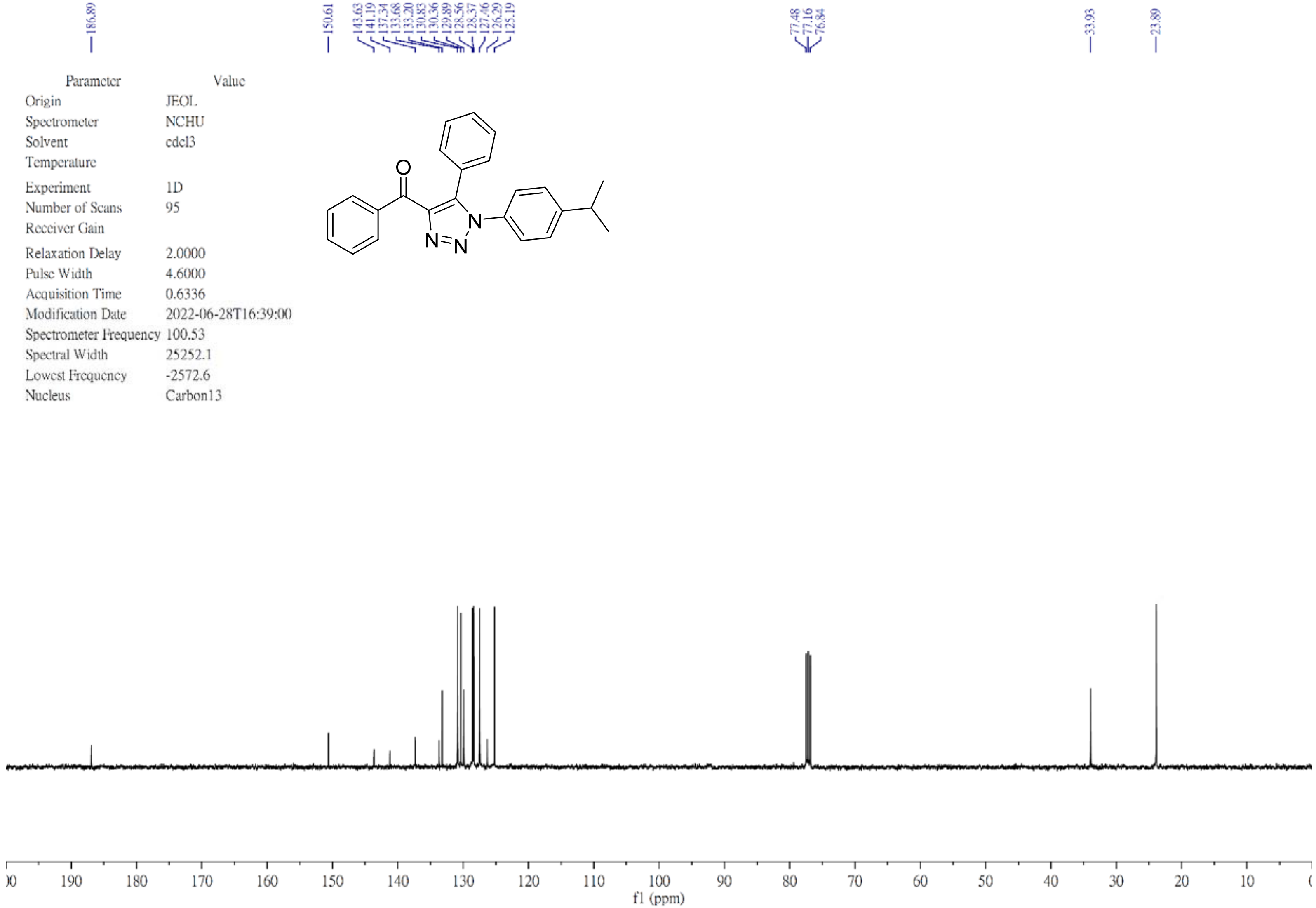

5av $^{13}C\{^1H\}$ NMR spectrum (100 MHz in $CDCl_3$)

Parameter	Value
Origin	Varian
Spectrometer	vnmrs
Solvent	cdcl3
Temperature	30.0
Pulse Sequence	s2pul
Experiment	1D
Number of Scans	20
Receiver Gain	54
Relaxation Delay	1.0000
Pulse Width	0.0000
Acquisition Time	2.5559
Spectrometer Frequency	399.76
Spectral Width	6410.3
Lowest Frequency	-800.6
Nucleus	1H

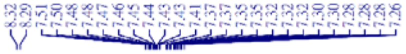

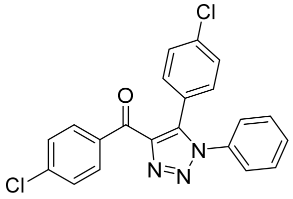

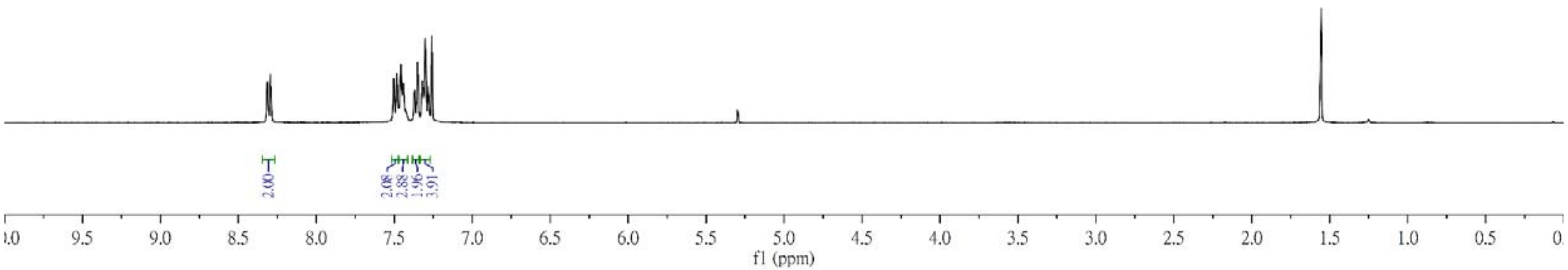

5ba ^{1}H NMR spectrum (400 MHz in $CDCl_3$)

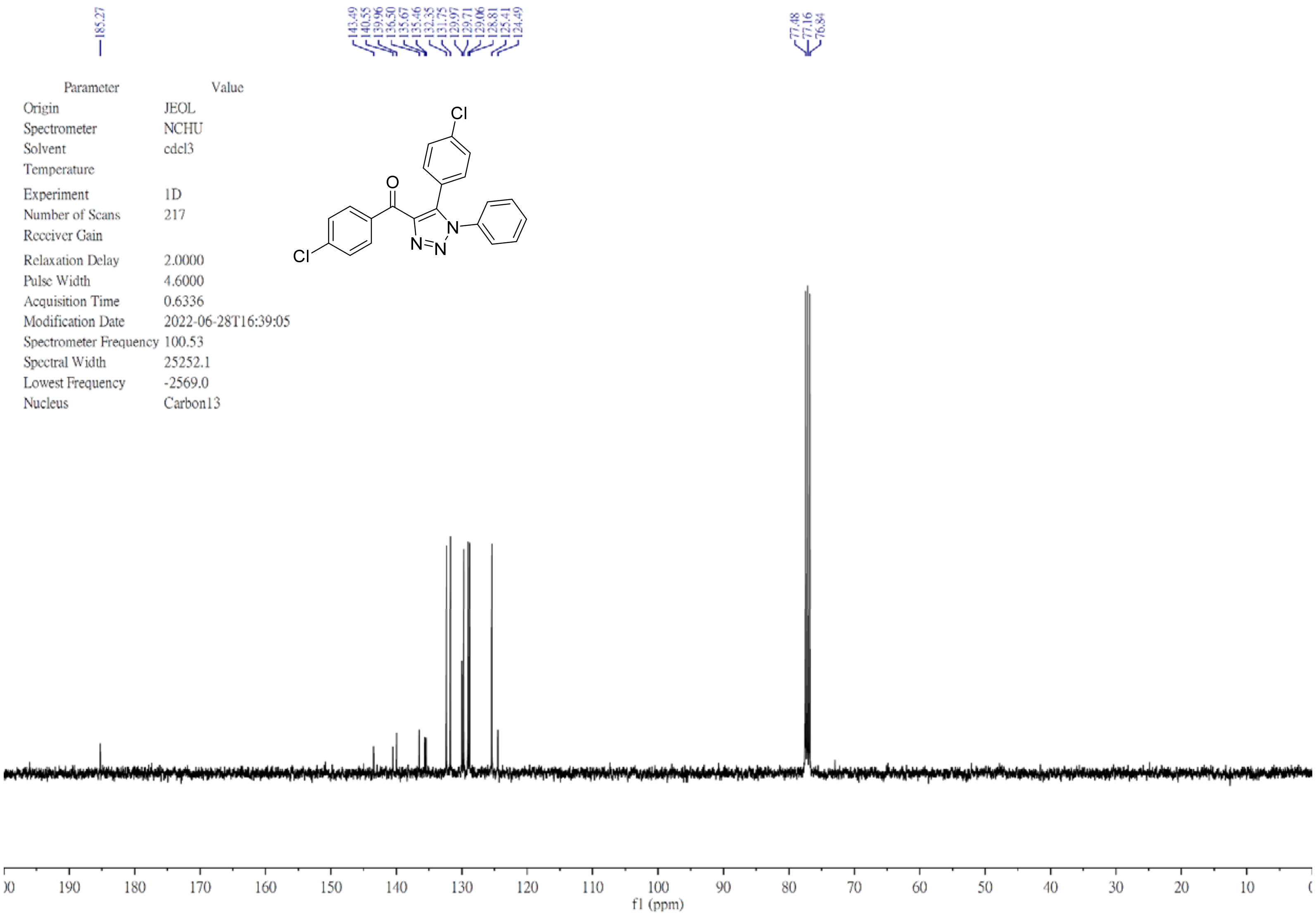

5ba $^{13}C\{^{1}H\}$ NMR spectrum (100 MHz in $CDCl_3$)

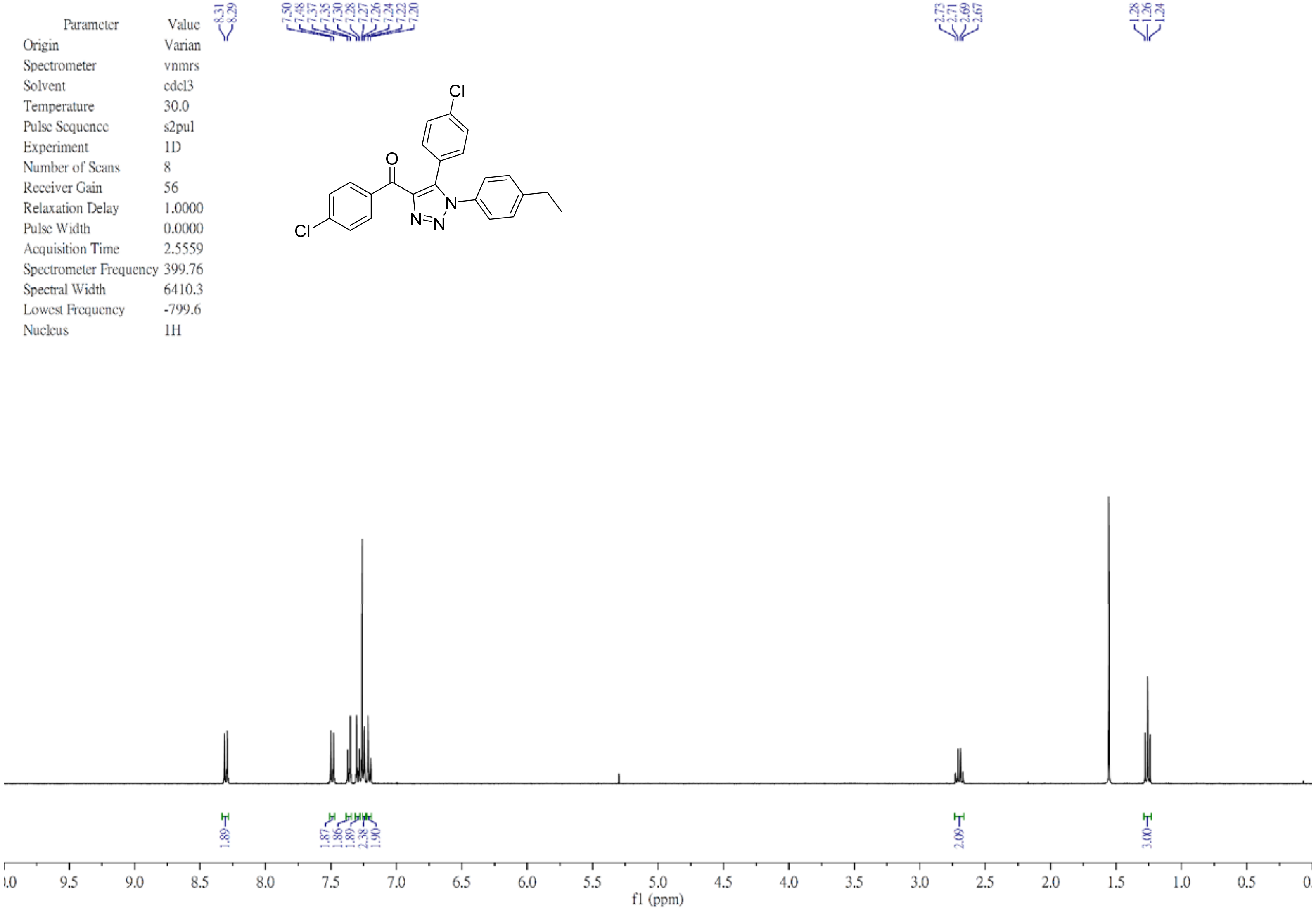

5be ^{1}H NMR spectrum (400 MHz in $CDCl_3$)

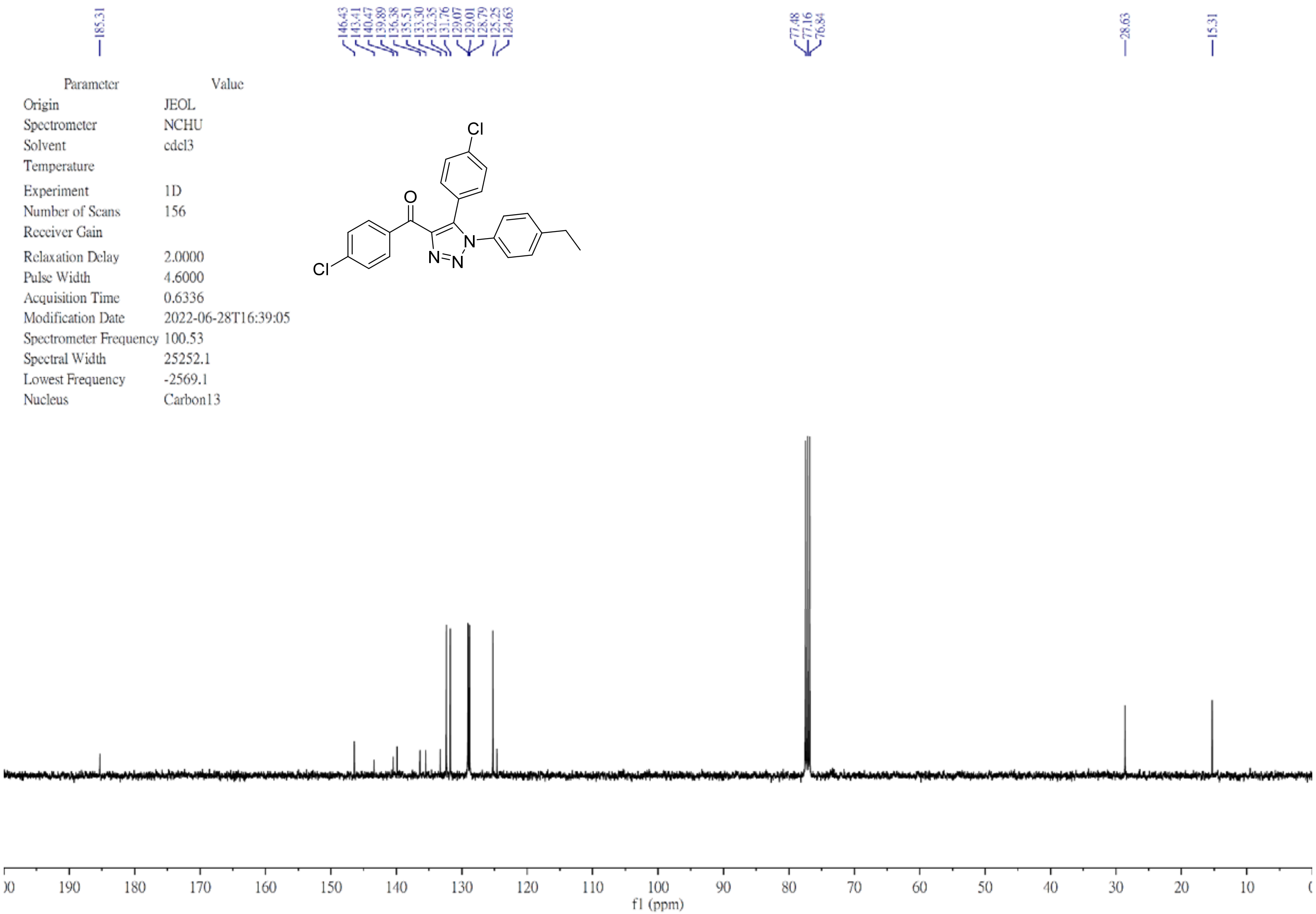

5be $^{13}C\{^{1}H\}$ NMR spectrum (100 MHz in $CDCl_3$)

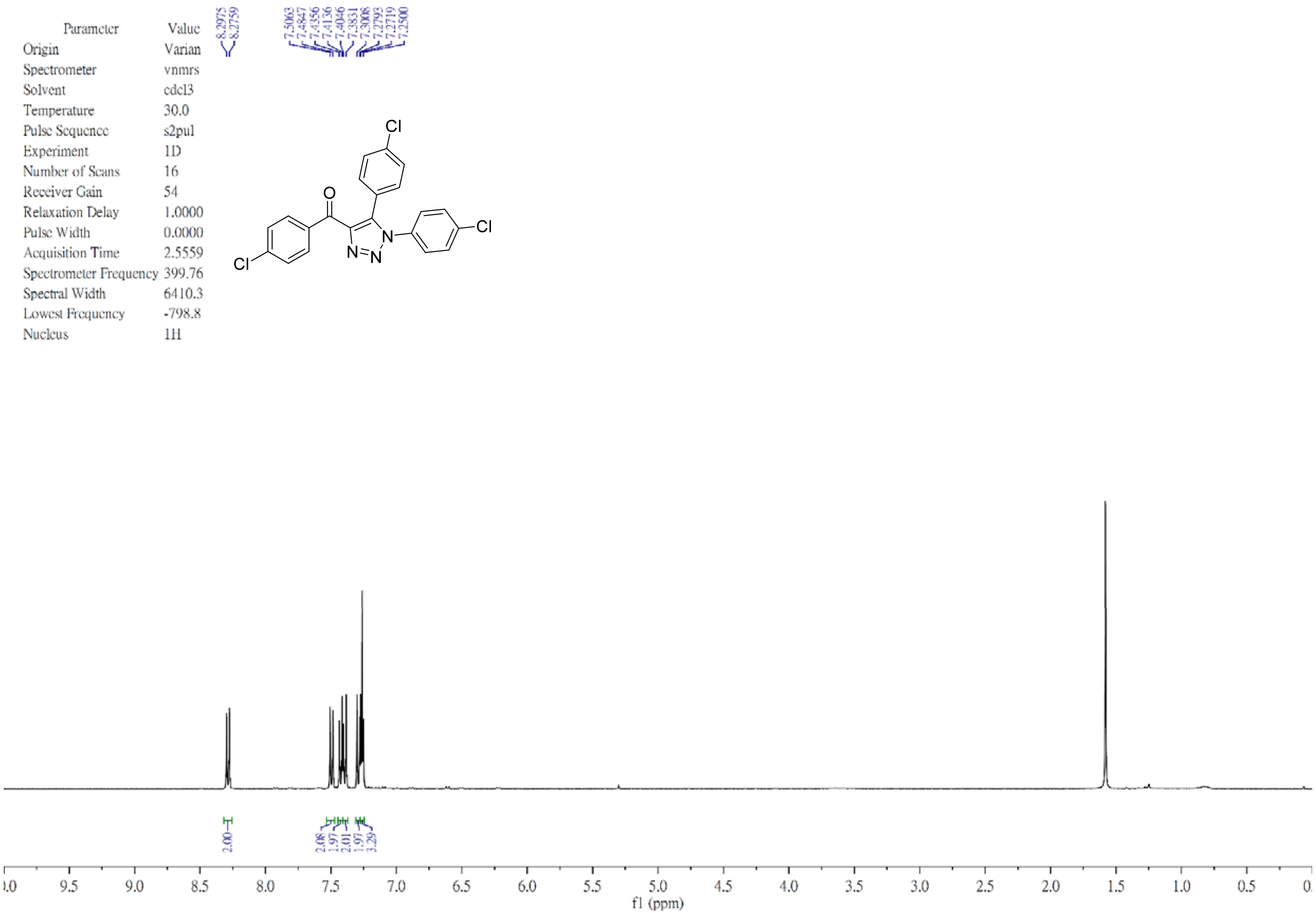

5bk ^{1}H NMR spectrum (400 MHz in $CDCl_3$)

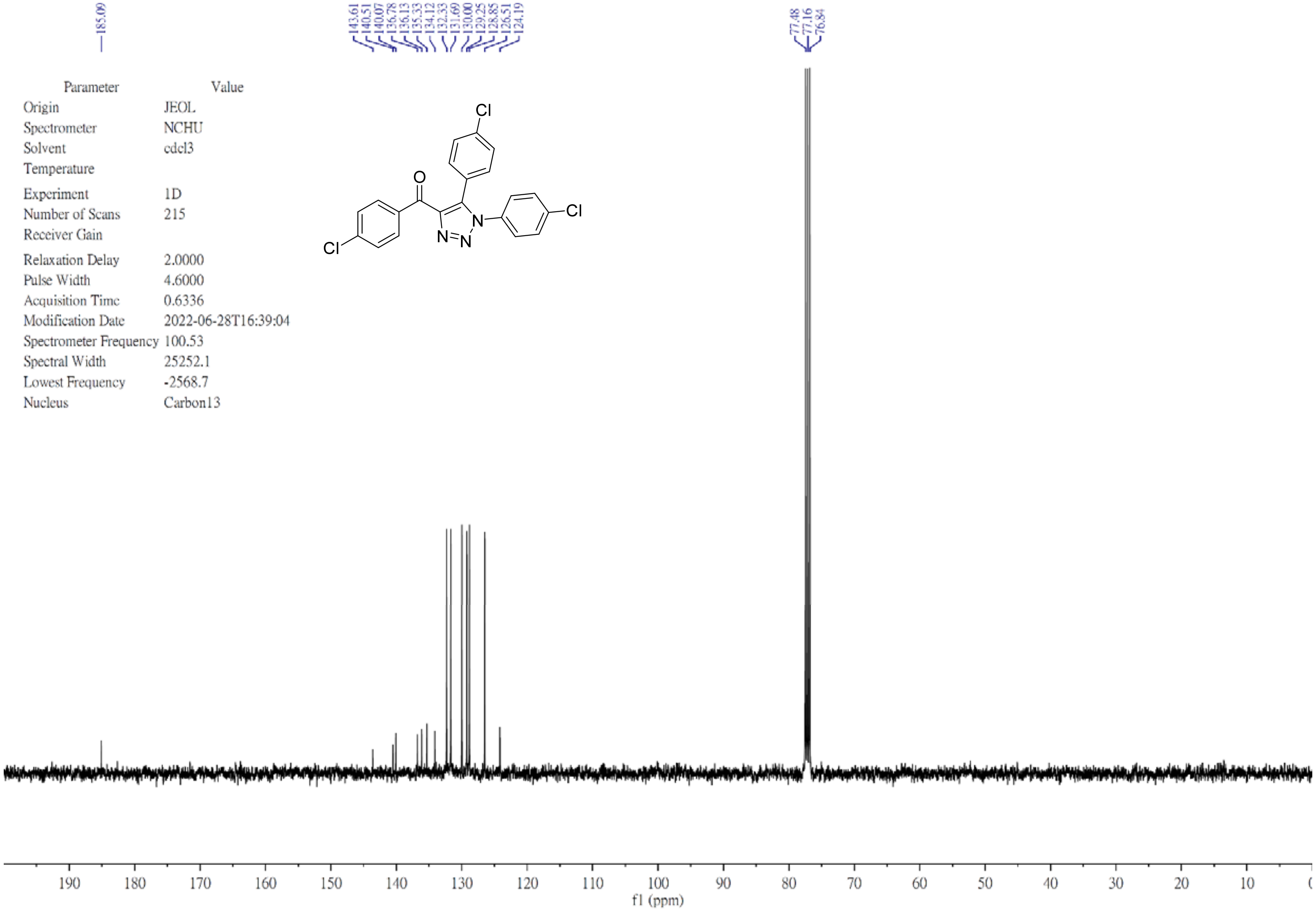

5bk $^{13}C\{^1H\}$ NMR spectrum (100 MHz in $CDCl_3$)

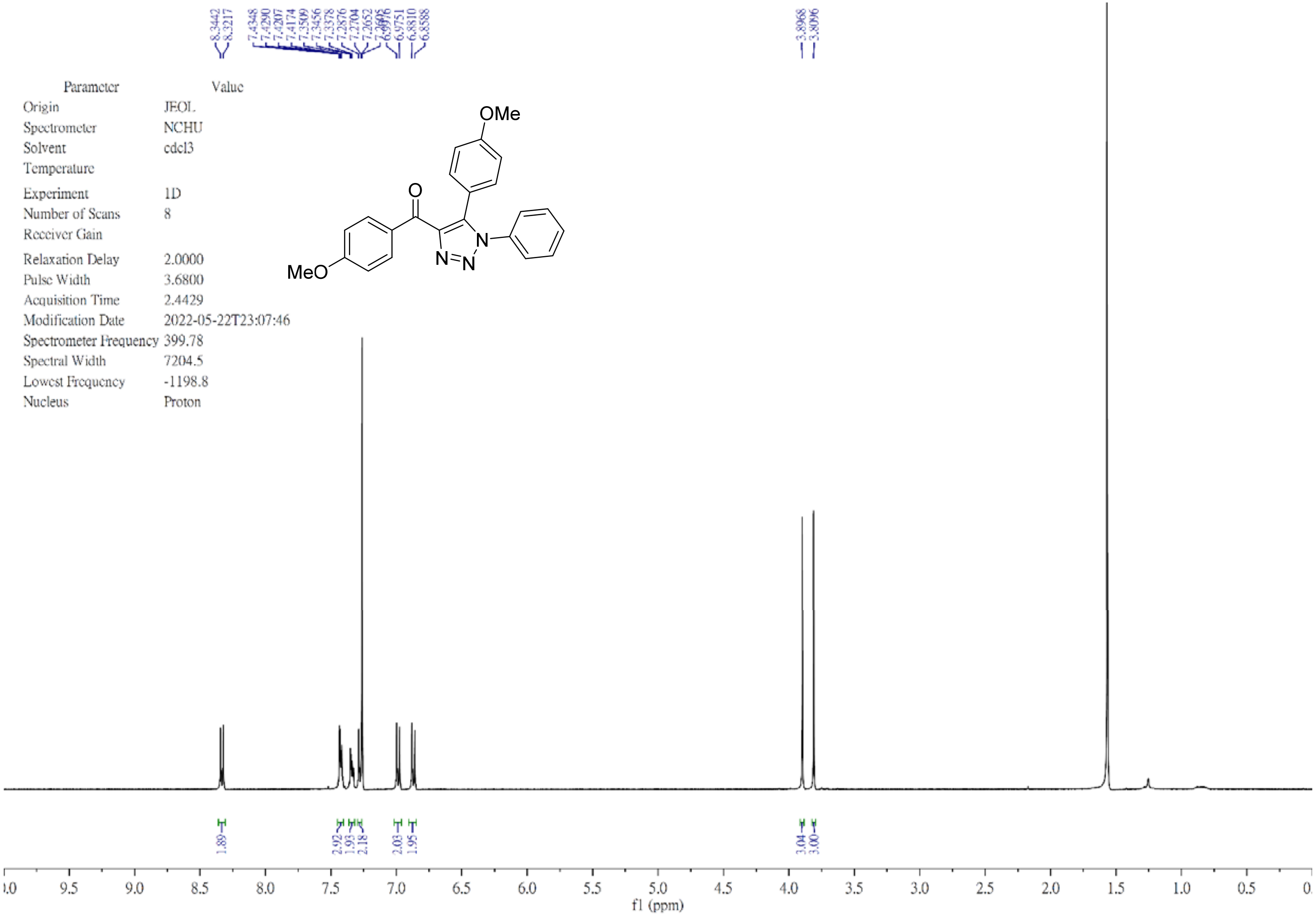

5ca ^{1}H NMR spectrum (400 MHz in $CDCl_3$)

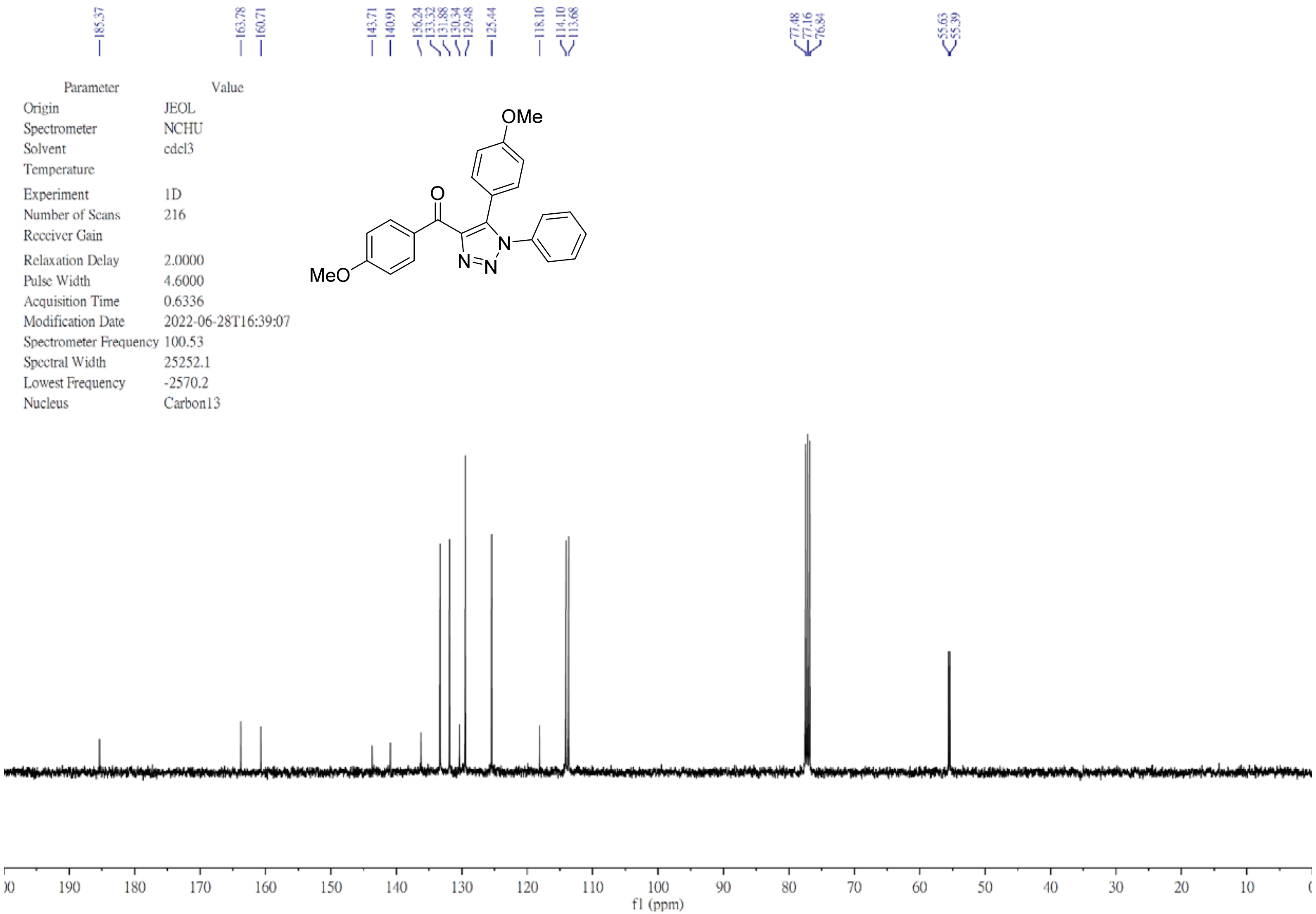

5ca $^{13}C\{^1H\}$ NMR spectrum (100 MHz in $CDCl_3$)

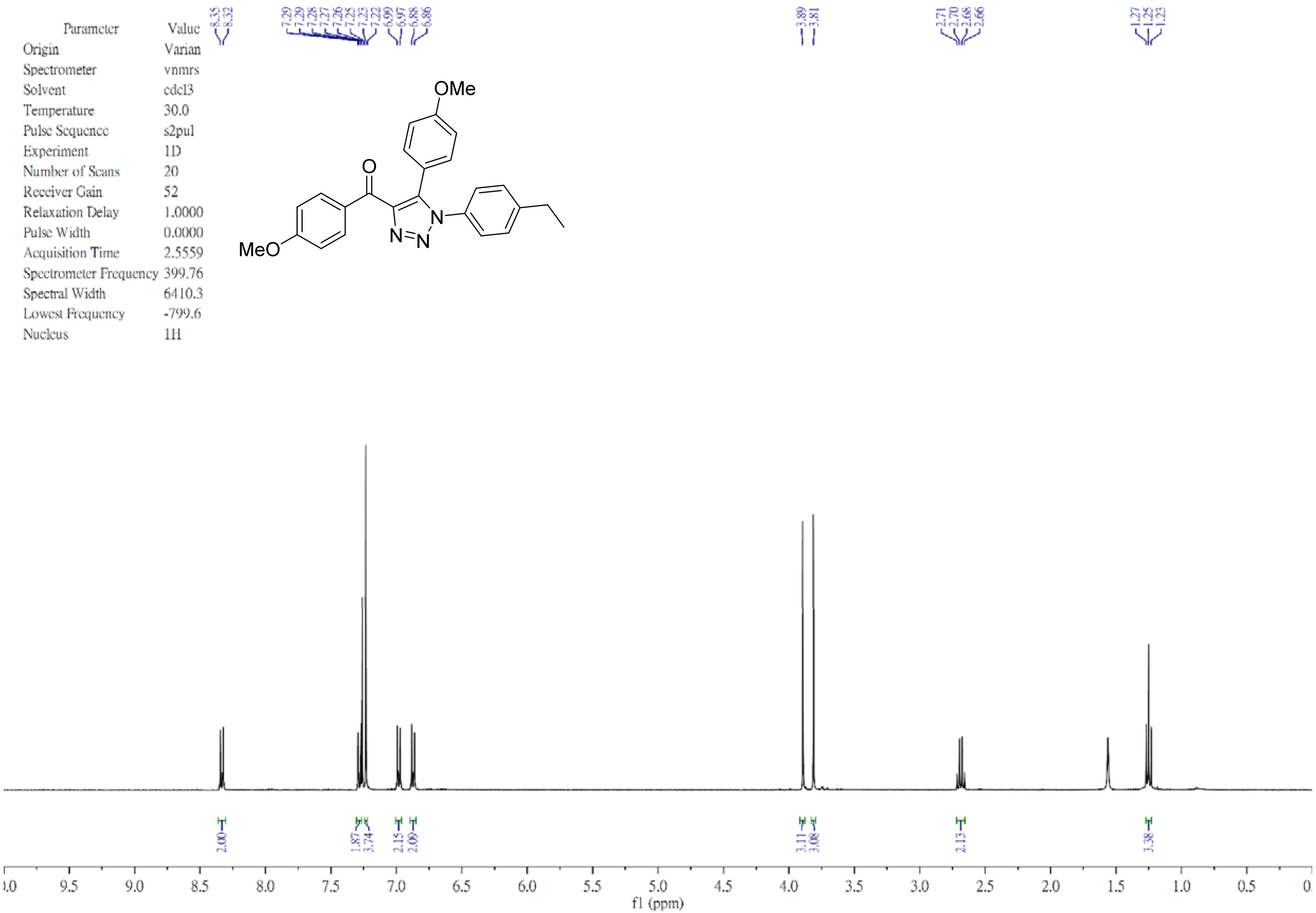

5ce ^{1}H NMR spectrum (400 MHz in $CDCl_3$)

Parameter	Value
Origin	JEOL
Spectrometer	NCHU
Solvent	cdcl3
Temperature	
Experiment	1D
Number of Scans	158
Receiver Gain	
Relaxation Delay	2.0000
Pulse Width	4.6000
Acquisition Time	0.6336
Modification Date	2022-06-28T16:39:08
Spectrometer Frequency	100.53
Spectral Width	25252.1
Lowest Frequency	-2570.3
Nucleus	Carbon13

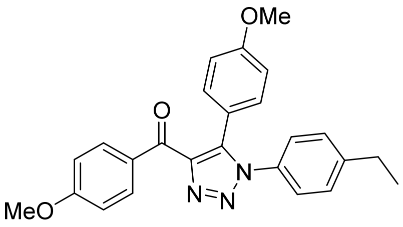

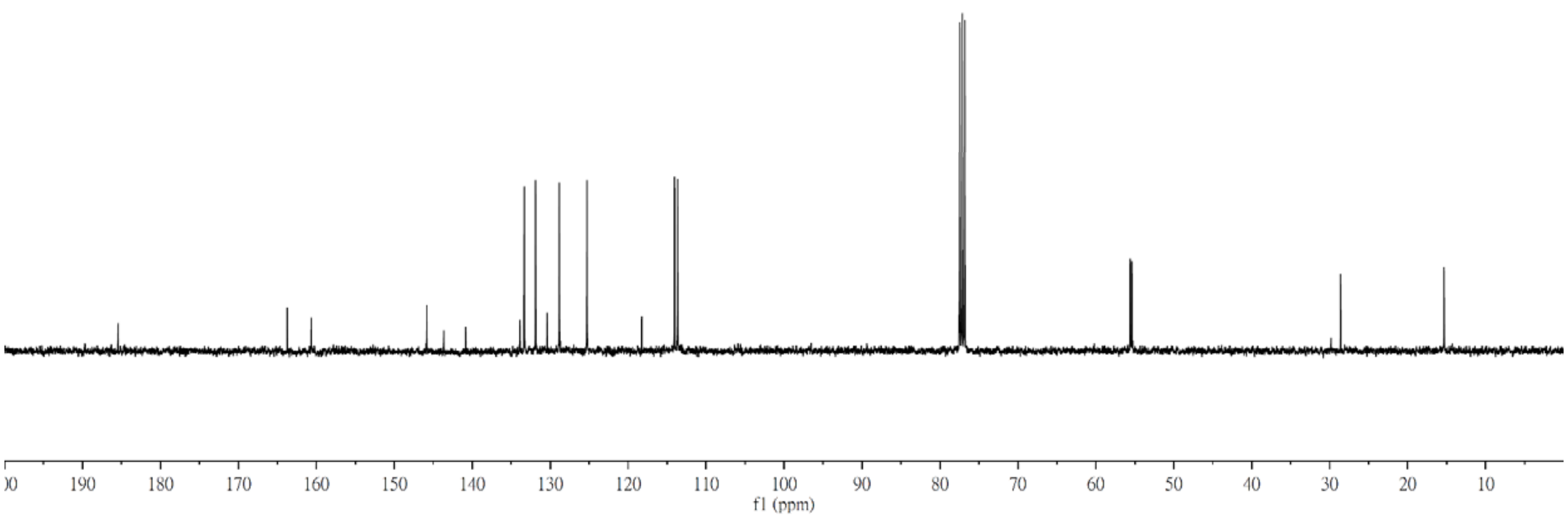

5ce $^{13}C\{^{1}H\}$ NMR spectrum (100 MHz in $CDCl_3$)

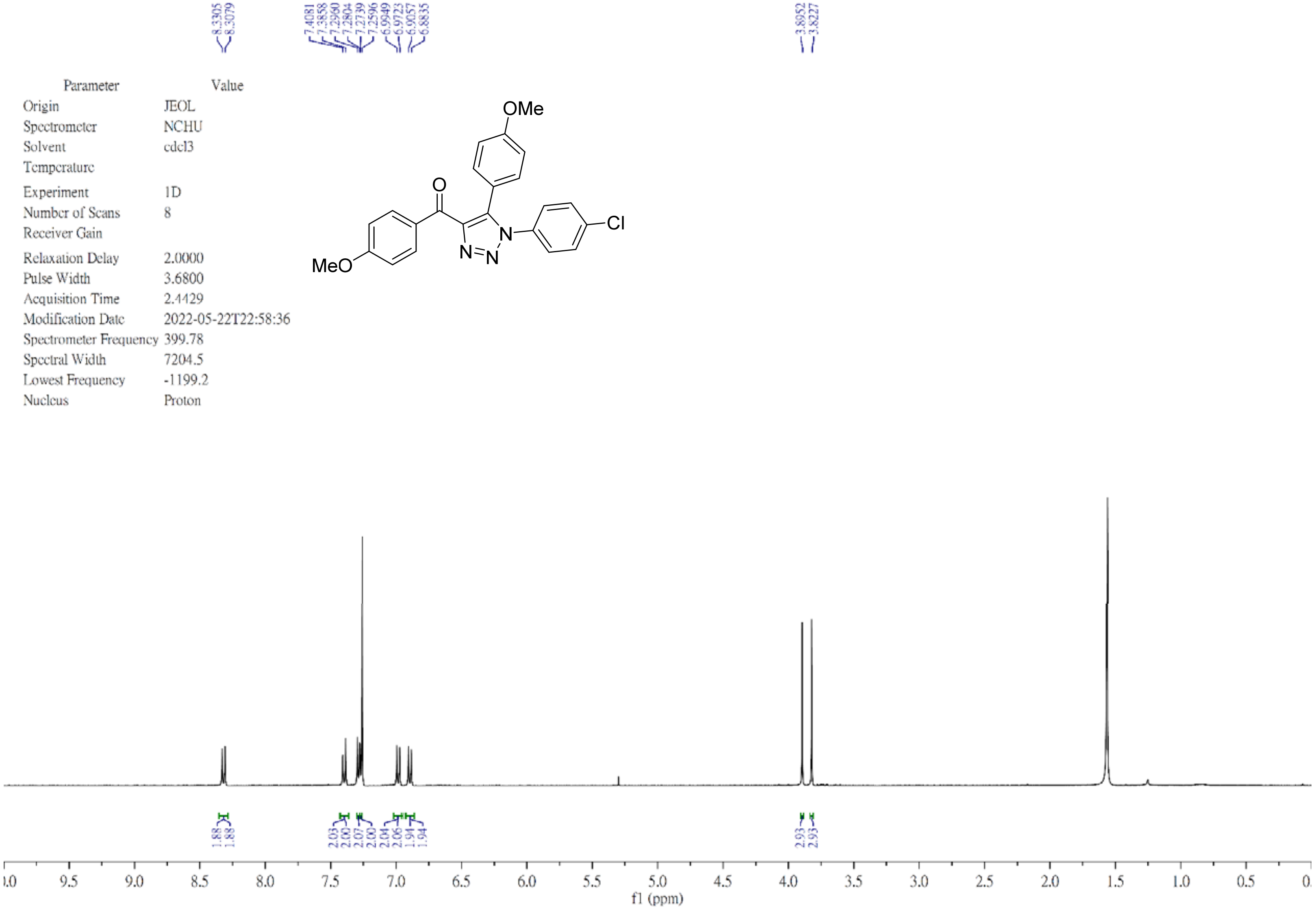

5ck ^{1}H NMR spectrum (400 MHz in $CDCl_3$)

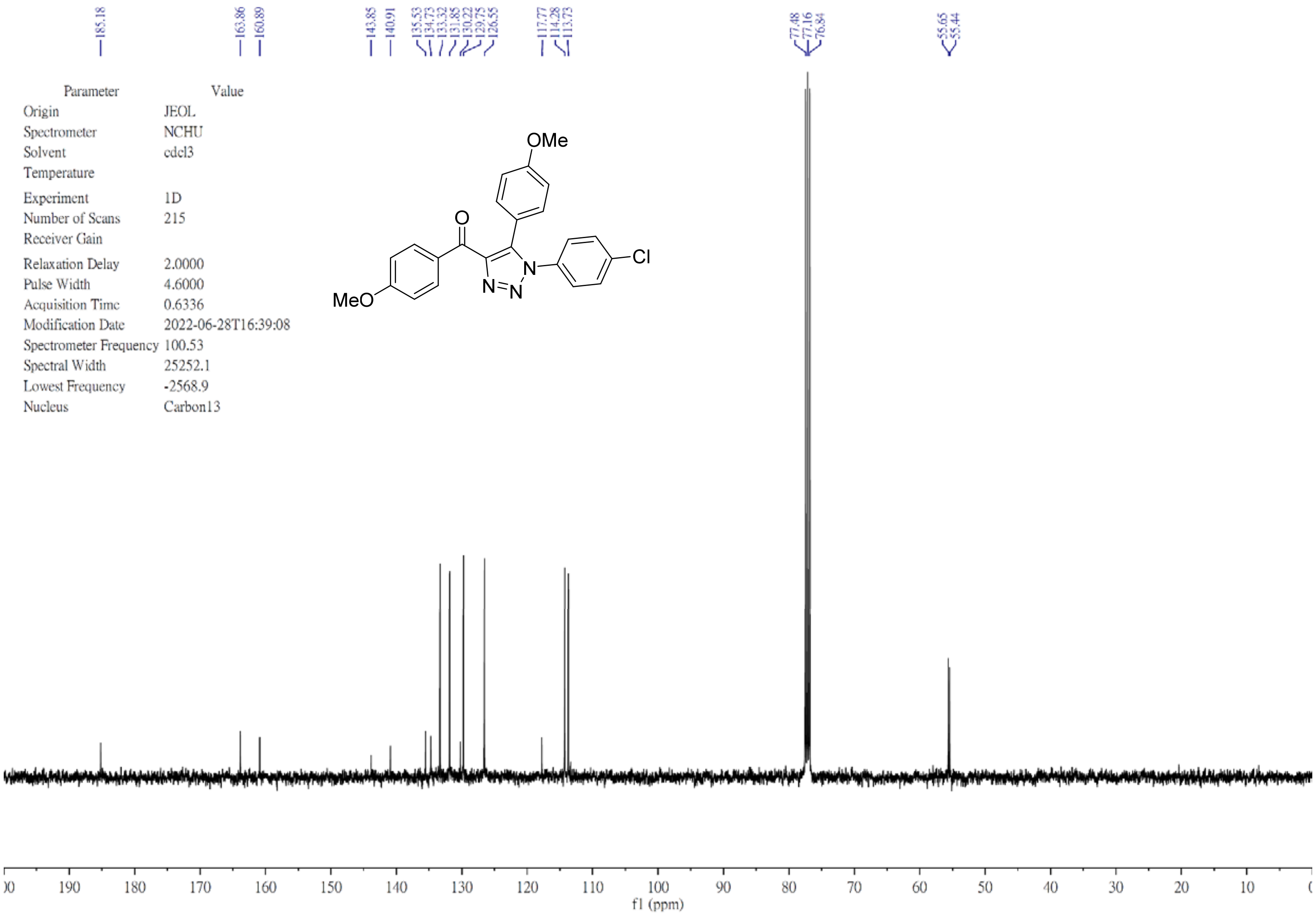

5ck $^{13}C\{^{1}H\}$ NMR spectrum (100 MHz in $CDCl_3$)

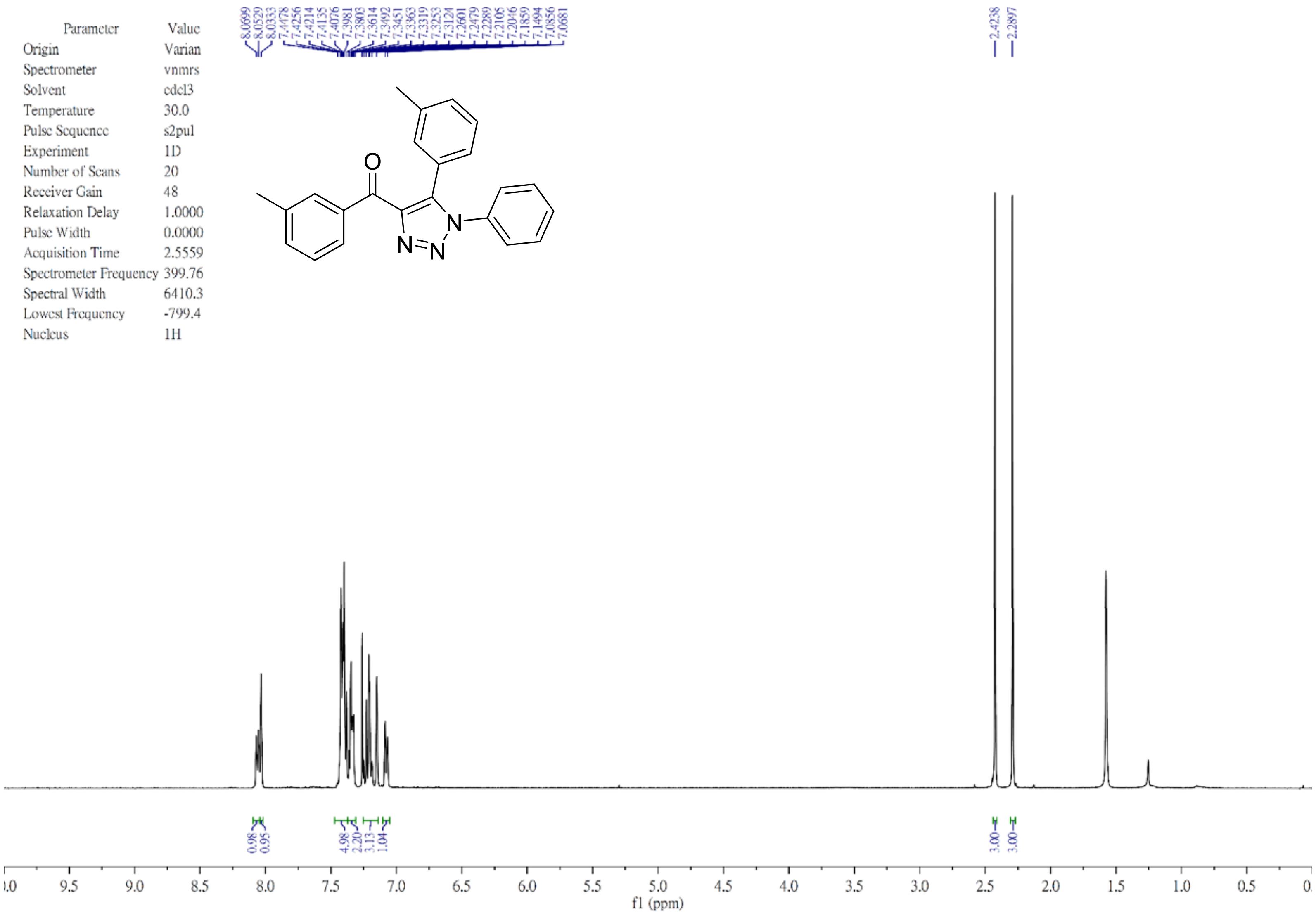

5da ^{1}H NMR spectrum (400 MHz in $CDCl_3$)

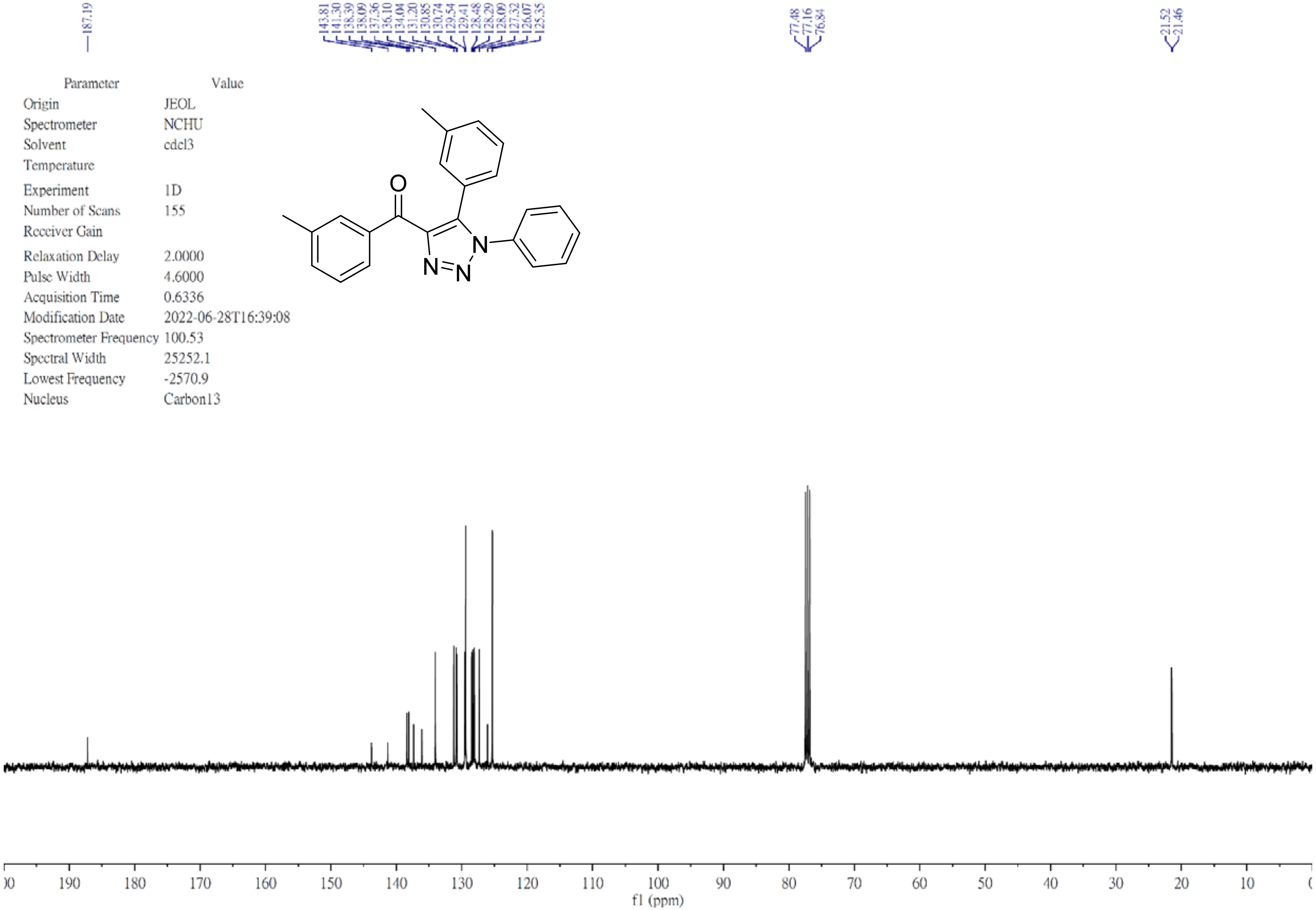

5da $^{13}C\{^{1}H\}$ NMR spectrum (100 MHz in $CDCl_3$)

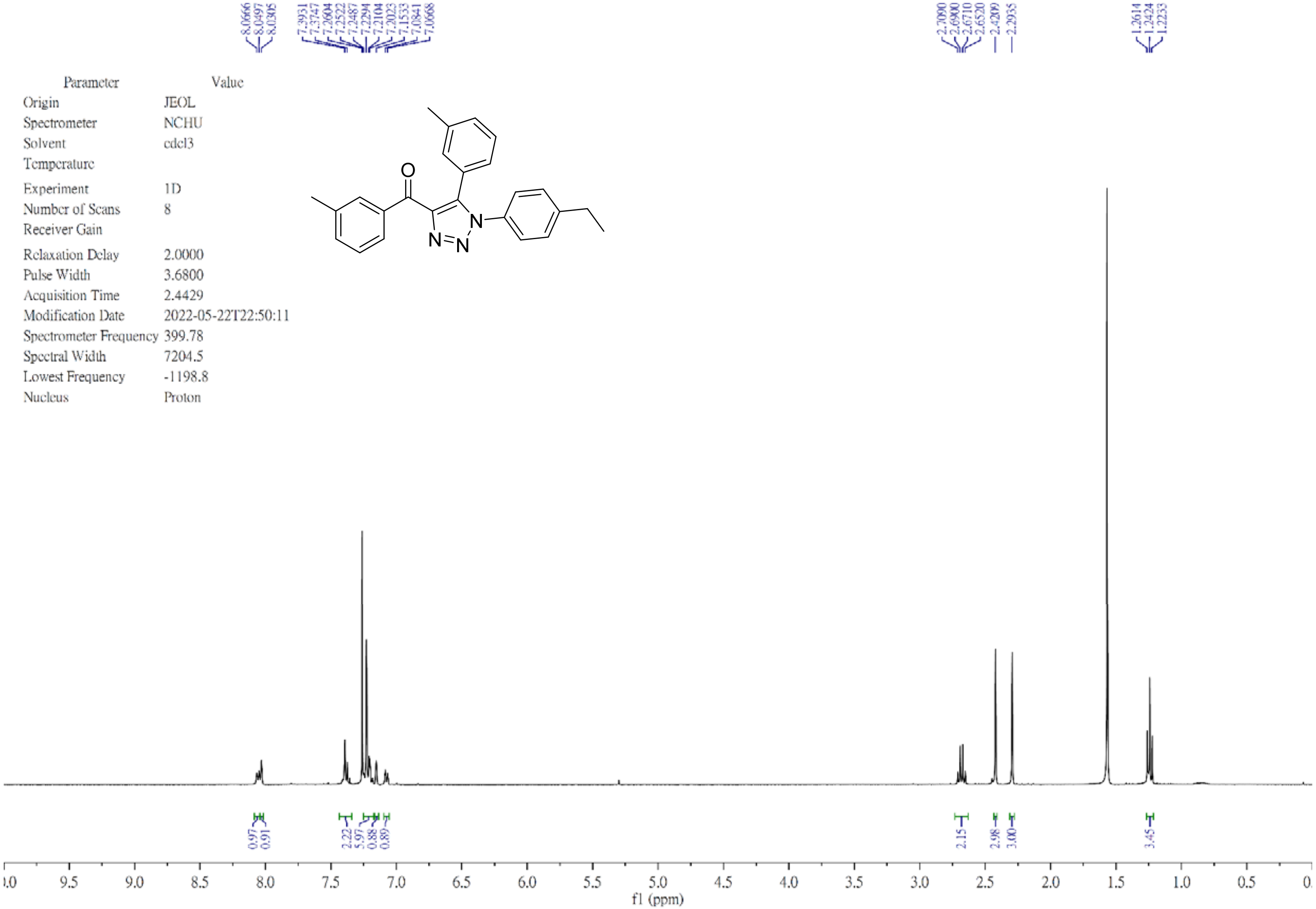

5de ^{1}H NMR spectrum (400 MHz in $CDCl_3$)

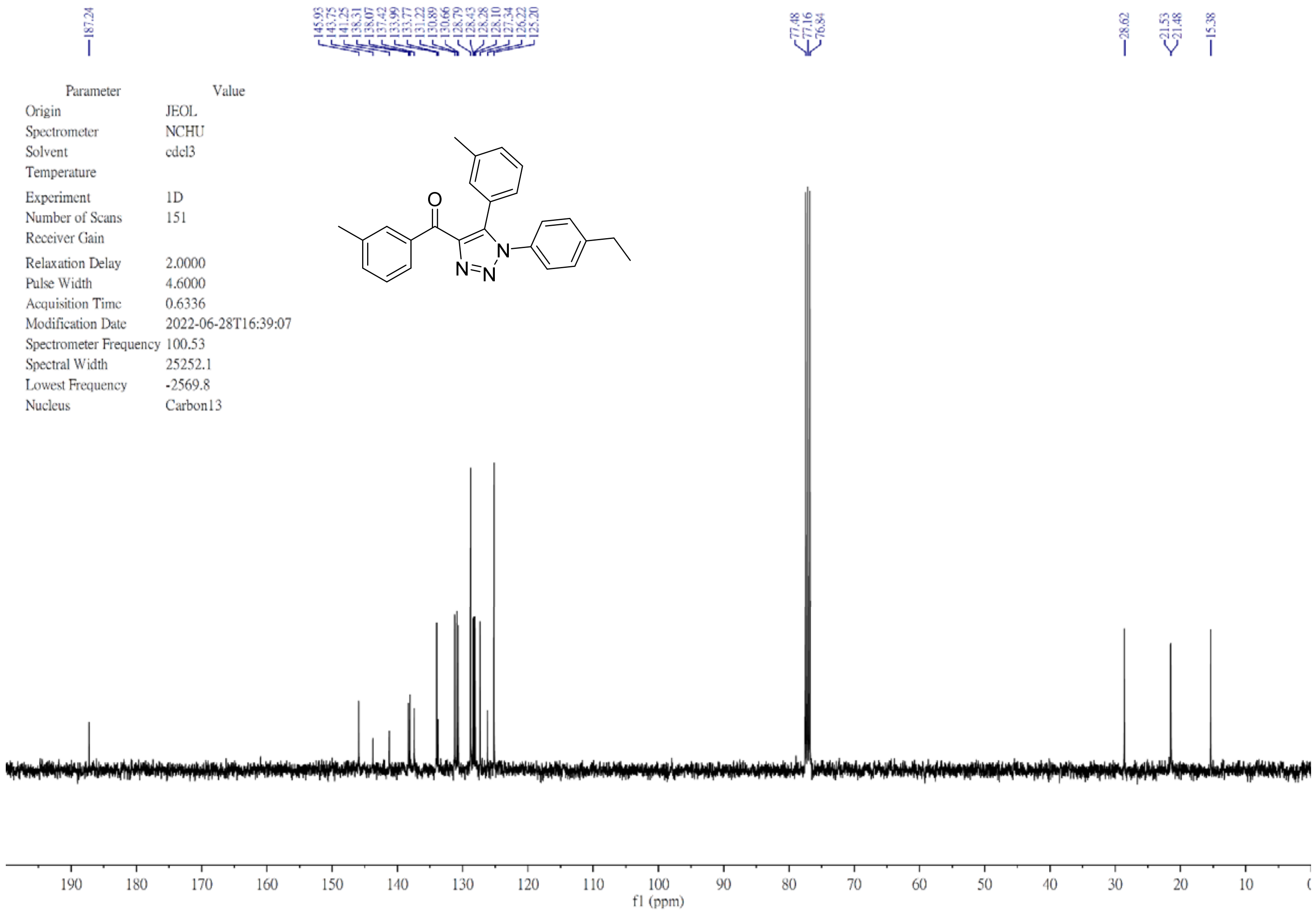

5de $^{13}C\{^{1}H\}$ NMR spectrum (100 MHz in $CDCl_3$)

Parameter	Value
Origin	Varian
Spectrometer	vnmrs
Solvent	cdcl3
Temperature	30.0
Pulse Sequence	s2pul
Experiment	1D
Number of Scans	12
Receiver Gain	52
Relaxation Delay	1.0000
Pulse Width	0.0000
Acquisition Time	2.5559
Spectrometer Frequency	399.76
Spectral Width	6410.3
Lowest Frequency	-799.8
Nucleus	1H

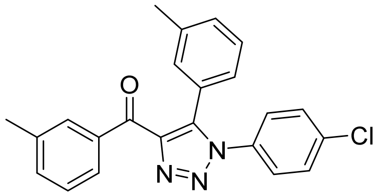

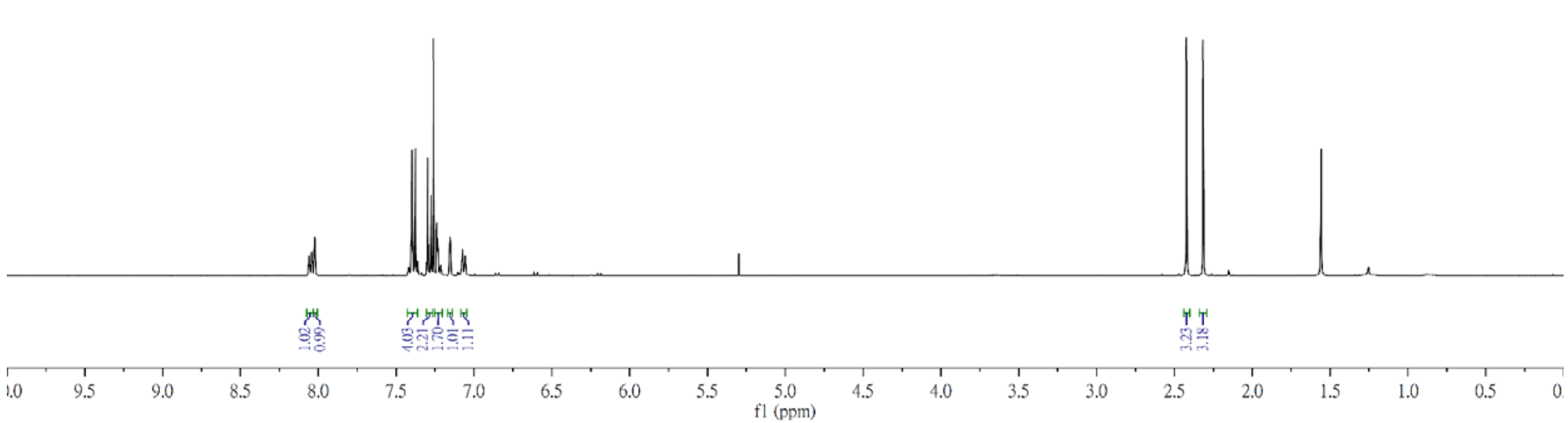

5dk ^{1}H NMR spectrum (400 MHz in $CDCl_3$)

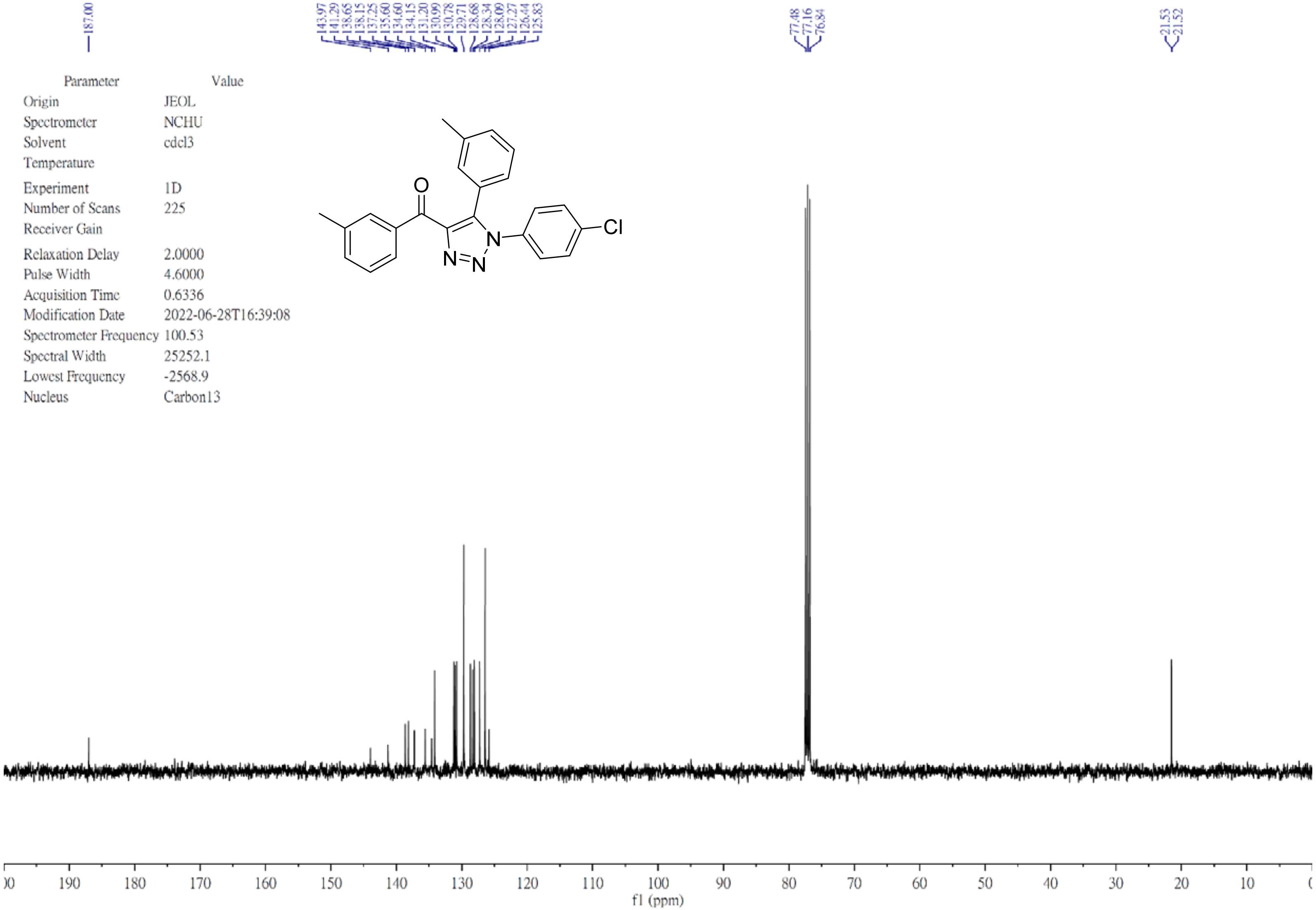

5dk $^{13}C\{^{1}H\}$ NMR spectrum (100 MHz in $CDCl_3$)

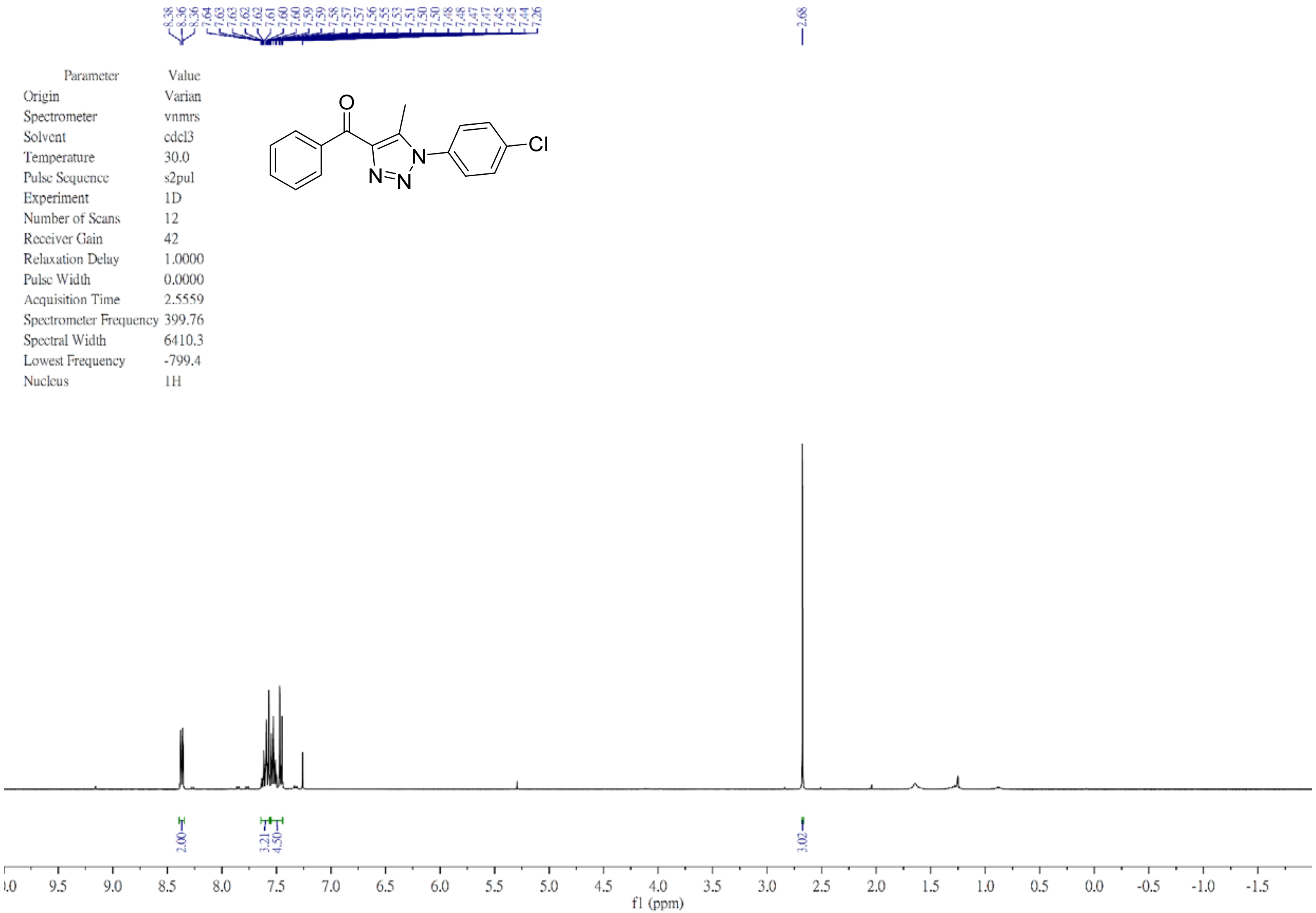

5ek ^{1}H NMR spectrum (400 MHz in $CDCl_3$)

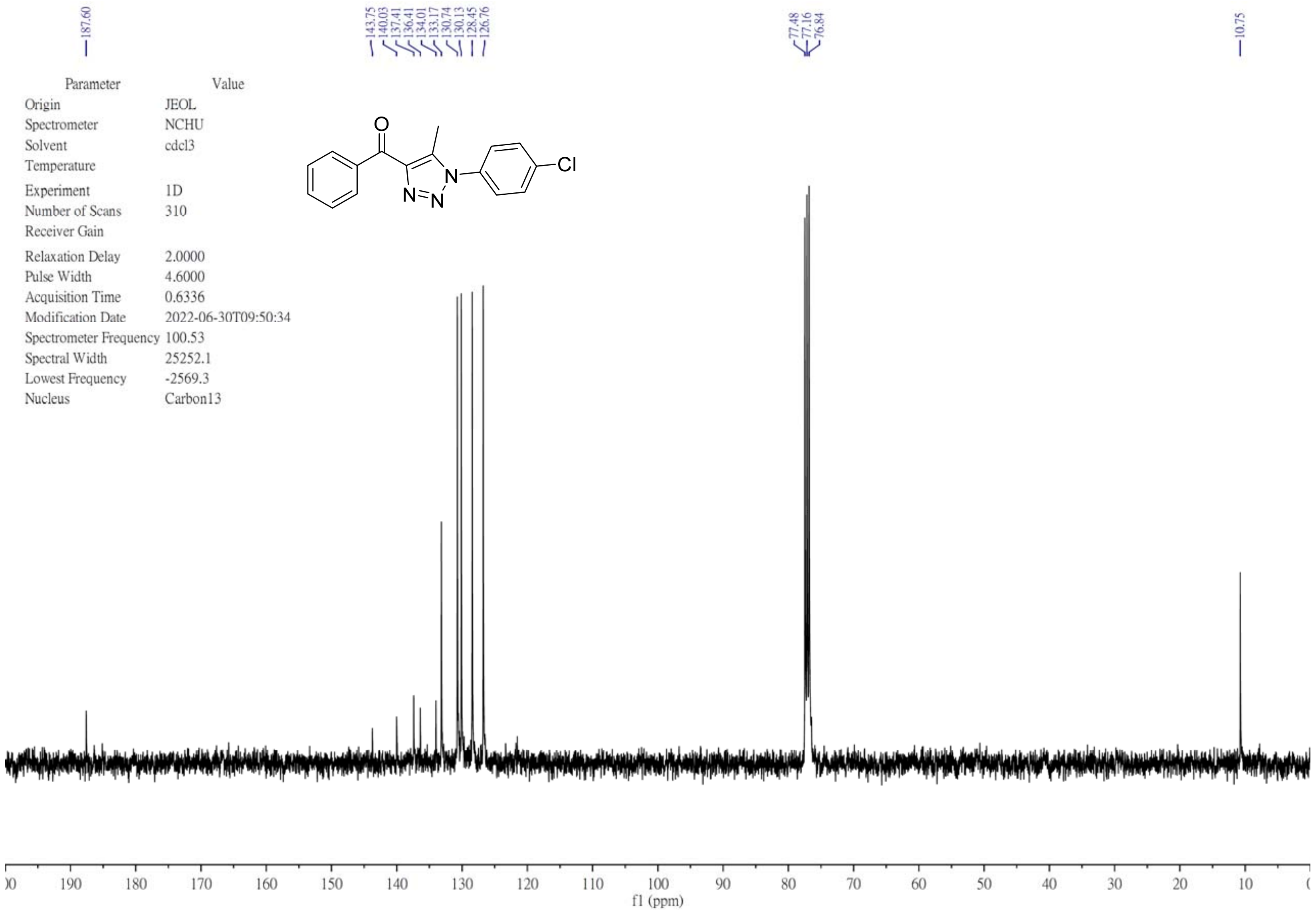

5ek $^{13}C\{^{1}H\}$ NMR spectrum (100 MHz in $CDCl_3$)

Parameter	Value
Origin	Varian
Spectrometer	vnmrs
Solvent	cdcl3
Temperature	30.0
Pulse Sequence	s2pul
Experiment	1D
Number of Scans	16
Receiver Gain	52
Relaxation Delay	1.0000
Pulse Width	0.0000
Acquisition Time	2.5559
Spectrometer Frequency	399.76
Spectral Width	6410.3
Lowest Frequency	-799.2
Nucleus	1H

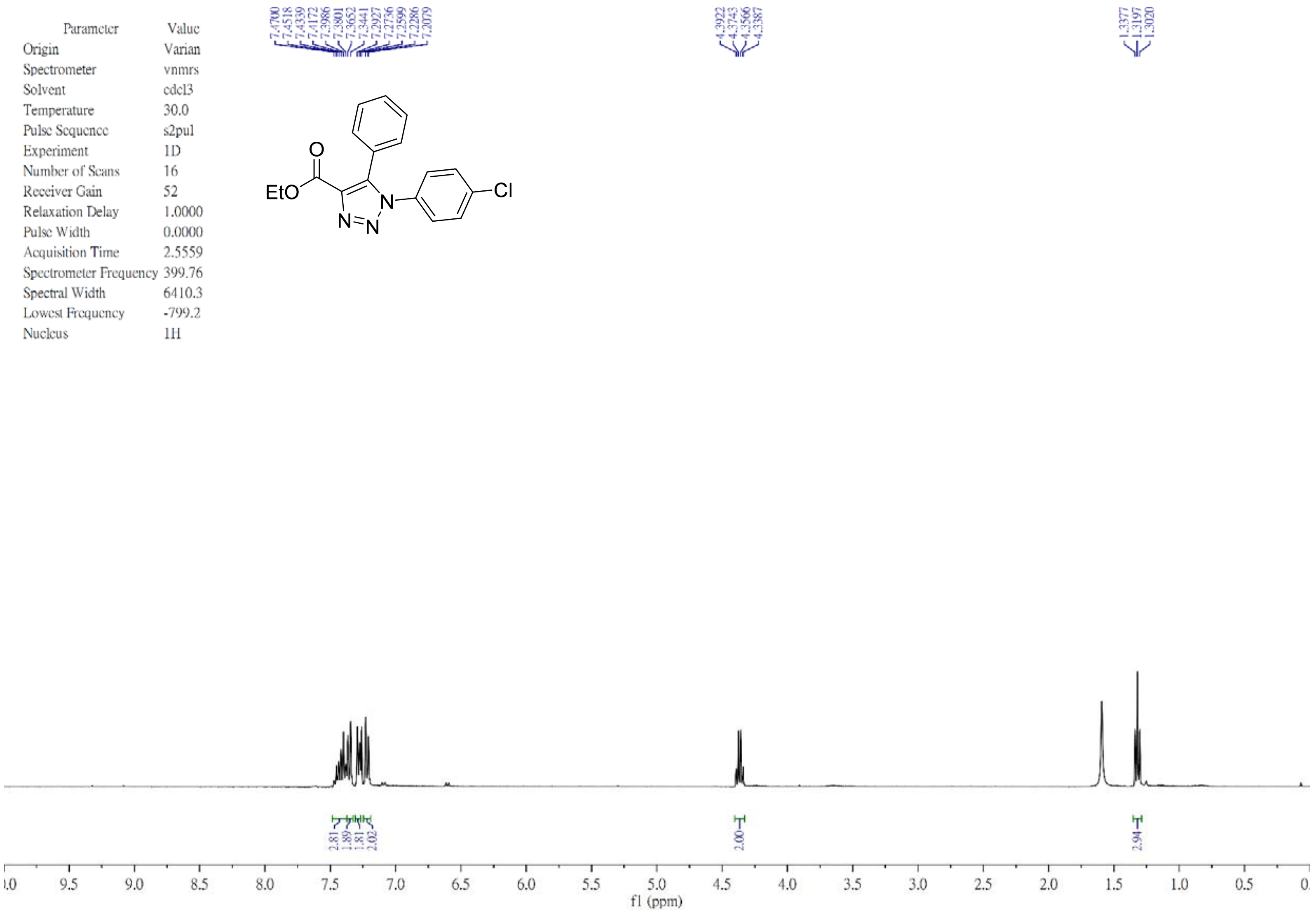

5fk ^{1}H NMR spectrum (400 MHz in $CDCl_3$)

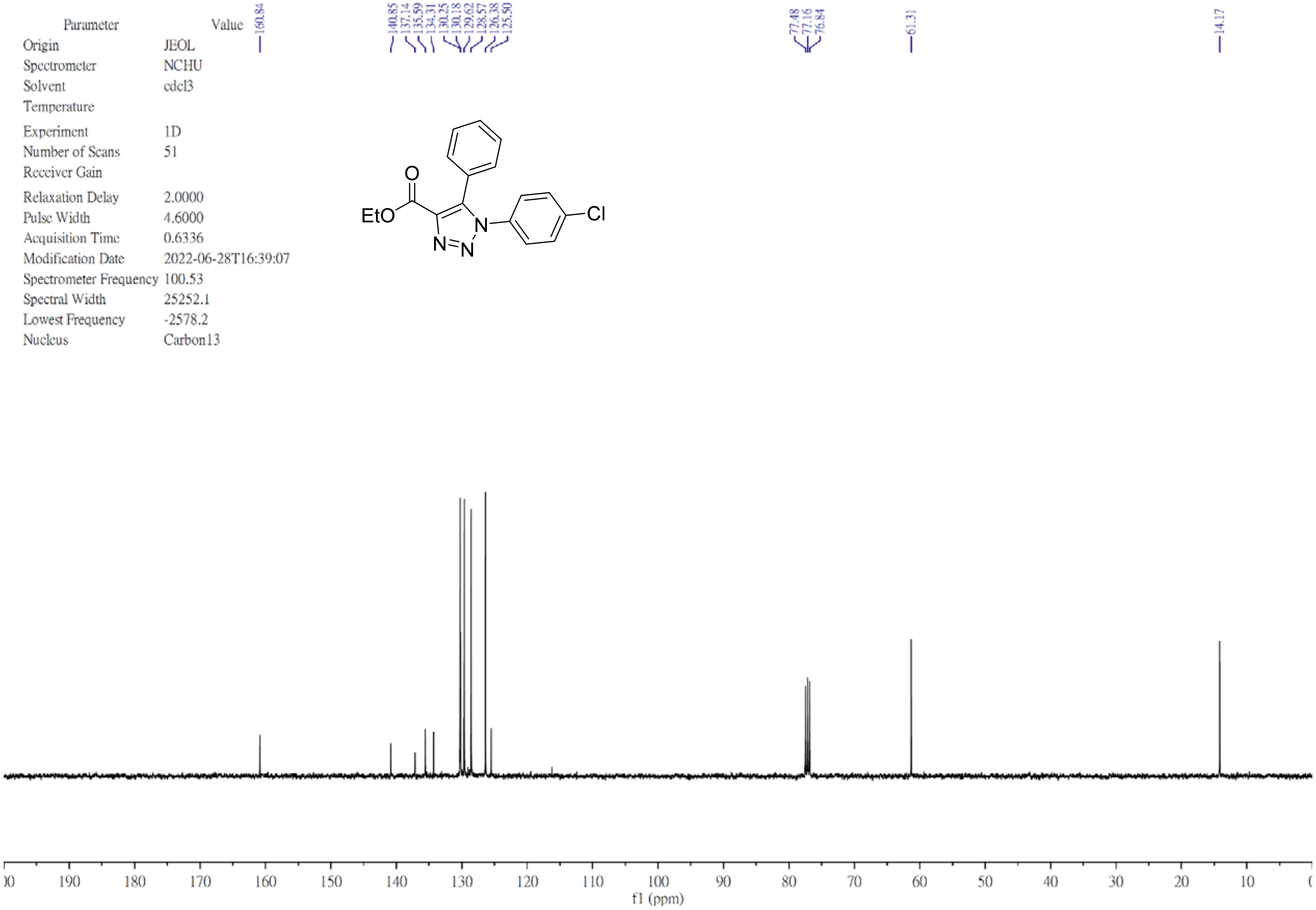

5fk $^{13}C\{^{1}H\}$ NMR spectrum (100 MHz in $CDCl_3$)

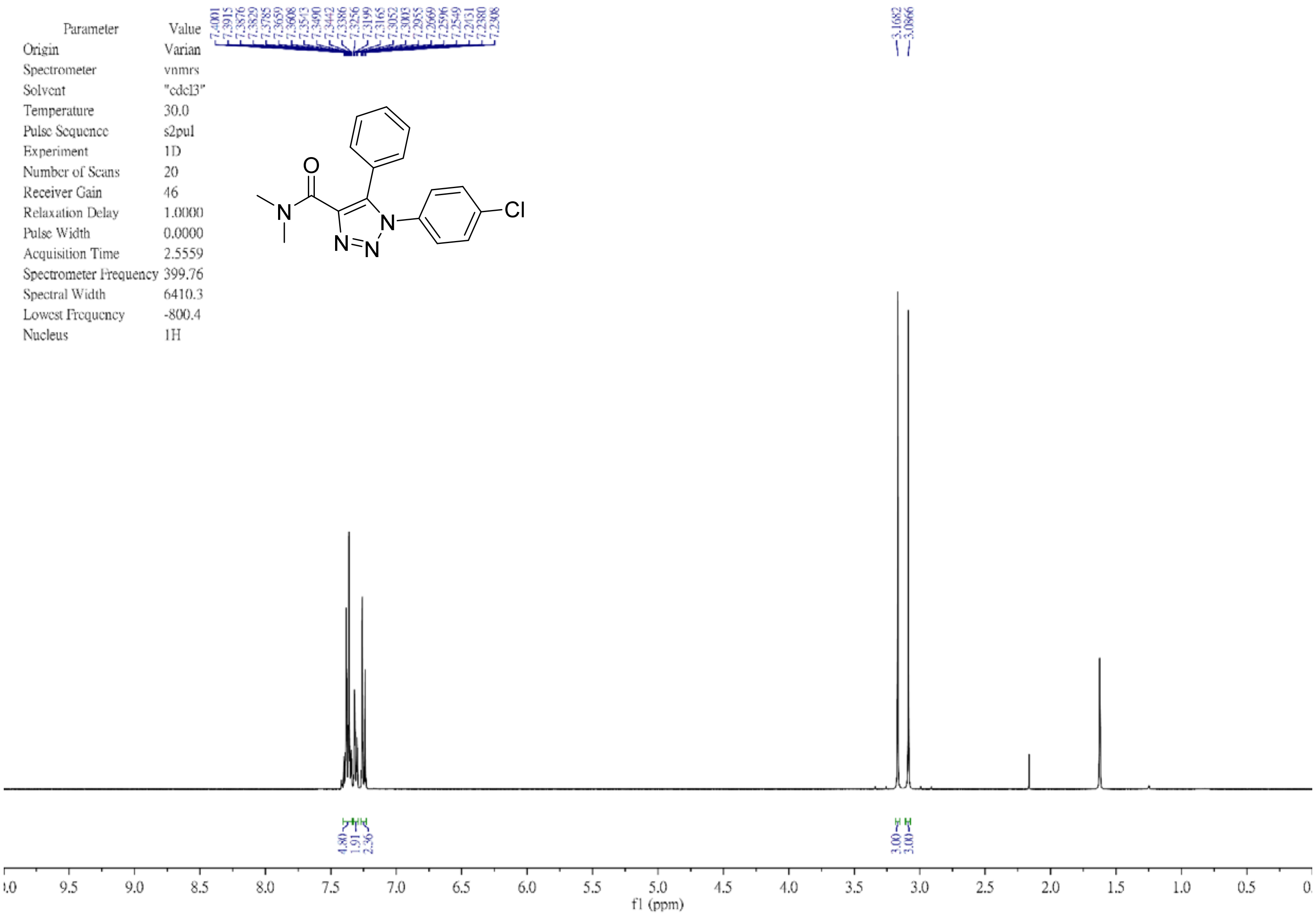

5gk ^{1}H NMR spectrum (400 MHz in $CDCl_3$)

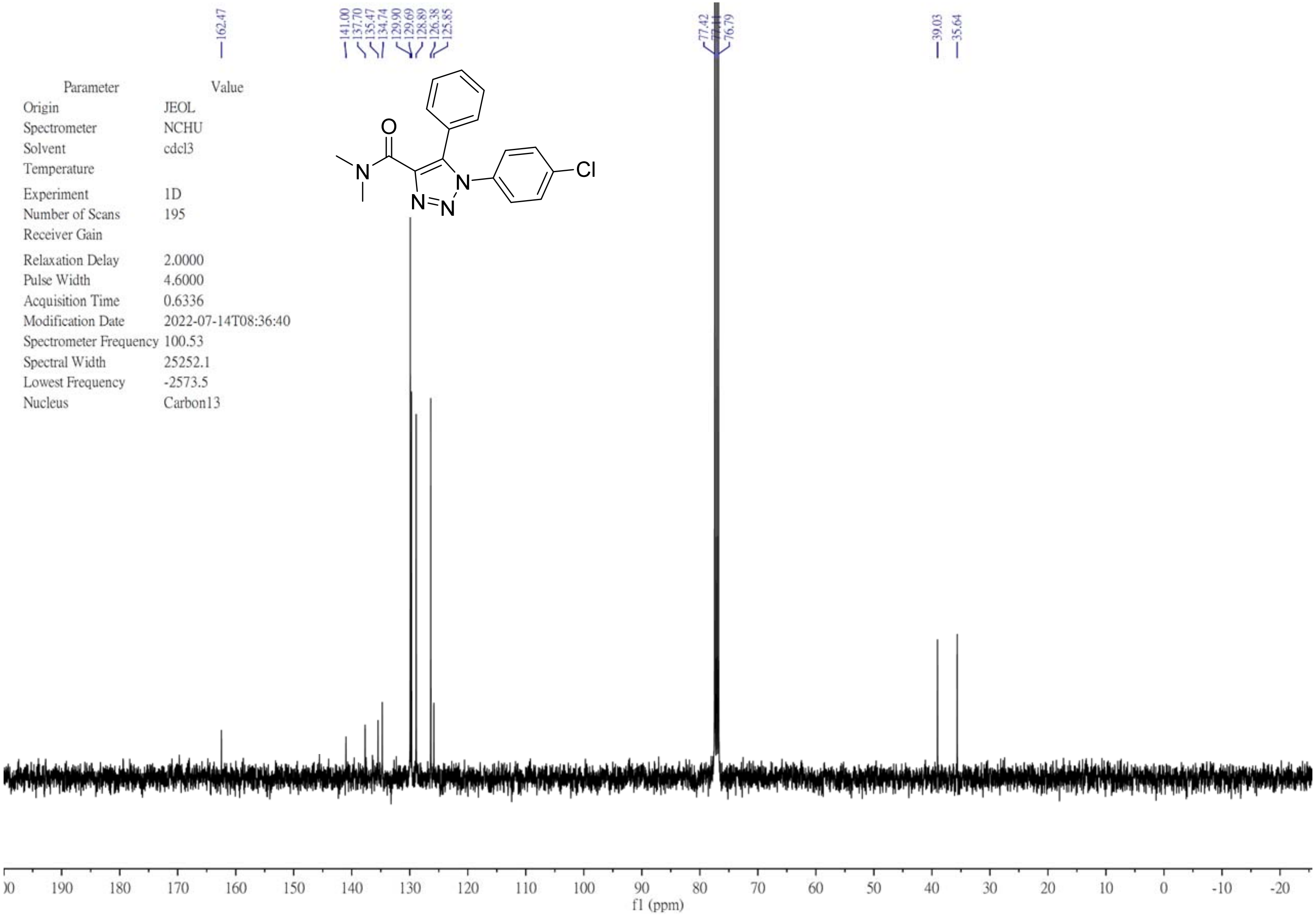

5gk $^{13}C\{^{1}H\}$ NMR spectrum (100 MHz in $CDCl_3$)

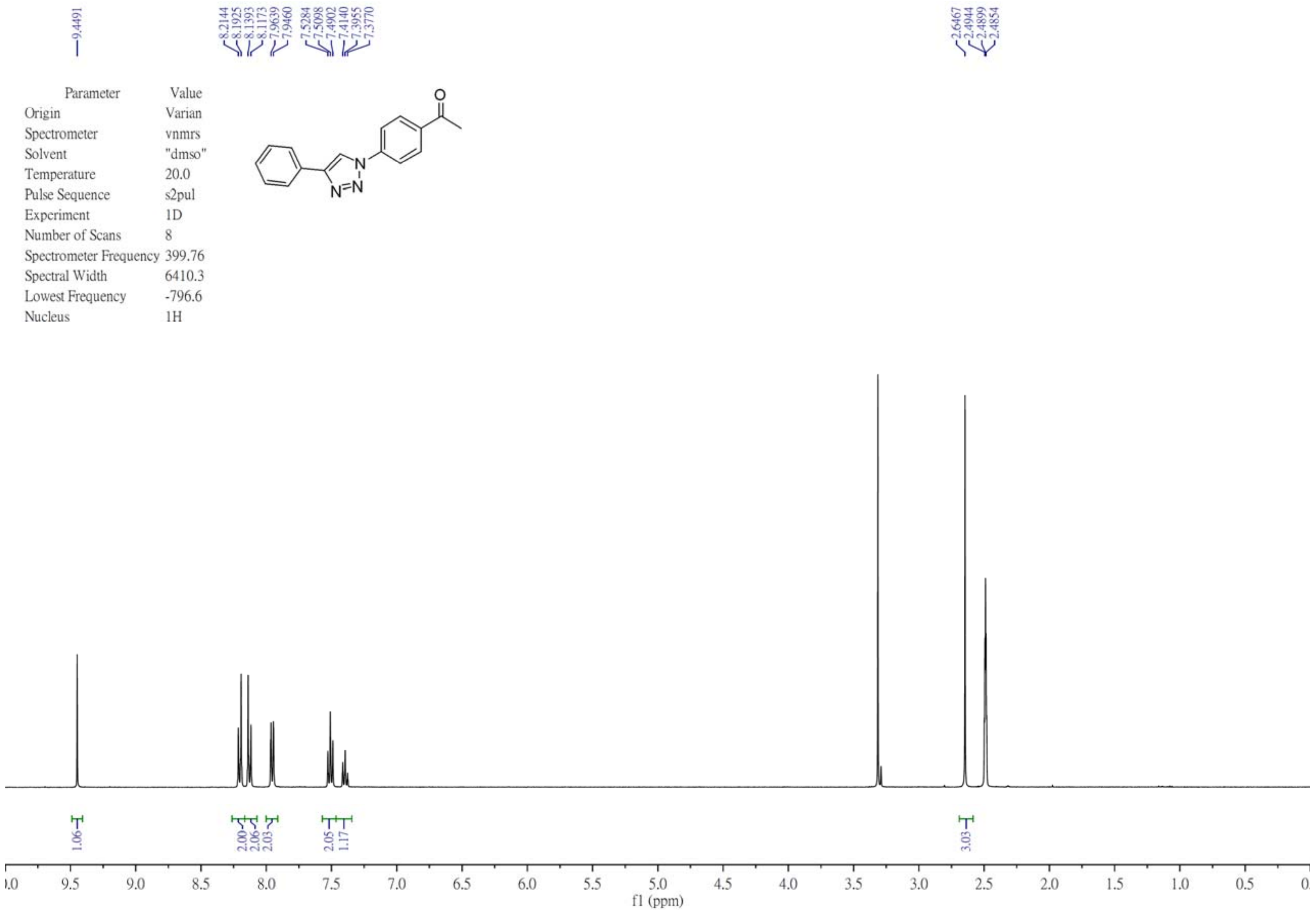

6 ^{1}H NMR spectrum (400 MHz in $(CD_3)_2SO$)

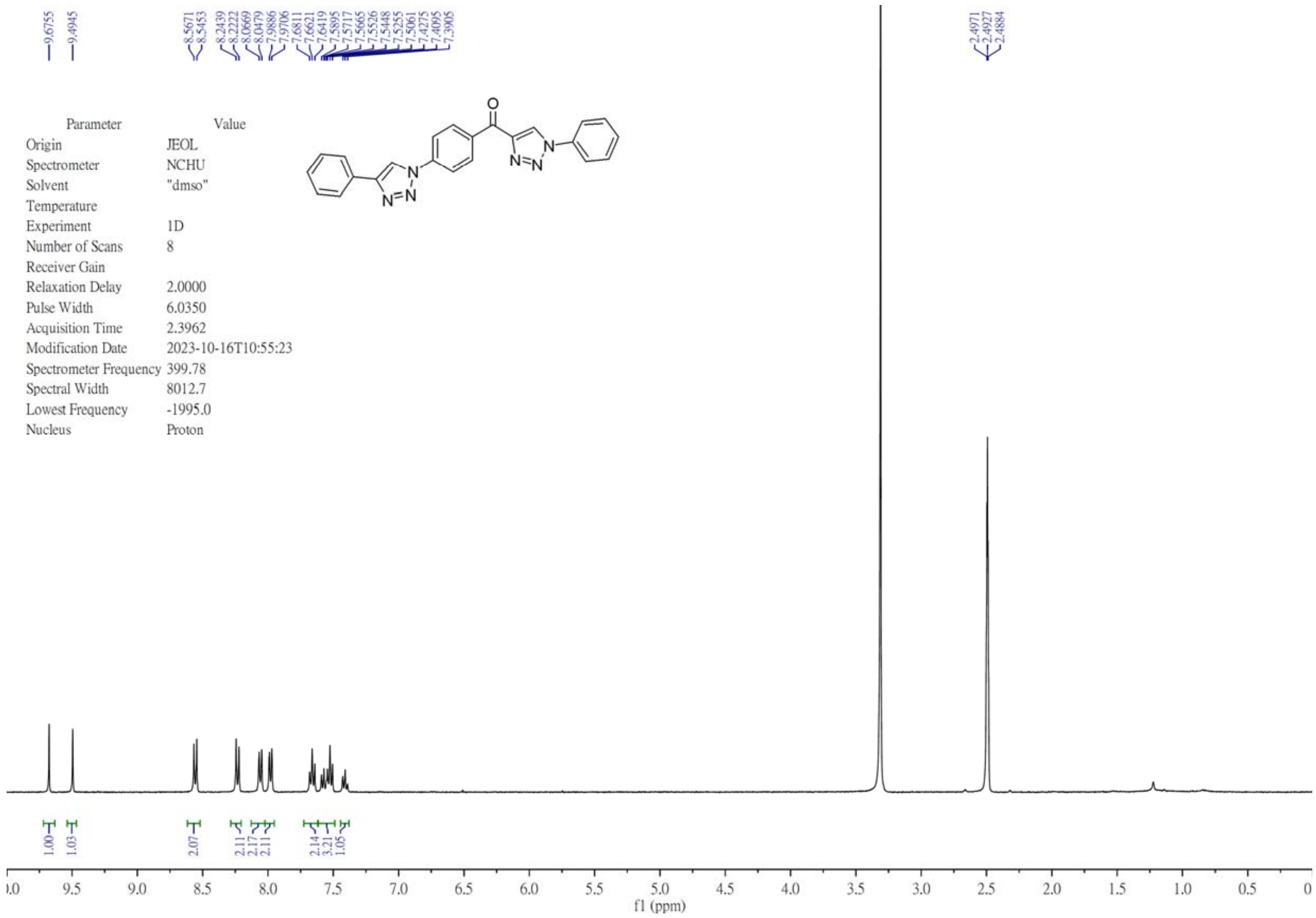

7 ^{1}H NMR spectrum (400 MHz in $(CD_3)_2SO$)

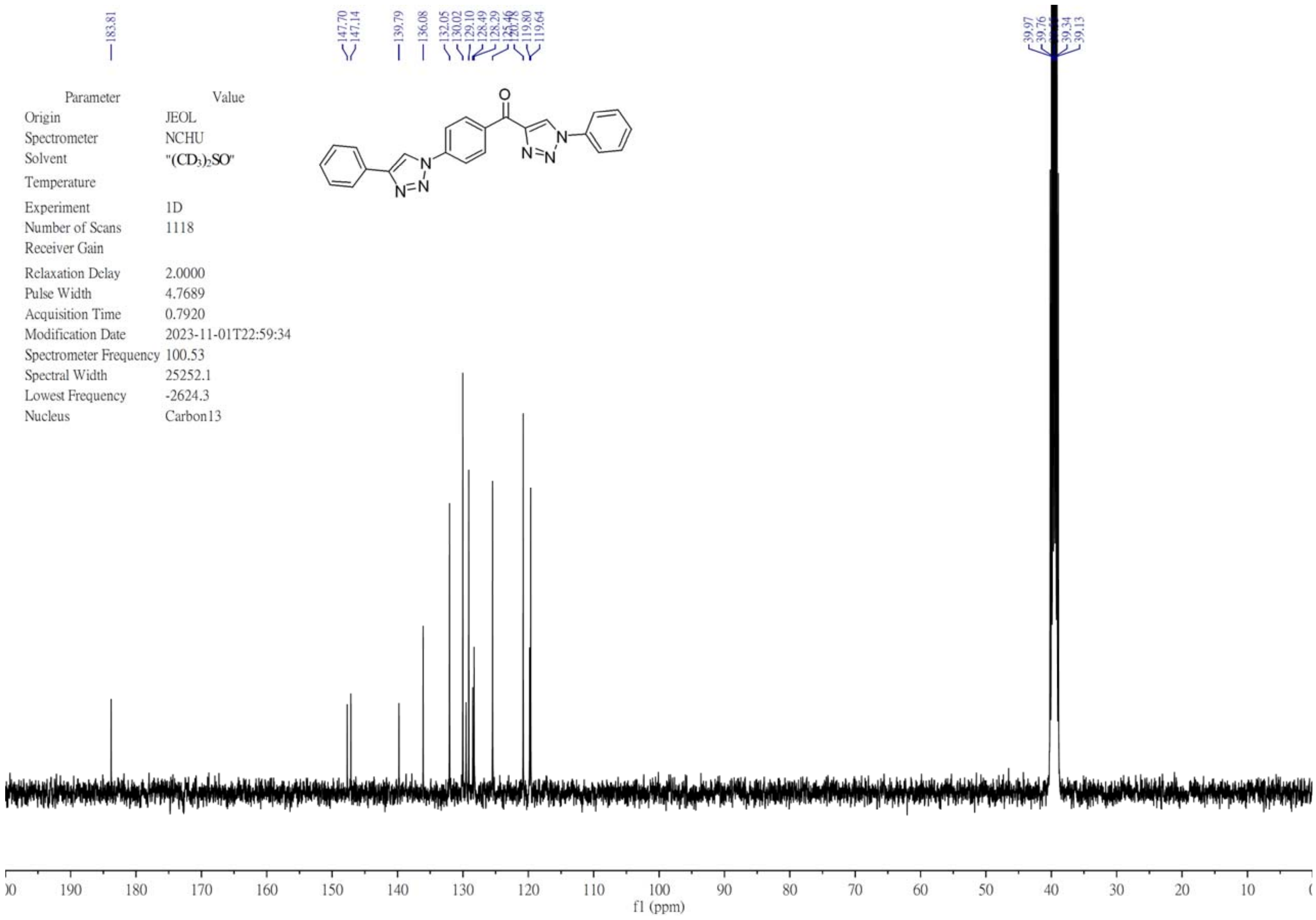

7 $^{13}C\{^1H\}$ NMR spectrum (100 MHz in $(CD_3)_2SO$)

Parameter	Value
Origin	Varian
Spectrometer	vnmrs
Solvent	"cdcl3"
Temperature	20.0
Pulse Sequence	s2pul
Experiment	1D
Number of Scans	40
Receiver Gain	50
Relaxation Delay	1.0000
Pulse Width	0.0000
Acquisition Time	2.5559
Modification Date	2024-01-02T21:04:00
Spectrometer Frequency	399.76
Spectral Width	6410.3
Lowest Frequency	-800.2
Nucleus	1H

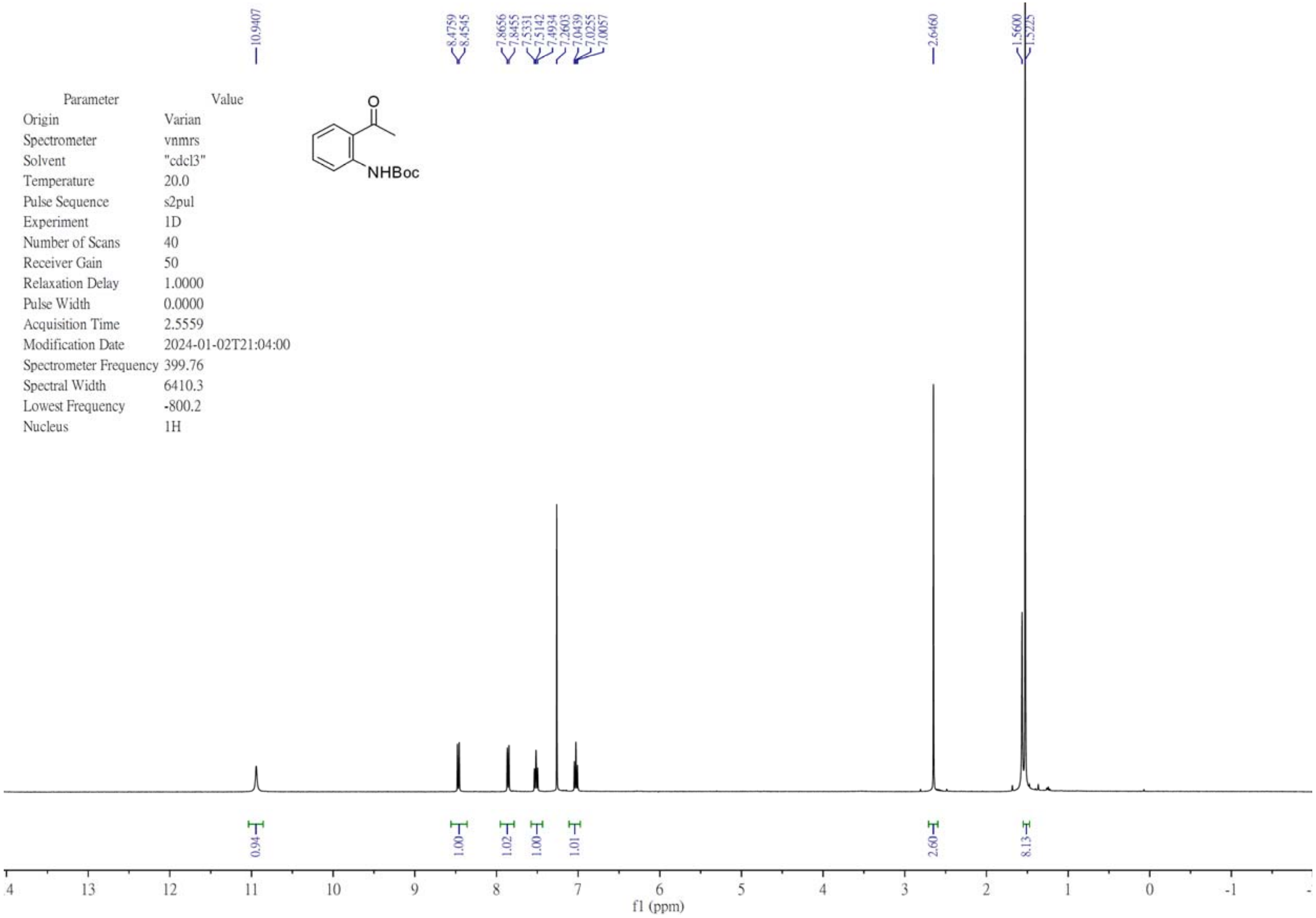

8 ^{1}H NMR spectrum (400 MHz in $CDCl_3$)

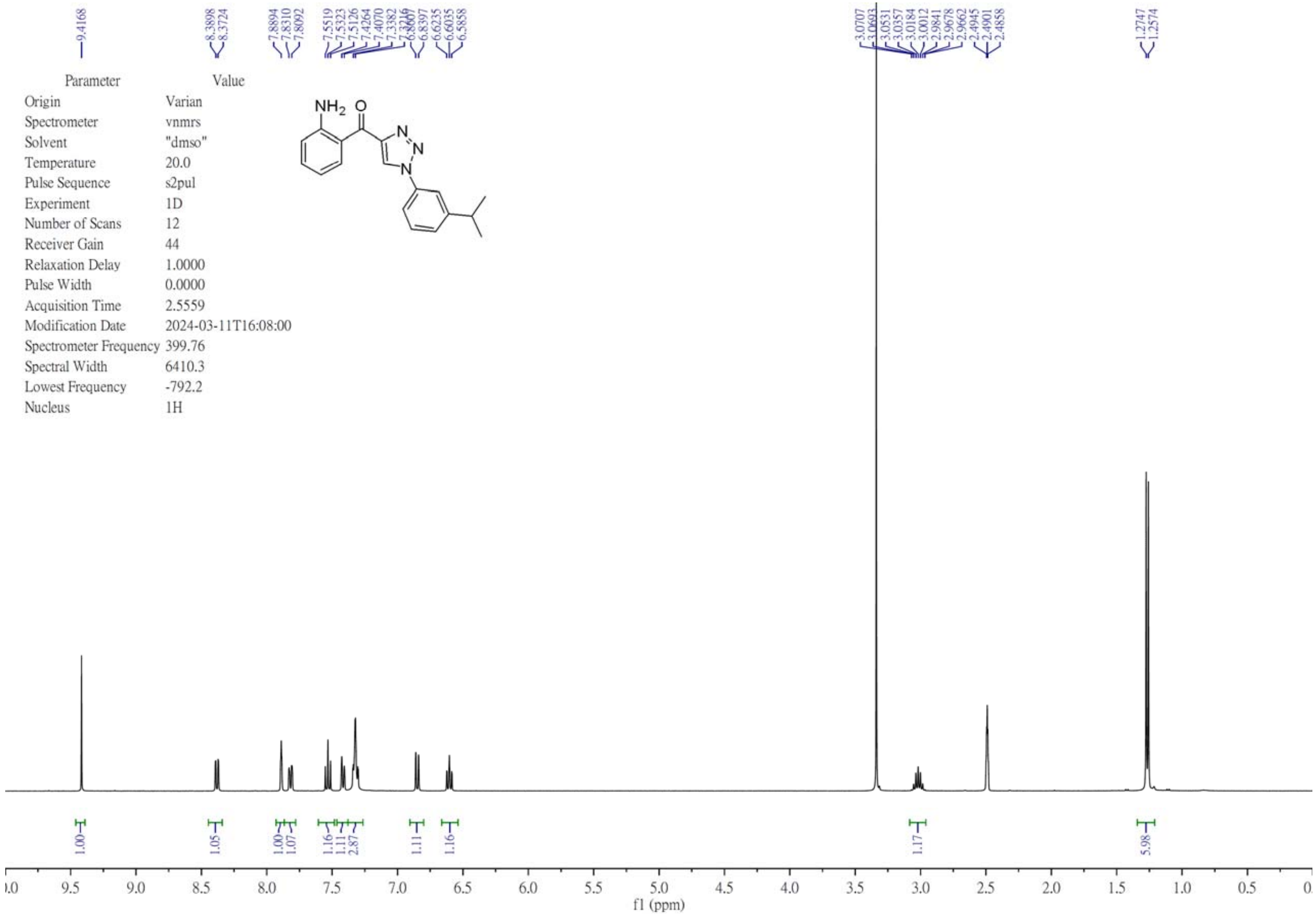

11 ^{1}H NMR spectrum (400 MHz in $(CD_3)_2SO$)

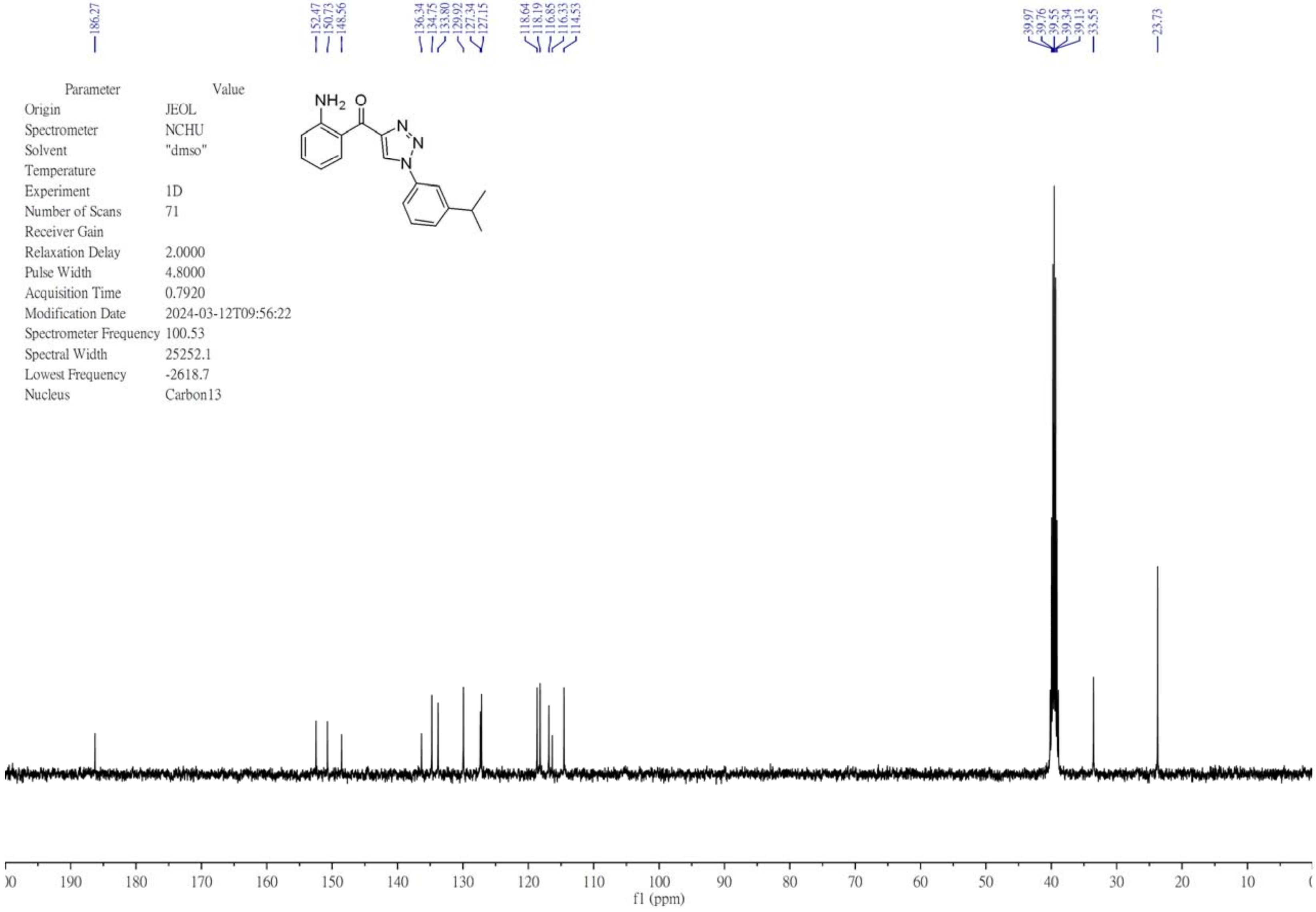

11 $^{13}C\{^{1}H\}$ NMR spectrum (100 MHz in $(CD_3)_2SO$)

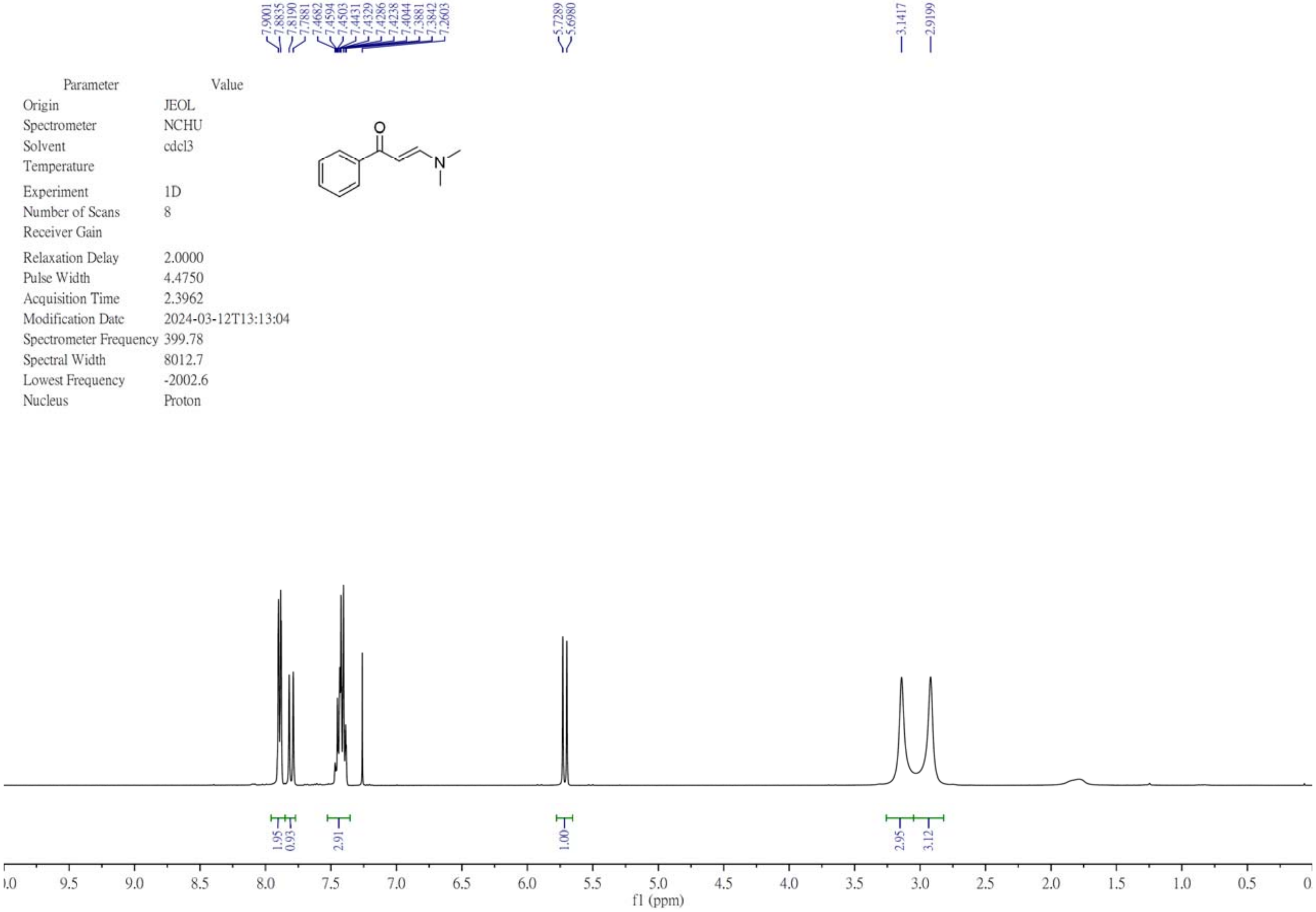

12 ^{1}H NMR spectrum (400 MHz in $CDCl_3$)

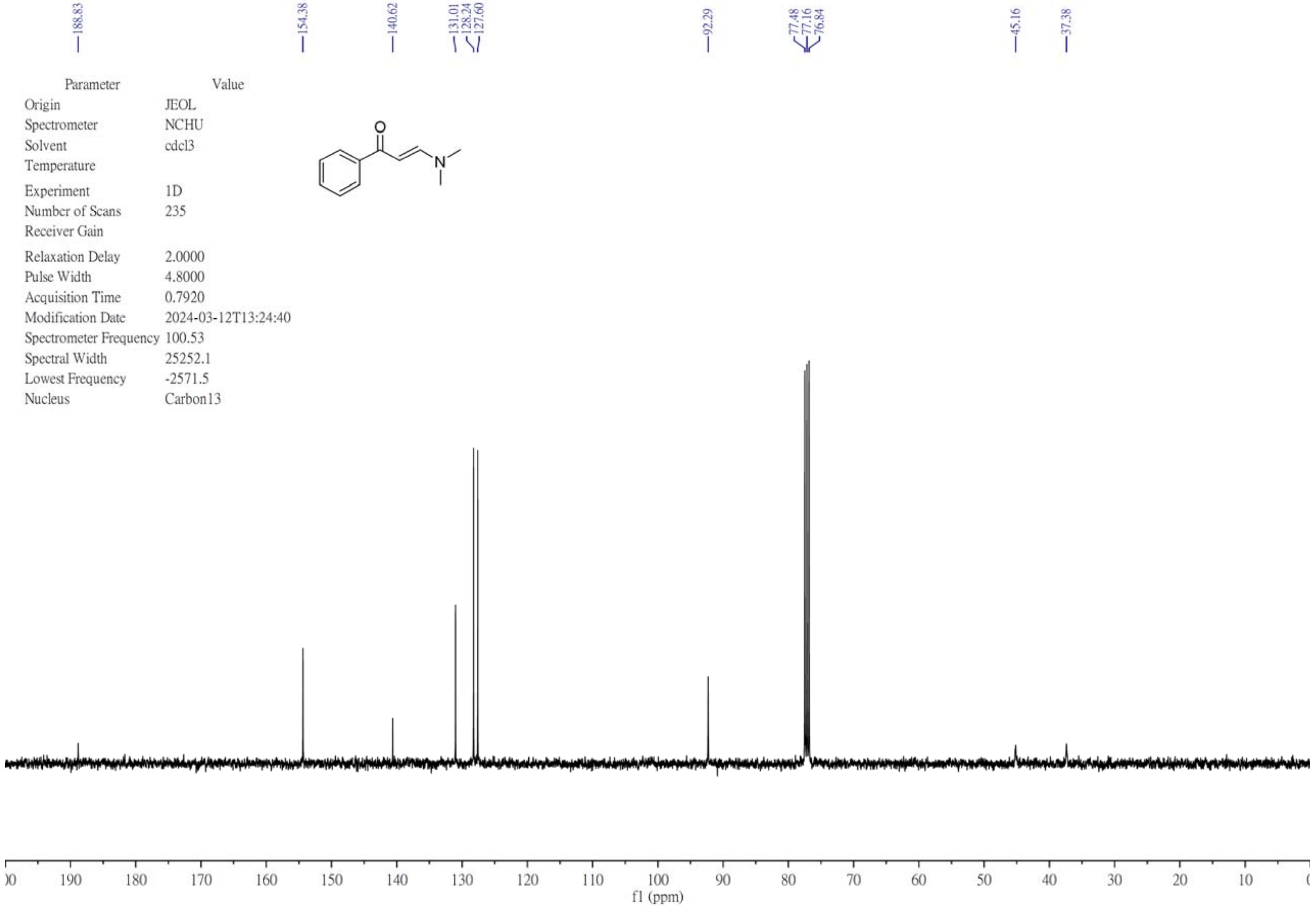

Parameter	Value
Origin	JEOL
Spectrometer	NCHU
Solvent	cdcl3
Temperature	
Experiment	1D
Number of Scans	235
Receiver Gain	
Relaxation Delay	2.0000
Pulse Width	4.8000
Acquisition Time	0.7920
Modification Date	2024-03-12T13:24:40
Spectrometer Frequency	100.53
Spectral Width	25252.1
Lowest Frequency	-2571.5
Nucleus	Carbon13

12 $^{13}C\{^{1}H\}$ NMR spectrum (100 MHz in $CDCl_3$)

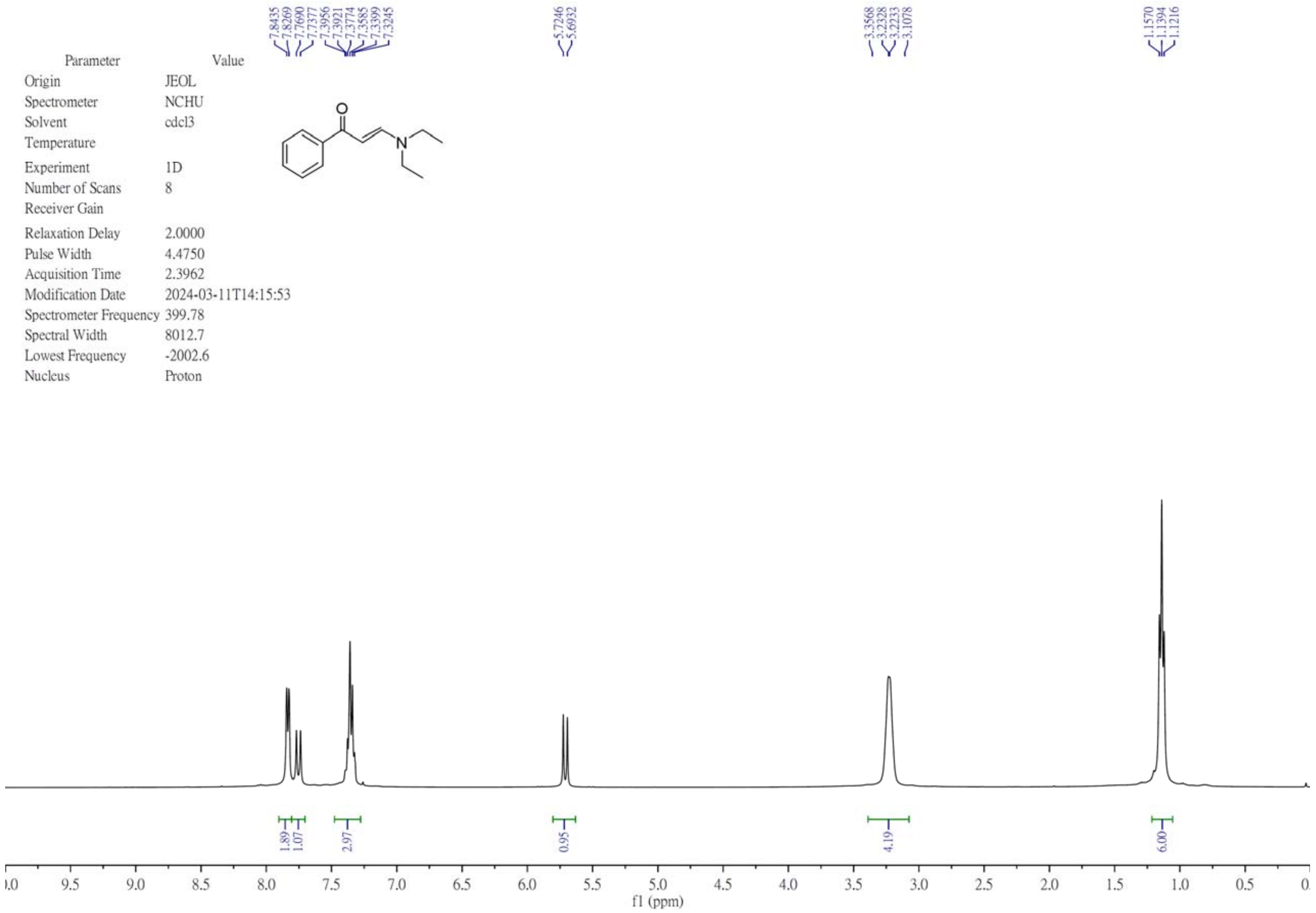

13 ^{1}H NMR spectrum (400 MHz in $CDCl_3$)

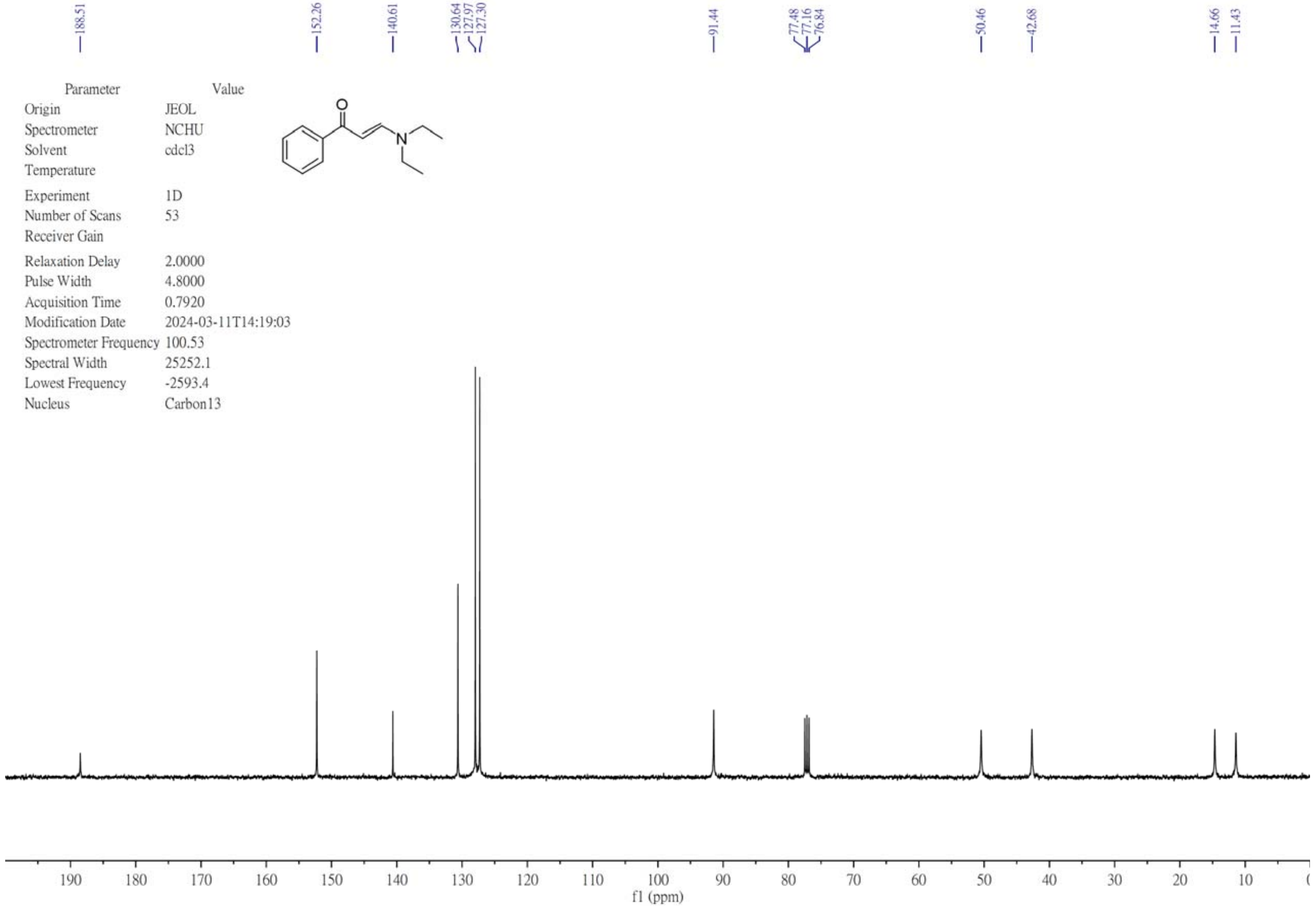

13 $^{13}C\{^{1}H\}$ NMR spectrum (100 MHz in $CDCl_3$)

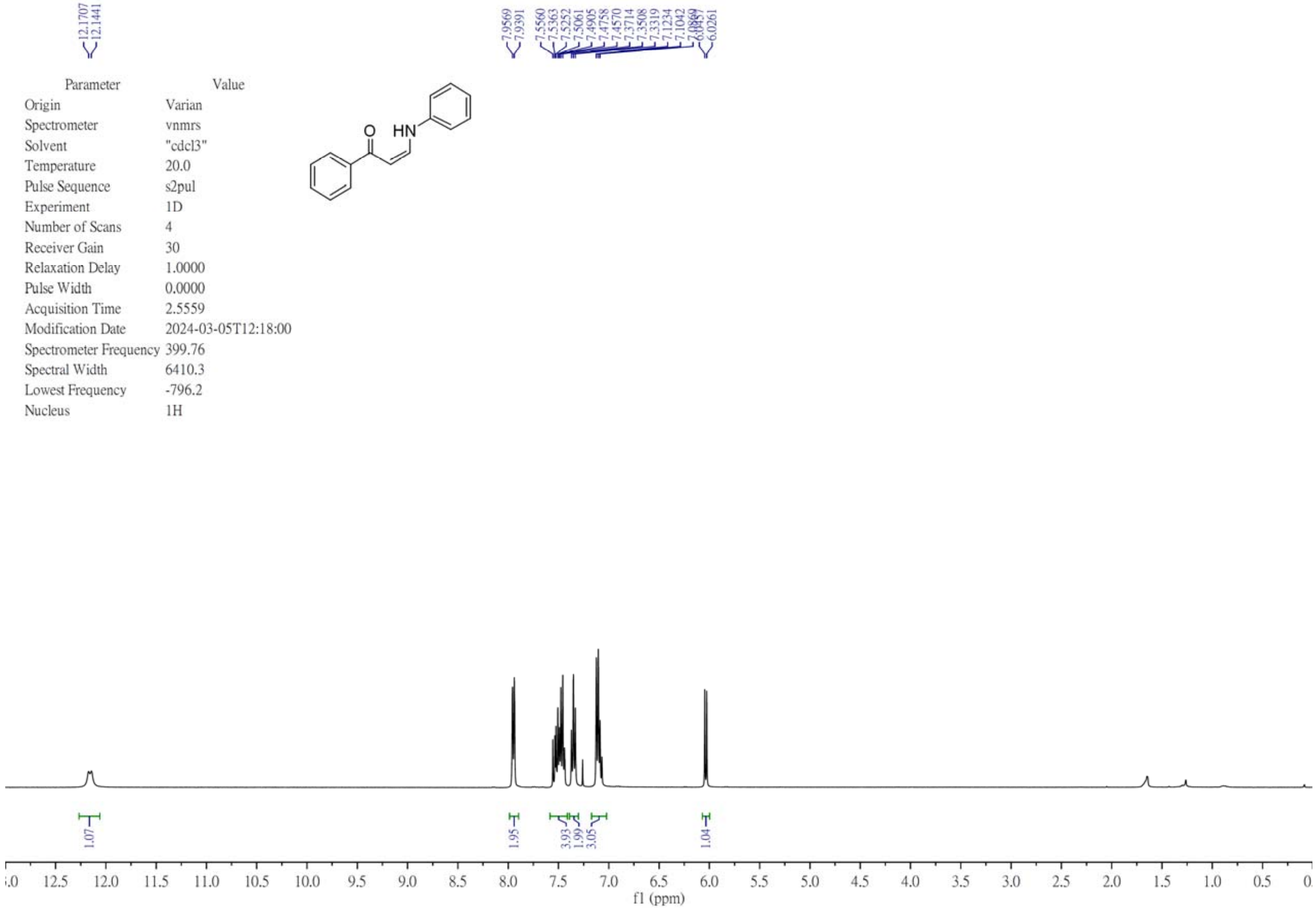

14 ^{1}H NMR spectrum (400 MHz in $CDCl_3$)

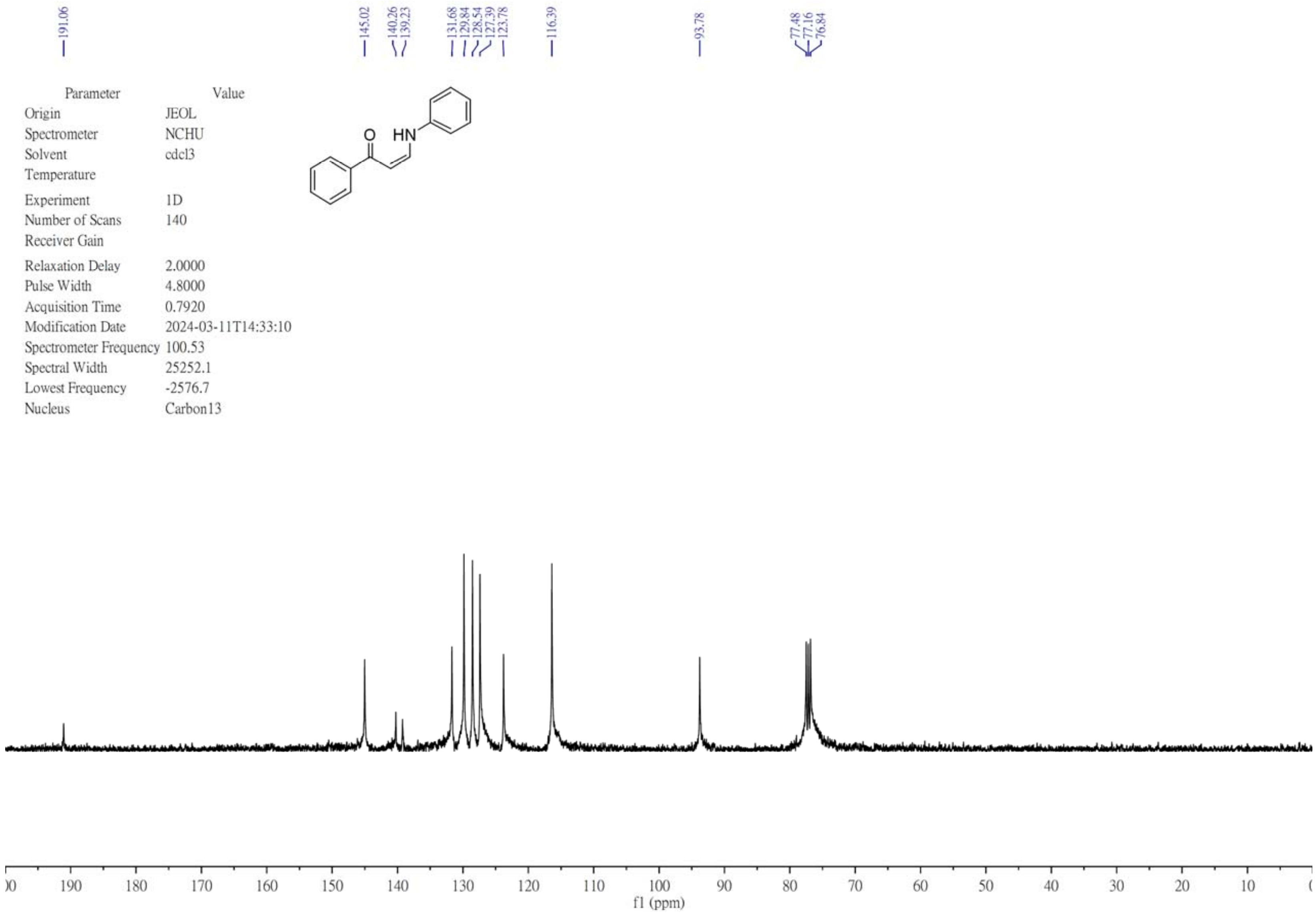

14 $^{13}C\{^{1}H\}$ NMR spectrum (100 MHz in $CDCl_3$)

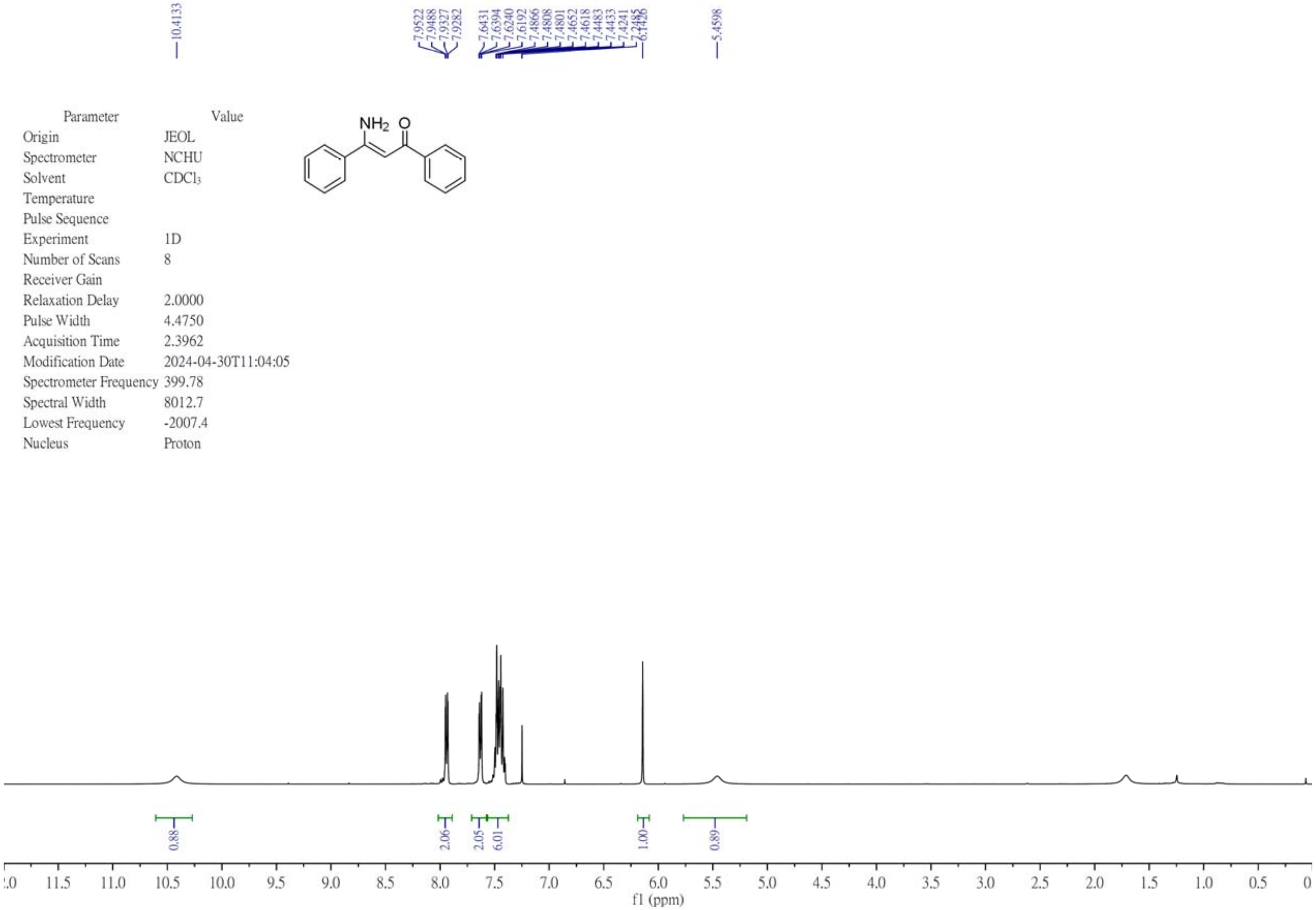

15 ^{1}H NMR spectrum (400 MHz in $CDCl_3$)

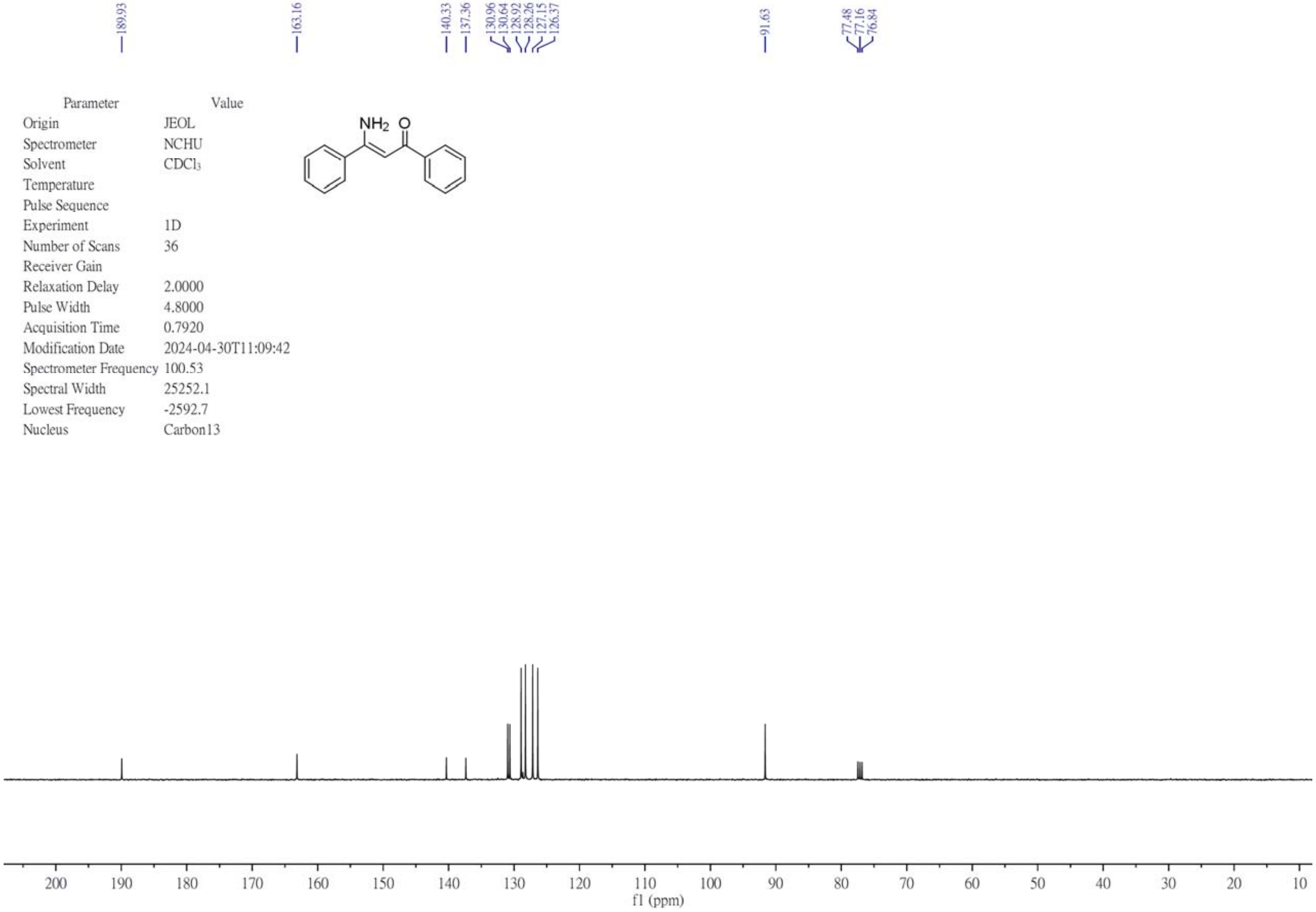

15 $^{13}C\{^{1}H\}$ NMR spectrum (100 MHz in $CDCl_3$)

Parameter	Value
Origin	Varian
Spectrometer	vnmrs
Solvent	"cdcl3"
Temperature	20.0
Pulse Sequence	s2pul
Experiment	1D
Number of Scans	16
Receiver Gain	44
Relaxation Delay	1.0000
Pulse Width	0.0000
Acquisition Time	2.5559
Modification Date	2024-07-31T23:24:00
Spectrometer Frequency	399.76
Spectral Width	6410.3
Lowest Frequency	-799.2
Nucleus	1H

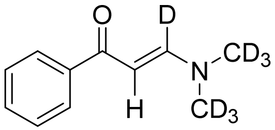

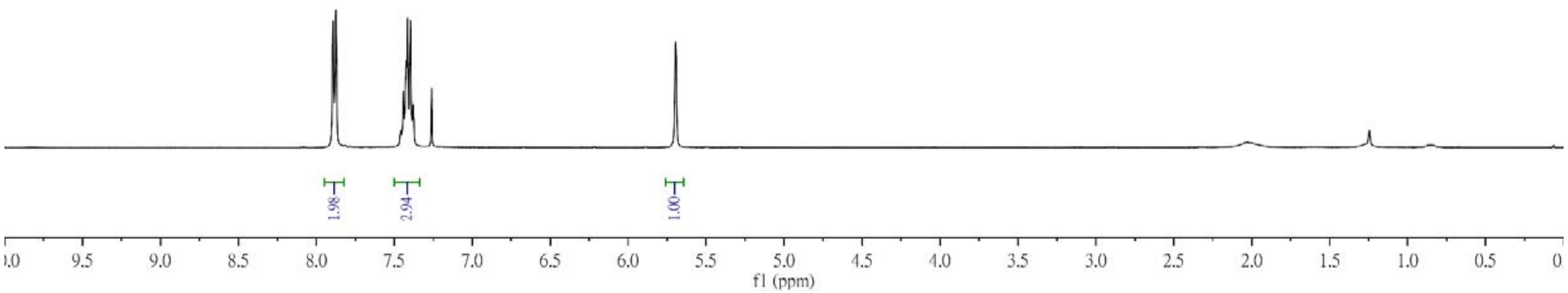

Deuterated enaminone ^{1}H NMR spectrum (400 MHz in $CDCl_3$)

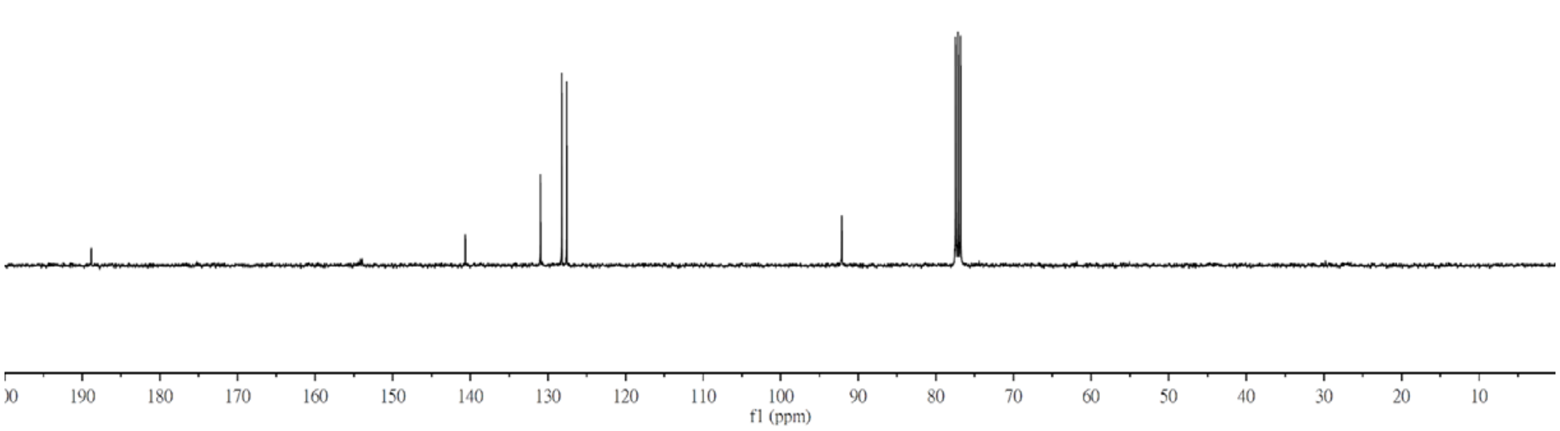

Parameter	Value
Origin	JEOL
Spectrometer	NCHU
Solvent	CDCl3
Temperature	
Experiment	1D
Number of Scans	884
Receiver Gain	
Relaxation Delay	2.0000
Pulse Width	4.8000
Acquisition Time	0.7920
Modification Date	2024-07-31T22:26:18
Spectrometer Frequency	100.53
Spectral Width	25252.1
Lowest Frequency	-2573.5
Nucleus	Carbon13

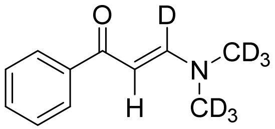

Deuterated enaminone $^{13}C\{^{1}H\}$ NMR spectrum (100 MHz in $CDCl_3$)

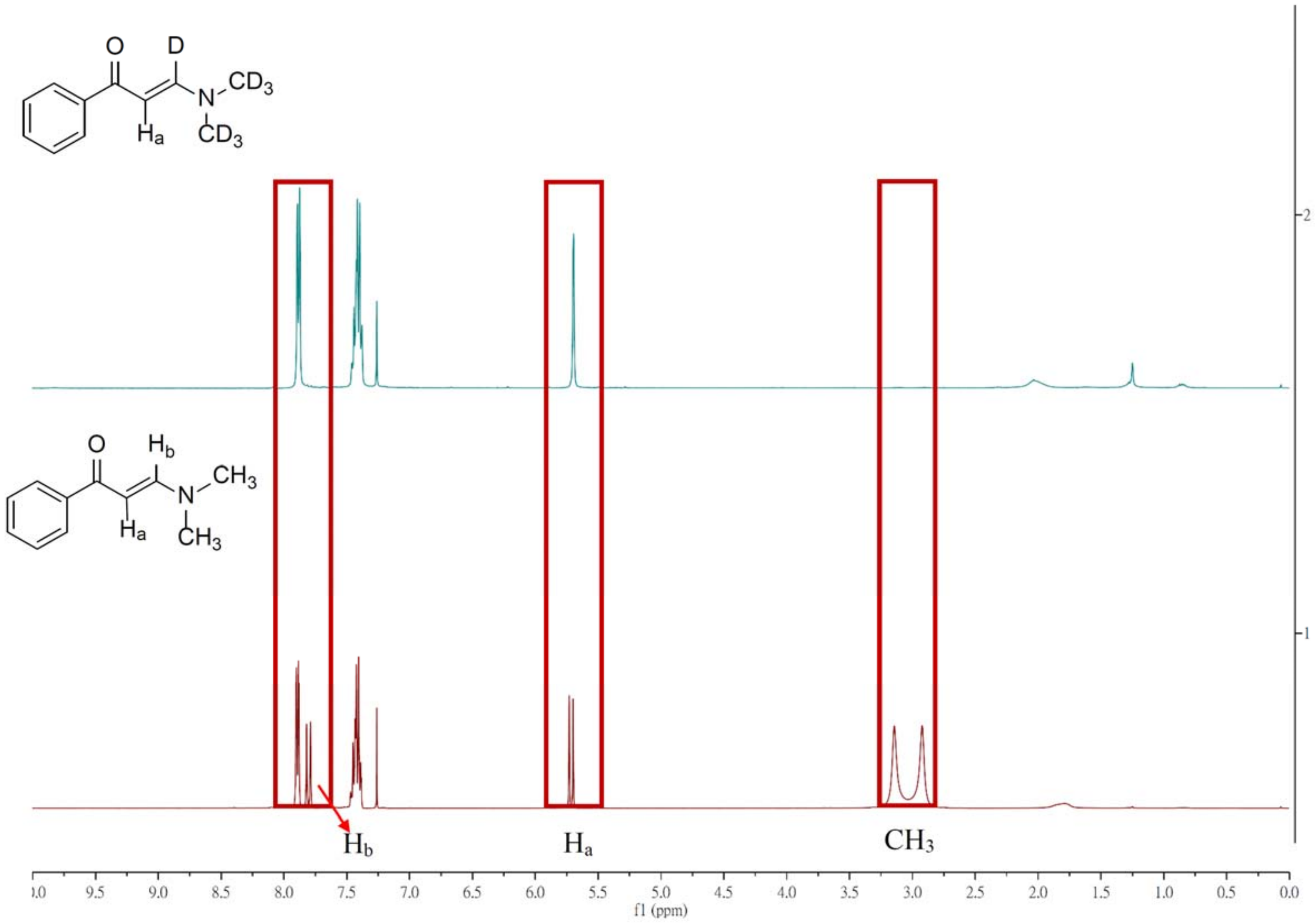

The combination ^{1}H spectrum of enaminone and deuterated enaminone
